# A more novel and powerful prognostic gene signature of lung adenocarcinoma determined from the immune cell infiltration landscape

**DOI:** 10.3389/fsurg.2022.1015263

**Published:** 2022-10-13

**Authors:** Chao Ma, Feng Li, Zhanfeng He, Song Zhao

**Affiliations:** Department of Thoracic Surgery, The First Affiliated Hospital of Zhengzhou University, Zhengzhou, China

**Keywords:** lung adenocarcinoma, LUAD, immune infiltration, ICI, tumor microenvironment, gene signature

## Abstract

**Background:**

Lung adenocarcinoma (LUAD) is the leading histological subtype of lung cancer worldwide, causing high mortality each year. The tumor immune cell infiltration (ICI) is closely associated with clinical outcome with LUAD patients. The present study was designed to construct a gene signature based on the ICI of LUAD to predict prognosis.

**Methods:**

Downloaded the raw data of three cohorts of the TCGA-LUAD, GSE72094, and GSE68465 and treat them as training cohort, validation cohort one, and validation cohort two for this research. Unsupervised clustering detailed grouped LUAD cases of the training cohort based on the ICI profile. The univariate Cox regression and Kaplan–Meier was adopted to identify potential prognostic genes from the differentially expressed genes recognized from the ICI clusters. A risk score-based prognostic signature was subsequently developed using LASSO-penalized Cox regression analysis. The Kaplan-Meier analysis, Cox analysis, ROC, IAUC, and IBS were constructed to assess the ability to predict the prognosis and effects of clinical variables in another two independent validation cohorts. More innovatively, we searched similar papers in the most recent year and made comprehensive comparisons with ours. GSEA was used to discover the related signaling pathway. The immune relevant signature correlation identification and immune infiltrating analysis were used to evaluate the potential role of the signature for immunotherapy and recognize the critical immune cell that can influence the signature's prognosis capability.

**Results:**

A signature composed of thirteen gene including ABCC2, CCR2, CERS4, CMAHP, DENND1C, ECT2, FKBP4, GJB3, GNG7, KRT6A, PCDH7, PLK1, and VEGFC, was identified as significantly associated with the prognosis in LUAD patients. The thirteen-gene signature exhibited independence in evaluating the prognosis of LUAD patients in our training and validation cohorts. Compared to our predecessors, our model has an advantage in predictive power. Nine well know immunotherapy targets, including TBX2, TNF, CTLA4, HAVCR2, GZMB, CD8A, PRF1, GZMA, and PDCD1 were recognized correlating with our signature. The mast cells were found to play vital parts in backing on the thirteen-gene signature's outcome predictive capacity.

**Conclusions:**

Collectively, the current study indicated a robust thirteen-gene signature that can accurately predict LUAD prognosis, which is superior to our predecessors in predictive ability. The immune relevant signatures, TBX2, TNF, CTLA4, HAVCR2, GZMB, CD8A, PRF1, GZMA, PDCD1, and mast cells infiltrating were found closely correlate with the thirteen-gene signature's power.

## Introduction

Lung cancer was estimated to account for about 1/4 of all cancer deaths in 2021 (1). Non-small cell lung cancer (NSCLC) accounts for 85% of all lung cancers, mainly including lung squamous cell carcinoma (LUSC) and lung adenocarcinoma (LUAD), of which LUAD is the most common subtype of NSCLC (1). Although surgery, radiotherapy, chemotherapy, and immunotherapy have made significant progress in coping with LUAD, the prognosis of LUAD patients is still unsatisfactory due to tumor cell invasion and adverse reactions during treatment (2). The prognosis of LUAD is related to many factors, such as TNM stage, tumor differentiation, and pathological subtypes. These factors are widely used to guide clinical decision-making but are still insufficient to accurately assess the prognosis of LUAD (2). Therefore, it is necessary to explore a clinically valid diagnostic and prognostic biomarker to understand the underlying molecular mechanisms of the occurrence and development of LUAD, and then to develop more promising individualized treatment strategies for LUAD.

Recently, the tumor microenvironment (TME) has attracted much attention. Accumulating evidence suggests that a comprehensive understanding of the molecular composition of LUAD requires a focus not only on tumor cells but also on the TME (3, 4). The TME is the environment surrounding the tumor, including surrounding blood vessels, immune cells, fibroblasts, signaling molecules, and the extracellular matrix (3, 4). Tumors are closely related to the surrounding microenvironment and constantly interact with each other. The TME contains multiple cell populations, signaling factors, and structural molecules that interact with tumor cells and support various stages of tumorigenesis (3, 4). Vast studies on TME have displayed that infiltrating immune cells play a key role in the development and progression of tumor and response to immunotherapy (5, 6). Recent research shows that tumor immune cell infiltration (ICI) is inextricably linked to the outcomes in lung cancer patients (7). A growing body of research suggests that LUAD can be regulated by interfering with inflammation and inflammatory signaling pathways (8). Understanding tumor growth, angiogenesis, and progression in the LUAD tumor microenvironment will contribute to developing more effective antitumor vaccines and other treatment strategies.

However, the detailed interaction between the immune cells infiltrating and LUAD outcomes has not been elucidated. Also, so far, no one has attempted to use ICI to construct a LUAD prognostic gene signature. The present work grouped patients using unsupervised clustering based on the ICI profile and dig into these ICI differentials for a prognostic model. More importantly, we tested the stability and broad applicability of our signature in independent cohorts and compared it with the findings obtained by our predecessors, which confirmed the superiority of our signature. At the end of the study, functional annotation analysis, immune signature correlation analysis, and 22 tumor-infiltrating immune cells (TICs) analysis revealed the potential functions and targets of our signatures in detail.

## Materials and methods

### Mining public databases

The Cancer Genome Atlas (TCGA) is a project, begun in 2005, to catalogue genetic mutations responsible for cancer, using genome sequencing and bioinformatics (9). The project TCGA-LUAD contains LUAD samples and their accordingly clinical data. We obtained the level 3 gene expression and clinical data of patients in the project TCGA-LUAD on the GDC Xena platform (https://gdc.xenahubs.net). We searched the GEO database (10) (https://www.ncbi.nlm.nih.gov/geo/) using the keyword “lung adenocarcinoma” and looked for the potential candidates using the following screening criteria: (1) total RNA expression data is available; (2) total LUAD cases that contain survival data are greater than 390. Finally, datasets named GSE72094 and GSE68465 were selected, and their gene expression data and clinical characteristics were acquired from the GEO portal. For detail, the dataset GSE72094 was analyzed on the GPL15048 platform (Rosetta/Merck Human RSTA Custom Affymetrix 2.0 microarray, HuRSTA_2a520709.CDF), and the dataset GSE68465 was performed on the GPL96 platform (HG-U133A, Affymetrix Human Genome U133A Array). For the datasets selected above, we only collected the tumor sample that contain gene expression data and survival data for our study. The eligible patients in the TCGA-LUAD, GSE72094, and GSE68465 were gathered into and taken as training cohort, validation cohort one, and validation cohort two, respectively.

### Consensus clustering for tumor-infiltrating immune cells and differentially expressed genes (DEGs) identification

CIBERSORT is an analytical tool in the Alizadeh laboratory developed by Newman et al. (11). This tool has been extensively studied and reported. It can estimate the abundance of member cell types in mixed cell populations using only gene expression data (11). Tumor-infiltrating leukocytes are immune cells surrounding tumor cells. Studies have shown that tumor-infiltrating leukocytes have properties that predict survival outcomes in different cancers (12). Developed by Kosuke Yoshihara and colleagues, the ESTIMATE algorithm uses gene expression data to output estimated levels of infiltrating stromal and immune cells and estimated tumor purity (13). CIBERSORT immune fractions and leukocyte fractions of the training cohort were download from one previous published study (14). We adopt the “ESTIMATE” R package to estimate the immune and stromal score for each case in training cohort. We merged the CIBERSORT immune fractions, leukocyte fraction, and ESTIMATE scores of each LUAD case as its the ICI pattern. Subsequently, the LUAD cases were clustered by the “ConsensusClusterPlus” R package developed by Matthew D. Wilkerson (15), the repeat time was set to “1,000”, the clustering algorithm was set to “Pam”, and the distance measure was set to “Euclidean distances”. Based on the ICI subgroups generated by the above analysis, we called the “limma” R package to identify the DEGs between different ICI subgroups, and the operating parameters were set to |log2(fold-change)| > 0.2 and *p*-value < 0.05.

### Construction and validation of the prognosis model

We evaluated the prognostic performance of DEGs by a univariate Cox regression analysis (*via* the “survival” R package) in the training cohort (*p*-value < 0.05). The LASSO is a regression analysis method that performs both variable selection and regularization to enhance the prediction accuracy and interpretability of the resulting statistical model. We deployed the LASSO Cox regression for constructing a prognostic model. In the actual operation, we called the “glmnet” R package to perform the LASSO algorithm to select, shrink, and identify potential prognostic genes (16–19). Ten-fold cross-validation was applied to tune the optimal value of the penalty parameter. The R package would be outputted the selected genes with coefficients. The formula for computing the risk score is as follows (n: hub gene, Expi: expression level, *β*i: coefficient):

$$Risk\;score=\overset{n}{\underset{i}{\sum }}Expi\;*\;\beta i$$

Patients in each studied cohort were divided into high/low risk groups according to the median risk score. Kaplan–Meier survival curve analysis was used to compare the overall survival of the two groups, and univariate and multivariable Cox analyses were used to evaluate the predictive value of gene signature. Kaplan–Meier plots were constructed by the integration of the “survival” and “survminer” R packages. Cox models, including univariate and multivariable were built *via* the “survival” R package. In addition, the ROC (20), integrated AUC (IAUC) (20) and integrated Brier score (IBS) (21) were performed for confirming the signature's prognosis capability *via* the R packages of “timeROC”, “survival” “Rcpp”, “ranger”, and “survival”.

### Comparison of our gene signature with previously published ones

To compare whether our findings are good or bad compared to our predecessors, we looked for candidate research from PubMed (https://pubmed.ncbi.nlm.nih.gov/) to compare. In detail, we used the search module of PubMed and filtered the results with the following criteria: (1) the keyword “gene signature prognosis lung adenocarcinoma”; (2) according to Clarivate Journal Citation Reports Year 2020, screened out studies with an impact factor greater than 6; (3) The publication date of the article was from September 1, 2020 to September 1, 2021; (4) Candidate genes and coefficients were clearly listed in the study. We extracted the gene signatures and coefficients from the found candidate studies and applied them to our study cohorts, calculating risk scores for LUAD cases as described above. Kaplan-Meier analysis and Cox models were used to assess the predictive power of each study for LUAD prognosis.

### Gene set enrichment analysis (GSEA)

In our study, Hallmark gene set collections (22) (v7.4, https://www.gsea-msigdb.org/gsea/downloads.jsp) were selected as the background for the GSEA. We conducted this analysis between the high- and low-risk LUAD group of the training cohort to uncover the signature's prospective mechanisms in tumor outcomes. In the GSEA analysis, | Normalized Enrichment Score (NES) | > 1, nominal (NOM) *p*-value < 0.05, and FDR *q*-value < 0.2 were used as cutoffs.

### Correlations between gene signature and immune relevant signatures

Immune checkpoints are modulators of immune activation. They play a vital role in maintaining immune homeostasis and preventing autoimmunity. In cancer, the immune checkpoint mechanism is often activated to suppress the developing anti-tumor immune response. For assessing the immune therapy potential of our signature, we selected CD274 (23), CTLA4 (24), HAVCR2 (25), IDO1 (26), LAG3 (27), PDCD1 (28), CD8A (29), CXCL10 (30), CXCL9 (31), GZMA (32), GZMB (33), IFNG (34), PRF1 (35), TBX2 (36), and TNF (37) from previous studies as immune relevant signatures, and used the Spearman coefficient and Wilcoxon rank-sum to measure the correlations.

### Determine the relationships between our signature and 22 TICs

The Spearman’s coefficient and Wilcoxon rank-sum test were used to analyze the relationship between the 22 TICs and the obtained signature of this study. Univariate Cox model, multivariable Cox model, and Kaplan-Meier analysis were used to assess the prognostic power of 22 TICs. Combining the above results can determine the TIC that affects the prognosis of the gene signature.

## Results

### Patient characteristics

**Figure 1:**
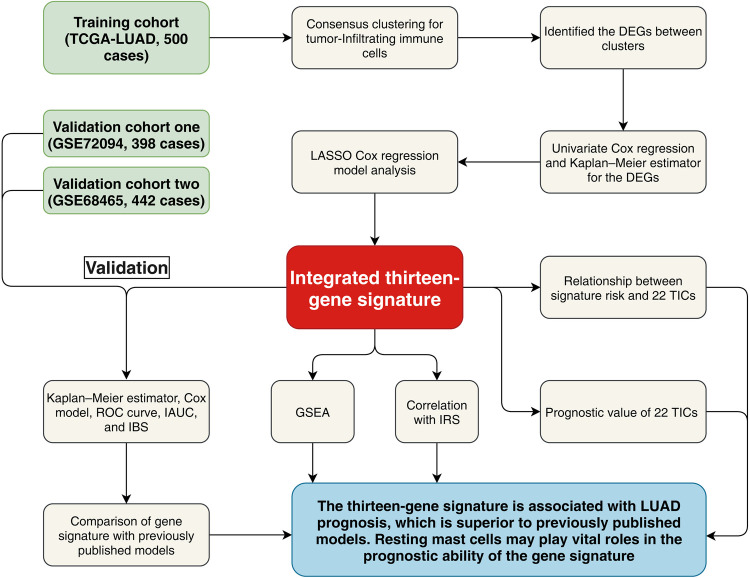
Research flow chart. TCGA, The Cancer Genome Atlas; LUAD, lung adenocarcinoma; LASSO, least absolute shrinkage and selection operator Cox regression model; DEGs, differentially expressed genes; ROC, receiver operating characteristic; AUC, Area under the ROC curve; IBS, integrated Brier score; IAUC, integrated AUC; IRS, immune relevant signature; GSEA, Gene Set Enrichment Analysis; TICs, tumor-infiltrating immune cells.

Figure 1 displays the analysis procedures of this study. We collected RNA sequencing profiles of 500 LUAD samples from the TCGA database, and treated them as the training cohort. For the validation purpose, 398 LUADs from the GSE72094 and 442 LUADs from the GSE68465 were selected as the validation cohort one and validation cohort two, repetitively. The detailed clinicopathological parameters were selected for subsequent analysis and shown in Table 1.

**Table 1:** Clinical characteristics of patients involved in the study.

Characteristics	Training cohort TCGA-LUAD, *n* = 500)	Validation cohort one (GSE72094, *n* = 398)	Validation cohort two (GSE68465, *n* = 442)
Age			
<65	219 (43.8%)	107 (26.88%)	214 (48.42%)
>=65	271 (54.2%)	291 (73.12%)	228 (51.58%)
Unknown	10 (2%)	0	0
Gender			
Female	270 (54%)	222 (55.78%)	219 (49.55%)
Male	230 (46%)	176 (44.22%)	223 (50.45%)
Race			
White	386 (77.2%)	377 (94.72%)	294 (66.52%)
Non-White	60 (12%)	18 (4.52%)	19 (4.29%)
Unknown	54 (10.8%)	3 (0.75%)	129 (29.19%)
Ethnicity			
Hispanic or Latino	7 (1.4%)	9 (2.26%)	NA
Non-Hispanic or Latino	381 (76.2%)	381 (95.73%)	NA
Unknown	112 (22.4%)	8 (2.01%)	NA
Tumor stage			
Stage I	268 (53.6%)	254 (63.82%)	NA
Stage II	119 (23.8%)	67 (16.83%)	NA
Stage III	80 (16%)	57 (14.32%)	NA
Stage IV	25 (5%)	15 (3.77%)	NA
Unknown	8 (1.6%)	5 (1.26%)	NA
T classification			
T1	167 (33.4%)	NA	150 (33.94%)
T2	267 (53.4%)	NA	251 (56.79%)
T3	45 (9%)	NA	28 (6.33%)
T4	18 (3.6%)	NA	11 (2.49%)
Unknown	3 (0.6%)	NA	2 (0.45%)
Prior malignancy			
Yes	79 (15.8%)	NA	NA
No	421 (84.2%)	NA	NA
Tissue origin			
Upper lobe lung	291 (58.2%)	NA	NA
Non-upper lobe lung	209 (41.8%)	NA	NA
Smoking history			
Ever	415 (83%)	300 (75.38%)	300 (67.87%)
Never	71 (14.2%)	31 (7.79%)	49 (11.09%)
Unknown	14 (2.8%)	67 (16.83%)	93 (21.04%)
KRAS mutation			
Yes	NA	139 (34.92%)	NA
No	NA	259 (65.08%)	NA
TP53 mutation			
Yes	NA	97 (24.37%)	NA
No	NA	301 (75.63%)	NA
EGFR mutation			
Yes	NA	41 (10.3%)	NA
No	NA	357 (89.7%)	NA
STK11 mutation			
Yes	NA	64 (16.08%)	NA
No	NA	334 (83.92%)	NA
Vital status			
Alive	318 (63.6%)	285 (71.61%)	206 (46.61%)
Dead	182 (36.4%)	113 (28.39%)	236 (53.39%)

### Two clustering found in the ICI landscape

**Figure 2:**
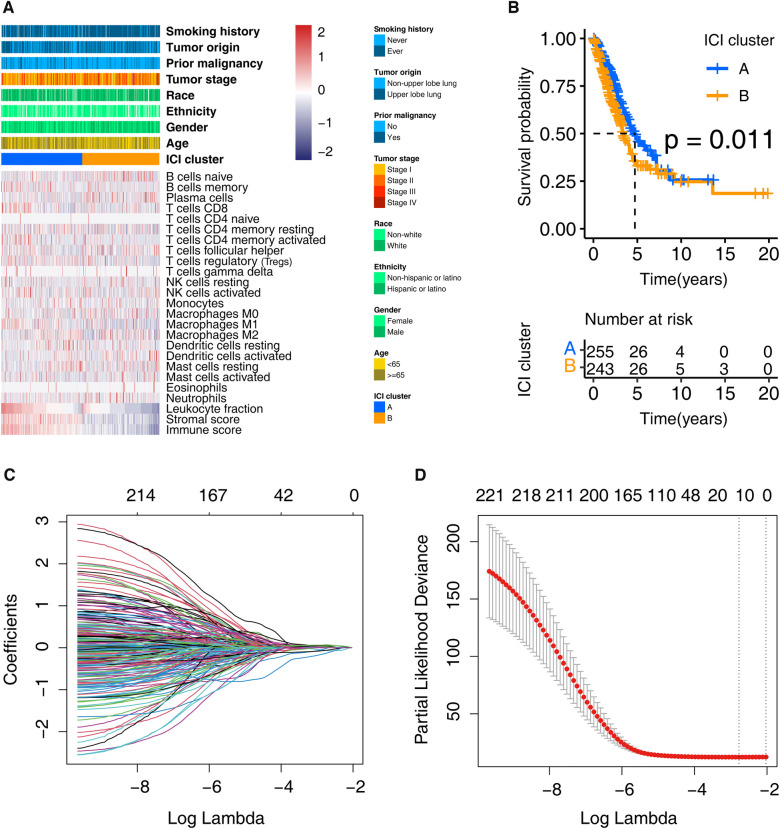
Clustering the LUADs based on the profile of ICI and identifying the prognosis signature. (**A**) LUADs in the training cohort were grouped into two clusters. Each row represents one ICI type, and each column indicates one LUAD case. (**B**) Kaplan-Meier analysis shows that the survival of these two clusters is statistically significant, that is, *p* = 0.011. (**C**) Ten-time cross-validation for tuning parameter selection in the LASSO model. (**D**) Partial likelihood deviation map with Lasso approach. ICI, immune cell infiltration; LUAD, lung adenocarcinoma.

We discovered two ICI subtypes (Supplementary Material Figure S1; Figure 2A) using the ICI profiles. Notably, the Kaplan-Meier analysis detected survival differences (log-rank test, *p* = 0.011; Figure 2B) exist between them. As shown in the figure, the cluster A was related to a promising overall survival, whereas the cluster B proved an unfavorable prognosis. The “limma” R package recognized 6015 DEGs between the two ICI clusters (Supplementary Material Table S1).

### A thirteen-gene prognostic signature found

First, we obtained 222 potential prognostic genes in the training cohort patients by univariate Cox regression and Kaplan–Meier analysis performed on the 6,015 DEGs (Supplementary Material Table S2). Then the LASSO analysis was carried out using the 222 genes for in-depth shrinkage and selection. 13 of the 222 candidate genes (ABCC2, CCR2, CERS4, CMAHP, DENND1C, ECT2, FKBP4, GJB3, GNG7, KRT6A, PCDH7, PLK1, and VEGFC) retained their prognostic significance and may thus impact the prognosis of LUAD (Figures 2C,D). Table 2 displays the 13 candidate genes' coefficients.

**Table 2:** Thirteen prognostic genes generated from the LASSO Cox regression.

Gene	Description	Coefficient
ABCC2	ATP Binding Cassette Subfamily C Member 2	0.021348192
CCR2	C-C Motif Chemokine Receptor 2	−0.137788931
CERS4	Ceramide Synthase 4	−0.064587353
CMAHP	Cytidine Monophospho-N-Acetylneuraminic Acid Hydroxylase, Pseudogene	−8.78E-05
DENND1C	DENN Domain Containing 1C	−0.023165087
ECT2	Epithelial Cell Transforming 2	0.000766385
FKBP4	FKBP Prolyl Isomerase 4	0.027608786
GJB3	Gap Junction Protein Beta 3	0.060365957
GNG7	G Protein Subunit Gamma 7	−0.062917285
KRT6A	Keratin 6A	0.015492812
PCDH7	Protocadherin 7	0.010744541
PLK1	Polo Like Kinase 1	0.06452668
VEGFC	Vascular Endothelial Growth Factor C	0.044307863

### Confirmation of the prognostic capacity of the thirteen-gene signature

To further elucidate the relationship between risk scores and survival status in LUAD patients, we generated risk curves and survival scatterplots for the training cohort, validation cohort one, and validation cohort two (Supplementary Material Figure S2). The results showed that mortality increased with increasing risk scores. A heatmap of the expression levels of thirteen identified genes in LUAD showed their expression pattern in the LUAD population (Supplementary Material Figure S2). As exhibited in Supplementary Material Figure S3, GNG7, DENND1C, CCR2, CERS4, and CMAHP were found associated with promising prognoses for LUADs, while KRT6A, ABCC2, GJB3, PCDH7, VEGFC, PLK1, ECT2, and FKBP4 were associated with poor outcomes.

**Figure 3:**
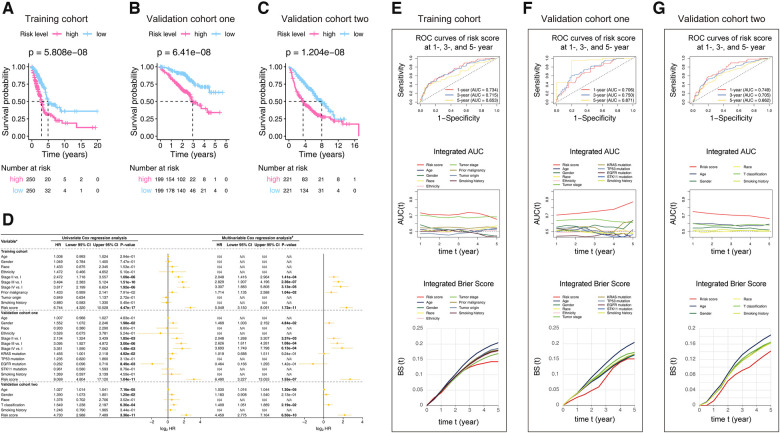
Kaplan-Meier analysis (**A–C**), Cox models (**D**), ROC, iAUC, and IBS (**E–G**) constructed in the studied cohorts. (**A–C**) Patients in each cohort were divided into low-risk groups and high-risk groups based on their median risk score. The log-rank test with a *p*-value < 0.05 suggests the survival difference is significant. The lower part displays the number of patients at risk. (**D**) The comparison methods involved in the studied cohorts, explains as follows: Gender: male vs. female; Race: white vs. non-white; Ethnicity: Hispanic or Latino vs. non-Hispanic or Latino; Prior malignancy: yes vs. no; Tumor origin: upper lobe lung vs. non-upper lobe lung; Smoking history: ever vs. never; KRAS mutation: yes vs. no; TP53 mutation: yes vs. no; EGFR mutation: yes vs. no; STK11 mutation: yes vs. no; T classification: T2-T4 vs. T1; Only univariate variables with *p*-values less than 0.1 were included in the multivariable analysis; The bold *p*-value indicates that <0.05, which considers significant. (**E–G**) The ROC curves valued the accuracy for LUAD outcome prediction of our signature at 1-, 3-, and 5-year, respectively. The IAUC and IBS analyses compared our signature’s prognostic ability with other available clinical characteristics. The larger the AUC, or the smaller the BS, the stronger the model’s predictive ability. HR, hazard ratio; CI, confidence interval; ROC, receiver operating characteristic; AUC, area under the ROC curve; IAUC, integrated AUC; BS, Brier score; IBS, integrated Brier score; LUAD, lung adenocarcinoma.

Kaplan-Meier survival curves **(**Figures 3A–C**)** showed that the survival rate of the patients in the low-risk group was significantly higher than that in the high-risk group in the training cohort (*p*-value = 5.808e-08), validation cohort one (*p*-value = 6.41e-08), and validation cohort two (*p*-value = 1.204e-08). To determine whether the prognostic significance of the risk score depends on clinicopathological parameters, univariate and multivariable Cox analyses were performed to analyze the following variables: risk score, age, tumor location, grade, tumor stage, etc. (Figure 3D). The Cox models that in the training cohort demonstrated that risk score and tumor stage interpedently impacted the LUAD patients' prognosis, whereas the *p*-value of the risk score was more significant (*p*-value <= 1.72e−11 vs. *p*-value <= 1.41e−04). Consistently, the univariate or multivariable Cox models of the validation cohort one found the risk score was the best factor that predicting the LUAD prognosis (*p*-value <= 1.55e−07). The analyses performed in validation cohort two demonstrated that age, T classification, and risk score could affect the prognosis. Still, the impact of the risk score (*p*-value <= 6.50e−10) was obviously the strongest among them.

We used ROC analysis, IAUC, and IBS to evaluate our model's discrimination performance and to assess heterogeneities in its predicting ability (Figures 3E–G). The AUC of the signature in the training cohort was 0.734 at 1-year, 0.715 at 3-year, and 0.653 at 5-year. Even though the tumor stage caught up with the risk score's IAUC around the 5-year time point, the risk score stayed at the best performance level in general. The value of the Brier score is always between 0.0 and 1.0, with a perfect model score of 0.0 and the worst score of 1.0 (21). Similarly, the risk score IBS stayed at the best level at the majority of period showed. The results generated from the validation cohort one and validation cohort two consistent with those from the training cohort, showing that the thirteen-gene model was better than other clinical factors in predicting the LUAD outcomes. In summary, the signature we discovered are better than other clinical factors at predicting the outcomes of LUADs.

### The thirteen-gene signature is better than our predecessors

**Figure 4:**
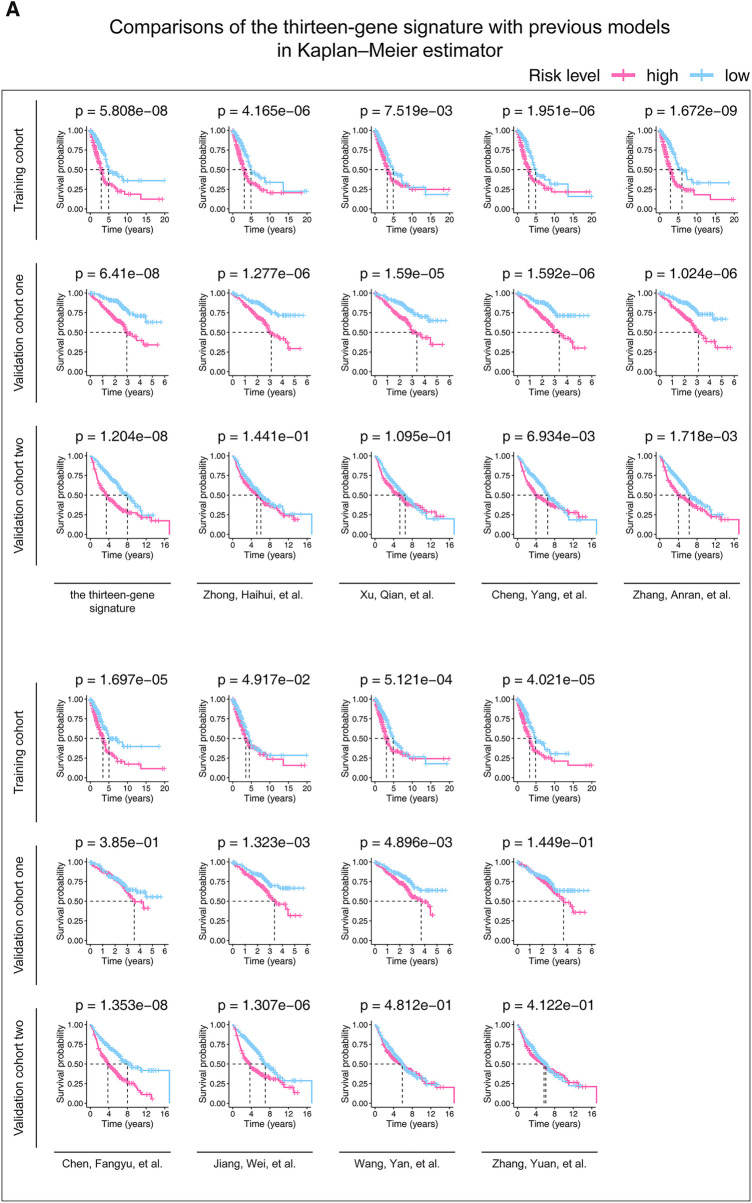

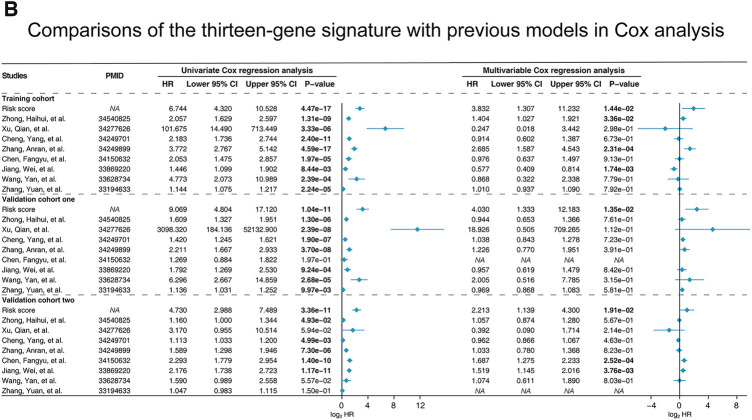
Kaplan-Meier analysis (**A**) and Cox models (**B**) were performed in the studied cohorts to compare our and previous studies’ predictive abilities. (**A**) Patients in each cohort were divided into low-risk groups and high-risk groups based on their median risk score. The log-rank test with a *p*-value < 0.05 suggests the survival difference is significant. (**B**) Only univariate variables with *p*-values less than 0.1 were included in the multivariable analysis. The bold words mean the *p*-value < 0.05. *P*-value is considered significantly. HR, hazard ratio; CI, confidence interval; PMID, PubMed ID, https://pubmed.ncbi.nlm.nih.gov/.

According to the screening criteria we set, eight previous studies were found and presented in Supplementary Material Table S3. The Kaplan–Meier estimators (Figure 4A) confirmed that our finding (*p*-value <= 6.41e-08) were more predictive than those of Cheng, Yang, et al.'s (*p*-value <= 6.934e-03), Zhang, Anran, et al.'s (*p*-value <= 1.718e-03), and Jiang, Wei, et al.'s (*p*-value <= 4.917e-02) in terms of their *p*-values. Furthermore, Cox univariate and multivariable regressions (Figure 4B) further indicated the stability and superiority of our gene model. The results showed that previous studies only displayed significance in some cohorts or only in part analyses. However, our model showed significance in univariate and multivariable analyses in all cohorts.

### GSEA determined the mechanisms of the prognosis signature

**Figure 5:**
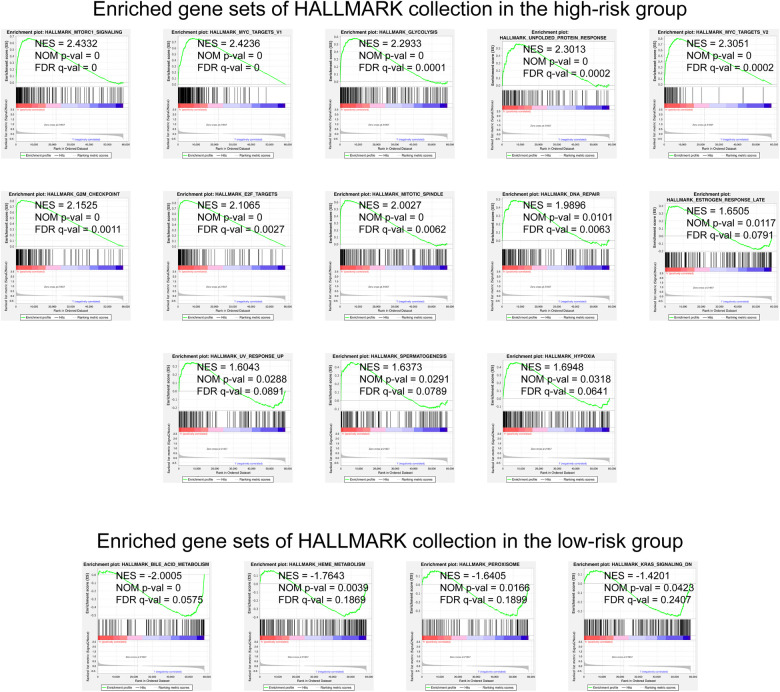
GSEA analysis with the HALLMARK gene set as the background identified relevant pathways of our signature. The significance threshold of this analysis was set as: | NES | >1, NOM *p*-value < 0.05, and FDR *q*-value < 0.25. GSEA, Gene Set Enrichment Analysis; NES, Normalized Enrichment Score; NOM, Nominal; FDR, False Discovery Rate.

We selected the Hallmark gene set collections to obtain a GSEA Hallmark enrichment analysis. The enrichment graph is presented as Figure 5. The results of the GSEA Hallmark analysis revealed significant pathways as follows: mTORC1 signaling, MYC, glycolysis / gluconeogenesis, unfolded protein response, G2/M checkpoint, E2F transcription factors, mitotic spindle assembly, DNA repair, estrogen response, ultraviolet radiation, spermatogenesis, hypoxia, bile acids metabolism, heme metabolism, peroxisome, and KRAS activation.

### Immune relevant signatures correlations

**Figure 6:**
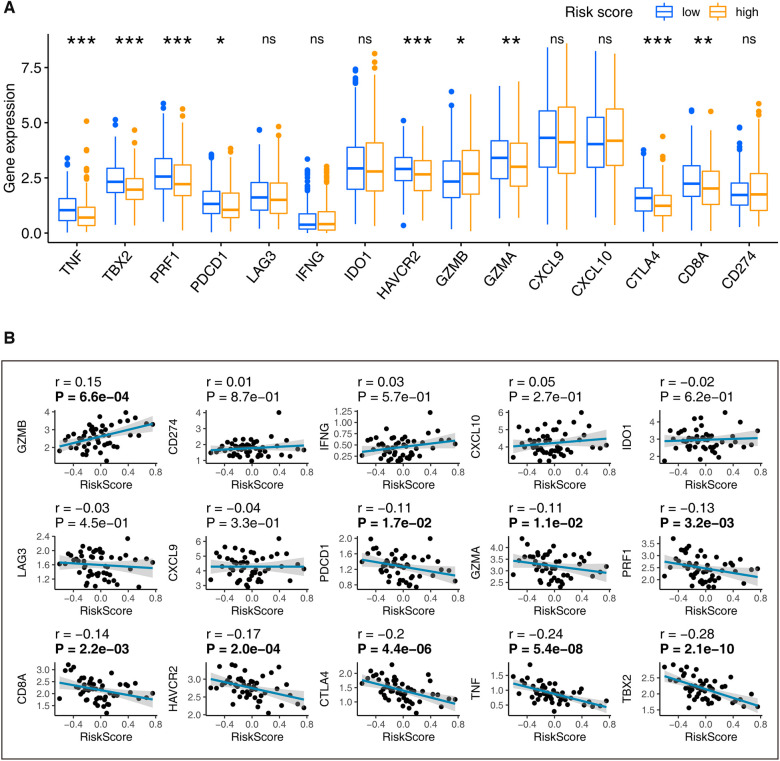
Determination of the immune relevant signatures that are significantly related to our signature. (**A**) LUADs were grouped into low- and high-risk based on their median risk score. The Wilcoxon rank-sum found immune signatures that differentially distributed in the high-risk and low-risk patients. (**B**) The Spearman’s coefficient evaluated the correlations between our signature and immune relevant signatures. ns: *p*-value > 0.05; *: *p*-value < 0.05; **: *p*-value < 0.01; A *p*-value < 0.05 means statistical significance; The bold *p*-value in plot B indicates that <0.05. LUAD, lung adenocarcinoma.

The immune relevant signatures, including TBX2, TNF, CTLA4, HAVCR2, GZMB, CD8A, PRF1, GZMA, and PDCD1, were found expressed differently between high- and low-risk LUADs, as demonstrated by the Wilcoxon test (Figure 6A). Interestingly, the Spearman coefficient examination found TBX2, TNF, CTLA4, HAVCR2, GZMB, CD8A, PRF1, GZMA, and PDCD1 correlated with the thirteen-gene signature (Figure 6B). Incorporating the above findings, nine signatures, including TBX2, TNF, CTLA4, HAVCR2, GZMB, CD8A, PRF1, GZMA, and PDCD1 were recognized associating with the thirteen-gene signature.

### Mast cells play vital roles in our signature's ability in LUADs

**Figure 7:**
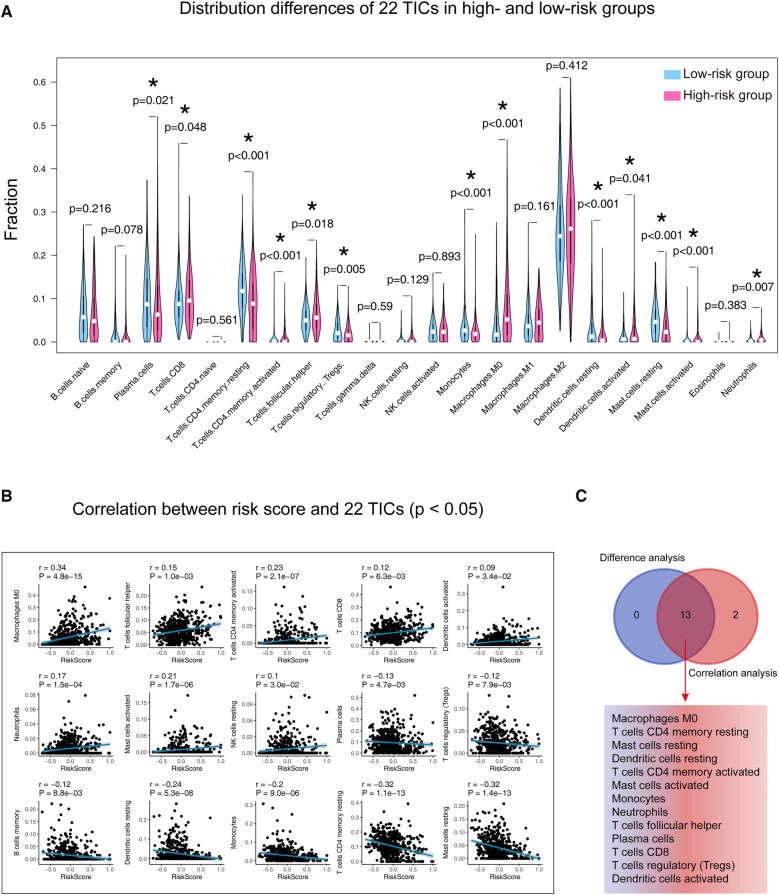
Determination of the relationship between the 22 TICs and our signature. (**A**) Patients were divided into high and low-risk groups according to their median risk score, and the Wilcoxon rank-sum was applied to detect differences in TIC distribution between different groups. (**B**) Spearman’s coefficient assessed the correlations between the 22 TICs and our model. Here, we plotted the TIC correlation with a *p*-value < 0.05. (**C**) The results of Wilcoxon’s rank-sum test and Spearman’s coefficient were intersected to determine stable and critical TICs. TIC, tumor-infiltrating immune cell; *: *p*-value < 0.05; *p*-value < 0.05 is considered significant.

Supplementary Material Figure S4 detailed displays the 22 TICs distribution and their inner relationships. As shown in Figure 7 and Supplementary Material Table S4, the Wilcoxon rank sum test identified 13 TICs related to the risk score, and Spearman's coefficient also found 15 TICs closely linked to our signature. In summary, a total of 13 TICs are significantly related to the gene signature, which included Macrophages M0, T cells CD4 memory resting, Mast cells resting, Dendritic cells resting, T cells CD4 memory activated, Mast cells activated, Monocytes, Neutrophils, T cells follicular helper, Plasma cells, T cells CD8, T cells regulatory (Tregs), and Dendritic cells activated. Specifically, our signature was positively correlated with Dendritic cells activated, T cells CD8, T cells follicular helper, Neutrophils, Mast cells activated, T cells CD4 memory activated, and Macrophages M0, while it was negatively correlated with the rest.

**Figure 8:**
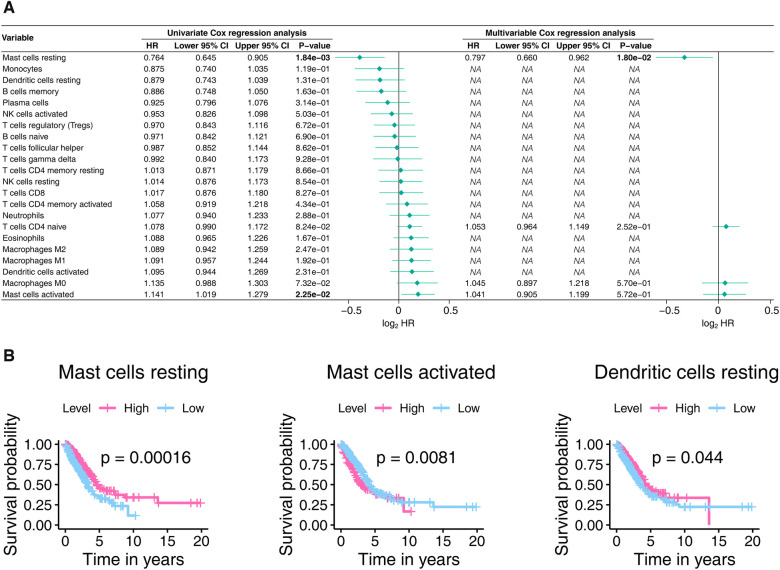
The prognostic power of the 22 TICs was determined using the Cox proportional hazards model (**A**) and the Kaplan-Meier estimator (**B**). In Panel A, only univariate variables with *p*-values less than 0.1 were included in the multivariable analysis, with bold text indicating *p*-values < 0.05. In Panel B, we only show Kaplan-Meier plots with *p*-values less than 0.05. TIC, tumor-infiltrating immune cell; *p*-value < 0.05 is considered significant.

The 22 TICs' prognostic importance were measured by consulting Cox's univariate and multivariable proportional hazard models and Kaplan-Meier estimator. As shown in Figure 8A, Mast cells resting and Mast cells activated significantly affect the prognosis of LUAD. The Kaplan-Meier analysis (Figure 8B, Supplementary Table S5) hinted that Mast cells resting, Mast cells activated, and Dendritic cells resting were able to predict LUAD prognosis. In aggregate, the mast cells significantly impacted the outcomes of LUAD.

The results in this section confirm that mast cells were significantly associated with our signature and have the ability to predict LUAD outcome, further implying a potential association of mast cells infiltration with our signature‘s prognostic power.

## Discussion

In this study, we innovatively established the ICI profile of LUAD by mining TCGA and GEO databases and discovered a robust thirteen-gene prognostic signature from the ICI differences. In the present research, we included three datasets, including TCGA-LUAD, GSE72094, and GSE68465, containing 1,340 samples. Not only that, the novelty of our study lies in the use of multiple statistical methods, including Kaplan-Meier analysis, Cox regression, ROC curve, IAUC, and IBS, to validate our trained model. Given the current plethora of studies of the same type, we also compared with them to highlight the strengths of our model. The most important thing is that we conducted a correlation analysis between our model and some current mainstream immune signatures to find potential immune targets with our model. Finally, GSEA and immune infiltration analysis revealed important mechanisms associated with gene signatures and speculated that mast cells might contribute to the predictive power of our signature.

Our signature contains thirteen genes (Table 2), which were ABCC2, CCR2, CERS4, CMAHP, DENND1C, ECT2, FKBP4, GJB3, GNG7, KRT6A, PCDH7, PLK1, and VEGFC. In our research, PLK1, GJB3, VEGFC, FKBP4, ABCC2, KRT6A, PCDH7, and ECT2 adversely affected LUAD outcomes, while the remaining genes displayed favorable impacts (Supplementary Material Figure S3). PLK1 is related to tumor aggressiveness and patient prognosis of non-small cell lung cancer (NSCLC). Studies have shown that PLK1 expression is up-regulated in various cancer types, which in turn induces cell proliferation and malignant transformation (38). Aasen and colleagues identified GJB3 to be significantly associated with poor prognosis in LUAD patients (39). In comparison, Want et al. demonstrated that GJB3 was overexpressed in squamous cell lung carcinoma (40). VEGF is a key mediator of cancer angiogenesis, and it is up-regulated by oncogene expression, a variety of growth factors, and hypoxia (41, 42). VEGF overexpression has been reported in lung cancer (41, 42). Recent studies have shown that VEGF-B may trigger tumor angiogenesis through a pathway independent of VEGF-A, and it may even be a prognostic marker of cancer metastasis (41, 42). Both VEGF-C and VEGF-D are closely related to lymphatic angiogenesis (41, 42). FKBP4 plays a role in immune regulation, protein folding, and transport. Experiments confirmed that FKBP4 activates the Akt/mTOR signaling pathway and acts as an oncogene to promote the malignant process of NSCLC (43). ABCC2 plays a vital role in transporting endogenous and exogenous substances and affecting drug absorption, distribution, and excretion (44). Chen's study proved that the high ABCC2 expression is linked to cisplatin resistance, and reducing the ABCC2 expression can gain the sensitivity of NSCLC patients to cisplatin (44). Previous studies have shown that KRT6A plays a crucial role in cell migration, especially keratinocyte migration (45). Yang and colleagues demonstrated that KRT6A is highly expressed in LUAD, especially in samples from lymph node-positive patients (45, 46). Another study found that LUAD with low KRT6A expression had a better prognosis than LUAD with high KRT6A expression (46). Moreover, Che's research revealed that KRT6A might serve as a potential prognostic indicator and therapeutic target for NSCLC (47). They showed an under-recognized mechanism that KRT6A acts downstream of LSD1 and upregulates G6PD through the MYC signaling pathway, demonstrating its vital role for NSCLC progression (47). PCDH7, a member of the protocadherins family, acts as a tumor suppressor in various human cancers (48). Although PCDH7 has recently been shown to have the effect of accelerating the distant metastasis of lung cancer and breast cancer, some studies have also shown that PCDH7 has low expression in colorectal cancer, gastric cancer, and invasive bladder cancer (49). Studies have shown that high ECT2 expression predicts the progression of NSCLC and the poor prognosis, and is related to the proliferation, survival, and invasion of tumor cells (50).

Bioinformatics reveals the biological mysteries endowed by large and complex biological data through the integrated use of biology, computer science and information technology. In recent years, using public databases and bioinformatics methods, researchers have explored and established many prognostic models related to LUAD. Previous studies have made great contributions to the further development of LUAD. Whether the LUAD prognostic model we established in this study has an advantage, we also answer in this paper. By comparison, the prognostic power of our model was better than the eight studies (51–58) from the most recent year.

GSEA found that the pathways of mTORC1 signaling, MYC, and glycolysis / gluconeogenesis ranked top potential that play vital roles in gene signature functioning. mTOR complex is recently depicted as a nutrient sensor in the metabolism of cancer (59). mTORC1 integrates signals from a variety of growth factors, nutrients and energy supply (60). mTORC1 promotes cell growth when energy is sufficient, and promotes catabolism when the body is hungry (60). Abnormal activation of the Akt/mTOR pathway is usually observed in lung cancer. The dysregulated PI3-K/Akt/mTOR activity is known to contribute to the development and maintenance of lung cancer (61). Targeting of mTOR is an attractive and promising approach in the development of therapeutic agents against lung cancer (61). MYC plays a key role in growth regulation, differentiation and apoptosis (62). The abnormal expression of MYC is related to a variety of tumors (62). Overexpression of MYC makes cells sensitive to apoptosis caused by various stimuli (62). MYC is an oncogene that is out of control in human cancers, including lung cancer, where it supports oncogenic processes and progression (63). MYC has a key role in regulating lung cancer cell behavior, regulating lung cancer cell growth, resistance, death, and dissemination by regulating kinesins, anti-apoptotic proteins, and metabolism (63). Glycolysis is an inefficient way of energy metabolism (64). Cancer cells are more likely to use glycolysis even when there is enough oxygen (64). This dependence on aerobic glycolysis is called the Warburg effect and promotes tumorigenesis and malignant tumor progression (64). Inhibition of glycolysis is considered to be a treatment for aggressive cancers including lung cancer. It has recently been discovered that the shortened gluconeogenesis mediated by phosphoenolpyruvate carboxykinase can partially avoid the need for glycolysis in lung cancer cells (65).

Immunotherapy is a treatment that uses parts of the body's immune system to fight diseases such as cancer. This can be done in several ways (66): (1) Stimulate or enhance the immune system's natural defenses, making it harder or smarter to find and attack cancer cells. (2) Making substances similar to components of the immune system in the lab and using them to help restore or improve the way the immune system finds and attacks cancer cells. In recent decades, with the development of early diagnosis techniques, immunotherapy has made a significant contribution to improving the survival rate of cancer patients (67). Immune checkpoint inhibitors, particularly inhibitors of the PD-1 axis, have altered the management of NSCLC over the last ten years (68). First demonstrated to improve outcomes in second-line or later therapy of advanced disease, immune checkpoint inhibitors were shown to improve overall survival compared to chemotherapy in first-line treatment for patients whose tumors express PD-L1 on at least 50% of cells (68). More recently, combining immune checkpoint inhibitors with chemotherapy has improved survival in patients with squamous and nonsquamous NSCLC, regardless of PD-L1 expression (68). Immune checkpoint blockade has been shown to be effective in a substantial proportion of patients' refractory to standard therapy. However, the effectiveness of this approach in LUAD is rarely reported (69). Several biological features of LUAD suggest that modulation of the immune response may confer benefits (69). One of the main challenges of LUAD immunotherapy is identifying predictive sensitive biomarkers so that treatment can be tailored to maximize benefit (67, 69). In the present study, nine immune relevant signatures, including TBX2, TNF, CTLA4, HAVCR2, GZMB, CD8A, PRF1, GZMA, and PDCD1 were determined as closely linked to our thirteen-gene signature and might contribute to the ongoing LUAD immunotherapy research.

Our study speculated that extensive infiltration of mast cells in LUAD tumors contributes to maintaining the stable predictive power of our signature. Mast cell is known for its involvement in allergic disorders, but in recent years, accumulating evidence has shed light on its involvement in cancer, including LUAD (70–72). Mast cells are involved in disease processes characterized by inflammation and tissue remodeling and correlated with innate and adaptive immune responses to respiratory pathogens to promote lung health (73). Mast cells contribute to the secretion of VEGF, which in turn is associated with poor prognosis in NSCLC (73). Since the immune microenvironment plays a key role in the tumor progression, mast cells, as a critical stromal component of the immune system, are undoubtedly a key regulator for maintaining tissue homeostasis. Therefore, we believe that it is crucial to conduct further studies to understand the role of mast cells in tumor microenvironment remodeling.

This study has certain limitations. We generated a thirteen-gene signature from retrospective data. Although our model has absolute advantages over its predecessors, its proof is still by adopting public databases. Its applicability in real life still needs more clinical data to support. In addition, when comparing with the predecessors, because the datasets used by them were not uploaded to their respective supplementary files, and the these authors did not clearly explain the preprocessing methods and specific data sources, we used the unified official TCGA dataset for comparison. These situations may contribute to slight deviations in the results. Moreover, the prognostic ability and mechanism of action of the thirteen-gene signature and mast cells still need to be verified by wet experimental data. Overall, our study brings more implications for future LUAD treatment and diagnosis, but also more research is needed to reveal a bright future for the thirteen-gene signature.

## Conclusion

This study established the ICI characteristics of LUAD and constructed a powerful thirteen-gene prognostic signature based on the ICI. By applying the signature to independent datasets, its stability and broad applicability are validated. More importantly, we compared ours with the previous studies and confirmed our signature's superiority. The mast cells potentially help the signature to maintain its predictive power. Our work may promote the evolution of diagnosis and treatment of LUAD.

## Data Availability

Publicly available datasets were analyzed in this study. This data can be found here: The following publicly available datasets were used in this study: TCGA-LUAD: https://gdc.xenahubs.net; GSE72094: https://www.ncbi.nlm.nih.gov/geo/query/acc.cgi?acc=GSE72094; GSE68465: https://www.ncbi.nlm.nih.gov/geo/query/acc.cgi?acc=GSE68465.
