# Supplementary material for: A more novel and powerful prognostic gene signature of lung adenocarcinoma determined from the immune cell infiltration landscape
Source: Front Surg. 2022 Oct 13;9:1015263. doi: 10.3389/fsurg.2022.1015263 (PMC9606711; doi:10.3389/fsurg.2022.1015263)
Supplement: Supplementary file 1 [file DataSheet1.pdf]

Figure S1

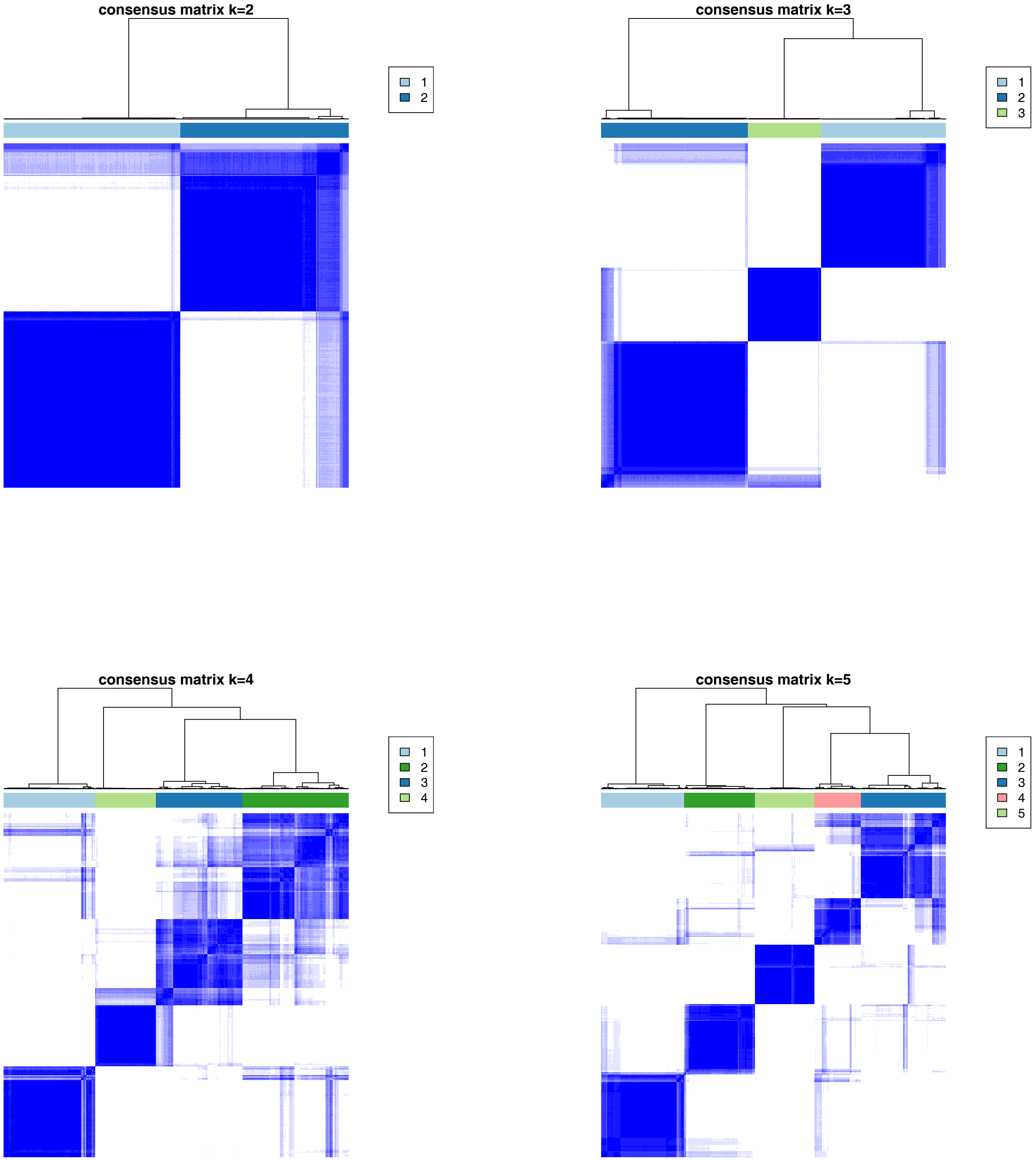
consensus matrix k=2
1
2
consensus matrix k=3
1
2
3
consensus matrix k=4
1
2
3
4
consensus matrix k=5
1
2
3
4
5

Figure S2

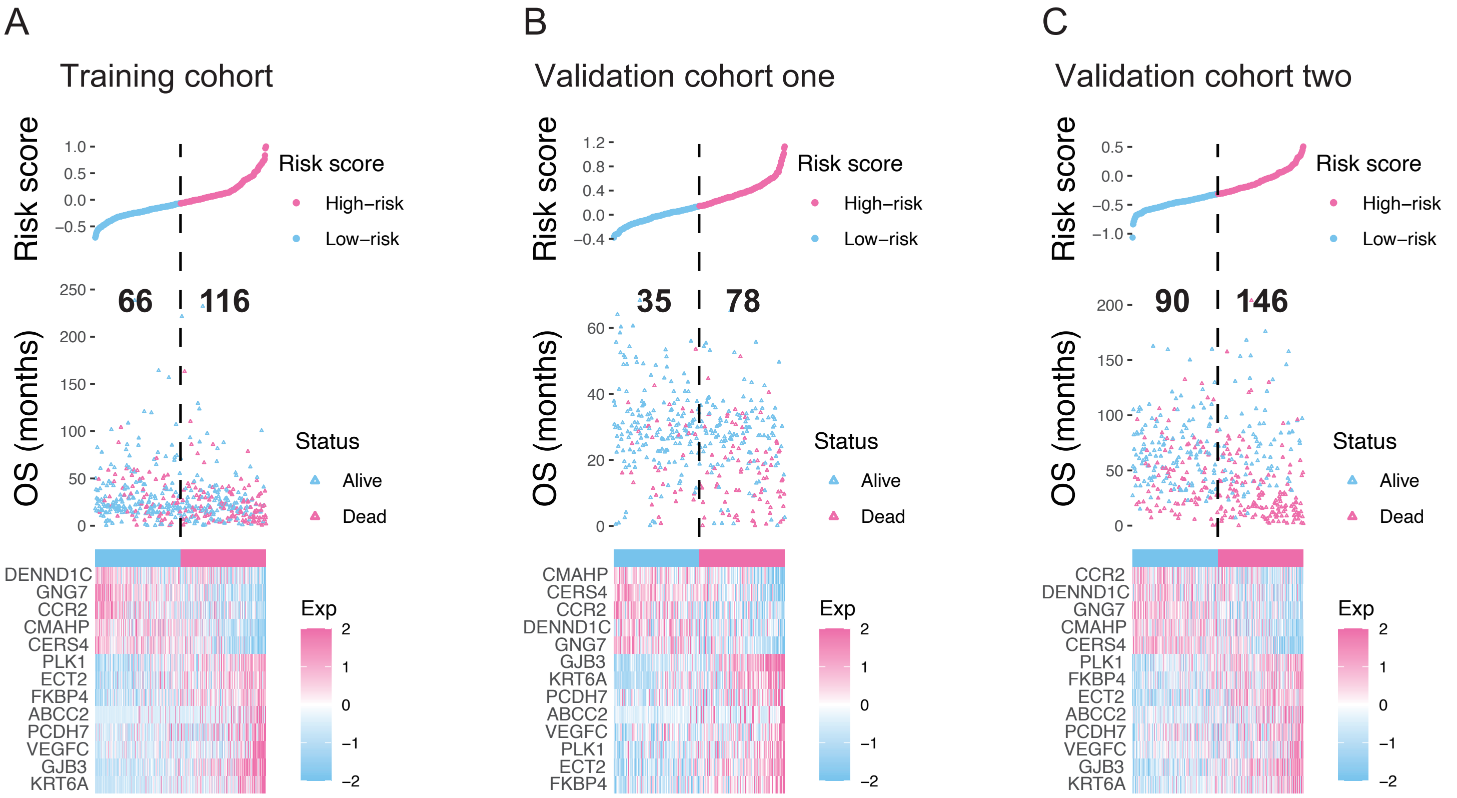
A
Training cohort
Risk score
1.0
0.5
0.0
−0.5
Risk score
High−risk
Low−risk
66
116
OS (months)
250
200
150
100
50
0
Status
Alive
Dead
DENND1C
GNG7
CCR2
CMAHP
CERS4
PLK1
ECT2
FKBP4
ABCC2
PCDH7
VEGFC
GJB3
KRT6A
Exp
2
1
0
−1
−2
B
Validation cohort one
Risk score
1.2
0.8
0.4
0.0
−0.4
Risk score
High−risk
Low−risk
35
78
OS (months)
60
40
20
0
Status
Alive
Dead
CMAHP
CERS4
CCR2
DENND1C
GNG7
GJB3
KRT6A
PCDH7
ABCC2
VEGFC
PLK1
ECT2
FKBP4
Exp
2
1
0
−1
−2
C
Validation cohort two
Risk score
0.5
0.0
−0.5
−1.0
Risk score
High−risk
Low−risk
90
146
OS (months)
200
150
100
50
0
Status
Alive
Dead
CCR2
DENND1C
GNG7
CMAHP
CERS4
PLK1
FKBP4
ECT2
ABCC2
PCDH7
VEGFC
GJB3
KRT6A
Exp
2
1
0
−1
−2

Figure S3

A

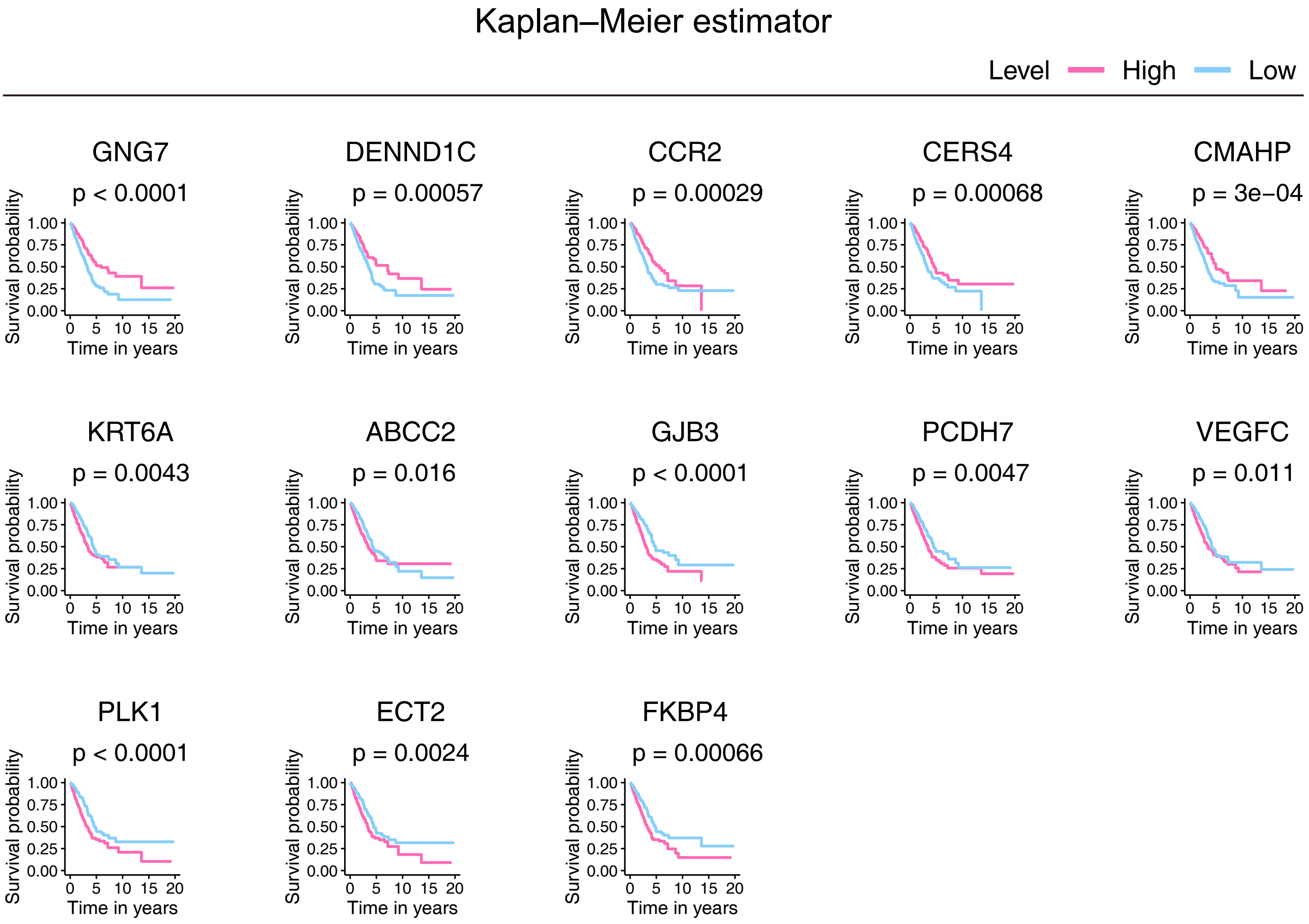

B

Univariate Cox regression

ID	HR	HR_95L	HR_95H	P-value
GNG7	0.575	0.456	0.725	3.01E-06
DENND1C	0.615	0.475	0.796	2.29E-04
CCR2	0.657	0.537	0.805	5.01E-05
CERS4	0.708	0.612	0.819	3.33E-06
CMAHP	0.709	0.608	0.828	1.33E-05
KRT6A	1.156	1.094	1.221	2.05E-07
ABCC2	1.191	1.101	1.288	1.35E-05
GJB3	1.287	1.172	1.412	1.14E-07
PCDH7	1.295	1.135	1.478	1.26E-04
VEGFC	1.373	1.201	1.569	3.27E-06
PLK1	1.405	1.215	1.625	4.46E-06
ECT2	1.416	1.214	1.651	8.94E-06
FKBP4	1.512	1.264	1.810	6.41E-06

Figure S4

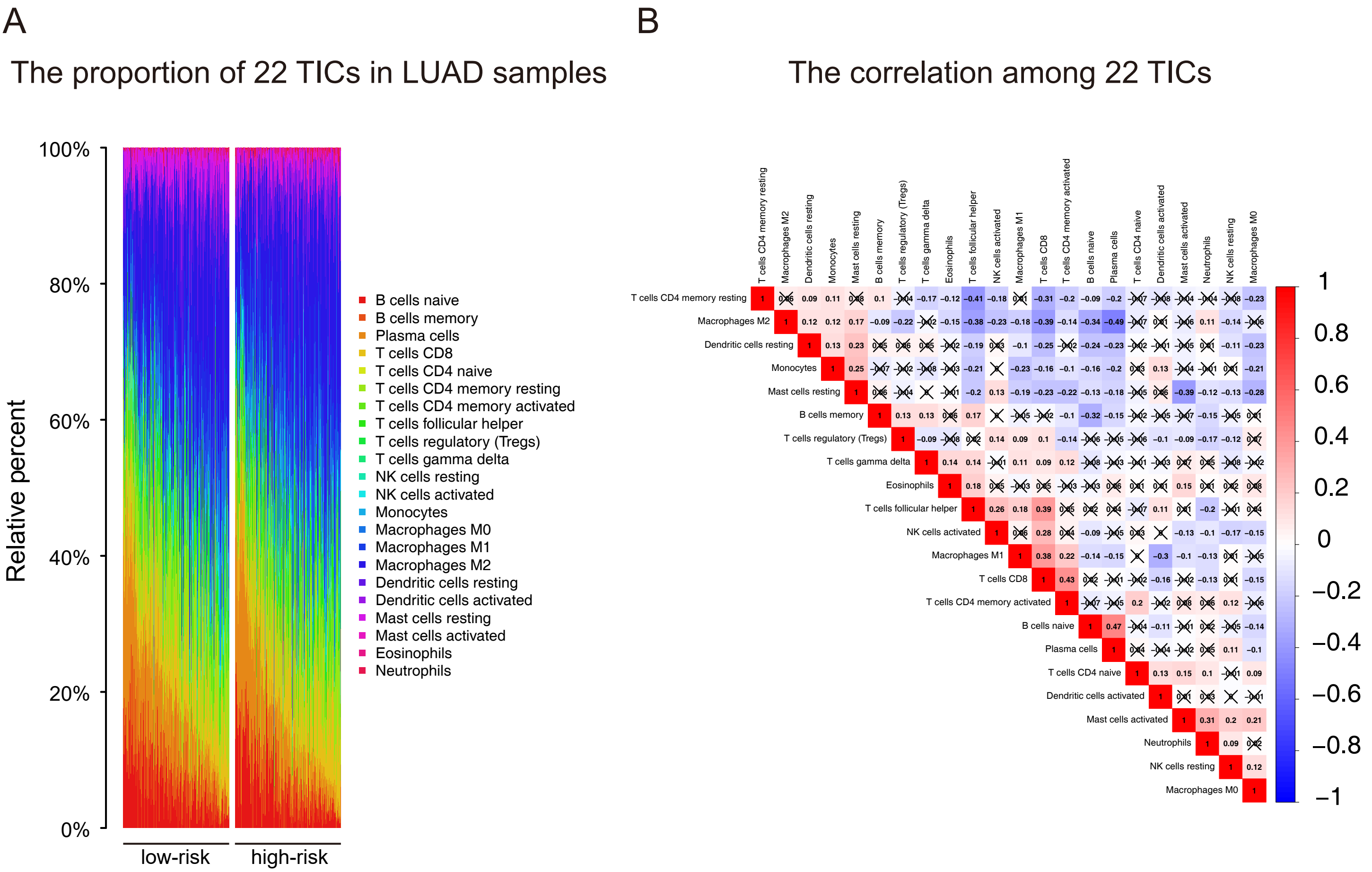
A
The proportion of 22 TICs in LUAD samples
100%
80%
60%
40%
20%
0%
Relative percent
low-risk
high-risk
B cells naive
B cells memory
Plasma cells
T cells CD8
T cells CD4 naive
T cells CD4 memory resting
T cells CD4 memory activated
T cells follicular helper
T cells regulatory (Tregs)
T cells gamma delta
NK cells resting
NK cells activated
Monocytes
Macrophages M0
Macrophages M1
Macrophages M2
Dendritic cells resting
Dendritic cells activated
Mast cells resting
Mast cells activated
Eosinophils
Neutrophils
B
The correlation among 22 TICs
T cells CD4 memory resting
Macrophages M2
Dendritic cells resting
Monocytes
Mast cells resting
B cells memory
T cells regulatory (Tregs)
T cells gamma delta
Eosinophils
T cells follicular helper
NK cells activated
Macrophages M1
T cells CD8
T cells CD4 memory activated
B cells naive
Plasma cells
T cells CD4 naive
Dendritic cells activated
Mast cells activated
Neutrophils
NK cells resting
Macrophages M0
1
0.8
0.6
0.4
0.2
0
−0.2
−0.4
−0.6
−0.8
−1

Table S1. Determination of differentially expressed genes between two types of ICI with the threshold parameters of p-value < 0.05 and | log2(fold-change)| > 0.2.

ID	log2(fold-change)	average expression	t	p-value	B
LCP2	-1.126705738	2.586045829	-23.97017259	6.13E-85	182.9916104
NCKAP1L	-1.218276852	2.330209997	-23.94881175	7.78E-85	182.7547517
EVI2B	-1.373416382	3.532214884	-23.62683625	2.81E-83	179.1826832
CD53	-1.468067369	4.855088869	-23.15794133	5.24E-81	173.9753614
CD4	-1.374899572	4.459245541	-23.02967288	2.19E-80	172.5500044
BTK	-1.063722036	1.88809879	-23.00462773	2.90E-80	172.2716596
SASH3	-1.327954213	3.295487363	-22.68496273	1.03E-78	168.7181463
WIPF1	-1.045202306	3.037797776	-22.63581773	1.78E-78	168.1717182
SNX20	-0.926084147	1.55357536	-22.58013401	3.31E-78	167.55256
SELPLG	-1.30379632	3.835612508	-22.53457487	5.51E-78	167.0459592
GIMAP4	-1.21075111	3.717538989	-22.41703935	2.05E-77	165.7389411
LPXN	-1.049406091	3.322878208	-22.34653765	4.49E-77	164.9549135
PTPRC	-1.486272514	3.156232642	-22.34438147	4.60E-77	164.930935
HAVCR2	-1.2504727	2.74395073	-22.25738572	1.22E-76	163.9634597
DOCK2	-1.032588524	1.786372692	-22.25381344	1.27E-76	163.9237322
LAPTM5	-1.41835288	6.328558744	-22.03155905	1.51E-75	161.4520427
PLEKHO2	-0.959055835	3.713883738	-22.02808563	1.57E-75	161.4134157
CD86	-1.151778532	2.58125386	-22.02057713	1.71E-75	161.3299157
PLEK	-1.456956769	3.445970643	-21.96725678	3.10E-75	160.7369618
CYTH4	-1.010757056	2.16746093	-21.72452858	4.66E-74	158.0379254
GPR65	-0.788604745	1.20160737	-21.62721918	1.38E-73	156.9560502
LAIR1	-1.159448825	2.39425368	-21.53587108	3.83E-73	155.940568
IKZF1	-0.996659571	1.679444566	-21.48469493	6.78E-73	155.3717183
CYBB	-1.708824733	4.307798978	-21.4409497	1.10E-72	154.8855025
IL10RA	-1.176953279	2.715780869	-21.38330288	2.10E-72	154.2448276
EVI2A	-1.231384513	2.699327621	-21.26946136	7.49E-72	152.9798123
RCSD1	-0.979397461	2.010990109	-21.2650271	7.87E-72	152.930544
SCIMP	-0.948743735	1.435983046	-21.17077728	2.25E-71	151.8834619
GNG2	-0.788148077	1.779391738	-21.05292134	8.38E-71	150.5744395
CD84	-1.10237479	1.833541139	-21.03642433	1.01E-70	150.3912379

TAGAP	-1.04998519	1.994775973	-21.00460003	1.44E-70	150.0378465
AIF1	-1.368098604	4.43498511	-20.94059111	2.93E-70	149.3271539
CSF2RB	-1.176414152	2.433104492	-20.89634268	4.80E-70	148.8359365
MNDA	-1.408005491	3.042466637	-20.88779067	5.28E-70	148.7410049
ARHGEF6	-0.961082164	2.177042304	-20.87525376	6.07E-70	148.6018431
IL16	-0.878693667	1.461952868	-20.84273714	8.72E-70	148.2409275
MS4A6A	-1.33353716	3.122875229	-20.76439413	2.09E-69	147.371514
ZEB2	-0.771382549	1.514770545	-20.75554992	2.30E-69	147.2733788
WAS	-1.107531148	2.896380135	-20.71322956	3.69E-69	146.8038319
CCR5	-1.147383354	2.061322481	-20.66086228	6.62E-69	146.2229067
GMFG	-1.144556681	3.813517597	-20.45896732	6.26E-68	143.9842581
C3AR1	-1.350773186	3.299990717	-20.39031849	1.34E-67	143.223468
SLAMF8	-1.341633959	3.196974957	-20.37691565	1.56E-67	143.0749583
CD37	-1.273847863	3.052350218	-20.25237134	6.24E-67	141.6953603
TNFAIP8L2	-1.131458545	2.561533239	-20.25138538	6.30E-67	141.6844416
SLA	-1.028289852	2.254789944	-20.22922838	8.07E-67	141.4390863
IGSF6	-1.274762565	2.794391694	-20.14218095	2.12E-66	140.4754125
C10orf128	-0.89667378	1.789604292	-20.06873725	4.80E-66	139.6626566
BIN2	-1.002745174	2.092469272	-19.97494213	1.36E-65	138.625123
SLCO2B1	-1.332045153	3.056723095	-19.92981966	2.25E-65	138.126174
PCED1B-AS1	-0.950838405	1.91185372	-19.87372964	4.19E-65	137.5061199
FERMT3	-1.199760663	3.61296167	-19.87279433	4.23E-65	137.4957821
IRF8	-1.185123359	2.429668634	-19.86894163	4.41E-65	137.4531993
TRPV2	-1.030199372	2.927887794	-19.86037643	4.85E-65	137.3585337
CSF1R	-1.365327006	3.844986461	-19.82658825	7.06E-65	136.9851396
CD28	-0.717200933	1.021810173	-19.79816513	9.68E-65	136.6710906
DOK2	-1.229499331	2.863563226	-19.77087284	1.31E-64	136.369585
PLXNC1	-0.938891712	2.131146446	-19.73542238	1.94E-64	135.9780259
SPN	-1.092018542	1.920337888	-19.71460786	2.45E-64	135.7481629
IL7R	-1.45424828	2.988883347	-19.65544521	4.71E-64	135.0949648
SAMSN1	-1.124675269	2.395103344	-19.63655157	5.81E-64	134.8864157
MYO1F	-0.952032722	2.242394152	-19.55737826	1.40E-63	134.0127662
ARHGAP25	-0.84581035	2.127891503	-19.55446623	1.44E-63	133.9806414

RP11-750H9.5	-0.815340532	1.250658997	-19.52705673	1.95E-63	133.6782967
CD33	-0.633412013	0.929493241	-19.52207958	2.07E-63	133.6234013
TNFSF13B	-1.1913078	2.665332343	-19.51073426	2.34E-63	133.498275
1-Mar	-0.695361952	1.357140535	-19.50498889	2.50E-63	133.4349135
ITGAL	-1.234426297	2.622265799	-19.47827879	3.35E-63	133.1403792
CLEC4A	-1.121504312	2.234844856	-19.47597062	3.44E-63	133.1149293
GIMAP6	-1.038157872	2.586772602	-19.4678392	3.77E-63	133.0252753
PDCD1LG2	-1.048615502	1.620087554	-19.44375659	4.92E-63	132.7597787
CCR2	-0.978869686	1.480615153	-19.41283648	6.92E-63	132.4189664
FPR3	-1.360017838	3.479508128	-19.35301011	1.34E-62	131.759747
MPEG1	-1.271279878	3.300222503	-19.3432518	1.49E-62	131.6522476
FAM78A	-0.793388906	1.580974778	-19.34211603	1.51E-62	131.6397363
AOAH	-1.096445435	1.976947439	-19.30378991	2.31E-62	131.2176048
LCP1	-1.395961484	5.142656456	-19.29109024	2.66E-62	131.0777538
PILRA	-1.083909534	2.551490602	-19.2598594	3.76E-62	130.7338896
TLR4	-1.041551224	2.019543385	-19.23370982	5.02E-62	130.4460329
P2RY13	-1.041033256	1.641400905	-19.22165646	5.73E-62	130.3133674
PIK3AP1	-1.144759899	2.574355727	-19.21964416	5.86E-62	130.2912201
HLA-DQA1	-1.762866153	4.890745085	-19.21156702	6.41E-62	130.202327
IL21R	-0.82790931	1.190806377	-19.19603454	7.61E-62	130.0313991
TFEC	-0.779047747	1.072100968	-19.16044936	1.13E-61	129.6398763
PTPN7	-0.962570132	1.846919754	-19.12240494	1.72E-61	129.2214142
ARHGAP30	-1.074770252	2.99786864	-19.06798878	3.13E-61	128.6230903
SIRPA	-1.134006399	3.459865089	-19.05696526	3.54E-61	128.5019144
TLR7	-0.790352608	1.113234	-19.02380333	5.10E-61	128.137447
SPI1	-1.309312082	4.138378829	-18.95968093	1.03E-60	127.4329859
GGTA1P	-0.784669717	1.447282063	-18.89380882	2.14E-60	126.7096925
TRAC	-1.419266369	3.851740344	-18.82049444	4.80E-60	125.9051582
RNASE6	-1.176375955	3.852119914	-18.80749602	5.54E-60	125.7625701
LY86	-1.262624518	2.882503159	-18.76760885	8.59E-60	125.3251239
ABI3	-0.927102372	2.723727993	-18.75751129	9.60E-60	125.2144076
FCER1G	-1.337796287	5.547575698	-18.71480469	1.54E-59	124.7462554
IL12RB1	-0.760369846	1.256023588	-18.69633467	1.88E-59	124.5438422

C1orf162	-1.070556989	2.868175558	-18.66055802	2.79E-59	124.1518627
GAS7	-0.970353166	2.0000322	-18.62802078	3.99E-59	123.7954879
SLC7A7	-1.043121515	2.85085204	-18.60799328	4.97E-59	123.5761841
AMICA1	-0.997627586	1.78484384	-18.56233079	8.22E-59	123.0763292
CD80	-0.522468552	0.718596121	-18.53016422	1.17E-58	122.7243418
TLR8	-0.947851154	1.270758343	-18.52165732	1.28E-58	122.6312721
ITGAM	-1.241386986	2.512764917	-18.51016407	1.46E-58	122.5055425
P2RX7	-0.848942205	1.404351111	-18.48012796	2.03E-58	122.1770313
CMKLR1	-1.064091021	2.052519112	-18.4766453	2.11E-58	122.138947
GLIPR1	-0.901133172	2.127319862	-18.42846914	3.58E-58	121.6122563
SIGLEC9	-0.831687322	1.495707101	-18.40649486	4.55E-58	121.3721046
ARHGAP9	-0.960504626	1.941395837	-18.40057492	4.86E-58	121.3074162
CD2	-1.384185085	3.455222563	-18.37712492	6.28E-58	121.0512111
DOCK8	-0.930868208	2.054763764	-18.36707677	7.01E-58	120.9414478
MAFB	-1.129840007	3.653432446	-18.27604815	1.90E-57	119.9475961
NFAM1	-0.954786972	1.802569323	-18.27174579	1.99E-57	119.9006462
GYPC	-0.933441165	2.648493308	-18.23732916	2.91E-57	119.5251496
MS4A4A	-1.259783809	3.063130432	-18.2307799	3.12E-57	119.4537108
CYTIP	-1.10946577	2.664207207	-18.20722452	4.04E-57	119.1968116
CD247	-0.895412623	1.522796782	-18.19721143	4.51E-57	119.0876268
TESPA1	-0.606237274	0.837972032	-18.15617671	7.07E-57	118.6403002
FLI1	-0.764245295	1.77829754	-18.12110542	1.04E-56	118.2581418
LRRC25	-0.986760492	2.248604182	-18.12102016	1.04E-56	118.2572129
CD180	-0.816204645	1.368171461	-18.11912546	1.06E-56	118.2365713
PIK3CG	-0.796950461	1.193633689	-18.08367665	1.56E-56	117.850459
HLA-DMB	-1.367529676	4.15983271	-18.04973547	2.27E-56	117.4809121
HCK	-1.282123627	3.360467109	-18.04124004	2.49E-56	117.3884373
FCGR1A	-0.941388506	1.631043247	-18.00408798	3.73E-56	116.9841336
ICOS	-0.728913498	0.980448609	-18.00385712	3.74E-56	116.9816218
GIMAP1	-0.697922614	1.303359607	-17.99550763	4.10E-56	116.8907833
PTGER4	-0.997383894	2.029614742	-17.98872167	4.42E-56	116.8169617
PIK3R5	-0.771754486	1.480306449	-17.97779269	4.97E-56	116.6980822
SAMHD1	-1.038978656	4.560712666	-17.94391415	7.20E-56	116.3296658

CLEC10A	-1.19135879	1.915775636	-17.93744926	7.73E-56	116.2593793
FYB	-1.154030282	2.503959071	-17.92798984	8.57E-56	116.1565456
ITGB2	-1.441035134	4.638198252	-17.91847525	9.51E-56	116.0531237
FGD2	-0.738403906	1.269834444	-17.91421553	9.96E-56	116.0068252
HLA-DRA	-1.610987986	9.824955596	-17.90088748	1.15E-55	115.8619786
PLXDC2	-1.0344091	2.767176502	-17.84789881	2.05E-55	115.2863369
TRBV20-1	-1.094749817	1.746366545	-17.8352458	2.36E-55	115.1489354
GIMAP7	-1.115523948	3.307651483	-17.81241205	3.02E-55	114.9010328
ARHGAP15	-0.629277746	1.106548131	-17.78354707	4.14E-55	114.5877492
PTPRO	-0.617511038	0.868725576	-17.7819435	4.22E-55	114.5703482
CD3E	-1.281710217	2.998527035	-17.77406367	4.59E-55	114.4848462
CD300C	-0.899335352	1.562510173	-17.77346483	4.62E-55	114.4783487
PSTPIP1	-0.808976155	1.456141476	-17.76373628	5.14E-55	114.3727989
SH2B3	-0.78043234	2.823793528	-17.70968372	9.26E-55	113.7865896
C1QA	-1.535663155	6.916069014	-17.64445884	1.88E-54	113.0797471
TNFSF8	-0.66977815	1.041767031	-17.63107699	2.18E-54	112.9348008
FGL2	-1.333536404	3.168580519	-17.61828676	2.50E-54	112.7962861
HLA-DPA1	-1.600358855	6.160151565	-17.61314736	2.65E-54	112.7406343
SH2D1A	-0.948380584	1.316548047	-17.60510937	2.89E-54	112.6536026
LST1	-1.203482569	2.857824027	-17.58398969	3.64E-54	112.4249717
NCF1	-0.781535433	1.21973382	-17.58067064	3.77E-54	112.3890471
HLA-DOA	-1.53063153	3.779620123	-17.55027676	5.25E-54	112.0601438
APBB1IP	-0.976077811	2.415421933	-17.51869716	7.40E-54	111.7185494
LAT2	-0.907702842	2.302884557	-17.47812239	1.15E-53	111.2798662
TYROBP	-1.359178311	6.120849782	-17.4736423	1.21E-53	111.2314434
FMNL3	-0.644128205	2.057147509	-17.46481839	1.33E-53	111.1360791
STK10	-0.75442689	2.424324964	-17.44029457	1.73E-53	110.8710985
CD52	-1.46778342	5.074449428	-17.43995387	1.74E-53	110.8674179
IL2RG	-1.315125211	3.93061561	-17.39815254	2.74E-53	110.4159599
HLA-DPB1	-1.520569028	6.917726755	-17.38075987	3.30E-53	110.2281942
SIGLEC7	-0.739150611	1.229717473	-17.37814187	3.40E-53	110.199935
SLAMF1	-0.712798605	1.069299947	-17.3578564	4.24E-53	109.9810047
TRAF3IP3	-0.749799483	1.142685635	-17.32083536	6.33E-53	109.5816165

TRBV28	-1.258900587	2.757982991	-17.30630552	7.41E-53	109.4249234
CD200R1	-0.491642847	0.699567755	-17.29511021	8.36E-53	109.3042126
LILRB1	-0.814168459	1.542631259	-17.27671867	1.02E-52	109.105952
FCGR3A	-1.464131072	4.836240186	-17.26915856	1.11E-52	109.0244691
C1QC	-1.524723367	6.85272403	-17.26053354	1.22E-52	108.9315195
SIRPB2	-0.657349038	1.073177246	-17.25461745	1.30E-52	108.86777
CD48	-1.275457569	2.691175745	-17.2535135	1.31E-52	108.8558748
NCF2	-1.18476539	3.727286679	-17.23258364	1.64E-52	108.6303897
SLAMF6	-1.069926326	1.90614499	-17.21327486	2.03E-52	108.4224296
HCST	-1.105584705	3.187107149	-17.20719752	2.16E-52	108.3569872
PARVG	-0.820785547	1.67784476	-17.20158836	2.30E-52	108.2965916
C1QB	-1.584717676	6.808900828	-17.20152223	2.30E-52	108.2958796
STAT5A	-0.735925949	2.736664891	-17.19744755	2.41E-52	108.2520093
TRBC2	-1.415747673	3.676321667	-17.17911134	2.93E-52	108.0546237
RAC2	-1.129279764	4.677519329	-17.16771969	3.32E-52	107.9320216
CXorf21	-0.806891623	1.57246241	-17.15941874	3.63E-52	107.842696
PTAFR	-1.178902558	2.880358152	-17.13170648	4.90E-52	107.5445668
CORO1A	-1.10381058	3.92013104	-17.06151412	1.04E-51	106.7899871
STX11	-0.905788036	2.037671038	-17.05159973	1.16E-51	106.6834701
ITK	-0.728060767	0.992047492	-16.99484653	2.15E-51	106.0740422
FGR	-1.052124709	2.661544433	-16.98380649	2.42E-51	105.9555537
KIAA0226L	-0.525643523	0.818892962	-16.97949948	2.53E-51	105.9093337
LCK	-1.085291549	2.301628663	-16.94632414	3.62E-51	105.5534213
FOLR2	-1.448712796	3.579651719	-16.90433727	5.69E-51	105.1032417
LSP1	-1.15228306	3.748688804	-16.8791388	7.46E-51	104.8332092
GAB3	-0.638607444	1.224399262	-16.82779135	1.30E-50	104.2832941
CASP1	-0.986887298	2.833479928	-16.80314224	1.69E-50	104.0194713
DAB2	-0.953665361	3.152388356	-16.79371642	1.87E-50	103.9186133
ALOX5AP	-1.419016825	4.253423016	-16.77392301	2.31E-50	103.7068707
VSIG4	-1.556551113	4.069408306	-16.75904282	2.71E-50	103.5477329
SLC1A3	-0.913550266	1.600599536	-16.75471058	2.84E-50	103.5014087
SIT1	-1.062360046	1.856662936	-16.7525816	2.91E-50	103.4786449
RGS18	-0.634130676	0.929724671	-16.74676621	3.10E-50	103.4164687

ENTPD1	-0.583662727	2.222929991	-16.74332997	3.21E-50	103.3797325
PRKCB	-0.785191333	1.338537111	-16.70898045	4.65E-50	103.0126222
MSR1	-1.292124988	2.778879174	-16.70425171	4.89E-50	102.9621002
TIGIT	-0.808924812	1.230915092	-16.66247976	7.65E-50	102.515981
VCAM1	-1.233948912	2.649701616	-16.66221234	7.68E-50	102.5131259
ALOX5	-1.231142619	3.593902638	-16.66121683	7.76E-50	102.5024979
NLRP3	-0.645429329	1.010819851	-16.62065614	1.20E-49	102.0696243
PTPN22	-0.929900111	1.679402202	-16.60145119	1.47E-49	101.8647683
PYHIN1	-0.623639519	0.805777799	-16.57617882	1.93E-49	101.5952949
LRRC8C	-0.565221332	1.362854629	-16.5751798	1.95E-49	101.5846451
RP11-121A8.1	-0.289631875	0.382115817	-16.53871687	2.88E-49	101.1960628
SLC15A3	-1.000181672	2.962704481	-16.5162429	3.67E-49	100.9566823
ICAM3	-0.707798969	1.110483968	-16.51081925	3.89E-49	100.8989266
TRAV13-1	-0.83931767	1.060732835	-16.49605663	4.55E-49	100.7417492
EPB41L2	-0.838151264	2.270180722	-16.47369991	5.78E-49	100.5037953
CRTAM	-0.623300187	0.804301035	-16.46746411	6.18E-49	100.4374412
BCL2A1	-1.195476528	3.072327085	-16.45963509	6.72E-49	100.3541444
TRAV9-2	-0.611207628	0.725383765	-16.44092526	8.20E-49	100.1551281
MRC1	-1.672214813	3.583135623	-16.42866822	9.35E-49	100.024786
GZMK	-1.239358362	1.865080372	-16.41731808	1.06E-48	99.90411337
NLRC4	-0.562902331	1.066569447	-16.41096326	1.13E-48	99.83656085
OSCAR	-1.128045625	2.475458507	-16.40880848	1.16E-48	99.81365698
LTA	-0.564466904	0.722754672	-16.40731747	1.17E-48	99.79780916
SLC31A2	-0.503983273	0.904296948	-16.38529248	1.49E-48	99.56375567
NCF4	-1.053371112	3.365048212	-16.38023247	1.57E-48	99.50999752
CARD8-AS1	-0.604382169	1.431532635	-16.33018379	2.68E-48	98.97854021
MS4A7	-1.118131487	2.848581508	-16.32571776	2.81E-48	98.93113993
LILRB2	-0.893833969	1.792798894	-16.32345766	2.87E-48	98.90715372
GIMAP2	-0.956447901	2.600479439	-16.30523765	3.49E-48	98.71382342
RASAL3	-0.935124781	1.996626007	-16.29086626	4.07E-48	98.56137625
PTGS1	-0.916273307	2.123298655	-16.28692327	4.24E-48	98.51955732
OLFML1	-0.815283861	1.802908318	-16.28637619	4.27E-48	98.51375526
GPR34	-1.071902501	2.134004126	-16.28138944	4.50E-48	98.46087121

KLHL6	-0.802419978	1.339447931	-16.28098347	4.52E-48	98.45656618
FCGR2A	-1.066337372	3.542875602	-16.25419775	6.01E-48	98.17259193
SAMD3	-0.251351002	0.274346019	-16.24862127	6.38E-48	98.11348961
TRAV8-2	-0.519791542	0.599501445	-16.24415346	6.69E-48	98.06614201
HSD11B1	-1.085431679	2.220983619	-16.22878404	7.88E-48	97.90329471
TRBV25-1	-0.532545122	0.654745715	-16.21364115	9.26E-48	97.74289349
LINC01094	-0.745825288	1.198672625	-16.19783985	1.10E-47	97.57556702
ITGA4	-0.80615965	1.634278812	-16.18669008	1.23E-47	97.45752759
LILRB4	-1.162902236	2.360137066	-16.17941764	1.33E-47	97.38054971
CCR1	-1.078402476	2.842251529	-16.16744473	1.51E-47	97.25384125
CD3D	-1.275033772	3.2602803	-16.10741822	2.87E-47	96.61902258
TRAV21	-0.685463681	0.824549691	-16.09868748	3.14E-47	96.52675061
HCLS1	-1.052580765	3.663191732	-16.08535127	3.62E-47	96.38583529
NCF1B	-0.593329892	0.837273291	-16.06512792	4.49E-47	96.17221762
TRAV8-6	-0.554799499	0.705495146	-16.05567484	4.96E-47	96.07239436
HVCN1	-0.64874362	1.747820196	-16.04611336	5.49E-47	95.97144529
APOL3	-0.998289957	2.649664026	-16.04034864	5.84E-47	95.91059111
CALHM2	-0.713624994	2.535845738	-16.0253893	6.84E-47	95.75270794
GIMAP8	-0.863308716	2.13716058	-16.01883357	7.33E-47	95.68353241
CLECL1	-0.490363729	0.647578903	-16.01821639	7.38E-47	95.67702044
CD3G	-0.810991988	1.337099814	-16.00916929	8.13E-47	95.58157212
TRAV12-3	-0.686850558	0.792584467	-16.00134869	8.83E-47	95.49907733
CD74	-1.432126798	9.885493956	-15.98693281	1.03E-46	95.34704651
P2RY10	-0.877000606	1.345753202	-15.98498161	1.05E-46	95.32647237
IL2RB	-1.089892756	2.458040849	-15.96733357	1.27E-46	95.14042166
GLIPR2	-1.037164167	3.438155908	-15.9478686	1.56E-46	94.93529247
SERPING1	-1.06665009	6.252609207	-15.93591419	1.77E-46	94.80935224
SLC9A9	-0.686244757	1.523814351	-15.91858241	2.12E-46	94.62681505
IL2RA	-1.036362509	1.839355643	-15.91538226	2.19E-46	94.59311825
F13A1	-1.546920977	3.129977767	-15.90996841	2.32E-46	94.53611673
SELL	-1.290283884	2.796498092	-15.89733435	2.66E-46	94.4031193
RTN1	-0.670312673	0.989092368	-15.89658075	2.68E-46	94.39518726
TRBV19	-0.829566955	1.183212925	-15.88363544	3.07E-46	94.25895078

BTN3A3	-0.802814489	2.797534343	-15.87459304	3.38E-46	94.16381
RUNX3	-0.955704591	2.311163457	-15.85623658	4.10E-46	93.97072407
TRBV5-1	-0.745657202	1.021816443	-15.85025168	4.37E-46	93.90778648
TRAT1	-0.66049985	0.800616327	-15.83637617	5.06E-46	93.76190059
CD5	-1.008476887	1.970255233	-15.83459463	5.16E-46	93.74317268
CD69	-1.115891086	2.040685774	-15.82165279	5.91E-46	93.60714599
FCGR2B	-0.85177629	1.577401035	-15.79565881	7.78E-46	93.33404366
CD300LF	-1.041167758	2.144269406	-15.79174314	8.10E-46	93.2929169
GVINP1	-0.625292601	0.833150077	-15.78318285	8.87E-46	93.20301909
DCN	-1.3339585	4.631157907	-15.77358603	9.82E-46	93.1022549
MAF	-0.802756378	2.691667482	-15.75164019	1.24E-45	92.87190543
KBTBD8	-0.448848096	0.749006382	-15.74780486	1.29E-45	92.83165973
RASSF2	-0.931766394	2.697109811	-15.73153203	1.53E-45	92.66093827
CXCL12	-1.141957057	2.586277554	-15.72735341	1.60E-45	92.61710904
DOCK10	-0.762638793	1.635245268	-15.72467501	1.64E-45	92.58901752
FOXP3	-0.779478267	1.61516866	-15.71922143	1.74E-45	92.53182436
C1R	-1.000456031	5.55009798	-15.71645408	1.79E-45	92.50280498
EMP3	-1.008156384	4.431008237	-15.70965767	1.93E-45	92.43154272
P2RY12	-0.660529013	0.759927972	-15.68210103	2.57E-45	92.14270918
CD163	-1.417120823	3.71675255	-15.68039831	2.62E-45	92.1248678
SIRPG	-0.836384148	1.316816381	-15.66240039	3.17E-45	91.9363223
CCR8	-0.505494345	0.659516018	-15.65320838	3.49E-45	91.84005522
IFFO1	-0.724890646	1.619246814	-15.64218323	3.92E-45	91.72461507
TRG-AS1	-0.459906571	0.643954614	-15.63096414	4.41E-45	91.60717251
B2M	-0.940254681	9.523345242	-15.60544372	5.77E-45	91.3401285
TRBV7-9	-0.925974782	1.391275367	-15.60397518	5.86E-45	91.32476635
TNFRSF1B	-0.899393852	4.072382493	-15.5886428	6.88E-45	91.1644058
ITGB7	-0.689065551	1.284076736	-15.5880282	6.93E-45	91.1579789
PREX1	-0.86993445	2.870744189	-15.57268267	8.14E-45	90.99753684
SIGLEC10	-1.009402672	1.936329049	-15.56657774	8.68E-45	90.93372296
KLRB1	-0.976097883	1.971878767	-15.5572598	9.57E-45	90.83634064
HLA-DRB1	-1.522674077	8.952200386	-15.53830257	1.17E-44	90.63827915
JAK2	-0.663855797	1.98083428	-15.52066553	1.41E-44	90.45408538

UBASH3A	-0.620180641	0.900174869	-15.51737911	1.45E-44	90.41977138
PMP22	-0.949832032	4.528661885	-15.50262397	1.70E-44	90.26574161
GPR174	-0.703126939	0.821232777	-15.48330561	2.08E-44	90.06415283
PLA2G5	-0.481973519	0.817526839	-15.47843611	2.19E-44	90.01335295
ATP8B4	-0.315922583	0.507197563	-15.46276967	2.58E-44	89.84995417
RP5-1091N2.9	-0.51564781	0.776965621	-15.46189495	2.60E-44	89.84083271
C10orf54	-0.789661621	3.333411302	-15.4567869	2.75E-44	89.78756997
GAL3ST4	-0.836001283	2.094531295	-15.39689353	5.14E-44	89.16350832
ETS1	-0.928697001	3.829411084	-15.37927902	6.18E-44	88.98013503
CD226	-0.30413496	0.421665577	-15.36300246	7.33E-44	88.81075627
TRAV12-1	-0.693742733	0.822900539	-15.35872703	7.67E-44	88.76627536
CTSS	-1.0167106	5.836737619	-15.3580762	7.72E-44	88.75950469
CASS4	-0.676066447	1.251520271	-15.30890282	1.29E-43	88.24823646
CCR4	-0.867841141	1.231077687	-15.28205893	1.71E-43	87.96938057
HNRNPA1P21	-0.706509779	1.256832921	-15.248504	2.42E-43	87.62105693
CD14	-1.199599544	5.463765608	-15.22700465	3.03E-43	87.39802369
TBC1D10C	-0.893342655	1.724453607	-15.22697818	3.03E-43	87.39774912
INPP5D	-0.889207992	2.356505198	-15.21235663	3.53E-43	87.24613096
MATK	-0.631054548	1.050909284	-15.20614374	3.77E-43	87.18172235
TRBV18	-0.613460214	0.797135543	-15.20482366	3.82E-43	87.16803842
CNRIP1	-0.604583675	1.537431234	-15.19588082	4.20E-43	87.07534834
NAALADL1	-0.417117476	0.848588539	-15.16340325	5.88E-43	86.73889416
CARD16	-0.837062319	2.345036563	-15.15606372	6.35E-43	86.662896
PIK3R6	-0.580538038	1.050737013	-15.14871386	6.86E-43	86.58680444
TRGV7	-0.471066756	0.621676566	-15.14271295	7.30E-43	86.52468831
RAB8B	-0.717348621	2.826374278	-15.1375405	7.70E-43	86.47115483
ABCD2	-0.286299458	0.323393065	-15.07904983	1.41E-42	85.86626111
FGD5	-0.675426561	1.757998039	-15.07825187	1.43E-42	85.85801481
CD6	-0.851640616	1.730636982	-15.07742803	1.44E-42	85.84950123
GAPT	-0.575962008	0.829611677	-15.06907647	1.57E-42	85.76320572
AC083949.1	-0.350885171	0.434135552	-15.06681398	1.61E-42	85.73983086
TRBV3-1	-0.666620725	0.848846772	-15.06259031	1.68E-42	85.69619747
NRROS	-0.745636402	1.919974886	-15.04379518	2.04E-42	85.50208607

CCR7	-1.055325313	1.859502443	-15.04189386	2.08E-42	85.48245476
C16orf54	-0.929666785	1.720522485	-15.00888915	2.93E-42	85.14182506
RASGRP4	-0.444731572	0.74843088	-15.0070201	2.99E-42	85.1225436
ITGAX	-1.002007711	2.701909479	-15.00622512	3.01E-42	85.11434273
CD40	-1.097944385	3.679475885	-15.00458358	3.06E-42	85.09740944
THEMIS	-0.589125072	0.741910559	-14.97482206	4.17E-42	84.79052467
DPEP2	-0.697478245	1.242948629	-14.96406049	4.66E-42	84.67961351
AGAP2	-0.507847183	0.968976776	-14.9606467	4.83E-42	84.64443651
TNFAIP8	-0.697423062	1.929392438	-14.95581114	5.08E-42	84.59461428
GPR183	-1.174906964	3.38407604	-14.93912274	6.04E-42	84.42271518
NAPSB	-1.181100085	2.883995757	-14.93520186	6.29E-42	84.38233868
TRAV29DV5	-0.519730323	0.612551441	-14.90767746	8.36E-42	84.09901008
HK3	-1.119266597	2.227658536	-14.90688766	8.43E-42	84.09088307
CXCR6	-0.852273262	1.569876066	-14.9065263	8.46E-42	84.08716475
TRAV12-2	-0.66827355	0.850102184	-14.89915994	9.13E-42	84.01137334
GBP4	-1.197314123	3.119941043	-14.89195891	9.84E-42	83.93729685
ACAP1	-0.831585165	1.73830656	-14.87931187	1.12E-41	83.80723049
RP11-23J18.1	-0.437142186	0.773520683	-14.83071085	1.85E-41	83.30779522
BTLA	-0.440908544	0.543908033	-14.80852914	2.33E-41	83.08005948
NCR3	-0.640084365	0.825965727	-14.80745857	2.35E-41	83.06907142
GNAI2	-0.581062014	5.362221102	-14.79866351	2.58E-41	82.97881305
CCL5	-1.453437837	4.680742897	-14.79239081	2.75E-41	82.91445266
RP11-327F22.2	-0.290811056	0.376413657	-14.77565042	3.27E-41	82.74274147
TRBV6-5	-0.664343837	0.940966891	-14.7728223	3.37E-41	82.71374004
FAM26F	-1.298127501	2.657399137	-14.7724582	3.38E-41	82.71000652
ADAP2	-0.710455918	2.48216954	-14.7637226	3.70E-41	82.62044032
TOMM20P2	-0.448683271	0.522835011	-14.75916459	3.87E-41	82.5737151
TRAV8-3	-0.661395024	0.86931923	-14.75867495	3.89E-41	82.56869604
SLC2A9	-0.370116558	0.725499507	-14.75238296	4.15E-41	82.50420551
GNGT2	-0.58219799	1.229003601	-14.74899306	4.30E-41	82.46946469
AC079767.4	-0.900163007	1.307171169	-14.73762887	4.84E-41	82.35302351
TLR1	-0.684272561	1.703433048	-14.73025967	5.22E-41	82.27753502
MILR1	-0.924260466	2.154361022	-14.7166616	6.00E-41	82.13827799

TMEM119	-1.104424181	3.192799178	-14.70524451	6.75E-41	82.02139515
VIM	-0.970699936	7.030459791	-14.69200091	7.74E-41	81.88585773
TRAV8-4	-0.494927549	0.644963861	-14.67305327	9.40E-41	81.69202768
GIMAP5	-0.373423985	0.468322003	-14.67258236	9.45E-41	81.6872116
DOK3	-0.733062721	2.009959266	-14.66711258	9.99E-41	81.63127598
FPR1	-1.062335937	2.368432294	-14.6587555	1.09E-40	81.54582983
CLEC7A	-1.035568217	2.559131593	-14.65825225	1.09E-40	81.54068499
ABCA6	-0.357713187	0.488124575	-14.64423356	1.26E-40	81.39739733
ARHGAP18	-0.737274811	3.497788555	-14.63006904	1.46E-40	81.25267414
TRBV9	-0.624825026	0.863029833	-14.62446903	1.55E-40	81.1954725
FAIM3	-1.027692438	2.428048965	-14.62446321	1.55E-40	81.19541308
LINC01150	-0.444385065	0.601446767	-14.61230095	1.76E-40	81.07121076
MAP4K1	-0.881349937	2.010840166	-14.59592388	2.08E-40	80.90403115
CXCR4	-1.058128386	5.122700263	-14.59320822	2.13E-40	80.87631648
AC147651.4	-0.637545802	1.287682956	-14.57690405	2.52E-40	80.70996755
TRAV4	-0.566243586	0.745866852	-14.56975633	2.72E-40	80.63706378
TRBV2	-0.62512773	0.786609787	-14.56269132	2.92E-40	80.56501782
DSE	-0.754911859	1.586296535	-14.55998279	3.00E-40	80.53740099
SLA2	-0.682225727	1.311539639	-14.55479286	3.17E-40	80.48448904
CD300A	-0.881125242	2.6339056	-14.55165765	3.27E-40	80.45252893
MFNG	-0.739475421	2.601603428	-14.54464564	3.51E-40	80.38105885
NLRC3	-0.629799351	1.18487352	-14.5328829	3.96E-40	80.26119792
HLA-E	-0.826672414	8.104930155	-14.51950329	4.54E-40	80.12490857
SLC37A2	-0.832963897	1.739396611	-14.51027163	5.00E-40	80.03090121
CPED1	-0.65019178	1.122387807	-14.4971502	5.71E-40	79.89732506
SH3BGRL	-0.685899948	5.45829995	-14.49315456	5.95E-40	79.85665918
CTLA4	-0.818000203	1.442578603	-14.49299283	5.96E-40	79.85501329
LYZ	-1.832281774	7.362967528	-14.4881383	6.27E-40	79.80561222
GPR84	-0.782376629	1.215614868	-14.48563179	6.43E-40	79.78010799
MIR155HG	-0.666663008	1.083584262	-14.4815846	6.70E-40	79.73893066
APOBEC3G	-0.867507318	2.049391604	-14.47155046	7.42E-40	79.63686047
LILRA1	-0.266842447	0.412753271	-14.46985915	7.55E-40	79.61965875
ANXA6	-0.762083855	4.472843214	-14.45462302	8.83E-40	79.46473462

AC109826.1	-0.250468092	0.407922771	-14.44887506	9.36E-40	79.40630534
CCL19	-1.89235481	4.162984586	-14.44455402	9.78E-40	79.36238728
SP140	-0.697206154	1.185768667	-14.42436656	1.20E-39	79.15727729
PPP1R16B	-0.86819155	1.707786532	-14.42275259	1.22E-39	79.14088395
OLR1	-1.353497789	3.136714948	-14.41181889	1.37E-39	79.02984857
SLC8A1	-0.385362022	0.715677994	-14.40455161	1.47E-39	78.95606594
FCGR1B	-0.313218248	0.429921284	-14.39442105	1.63E-39	78.8532387
C1S	-1.09117461	5.62849899	-14.39172957	1.68E-39	78.82592459
TREM2	-1.291676884	4.129758219	-14.3867344	1.77E-39	78.77523732
USP30-AS1	-0.761194847	1.344785958	-14.3835174	1.82E-39	78.7425975
TRAV26-1	-0.407443811	0.490365777	-14.36093349	2.30E-39	78.51354417
SIGLEC14	-0.835846937	1.562596569	-14.34875898	2.60E-39	78.39012777
FTH1P22	-0.639819557	0.865370303	-14.34519879	2.70E-39	78.35404526
NCF1C	-0.757009532	1.452085699	-14.33312929	3.05E-39	78.23174859
SIGLEC1	-1.030917155	2.104890531	-14.33117153	3.11E-39	78.21191519
EMILIN2	-0.77942885	2.430787101	-14.28846149	4.81E-39	77.77951365
ATP6V1B2	-0.546978676	3.949297044	-14.26977599	5.81E-39	77.59050797
CCL4	-1.068268993	2.477735493	-14.2268691	8.99E-39	77.15689156
ZNF831	-0.32411175	0.364056987	-14.21696659	9.94E-39	77.05689458
TRBV10-3	-0.610362519	0.70030103	-14.20668187	1.10E-38	76.95306905
ADORA3	-0.722564877	1.409411378	-14.20434052	1.13E-38	76.92943724
VAV1	-0.84260062	2.633631412	-14.19786538	1.21E-38	76.8640905
GM2A	-0.761127837	4.767667743	-14.19389405	1.26E-38	76.8240184
TRAV13-2	-0.482458745	0.578241466	-14.17586717	1.51E-38	76.64217995
BHLHE41	-1.019710491	2.453470155	-14.17345826	1.55E-38	76.61788852
KCTD12	-1.000144609	4.138887419	-14.17256215	1.56E-38	76.60885261
MYLK	-0.862149654	2.317742043	-14.15885943	1.79E-38	76.4707112
AXL	-0.965277029	3.411580545	-14.14808681	2.00E-38	76.3621488
LILRA4	-0.47080377	0.530104051	-14.13894389	2.19E-38	76.27003743
RASGRP3	-0.452055697	1.290866092	-14.12650028	2.49E-38	76.14471343
CD40LG	-0.64521685	0.929565569	-14.11632583	2.76E-38	76.04227785
CTSK	-1.253034653	4.980635181	-14.10476013	3.10E-38	75.92587335
CIITA	-0.939578331	2.093208593	-14.08347299	3.85E-38	75.71173273

HLA-B	-1.093733789	9.324387845	-14.05668536	5.04E-38	75.44245585
C1orf54	-0.649786282	2.62951485	-14.05490571	5.13E-38	75.42457406
RP5-899E9.1	-0.394727399	0.602037858	-14.03025467	6.59E-38	75.17698285
CR1	-0.448658434	0.602895795	-14.01509234	7.67E-38	75.02478772
CD209	-0.82341505	1.470427797	-14.01435269	7.73E-38	75.01736521
PPM1M	-0.598456774	3.10255067	-13.99840183	9.08E-38	74.85733538
HAPLN3	-0.920273457	2.50203586	-13.98176068	1.07E-37	74.6904642
GBP5	-1.160966845	2.075743812	-13.97887147	1.11E-37	74.66150104
RP3-477O4.14	-0.267927467	0.443076127	-13.95597606	1.39E-37	74.43207586
ABI3BP	-0.921173409	1.590297089	-13.94652733	1.53E-37	74.33744175
ANKRD44	-0.627650822	1.489928906	-13.94249705	1.60E-37	74.29708491
STAC3	-0.573712455	1.625759666	-13.93568079	1.71E-37	74.22884241
STARD8	-0.574597875	1.512291036	-13.91406648	2.12E-37	74.01254225
CSF2RA	-0.925795204	2.310124064	-13.89783196	2.50E-37	73.85017568
PTGIR	-0.520256958	1.238138965	-13.88059665	2.97E-37	73.67789094
TRBV12-4	-0.547689622	0.645801433	-13.86886592	3.35E-37	73.56068389
CD27	-1.159153053	2.707620854	-13.86308281	3.55E-37	73.50291824
LUM	-1.245806148	7.177430815	-13.84416012	4.29E-37	73.31397979
TRBV5-4	-0.551920955	0.635367331	-13.84223592	4.37E-37	73.29477344
TRAV17	-0.514606608	0.660680211	-13.81161687	5.95E-37	72.98931021
TMEM200A	-0.664403245	1.279190652	-13.80333394	6.46E-37	72.90672911
RP11-8L8.2	-0.254765124	0.35113823	-13.80192673	6.55E-37	72.8927014
TRBV29-1	-0.836577657	1.204187372	-13.79291042	7.17E-37	72.80283756
RP11-219E7.1	-0.570721608	0.991261932	-13.78683521	7.62E-37	72.74230189
PIK3CD	-0.736995604	2.530982537	-13.77868087	8.27E-37	72.66106756
BTN3A1	-0.741181594	3.413245247	-13.76655897	9.34E-37	72.54034767
TIFAB	-0.252643787	0.25893155	-13.75857906	1.01E-36	72.46090293
HLA-DOB	-0.998386313	2.26202454	-13.75092181	1.09E-36	72.38468986
C5AR1	-0.958444577	3.059396485	-13.72200379	1.46E-36	72.09703837
ZEB1	-0.601752281	1.768369603	-13.7094752	1.65E-36	71.97249914
SLAMF7	-1.234492907	3.056585333	-13.70722379	1.69E-36	71.95012461
DOCK11	-0.763086426	2.09362601	-13.7034739	1.76E-36	71.91286183
LILRB3	-0.513386967	1.10816207	-13.69650021	1.88E-36	71.84357638

DNAJC5B	-0.56814885	0.844929108	-13.67297492	2.38E-36	71.6099635
TRAF1	-0.721563232	2.108420017	-13.67141889	2.42E-36	71.59451802
TM6SF1	-0.582041297	1.221958733	-13.67008912	2.45E-36	71.58131909
P2RY8	-0.730759381	1.502837881	-13.66892994	2.48E-36	71.56981389
CCDC80	-1.154074701	3.196667928	-13.66836502	2.49E-36	71.56420705
KCNAB2	-0.713933756	2.178965719	-13.66762648	2.51E-36	71.55687721
AC011899.9	-0.390470619	0.622351355	-13.61930133	4.07E-36	71.07764963
CYSLTR2	-0.323306331	0.476386601	-13.61165393	4.39E-36	71.00188305
IL4I1	-1.170407531	2.681934739	-13.59744762	5.06E-36	70.86118556
SRGN	-1.146759364	6.603271174	-13.59677512	5.09E-36	70.85452679
P2RY14	-0.562800564	1.127173161	-13.57482046	6.34E-36	70.63722737
TRBV6-1	-0.579318585	0.745440573	-13.57151311	6.55E-36	70.60450634
SSPN	-0.745596236	1.649800402	-13.55886104	7.43E-36	70.47936759
EBI3	-0.756367681	1.550587873	-13.55372844	7.82E-36	70.42861738
SLFN12L	-0.382766307	0.59217501	-13.55193096	7.96E-36	70.41084637
LINC00426	-0.387157602	0.572848998	-13.54198715	8.79E-36	70.31255495
LINC00996	-0.429464144	0.576708484	-13.50033523	1.33E-35	69.90119968
IL9R	-0.255827391	0.309055464	-13.4963585	1.38E-35	69.86195594
TRBV6-6	-0.515337558	0.601899403	-13.49054562	1.46E-35	69.80460209
AD000864.6	-0.237307552	0.433298421	-13.48673592	1.52E-35	69.76701924
HLA-DRB5	-1.652840311	7.304510556	-13.46412275	1.90E-35	69.54404014
KCNMB1	-0.455629167	0.838887712	-13.45086811	2.17E-35	69.41342227
CD8A	-1.095088759	2.272802179	-13.43699249	2.49E-35	69.276749
CRYBB1	-0.342529974	0.633349117	-13.42788683	2.73E-35	69.187095
LACTB	-0.471190366	2.729886669	-13.41901569	2.98E-35	69.09977729
TRAV16	-0.489744056	0.602591357	-13.41008429	3.25E-35	69.01189362
MMP2	-1.227732763	5.836764961	-13.40661915	3.37E-35	68.97780456
FAS	-0.90924034	2.767109777	-13.40517153	3.41E-35	68.96356441
CD72	-0.637837439	1.444251845	-13.39085969	3.93E-35	68.8228192
FMO2	-1.062623628	2.274416685	-13.38486334	4.17E-35	68.7638709
PLEKHO1	-0.757732441	3.003707542	-13.38448323	4.19E-35	68.76013459
LTB	-1.229539327	3.342287313	-13.35767255	5.46E-35	68.49672188
FCGR1C	-0.45219628	0.644192903	-13.35621045	5.54E-35	68.482364

CCL13	-1.557870456	3.340174883	-13.34593825	6.13E-35	68.3815114
TMEM150B	-0.736500717	1.388761306	-13.32759818	7.35E-35	68.20153929
LMO2	-0.622427385	2.144242099	-13.32713984	7.38E-35	68.19704314
FUT7	-0.360639619	0.483117334	-13.32340834	7.66E-35	68.16044062
TRAV5	-0.423029077	0.475000768	-13.32005486	7.92E-35	68.12755024
LYL1	-0.547079894	1.49399508	-13.31552455	8.28E-35	68.08312387
CD1D	-0.577163126	1.278189111	-13.31142517	8.62E-35	68.04292958
CD101	-0.501404201	0.967424468	-13.28012483	1.17E-34	67.73622406
GPNMB	-1.266303892	5.24057216	-13.26919457	1.31E-34	67.62920138
TRBV4-2	-0.622985383	0.748293861	-13.25519432	1.50E-34	67.49218035
LIPA	-0.891055027	4.818319749	-13.23980465	1.75E-34	67.34164039
GRAP2	-0.475060452	0.796549962	-13.23319873	1.86E-34	67.27704766
FAM49A	-0.702151406	1.953273137	-13.2242785	2.03E-34	67.18985006
FCN1	-0.912767525	1.568618522	-13.20859031	2.37E-34	67.036562
EOMES	-0.558016531	0.714868435	-13.20471272	2.47E-34	66.99868772
COLEC12	-1.092480233	2.468204032	-13.1841242	3.02E-34	66.79767945
LINC00861	-0.469628854	0.555389222	-13.17968745	3.15E-34	66.75438266
ZAP70	-0.820703865	1.430040277	-13.16406957	3.68E-34	66.60202845
CXCR3	-0.936467692	1.960717153	-13.15672731	3.95E-34	66.53043389
MFSD1	-0.517838443	4.024204263	-13.14469357	4.45E-34	66.41313434
GBP1	-1.188237691	3.738035524	-13.1116169	6.15E-34	66.09098458
STAP1	-0.629543086	0.933603642	-13.10491026	6.56E-34	66.02571326
FLT3	-0.271648834	0.376385761	-13.10162213	6.78E-34	65.99371794
SIGLEC22P	-0.210522613	0.245647652	-13.06815051	9.41E-34	65.66824284
CD96	-0.825682897	1.52934832	-13.06584474	9.62E-34	65.64583662
GPR18	-0.457193547	0.619602158	-13.05651141	1.05E-33	65.55516018
IL15RA	-0.717959975	2.67718874	-13.05633759	1.06E-33	65.55347171
SUSD3	-0.782010707	2.251203206	-13.04494714	1.18E-33	65.44285316
LILRA6	-0.535599931	0.906116697	-13.04246723	1.21E-33	65.41877578
BHLHE22	-0.453945728	0.786607883	-13.03178442	1.34E-33	65.31508191
CLEC2B	-0.905695901	2.559212757	-13.01913668	1.52E-33	65.19236909
TRGV3	-0.311333926	0.368249314	-13.01804962	1.53E-33	65.18182476
RP11-1094M14.8	-0.673971584	1.169116485	-13.00211227	1.79E-33	65.02728443

CTSB	-0.760585496	7.513893539	-13.00196623	1.80E-33	65.02586869
THEMIS2	-0.789629936	3.378037229	-12.99305809	1.96E-33	64.93952971
SCML4	-0.313079924	0.362306792	-12.98226574	2.18E-33	64.83496768
AC133644.2	-0.415234646	0.70388015	-12.98005475	2.22E-33	64.81355176
TRBV7-3	-0.484635311	0.619091944	-12.97070555	2.44E-33	64.72301381
CST7	-1.106434986	3.338441877	-12.96577655	2.56E-33	64.67529425
TRIM22	-0.923488592	3.660046725	-12.93829274	3.34E-33	64.40937657
PTGDS	-1.440497363	4.308300751	-12.93440639	3.47E-33	64.37179696
SAMD9L	-0.967441241	2.669575269	-12.92891553	3.66E-33	64.31871181
TRAV23DV6	-0.41456804	0.463856812	-12.91754492	4.09E-33	64.20881729
RP11-1094M14.5	-0.491200262	0.690666201	-12.91283734	4.28E-33	64.16333363
FMNL1	-0.744966579	2.882376854	-12.90862574	4.46E-33	64.1226489
TNFAIP3	-0.906113031	3.491557609	-12.89657387	5.01E-33	64.00626248
CD244	-0.463959778	0.732931408	-12.89520446	5.08E-33	63.99304126
PLCG2	-0.574549736	1.52943419	-12.89192643	5.24E-33	63.961396
RP11-47L3.1	-0.551197449	0.940476408	-12.88523844	5.60E-33	63.89684403
TRBV7-6	-0.510856981	0.52295006	-12.88304796	5.72E-33	63.87570537
PTCRA	-0.370468324	0.459066678	-12.88080753	5.84E-33	63.85408652
KLRG1	-0.350393672	0.676017471	-12.87671588	6.08E-33	63.81460925
PECAM1	-0.722218852	4.50638995	-12.87148895	6.39E-33	63.76418761
IL18BP	-0.733037297	2.617637771	-12.85698363	7.36E-33	63.62431531
TRBV11-2	-0.496987786	0.665528652	-12.84884927	7.97E-33	63.54591156
DPT	-1.318292619	3.348570467	-12.84625389	8.17E-33	63.52090098
CD83	-0.878717618	3.388256533	-12.82545591	9.99E-33	63.32057086
PRF1	-1.036538656	2.57568626	-12.80156073	1.26E-32	63.09060887
GZMA	-1.25852881	3.298096694	-12.78490696	1.48E-32	62.93046406
NPL	-0.785311903	2.560496688	-12.78074957	1.54E-32	62.8905024
SIRPB1	-0.53395804	0.78424022	-12.77778552	1.59E-32	62.86201538
1-Sep	-0.750379716	1.929700738	-12.7731734	1.66E-32	62.81769564
RIN3	-0.585045698	2.285707189	-12.76483186	1.80E-32	62.73755885
LHFPL2	-0.743018475	3.006853836	-12.75681096	1.94E-32	62.66052738
RP11-399O19.9	-0.376593215	0.758168605	-12.75103368	2.05E-32	62.60505844
CD200	-0.636612489	1.97203862	-12.746976	2.14E-32	62.56610731

CD79B	-1.028278706	1.987110535	-12.74465679	2.18E-32	62.54384729
LPAR6	-0.63996557	2.29741072	-12.73079814	2.50E-32	62.41087275
RCBTB2	-0.52507249	2.088619797	-12.71982381	2.78E-32	62.30562546
TRAV20	-0.344875655	0.401298535	-12.71633003	2.87E-32	62.27212868
CPVL	-1.083391788	3.267874152	-12.71204527	2.99E-32	62.23105479
TRAV8-1	-0.33878394	0.367889485	-12.71099939	3.02E-32	62.22102994
RP11-652L8.4	-0.228169235	0.329444452	-12.70957935	3.06E-32	62.20741948
HS3ST3B1	-0.503394384	0.948996469	-12.70901363	3.08E-32	62.20199746
JAK3	-0.714692343	2.308006333	-12.70891755	3.08E-32	62.20107668
DAPP1	-0.801504471	2.198428959	-12.70523857	3.20E-32	62.16581975
CYLD	-0.481728883	2.323691986	-12.69345227	3.58E-32	62.05290257
TNFRSF9	-0.648985721	1.004846269	-12.69110346	3.66E-32	62.03040651
AP1S2	-0.65497433	2.233261978	-12.69088177	3.67E-32	62.02828334
TMEM140	-0.549995901	3.526589513	-12.68737176	3.80E-32	61.99466989
DDR2	-0.720885258	1.779483779	-12.68045077	4.06E-32	61.92840521
RP5-1028K7.2	-0.638665432	0.966747588	-12.67965006	4.09E-32	61.92074009
NKG7	-1.227727246	3.274053155	-12.66746612	4.60E-32	61.80413413
ACP5	-0.923042243	5.13226363	-12.66669116	4.63E-32	61.79671938
HLA-DMA	-1.073139275	5.809023614	-12.66473281	4.72E-32	61.77798294
PDCD1	-0.792210782	1.376714484	-12.65546585	5.16E-32	61.68934199
MSC	-0.858382169	2.024866876	-12.65119323	5.38E-32	61.64848437
PSMB8-AS1	-0.722215776	2.829697661	-12.64083622	5.94E-32	61.54947304
HLA-DQB1	-1.408265787	5.605688092	-12.63963185	6.01E-32	61.53796222
RHOG	-0.460410607	5.016551191	-12.62651489	6.82E-32	61.412632
EGR2	-0.840225334	2.101533492	-12.6254124	6.89E-32	61.40210088
LLNLR-470E3.1	-0.60155777	1.028282232	-12.62136404	7.17E-32	61.36343473
FGD3	-0.640811161	1.787788586	-12.62053923	7.23E-32	61.35555769
BZRAP1-AS1	-0.21431794	0.372008574	-12.6199943	7.26E-32	61.35035371
PEA15	-0.534122147	6.090645226	-12.61692754	7.48E-32	61.32106874
SOWAHD	-0.46414573	0.958640136	-12.60743954	8.20E-32	61.23048956
PLA2G2D	-1.264679337	1.753969754	-12.57503422	1.12E-31	60.92138956
TRBJ2-1	-0.884956541	1.180048863	-12.56359021	1.25E-31	60.81232827
ISLR	-1.146261897	4.552588137	-12.5551479	1.35E-31	60.7319059

APOL4	-0.798037493	2.009951004	-12.55022799	1.42E-31	60.68505124
TRAV6	-0.301669007	0.342809448	-12.54321966	1.52E-31	60.61832399
CCND2	-0.830029214	2.553175273	-12.54209604	1.53E-31	60.6076276
CECR1	-0.946588929	3.88682771	-12.5389558	1.58E-31	60.57773663
LY9	-0.418297049	0.667932993	-12.53584746	1.63E-31	60.54815303
SERPINB9	-0.778000415	2.849660287	-12.53523314	1.64E-31	60.54230676
GBP2	-0.875134881	3.905435331	-12.52721015	1.77E-31	60.46596764
RP11-848P1.3	-0.616011188	1.783851244	-12.52612361	1.79E-31	60.45563109
PTGDR	-0.234526799	0.299556762	-12.50701974	2.15E-31	60.2739672
NFATC2	-0.599769478	1.850944076	-12.50455359	2.20E-31	60.25052638
CLEC12A	-0.72588285	1.098120351	-12.49924399	2.31E-31	60.20006672
LINC01272	-0.857587745	2.19435457	-12.492883	2.46E-31	60.13962984
RP11-693N9.2	-0.44278118	0.863332391	-12.48786117	2.58E-31	60.09192789
AC006129.2	-0.60368742	0.968874164	-12.47959649	2.79E-31	60.01344407
AKNA	-0.645980763	2.55874273	-12.46164876	3.31E-31	59.8431007
CHST11	-0.782337502	2.755129884	-12.46154908	3.32E-31	59.84215497
RASSF4	-0.752062229	2.839587778	-12.45957294	3.38E-31	59.82340715
MPP1	-0.708893583	2.43103007	-12.44868062	3.75E-31	59.7200986
ECM2	-0.662157453	1.601312115	-12.4062627	5.62E-31	59.31823594
NECAP2	-0.423195113	4.010488518	-12.39942975	6.00E-31	59.25356874
PRAM1	-0.62638149	1.20881372	-12.39750447	6.11E-31	59.23535115
RASGRP2	-0.625901039	1.056730027	-12.38310379	7.01E-31	59.09913521
IL10	-0.377675027	0.573024217	-12.34309785	1.03E-30	58.72115778
SYNE3	-0.331417958	0.589839335	-12.33227343	1.14E-30	58.61899955
FGF7	-0.697947718	1.312555568	-12.32277233	1.24E-30	58.52936964
CXCL9	-1.77641474	4.258533023	-12.31607378	1.33E-30	58.46619988
TRBV5-6	-0.53243665	0.675819988	-12.31004737	1.40E-30	58.4093843
ARID5A	-0.595289325	2.999509983	-12.30333572	1.50E-30	58.34612591
RAB33A	-0.439522038	0.880747384	-12.29206512	1.67E-30	58.23993976
FNBP1	-0.610164219	3.12484561	-12.28974435	1.70E-30	58.2180811
RP11-1334A24.6	-0.482053603	1.081709451	-12.26755795	2.10E-30	58.00922411
GZMH	-1.057991611	2.205950387	-12.26704825	2.11E-30	58.00442835
MCOLN2	-0.620258203	1.299045176	-12.2625341	2.20E-30	57.96195892

CD44	-0.909510436	4.888571349	-12.24343715	2.64E-30	57.78238598
TSHZ3	-0.531791412	1.149883304	-12.24307507	2.65E-30	57.77898261
C19orf35	-0.218962779	0.376604796	-12.24163641	2.69E-30	57.76546089
STAT4	-0.558769901	1.449383726	-12.22510342	3.14E-30	57.61013065
PLA2G4C	-0.477893708	1.001082376	-12.21953178	3.31E-30	57.55780954
RECK	-0.495693766	1.411687882	-12.21077651	3.60E-30	57.47561793
SNX10	-0.868707085	3.105458379	-12.2088465	3.66E-30	57.45750395
PDGFRA	-0.824300841	2.234431406	-12.20840755	3.68E-30	57.45338439
COTL1	-0.841786386	4.465010764	-12.18432004	4.62E-30	57.22744591
MOXD1	-0.967302896	2.943556712	-12.17781533	4.91E-30	57.16647357
ZCCHC24	-0.599805714	2.689137765	-12.17321592	5.13E-30	57.12337129
BTN3A2	-0.742294749	3.228007482	-12.17297744	5.14E-30	57.12113666
LINC00892	-0.29357761	0.370527414	-12.15991823	5.81E-30	56.99880453
CXCL10	-1.574665255	4.235459288	-12.15887893	5.87E-30	56.9890719
HLA-C	-0.848757398	8.957578914	-12.15282217	6.21E-30	56.93236189
GPSM3	-0.806419037	4.424164298	-12.14621225	6.61E-30	56.87049004
LAP3	-0.60071082	5.028320141	-12.12671389	7.95E-30	56.68808256
TRBV14	-0.360520462	0.41991146	-12.12564345	8.03E-30	56.67807323
RP4-742C19.12	-0.32422412	0.37718133	-12.12557984	8.03E-30	56.67747842
TRAV39	-0.395551885	0.518614019	-12.10624519	9.64E-30	56.49676857
COL6A3	-1.109270299	4.913807872	-12.10572952	9.68E-30	56.49195103
TNFSF12	-0.611862098	3.825144055	-12.10154702	1.01E-29	56.45288112
TRAV14DV4	-0.450080573	0.546822163	-12.08975049	1.13E-29	56.34272586
TRAV19	-0.555013363	0.827088236	-12.08624622	1.16E-29	56.31001455
LILRB5	-0.589810653	0.928164785	-12.08347621	1.19E-29	56.28416091
ADAM19	-0.784386382	2.166546193	-12.08014519	1.23E-29	56.25307546
TCF4	-0.556513397	1.600524335	-12.07920004	1.24E-29	56.24425603
FEZ1	-0.461042746	1.082841981	-12.07880545	1.25E-29	56.2405741
TRGV10	-0.531155452	0.704731936	-12.06738896	1.39E-29	56.13407601
GPR68	-0.886474481	2.275439708	-12.06673176	1.40E-29	56.12794698
LAX1	-0.811244251	1.368688843	-12.06370928	1.44E-29	56.09976198
RRN3P2	-0.276230512	0.518175047	-12.06270764	1.45E-29	56.09042242
TRAV35	-0.302180072	0.325385312	-12.05132029	1.61E-29	55.98427329

ARHGDIB	-0.81459798	6.705078262	-12.04079515	1.78E-29	55.8862101
MFAP4	-1.388652821	4.82522627	-12.03640671	1.86E-29	55.84533674
TRAV41	-0.435453166	0.600790519	-12.03190002	1.94E-29	55.80337041
TIMP2	-0.900946013	5.625767527	-12.02465155	2.07E-29	55.73589069
PRKG1	-0.511681087	1.194171622	-12.02306134	2.11E-29	55.72108958
HPGDS	-0.82145162	1.388019338	-12.02230013	2.12E-29	55.71400491
SVEP1	-0.719141699	1.196197989	-12.02022509	2.16E-29	55.69469348
PLA2G7	-0.934600734	2.914206592	-12.01870076	2.19E-29	55.68050836
CAMK4	-0.26353709	0.403298082	-12.00301359	2.54E-29	55.53458441
RP11-18H21.1	-0.21734096	0.274868922	-12.00042317	2.60E-29	55.51049813
MSRB3	-0.736160267	2.160194719	-11.98527143	3.00E-29	55.36967093
JAZF1	-0.510394871	2.030026765	-11.94534659	4.36E-29	54.99906076
PRRX1	-0.852800309	2.448294079	-11.93628473	4.74E-29	54.9150373
PPP1R18	-0.749223439	4.105612199	-11.92087677	5.48E-29	54.77225255
CCL18	-1.571572634	5.459882551	-11.90644741	6.26E-29	54.63862902
TRBJ2-7	-0.943090242	1.438552233	-11.9009098	6.60E-29	54.58737167
STAB1	-0.793927771	2.796636265	-11.88893261	7.38E-29	54.47655348
ITM2A	-0.879783717	3.064521983	-11.88669648	7.53E-29	54.45587068
ASB2	-0.426690509	0.713273823	-11.88259112	7.82E-29	54.41790424
EMILIN1	-0.980759227	4.335561078	-11.87390239	8.48E-29	54.33757497
RPL7AP64	-0.361122754	0.562642731	-11.87279436	8.57E-29	54.32733339
TRGV5	-0.263264173	0.321400776	-11.86576868	9.15E-29	54.26240665
IL3RA	-0.625134033	2.780160521	-11.86424162	9.28E-29	54.24829734
TRAV22	-0.336587744	0.383176414	-11.8590298	9.74E-29	54.20015047
CD22	-0.758122828	1.185121365	-11.85793344	9.84E-29	54.19002369
DCSTAMP	-0.526736728	0.649409495	-11.85604902	1.00E-28	54.1726192
SH2D3C	-0.594616997	2.179678398	-11.83826963	1.18E-28	54.00848461
WNT2	-0.837529825	1.792277702	-11.82454734	1.34E-28	53.8818982
CCL3	-0.936302818	2.436925614	-11.82264223	1.37E-28	53.86433026
CEACAM21	-0.6052223	1.200792507	-11.81909579	1.41E-28	53.83163111
MARCO	-1.641648877	3.978830018	-11.81642953	1.45E-28	53.80705107
PRDM1	-0.712482345	2.419723287	-11.8126799	1.50E-28	53.77248876
FCRL3	-0.536097349	0.610036272	-11.80445232	1.62E-28	53.69667237

TRANK1	-0.606447814	2.17844701	-11.79126105	1.83E-28	53.57517803
NOD2	-0.424274511	1.153233746	-11.78638132	1.91E-28	53.53025395
RASGEF1B	-0.481171593	1.591528888	-11.78606442	1.92E-28	53.52733683
ACVRL1	-0.623382421	2.768970714	-11.78266648	1.98E-28	53.49606121
TRBJ2-3	-0.859507325	1.194260562	-11.7744648	2.14E-28	53.42059133
TRGV4	-0.271085003	0.316837619	-11.77029844	2.22E-28	53.38226483
IL15	-0.382723477	0.845708166	-11.7536487	2.59E-28	53.2291796
TRBV12-3	-0.400483751	0.422574751	-11.7400333	2.94E-28	53.10408414
RP11-686D22.10	-0.520366385	0.947647678	-11.7381582	2.99E-28	53.08686251
TRAV27	-0.37203183	0.390112452	-11.72700846	3.32E-28	52.98449132
CLEC5A	-0.848899317	1.665791329	-11.72478119	3.39E-28	52.96404827
TRBV15	-0.325064933	0.365675849	-11.72397296	3.41E-28	52.95663039
C5orf56	-0.385380821	0.971513978	-11.70712458	3.99E-28	52.80206414
AC021188.4	-0.233598338	0.354045036	-11.7058641	4.03E-28	52.79050565
HIVEP3	-0.415234434	1.05746877	-11.70376664	4.11E-28	52.77127369
RP11-1008C21.1	-0.566481052	0.721978334	-11.70158856	4.20E-28	52.75130445
ARHGAP31	-0.706356287	2.190501658	-11.69503553	4.46E-28	52.69123737
CLEC1A	-0.372307963	0.881868071	-11.69118282	4.62E-28	52.65593116
TRAV36DV7	-0.338181036	0.428239692	-11.68750066	4.78E-28	52.62219409
TRBJ2-2P	-0.808351091	1.043592242	-11.68726731	4.79E-28	52.62005629
TRBV4-1	-0.55513297	0.657192159	-11.68545151	4.87E-28	52.60342169
FLT3LG	-0.301471743	0.754680703	-11.67919462	5.16E-28	52.54611361
IL24	-0.567101241	1.119298453	-11.6711405	5.56E-28	52.47237009
TRAV25	-0.332335616	0.373722949	-11.67046536	5.59E-28	52.46618979
TRAV10	-0.289333801	0.310409007	-11.66016961	6.15E-28	52.37196707
RP11-222K16.2	-0.367437055	0.47358224	-11.6570533	6.33E-28	52.34345713
CAMK1G	-0.239881108	0.478328676	-11.64753622	6.91E-28	52.25641579
TBX5-AS1	-0.52351072	1.116046537	-11.64562135	7.03E-28	52.23890768
C19orf38	-0.567156038	1.388574255	-11.6395376	7.44E-28	52.18329333
MRGPRF	-0.667323169	1.548719557	-11.63735589	7.59E-28	52.16335339
RASA3	-0.661793615	2.412776966	-11.61551984	9.28E-28	51.96389811
P2RX1	-0.513165267	0.920715257	-11.61524218	9.30E-28	51.96136329
CLEC2D	-0.572502513	1.535196195	-11.60033485	1.07E-27	51.82532141

MITF	-0.509220032	1.508775732	-11.59616544	1.11E-27	51.78728989
APOBR	-0.790297583	2.343700072	-11.58331745	1.25E-27	51.67014561
CLMP	-0.893832868	1.991185877	-11.5711454	1.39E-27	51.55923311
TRAV3	-0.416263411	0.590197416	-11.5707979	1.40E-27	51.55606764
MEF2C	-0.633098939	1.984206612	-11.55566939	1.61E-27	51.41831133
CCL2	-1.04103464	4.222964861	-11.54988673	1.70E-27	51.36568335
IRF1	-0.759694662	3.513766559	-11.54913455	1.71E-27	51.35883884
RGS1	-1.151729953	4.178608086	-11.53873829	1.88E-27	51.26426412
SIGLEC8	-0.505872157	0.735076242	-11.53483914	1.95E-27	51.22880629
GFI1	-0.517149904	1.011281394	-11.53037832	2.03E-27	51.18824918
OSTM1	-0.467258331	2.834347429	-11.52696911	2.09E-27	51.1572592
COL6A5	-0.440615373	0.534322778	-11.52417082	2.15E-27	51.13182656
CACNA2D4	-0.244619292	0.573958115	-11.5238885	2.15E-27	51.12926086
LAMA2	-0.699797049	1.820943352	-11.51088419	2.42E-27	51.0111177
FKBP15	-0.376228693	2.705106143	-11.48063923	3.20E-27	50.7366429
TRBV27	-0.387834071	0.444885562	-11.46871317	3.56E-27	50.62852826
HLA-F	-0.965061338	4.519475119	-11.46483551	3.69E-27	50.59338965
RP11-389C8.2	-0.428324695	1.093783464	-11.45681801	3.97E-27	50.52075862
POU2F2	-0.57245661	1.232696402	-11.44121446	4.58E-27	50.3794897
DPYD	-0.892669454	3.139630548	-11.42783492	5.18E-27	50.25844539
IRF4	-0.84662827	1.509173321	-11.42678346	5.23E-27	50.24893633
TGM2	-0.937964567	5.728395317	-11.42498588	5.31E-27	50.23268091
PSMB9	-0.992848581	4.205216767	-11.42043229	5.54E-27	50.19150946
TREML1	-0.330842951	0.513686368	-11.41459885	5.84E-27	50.13878024
APOL6	-0.663675899	3.46583627	-11.41301277	5.92E-27	50.1244462
TMC8	-0.750862276	2.600709622	-11.40709504	6.25E-27	50.07097547
PDPN	-0.998418786	2.845033651	-11.39622313	6.90E-27	49.97278256
LRMP	-0.66211233	1.268396124	-11.39223854	7.16E-27	49.93680829
CPA3	-1.328235269	3.231414983	-11.38883142	7.38E-27	49.90605343
CARD8	-0.382567285	1.769956683	-11.3838371	7.73E-27	49.86098114
GPR132	-0.664982421	1.763249787	-11.36970033	8.79E-27	49.73346382
UBE2L6	-0.781962583	5.418649029	-11.36923914	8.82E-27	49.72930533
FIBIN	-0.91116648	2.098802302	-11.35832604	9.74E-27	49.63093213

LXN	-0.659796664	3.551641934	-11.34430249	1.11E-26	49.50460198
TPSAB1	-1.179426091	2.972246105	-11.33332774	1.22E-26	49.4058008
TNFSF4	-0.529370395	1.079590996	-11.32967262	1.26E-26	49.37290768
SGCD	-0.461507395	0.812601379	-11.32816792	1.28E-26	49.35936842
ZYX	-0.640927762	5.689065749	-11.32641431	1.30E-26	49.34359081
ZNF267	-0.385302458	1.998143379	-11.31753778	1.41E-26	49.26374877
PIP4K2A	-0.590728812	3.352790487	-11.31517793	1.44E-26	49.24252881
TRBV30	-0.561049682	0.697495315	-11.3031274	1.61E-26	49.13420985
FBN1	-0.926393033	2.814236622	-11.28491977	1.90E-26	48.9706756
N4BP2L1	-0.519707922	1.68932299	-11.23848034	2.88E-26	48.55428007
CTSZ	-0.605177657	7.474858099	-11.23777558	2.90E-26	48.54796875
BMP2K	-0.352160114	1.082869936	-11.23445814	2.99E-26	48.51826319
LRRK1	-0.417519137	1.273905571	-11.23067303	3.10E-26	48.48437644
MS4A1	-1.092330898	1.454760394	-11.2293457	3.13E-26	48.47249484
CXCL13	-1.596027501	3.737578819	-11.22575858	3.24E-26	48.4403891
SYNPO2	-0.508759174	1.034133808	-11.22040369	3.40E-26	48.39247262
MRVI1	-0.583010498	1.614458645	-11.21909784	3.44E-26	48.38078968
TGFB1	-0.704426695	4.529146096	-11.20799296	3.80E-26	48.28147155
FCRLA	-0.756105827	1.028584043	-11.20533127	3.89E-26	48.257675
FBLN1	-0.993622654	4.261925658	-11.19684024	4.20E-26	48.18178447
CCL23	-0.690484113	1.112446815	-11.19426383	4.30E-26	48.15876396
IL18R1	-0.47806282	1.033642748	-11.18983196	4.47E-26	48.11917219
LSAMP	-0.517341534	1.032792688	-11.17761557	4.99E-26	48.01008624
GBP1P1	-0.75145495	1.506900326	-11.17619694	5.06E-26	47.99742325
FASLG	-0.562095206	0.816530088	-11.17235707	5.24E-26	47.96315254
GPR82	-0.287535878	0.444131489	-11.16595338	5.55E-26	47.90601551
SIGLEC11	-0.26549461	0.369066471	-11.15752809	5.98E-26	47.83087048
BTN2A2	-0.473414128	2.125421382	-11.14823565	6.50E-26	47.7480307
AOC3	-0.964487615	3.333101691	-11.14553387	6.66E-26	47.72395279
TRAV38-1	-0.235313214	0.247745042	-11.13175676	7.54E-26	47.60122725
FAP	-0.793349733	1.864618164	-11.11732322	8.59E-26	47.47275189
RAI2	-0.687169346	2.099042733	-11.11402211	8.84E-26	47.44338226
KCNK13	-0.425096378	0.703216901	-11.11062204	9.12E-26	47.4131376

RARRES1	-1.170120763	3.424423432	-11.10099163	9.94E-26	47.32750247
TMEM52B	-0.288963821	0.46289	-11.10071285	9.96E-26	47.32502414
SPON1	-0.988492115	3.065730327	-11.09838823	1.02E-25	47.30436032
CCRL2	-0.633263634	1.673969676	-11.09618828	1.04E-25	47.28480704
SOCS1	-0.778463147	2.840728681	-11.09377249	1.06E-25	47.263338
PLCL2	-0.517354429	1.391336635	-11.09174342	1.08E-25	47.24530795
CSGALNACT2	-0.449963245	3.291245103	-11.0719647	1.29E-25	47.06966088
C1QTNF1	-0.74451337	2.354645481	-11.0718797	1.29E-25	47.06890642
KLRD1	-0.269759266	0.337369704	-11.06868029	1.33E-25	47.04051155
ABCA9	-0.260690496	0.408053879	-11.06722818	1.34E-25	47.02762572
ARSB	-0.410686033	2.027665678	-11.06630988	1.36E-25	47.01947734
CSF1	-0.85006552	3.548634256	-11.05495816	1.50E-25	46.91878363
FMO3	-0.770545194	1.839180057	-11.04842552	1.59E-25	46.86086508
EPSTI1	-0.773582166	2.563085014	-11.04488831	1.64E-25	46.82951277
S1PR1	-0.713516423	2.913427784	-11.02511001	1.96E-25	46.65431823
TLR10	-0.620778885	0.864727202	-11.01496856	2.15E-25	46.56455988
CCL4L1	-0.837433839	1.970935046	-11.01302247	2.18E-25	46.54734143
FAM26E	-0.363549627	0.735102586	-11.00969402	2.25E-25	46.51789656
PLD4	-0.48372375	0.824262868	-11.00886463	2.27E-25	46.51056029
GJA5	-0.800652135	2.978534949	-11.00758874	2.29E-25	46.49927515
S1PR4	-0.660304281	2.082549948	-10.99980682	2.46E-25	46.4304622
TNS3	-0.626453777	3.732542629	-10.99182412	2.64E-25	46.35990441
AF127936.5	-0.289478378	0.420801318	-10.9870701	2.75E-25	46.31789918
BNC2	-0.336752172	0.569908261	-10.98130745	2.90E-25	46.26699682
RASSF5	-0.615227923	2.923097654	-10.97100258	3.17E-25	46.17601287
PDZRN3	-0.545991312	1.324432839	-10.96918146	3.23E-25	46.15993921
GLT8D2	-0.680853951	1.756358741	-10.96719687	3.28E-25	46.14242461
TRBV21-1	-0.238872421	0.219092377	-10.96497867	3.35E-25	46.12285057
ATG7	-0.310043143	2.043123467	-10.9629818	3.41E-25	46.1052317
PLSCR4	-0.632328693	2.566099699	-10.96012811	3.50E-25	46.08005619
LILRA5	-0.655340098	1.466170814	-10.95850415	3.55E-25	46.06573132
LPAR5	-0.576928662	1.454865939	-10.95637975	3.62E-25	46.04699406
RP11-356I2.4	-0.280212277	0.558095992	-10.95190751	3.76E-25	46.00755592

RP4-728D4.2	-0.29664029	0.613067664	-10.94228109	4.10E-25	45.92269931
GPR141	-0.355220364	0.548083334	-10.93803725	4.26E-25	45.88530445
BICC1	-0.751712254	1.739488847	-10.93383583	4.42E-25	45.84829217
BCL11B	-0.478108178	0.886382493	-10.92777591	4.66E-25	45.79492276
CXCL11	-1.125688174	1.848112399	-10.89610614	6.18E-25	45.51630326
LTBP2	-0.963538622	4.411987841	-10.89344739	6.32E-25	45.49293504
CD300LB	-0.284066141	0.532028263	-10.89313974	6.34E-25	45.49023129
COL8A2	-0.952896165	2.543903142	-10.89253789	6.37E-25	45.48494206
CD93	-0.77179212	3.612826269	-10.89252306	6.38E-25	45.48481171
PTPN6	-0.437227743	3.965414989	-10.89153761	6.43E-25	45.47615184
HCP5	-0.888431896	4.000871473	-10.88286107	6.95E-25	45.39992479
C11orf21	-0.374982695	0.554224515	-10.88281422	6.95E-25	45.39951329
IGFBP7	-0.749801366	6.92075945	-10.87545387	7.42E-25	45.33487897
GSTM5	-0.329121254	0.559439346	-10.87030637	7.76E-25	45.28969253
APOC1	-1.156371773	5.86686879	-10.86676549	8.01E-25	45.25861719
FAM159A	-0.272999477	0.46709166	-10.85575327	8.83E-25	45.16201199
DUSP2	-0.750012911	2.400424124	-10.85404115	8.96E-25	45.14699773
APOBEC3C	-0.74850162	4.106481023	-10.83684058	1.04E-24	44.99624024
QKI	-0.472366777	2.681470829	-10.82832452	1.13E-24	44.92165426
OR2I1P	-1.257227925	2.456845228	-10.82525343	1.16E-24	44.89476569
CYP1B1	-0.970278481	3.386916203	-10.80666547	1.36E-24	44.7321215
FCGR2C	-0.613293408	1.205974747	-10.80497564	1.38E-24	44.71734407
RHOJ	-0.510096646	1.851422226	-10.77899671	1.74E-24	44.49034089
IFNGR1	-0.592394399	5.447463395	-10.77406793	1.82E-24	44.44731156
WISP1	-0.760928267	1.932290702	-10.77399481	1.82E-24	44.44667333
CTSO	-0.655621266	4.113695806	-10.77363925	1.82E-24	44.44356967
GIT2	-0.279441707	1.661547915	-10.76908844	1.90E-24	44.40385189
XCL2	-0.614348691	1.035174623	-10.76584661	1.95E-24	44.37556481
SPIB	-0.727251627	0.942486724	-10.76037423	2.05E-24	44.32782659
IKZF3	-0.670967319	1.87867687	-10.75847685	2.08E-24	44.3112784
CAPG	-0.681560795	6.296377519	-10.75742801	2.10E-24	44.30213164
PIM2	-0.901155381	4.524415757	-10.75520758	2.14E-24	44.28276931
TGFB3	-0.636303568	2.393754006	-10.73219463	2.62E-24	44.08224157

PTRF	-0.777858341	5.46458766	-10.73137124	2.64E-24	44.07507178
UNC5C	-0.351042282	0.70967209	-10.72668781	2.75E-24	44.0342965
RP11-118B22.4	-0.278022794	0.322891649	-10.72588415	2.77E-24	44.02730068
AC020571.3	-0.354226047	0.678424422	-10.7227367	2.85E-24	43.99990561
LAMA4	-0.689813134	2.57879811	-10.72147294	2.88E-24	43.98890737
NEXN	-0.588987616	1.758606436	-10.71866583	2.96E-24	43.9644806
SPARCL1	-0.884630777	5.310786946	-10.71692011	3.00E-24	43.9492918
ARHGEF3	-0.400000973	2.731072359	-10.69049895	3.78E-24	43.71960026
AIM2	-1.160552348	1.976827934	-10.68089176	4.12E-24	43.63616827
PDE4B	-0.573950528	1.704542857	-10.67084668	4.50E-24	43.54898373
ZNF366	-0.300541775	0.554295857	-10.66817226	4.60E-24	43.52578022
CD79A	-1.356595797	3.787152902	-10.66648506	4.67E-24	43.51114377
CASP5	-0.265767191	0.37748014	-10.66030394	4.93E-24	43.45753501
CD274	-0.891477261	1.911321449	-10.65752837	5.05E-24	43.43346888
SPOCK2	-0.926352847	3.284569638	-10.65133568	5.33E-24	43.37978815
MAN2B1	-0.469398045	4.506320616	-10.64109627	5.83E-24	43.29107171
RHOH	-0.557680924	1.356804288	-10.62984617	6.43E-24	43.19366018
A2M	-0.990665334	7.083376149	-10.62366671	6.79E-24	43.14018152
TCL1A	-0.554025782	0.578345058	-10.60321825	8.12E-24	42.96335456
S100B	-1.152702302	2.414621366	-10.60229797	8.18E-24	42.95540158
PXK	-0.366286323	1.981328179	-10.59878653	8.44E-24	42.92505981
BIRC3	-0.999386021	3.373086222	-10.59624788	8.63E-24	42.90312775
CERKL	-0.576489716	1.998668691	-10.59537322	8.69E-24	42.89557211
PRKCH	-0.421104369	2.266537032	-10.59152857	8.99E-24	42.86236515
OGFRL1	-0.599440106	2.502581322	-10.58803483	9.27E-24	42.83219568
RARRES2	-0.957315669	5.047998217	-10.57248763	1.06E-23	42.69801724
SMIM3	-0.733786753	3.4590603	-10.56525404	1.13E-23	42.63563096
RP11-733O18.1	-0.205022902	0.309516348	-10.56311307	1.15E-23	42.6171713
PLEKHA2	-0.554536096	2.794925677	-10.55185187	1.27E-23	42.52011495
PALD1	-0.587754277	2.128659765	-10.55148513	1.27E-23	42.51695526
TRAV2	-0.494832453	0.767952876	-10.53706461	1.44E-23	42.39276828
MMP19	-0.726741457	2.059004324	-10.53475928	1.47E-23	42.37292522
GNB4	-0.598526508	2.416967481	-10.52927965	1.54E-23	42.32577032

TMIGD2	-0.343054212	0.515183885	-10.52897905	1.55E-23	42.32318397
OR7E47P	-0.575683321	1.330211723	-10.5223474	1.64E-23	42.26613733
TBC1D2B	-0.47208663	2.685668625	-10.52194215	1.65E-23	42.26265198
CD1C	-0.985832375	1.875778559	-10.52176804	1.65E-23	42.26115465
CDH11	-0.853384439	2.955769327	-10.51832277	1.70E-23	42.23152782
TRBV24-1	-0.363770274	0.48089663	-10.50291505	1.94E-23	42.09910808
OSTF1	-0.368659928	5.089855437	-10.49247055	2.13E-23	42.00941428
COL6A2	-0.994651781	6.04857957	-10.49030006	2.17E-23	41.99078197
CYP27A1	-0.911328206	4.233873654	-10.48783409	2.21E-23	41.96961616
LINC01215	-0.414578522	0.528294259	-10.48750125	2.22E-23	41.96675954
RP11-383H13.1	-0.35338498	0.615026552	-10.483448	2.30E-23	41.93197737
RP11-598F7.3	-0.590279189	0.772059424	-10.47275478	2.52E-23	41.84025681
ISM1	-0.835746699	1.867314879	-10.46807386	2.63E-23	41.80012523
KIAA1755	-0.288499499	0.644166497	-10.45485135	2.94E-23	41.68682457
EVL	-0.564392308	2.931845073	-10.45482177	2.94E-23	41.68657119
ELK3	-0.62362088	4.024979303	-10.45068779	3.05E-23	41.65116691
HEG1	-0.709018574	2.922794281	-10.42016442	3.97E-23	41.39003528
MYO5A	-0.46259	1.874595467	-10.41423991	4.18E-23	41.3394069
ZNF683	-0.750322007	1.151346867	-10.40803846	4.41E-23	41.28643171
HTRA4	-0.551308423	0.862053321	-10.40771225	4.42E-23	41.28364569
TLR2	-0.911473134	3.313327429	-10.40581964	4.50E-23	41.26748259
GRAP	-0.240991833	0.545029454	-10.40287471	4.61E-23	41.24233645
TMEM236	-0.264488059	0.371418641	-10.4009198	4.69E-23	41.22564634
RP1-47M23.3	-0.232368529	0.258644212	-10.39937962	4.75E-23	41.21249845
AC092580.4	-0.875132316	1.908888359	-10.3991163	4.76E-23	41.21025072
ZBP1	-0.529081521	0.997902384	-10.3956089	4.91E-23	41.18031453
RP11-169D4.2	-0.266831584	0.48041574	-10.39504972	4.93E-23	41.17554248
FBLN2	-0.929463179	3.676892747	-10.39451207	4.96E-23	41.17095423
SYT11	-0.610184537	2.048170127	-10.39303774	5.02E-23	41.15837349
TSPAN2	-0.532212087	1.297889815	-10.38190357	5.52E-23	41.06339985
CHIT1	-1.431233368	2.472382587	-10.36633098	6.32E-23	40.93067654
GPR124	-0.601397413	2.216159711	-10.36424118	6.43E-23	40.91287522
SYNPO	-0.694377197	3.516278502	-10.36350895	6.47E-23	40.90663848

IL18RAP	-0.358184892	0.592957002	-10.36328037	6.48E-23	40.90469156
NLRC5	-0.657251915	2.294437445	-10.36163496	6.58E-23	40.89067814
PAPLN	-0.661296337	2.077955985	-10.35782332	6.80E-23	40.85822092
SP100	-0.384622185	2.850168895	-10.35697264	6.84E-23	40.85097821
VASH1	-0.541813038	2.21327307	-10.35692513	6.85E-23	40.85057376
JAM2	-0.473321925	1.428247543	-10.3554165	6.94E-23	40.83773017
KAT2B	-0.493222576	2.228353044	-10.35040137	7.24E-23	40.79504315
LRRN4CL	-0.346349156	0.528687887	-10.34103503	7.85E-23	40.7153557
NBL1	-0.835840016	4.117031463	-10.33825601	8.04E-23	40.69172115
CD8B	-0.711816048	1.401044883	-10.33142897	8.52E-23	40.63367708
LGALS9	-0.793063421	3.836702524	-10.31649797	9.69E-23	40.50681879
LMCD1	-0.485971723	2.040960573	-10.31520491	9.80E-23	40.49583816
RP11-473M20.9	-0.438229481	0.806905239	-10.31410071	9.89E-23	40.486462
LINC01358	-0.320119812	0.51846272	-10.30059141	1.11E-22	40.37180232
CTSW	-0.908970616	2.594991129	-10.29947803	1.12E-22	40.36235687
GAS1	-0.805688143	1.743311307	-10.29898995	1.13E-22	40.35821645
RGS9	-0.253555538	0.467614754	-10.29659349	1.15E-22	40.33788879
TRDC	-0.718669884	1.17494167	-10.28866287	1.23E-22	40.2706402
COL8A1	-0.956717804	3.686278323	-10.28592759	1.26E-22	40.24745381
TNFAIP8L3	-0.525994665	1.441041636	-10.28497552	1.27E-22	40.2393843
ITGBL1	-0.781805353	1.991470391	-10.28495018	1.27E-22	40.23916952
GMIP	-0.435267231	3.238242524	-10.28284251	1.29E-22	40.22130711
LINC00987	-0.267239319	0.611438403	-10.27540262	1.38E-22	40.15827334
MEOX2	-0.640882541	1.394161238	-10.2642261	1.52E-22	40.06363689
RP11-452C13.1	-0.243706403	0.370266996	-10.2529942	1.67E-22	39.96859894
MS4A2	-0.559792382	0.873267134	-10.24956451	1.72E-22	39.9395923
6-Sep	-0.533908572	3.019297112	-10.2430868	1.82E-22	39.8848243
NTAN1	-0.375381983	2.635640566	-10.24215015	1.83E-22	39.87690692
PTGIS	-0.847615818	2.21740902	-10.2371758	1.91E-22	39.83486735
CYGB	-0.554958647	2.566640305	-10.23685987	1.91E-22	39.83219782
IL32	-0.870207231	4.521082628	-10.22374536	2.14E-22	39.72142951
HLA-DQB2	-1.393790351	4.403555229	-10.22316005	2.15E-22	39.71648799
CCL17	-1.160794436	1.957701334	-10.21915764	2.23E-22	39.68270233

RP1-111C20.3	-0.2442211	0.303638663	-10.217037	2.27E-22	39.6648048
TRAV1-2	-0.350005749	0.539264326	-10.2144171	2.32E-22	39.64269704
TNFRSF8	-0.347633053	0.548186888	-10.20361679	2.54E-22	39.55159877
COX7A1	-0.580871141	2.430741864	-10.20127842	2.59E-22	39.53188342
HTRA1	-0.787425365	5.205335719	-10.19647479	2.70E-22	39.49139209
PODN	-0.834969898	3.067004567	-10.19647178	2.70E-22	39.49136674
RP11-67C2.2	-0.51182198	0.944652319	-10.1947031	2.74E-22	39.47646104
SPARC	-0.832816489	7.759217036	-10.19410921	2.76E-22	39.4714564
PIK3IP1	-0.622234312	3.834477021	-10.19039601	2.84E-22	39.44016992
LRP1	-0.751114655	4.120731289	-10.18915072	2.87E-22	39.42967912
MAP3K3	-0.441144629	2.829994812	-10.18823964	2.90E-22	39.42200437
SLIT3	-0.721120249	1.440486684	-10.1870733	2.93E-22	39.41217997
EGFL6	-0.714698018	2.602136353	-10.18343495	3.02E-22	39.3815379
CLEC17A	-0.325310534	0.378103284	-10.18228208	3.05E-22	39.37183001
TRAV24	-0.282104545	0.33070119	-10.17669598	3.20E-22	39.32480151
PLA2G15	-0.433266294	3.010256345	-10.17387892	3.27E-22	39.30109149
IRF5	-0.613998789	2.450689812	-10.17380946	3.28E-22	39.30050691
CCDC109B	-0.599595072	2.575358728	-10.17156969	3.34E-22	39.28165895
MAGI2-AS3	-0.388123851	0.973104131	-10.17076917	3.36E-22	39.27492306
CD19	-0.763841884	1.231242619	-10.16951487	3.40E-22	39.26436968
STAMBPL1	-0.363956026	1.132006403	-10.16828675	3.43E-22	39.25403734
PSAP	-0.473301085	9.19263223	-10.16505944	3.53E-22	39.22688947
MXRA5	-1.034353581	3.428264963	-10.15685833	3.78E-22	39.15792791
KIAA1462	-0.639381216	2.355126004	-10.1476669	4.09E-22	39.08068227
FN1	-1.074201936	7.042897367	-10.14678112	4.12E-22	39.07324054
LDB2	-0.523135506	1.827304376	-10.14192135	4.29E-22	39.03241946
ITGA8	-0.648331386	1.510523807	-10.1370645	4.47E-22	38.99163568
ZNF521	-0.513488991	1.188840003	-10.13674715	4.49E-22	38.9889713
ARL6IP5	-0.548115036	6.205993464	-10.1334021	4.62E-22	38.96089038
AP003774.1	-0.281607125	0.4262188	-10.13166617	4.68E-22	38.94632005
CD1B	-0.575888137	0.719886319	-10.13159283	4.69E-22	38.94570458
TMEM106A	-0.394767091	2.136660165	-10.11923688	5.20E-22	38.8420443
ST3GAL2	-0.381488707	1.917181095	-10.11865774	5.23E-22	38.83718759

CD68	-0.440166628	1.043745939	-10.11540714	5.38E-22	38.80993157
ENG	-0.645476517	5.111466408	-10.11382935	5.45E-22	38.7967039
TMEM229B	-0.373997266	0.912196106	-10.11086094	5.59E-22	38.77182156
DLEU7	-0.200896193	0.466758725	-10.10928293	5.66E-22	38.75859604
RGL1	-0.669223823	3.110616032	-10.0984168	6.21E-22	38.66756232
PDLIM3	-0.677492934	2.025985897	-10.09678122	6.29E-22	38.65386547
RGS19	-0.492051471	3.532267265	-10.09478047	6.40E-22	38.63711244
RP11-532F6.3	-0.270036725	0.463812224	-10.09467885	6.41E-22	38.6362616
LINC00528	-0.214748624	0.343939014	-10.0927124	6.51E-22	38.61979814
CCL22	-0.948455071	2.200410419	-10.0883628	6.76E-22	38.58338983
TRAV26-2	-0.33357599	0.389932494	-10.08728516	6.82E-22	38.5743711
NAAA	-0.496285843	2.471314154	-10.0867908	6.85E-22	38.57023398
CFP	-0.397591744	0.782986507	-10.08478735	6.97E-22	38.55346941
RNASE2	-0.653470737	1.39751023	-10.08190153	7.14E-22	38.52932502
IL7	-0.449389981	1.139294661	-10.06969317	7.91E-22	38.42723369
PRELP	-0.978642086	3.543454568	-10.06955652	7.92E-22	38.42609139
DACT3	-0.405985494	1.051010087	-10.06818165	8.01E-22	38.41459938
SLC2A5	-0.592275136	1.158304538	-10.0649491	8.24E-22	38.3875838
TPK1	-0.28286446	0.694756218	-10.05111503	9.26E-22	38.27203236
SLIT2	-0.608556992	1.614320478	-10.04169541	1.00E-21	38.19341345
GFPT2	-0.707993031	1.961153649	-10.03320946	1.08E-21	38.12262897
OLFML2B	-0.783353492	3.232014452	-10.02802266	1.12E-21	38.07938347
AC017002.1	-0.213411149	0.352034623	-10.02563101	1.15E-21	38.05944783
TTC24	-0.207053041	0.22625189	-10.02536557	1.15E-21	38.05723548
TBX5	-0.515875356	1.468039975	-10.02055721	1.20E-21	38.01716548
GPR137B	-0.586335547	3.23618047	-10.0190047	1.21E-21	38.00423057
SLC29A3	-0.461201585	2.753948651	-10.01461674	1.26E-21	37.9676788
RP11-472N13.3	-0.305444738	0.473920531	-10.01407437	1.26E-21	37.96316166
TAGLN	-0.856352933	5.343994063	-9.996249345	1.47E-21	37.81479373
CLEC6A	-0.256168463	0.258061083	-9.987513849	1.58E-21	37.74214729
IL1B	-0.70760342	1.623812121	-9.986118491	1.60E-21	37.73054707
FCRL6	-0.456392368	0.793039494	-9.985088826	1.61E-21	37.72198772
LDLRAD4	-0.402361325	0.983646714	-9.984164547	1.63E-21	37.7143049

KMO	-0.359268361	0.788136714	-9.983721624	1.63E-21	37.71062339
MRAS	-0.45444672	2.078922495	-9.956267298	2.05E-21	37.48263956
PRDM6	-0.343850548	0.734398002	-9.945776221	2.24E-21	37.39563094
CTD-2341M24.1	-0.275066865	0.463514264	-9.933051352	2.50E-21	37.29017831
RPL32P1	-0.421308709	0.578800416	-9.928294425	2.60E-21	37.2507802
TRDV1	-0.489991521	0.569955075	-9.927411489	2.62E-21	37.24346888
MMP9	-1.243139373	4.545456717	-9.927029782	2.62E-21	37.24030822
COL6A6	-0.518573584	0.711156906	-9.925850013	2.65E-21	37.23053984
HLA-DQB1-AS1	-0.909527711	2.076591812	-9.920837345	2.76E-21	37.18904408
FILIP1L	-0.748961368	3.034001091	-9.90922253	3.05E-21	37.09294843
UBD	-1.238472588	2.98474392	-9.90886747	3.05E-21	37.09001202
AEBP1	-0.970745187	5.927761914	-9.908724178	3.06E-21	37.08882699
SP110	-0.322258425	1.398958435	-9.903977312	3.18E-21	37.0495766
ADAMTS2	-0.734969294	2.313443343	-9.898237736	3.34E-21	37.00213461
CXorf65	-0.313398283	0.475323169	-9.896621692	3.38E-21	36.98878009
PDGFRB	-0.691039207	4.065279461	-9.896225534	3.40E-21	36.98550658
FBXL7	-0.498121872	1.41389012	-9.894081981	3.46E-21	36.96779561
PTK2B	-0.551716177	2.904520013	-9.893099754	3.48E-21	36.95968088
STK4	-0.372502319	2.701319784	-9.883844239	3.76E-21	36.88324239
RASL12	-0.596492645	2.308763801	-9.866567952	4.35E-21	36.74069116
TRBV13	-0.34010944	0.377986972	-9.861220768	4.55E-21	36.69660406
NUGGC	-0.412841457	0.645625027	-9.858957727	4.63E-21	36.6779503
DOCK4	-0.450215205	1.729257489	-9.847417402	5.10E-21	36.58287066
RP11-428G5.5	-0.283591837	0.30213319	-9.846405199	5.14E-21	36.57453478
AC069363.1	-0.291893519	0.361249664	-9.842369156	5.32E-21	36.54130218
OMD	-0.706389243	1.115672231	-9.842074406	5.33E-21	36.53887559
ENPP2	-0.710401756	3.05358689	-9.841193051	5.37E-21	36.53161991
GZMM	-0.764191222	1.757971417	-9.83369616	5.72E-21	36.46992014
LRRC32	-0.617970729	2.942024419	-9.833355673	5.73E-21	36.46711866
GAS6	-0.763652279	3.928617822	-9.830342813	5.88E-21	36.44233218
TSPAN32	-0.342210576	0.57860301	-9.808323254	7.06E-21	36.26133497
TGFBI	-1.052579075	4.762550696	-9.804897699	7.26E-21	36.23320207
TAP2	-0.600906877	3.010001049	-9.793096107	8.01E-21	36.13633047

FCRL1	-0.421032303	0.466394999	-9.792896563	8.02E-21	36.13469323
LIMS1	-0.383664328	3.36213155	-9.791224701	8.13E-21	36.12097656
CALD1	-0.683504742	4.113399254	-9.790741195	8.17E-21	36.11700996
HLA-DQA2	-1.505787634	4.421629564	-9.788045372	8.35E-21	36.09489635
RP11-24F11.2	-0.504320764	1.123386355	-9.781831847	8.79E-21	36.04394302
CTD-2353F22.1	-0.258832446	0.388683698	-9.779858738	8.94E-21	36.02776732
ITPRIP	-0.533657733	2.44595734	-9.76700006	9.94E-21	35.92240503
MRPS26	0.579064284	5.206117237	9.766667078	9.97E-21	35.91967786
LMOD1	-0.701608313	2.222607063	-9.762786471	1.03E-20	35.88789985
CD1E	-0.780633515	1.185143678	-9.756816328	1.08E-20	35.83902748
COL6A1	-0.905412631	5.576867672	-9.754796126	1.10E-20	35.82249444
PSMB10	-0.619820864	4.13055127	-9.743266206	1.21E-20	35.72817961
FYN	-0.576330114	2.705636824	-9.74209484	1.22E-20	35.71860206
APOBEC3H	-0.501307627	1.044582998	-9.741985019	1.22E-20	35.71770416
CDK14	-0.567717508	1.932900645	-9.735588289	1.29E-20	35.66541617
FMO1	-0.493390556	0.827303091	-9.733354817	1.31E-20	35.64716487
MAT2B	-0.415159296	4.02090607	-9.73170802	1.33E-20	35.63370952
APOBEC3D	-0.535609665	1.724399858	-9.72849565	1.37E-20	35.60746694
RGS10	-0.698718314	4.481471188	-9.703005193	1.69E-20	35.3994384
ACTA2	-0.846860306	5.680047981	-9.698907141	1.74E-20	35.36602868
ADARB1	-0.432243526	1.6313009	-9.696876798	1.77E-20	35.34947971
RP11-498E2.9	-0.259921236	0.427631942	-9.695994464	1.79E-20	35.34228868
SNX2	-0.346934617	4.031006856	-9.689145681	1.89E-20	35.28648628
LGALS2	-0.800782207	1.980073135	-9.6781177	2.07E-20	35.19668916
RP11-556E13.1	-0.253231001	0.382226317	-9.672921091	2.16E-20	35.1543991
CTC-251I16.1	-0.5755042	1.500102322	-9.672898196	2.16E-20	35.15421282
CACNA1C	-0.269110266	0.580916363	-9.66722229	2.26E-20	35.10804
BANK1	-0.542664407	1.546329584	-9.66483709	2.31E-20	35.08864221
CCDC69	-0.681908106	3.183533488	-9.657959209	2.44E-20	35.03272571
GXYLT2	-0.624832776	1.710354881	-9.65384112	2.53E-20	34.99925905
AC109642.1	-0.471499977	0.858737045	-9.649704446	2.61E-20	34.96565119
AP002954.4	-0.428926764	0.76263642	-9.648656636	2.64E-20	34.95713995
PDE6G	-0.461616084	0.941098269	-9.647387232	2.66E-20	34.94682958

IFI16	-0.819641317	4.350590397	-9.644411098	2.73E-20	34.92266043
CLEC4E	-0.605283204	1.161292452	-9.643990177	2.74E-20	34.91924255
TAP1	-0.7906859	5.088652111	-9.640321039	2.82E-20	34.88945342
HLX	-0.400463158	1.319373806	-9.638234984	2.87E-20	34.87252054
EMR4P	-0.298605209	0.472958689	-9.631915016	3.03E-20	34.82123555
RAB32	-0.637051903	4.109537974	-9.629324117	3.09E-20	34.80021769
SPRED1	-0.555217347	3.304143682	-9.628176915	3.12E-20	34.79091261
ZBED2	-0.665836328	1.324540782	-9.627064193	3.15E-20	34.78188793
RP11-405M12.4	-0.276913729	0.667506853	-9.615787088	3.45E-20	34.69046577
ST8SIA4	-0.691530207	2.333996435	-9.615401914	3.46E-20	34.6873445
TNFRSF4	-0.545037912	1.997515956	-9.613688912	3.51E-20	34.6734642
CYSLTR1	-0.539952296	1.129134075	-9.612801035	3.54E-20	34.66627048
LITAF	-0.447004888	5.503445286	-9.610583391	3.60E-20	34.64830477
LINC01480	-0.369715266	0.702002016	-9.607762079	3.69E-20	34.62545269
SGTB	-0.3319236	1.557420395	-9.589157601	4.29E-20	34.47487533
OVOL2	0.532270971	2.437149004	9.581949502	4.55E-20	34.41658968
HPS5	-0.344018739	2.500041841	-9.581605136	4.57E-20	34.41380584
COL14A1	-0.909523998	2.402671111	-9.577943834	4.71E-20	34.38421233
BGN	-0.809673366	7.739243305	-9.576231666	4.77E-20	34.37037592
HSPE1P18	-0.294096452	0.387314037	-9.568876553	5.07E-20	34.31095693
ZNF438	-0.260566385	1.860056067	-9.562653511	5.33E-20	34.26070803
HGF	-0.543849491	1.164598581	-9.557452228	5.56E-20	34.21872674
AC004988.1	-0.223403289	0.305594278	-9.551556935	5.84E-20	34.17116288
LY75	-0.472521259	1.42664543	-9.550435939	5.89E-20	34.16212086
ATP10D	-0.448003058	1.802894082	-9.549020369	5.96E-20	34.15070381
RBP5	-0.543105786	2.132902353	-9.546016901	6.11E-20	34.12648372
PALLD	-0.675562862	3.78977013	-9.540559549	6.38E-20	34.08248884
BOC	-0.338469263	0.699309025	-9.538602455	6.49E-20	34.06671579
IGKV1-17	-1.588020877	5.147790133	-9.531819946	6.86E-20	34.01206997
DKK2	-0.41315463	0.867001521	-9.531789257	6.86E-20	34.01182277
SNAI3	-0.37411504	1.196342465	-9.523892291	7.31E-20	33.94823185
SLC36A1	-0.40275497	1.763331748	-9.51622507	7.78E-20	33.88652578
LRRC15	-1.043456885	1.965662088	-9.515370012	7.84E-20	33.87964637

CPNE5	-0.630781482	1.611726786	-9.511943243	8.06E-20	33.85208043
TBX21	-0.532477776	0.844191471	-9.509402696	8.23E-20	33.83164795
TRGV2	-0.226673103	0.274436983	-9.505813477	8.47E-20	33.80278789
GZMB	-0.998261019	2.658756404	-9.498412179	9.00E-20	33.74329961
ERAP1	-0.529227849	3.46349488	-9.492373302	9.45E-20	33.69478561
APOE	-1.03873991	6.751579328	-9.491737995	9.50E-20	33.68968304
AQP9	-0.782023066	1.975056176	-9.491257302	9.54E-20	33.68582243
PRNP	-0.590247436	5.046080482	-9.486019792	9.95E-20	33.64376702
ARRB2	-0.415924779	3.767431215	-9.483426788	1.02E-19	33.62295203
TDO2	-0.500191564	0.956317379	-9.480434953	1.04E-19	33.59894038
MYD88	-0.391612025	4.255210777	-9.478013396	1.06E-19	33.57950947
TRBV7-4	-0.226488003	0.24126138	-9.476309151	1.08E-19	33.56583643
DACT1	-0.597786186	1.548777653	-9.471888204	1.12E-19	33.53037543
PRR33	-0.363759753	0.556471069	-9.467019241	1.16E-19	33.49133411
GPR171	-0.780101412	1.558159887	-9.466892164	1.16E-19	33.49031534
POPDC2	-0.242869886	0.779627065	-9.46238776	1.21E-19	33.45420994
AIM1	-0.803603564	2.801335119	-9.459566988	1.23E-19	33.4316059
UBASH3B	-0.48066825	1.235533717	-9.453935207	1.29E-19	33.38649002
MEI1	-0.542097226	1.166588043	-9.452302472	1.31E-19	33.37341377
ANTXR2	-0.555462581	2.802526629	-9.447143259	1.36E-19	33.33210495
TBC1D4	-0.460094303	2.217295231	-9.441754183	1.42E-19	33.28897238
FSTL1	-0.739238667	4.549691934	-9.440594805	1.44E-19	33.2796953
CNR2	-0.24561316	0.260493881	-9.43871135	1.46E-19	33.26462601
FRMD4A	-0.284778616	0.967635233	-9.429059373	1.58E-19	33.18743452
MS4A14	-0.343969565	0.630429786	-9.422813132	1.66E-19	33.13750962
FDCSP	-1.295568655	2.15193893	-9.420980433	1.68E-19	33.12286561
EHD4	-0.351718703	3.420642137	-9.414629253	1.77E-19	33.07213244
FMOD	-0.780759571	4.289692384	-9.414476678	1.78E-19	33.07091396
C7	-1.29290459	3.333778717	-9.409582237	1.85E-19	33.03183391
CYP2U1	-0.360514948	1.353617261	-9.407668725	1.88E-19	33.01655917
ADPRH	-0.421640829	2.065613613	-9.403905542	1.93E-19	32.98652562
LOX	-0.785091547	2.801959658	-9.402344556	1.96E-19	32.97407002
IGHV3-11	-1.576192098	5.612026365	-9.400217589	1.99E-19	32.9571006

IGKV1-16	-1.622625001	5.226345518	-9.40009757	1.99E-19	32.95614314
CCL8	-0.889733813	1.779238365	-9.398710586	2.02E-19	32.94507901
SYK	-0.554106651	3.668057635	-9.397835048	2.03E-19	32.93809533
ARL11	-0.432348273	1.186614678	-9.385774004	2.24E-19	32.84193727
APOC2	-0.229789205	0.288309972	-9.385679185	2.24E-19	32.84118166
RP11-367G6.3	-0.267547206	0.448843379	-9.384969545	2.25E-19	32.8355267
RARRES3	-1.066022628	5.58951916	-9.381280953	2.32E-19	32.80613794
GRB2	-0.30239842	5.32408021	-9.376555905	2.41E-19	32.76850304
MDGA1	-0.262124867	0.504562383	-9.363187061	2.69E-19	32.66209227
ADAMTSL4	-0.685387489	2.080117743	-9.352269506	2.93E-19	32.57527157
SEMA7A	-0.726437948	2.22885887	-9.347381224	3.05E-19	32.53642099
IGHV1-46	-1.541506584	5.135128196	-9.334773394	3.37E-19	32.43628347
MFGE8	-0.591306862	3.806780019	-9.330600038	3.49E-19	32.40315751
CLIC4	-0.538665509	5.263838215	-9.322793586	3.71E-19	32.34122177
MAN1A1	-0.667977772	3.7409453	-9.321663092	3.75E-19	32.33225554
SAMD9	-0.683632509	2.280171304	-9.319211693	3.82E-19	32.31281551
EMR1	-0.411186483	0.596545188	-9.314042203	3.98E-19	32.27183228
RAB30	-0.344580714	1.170024762	-9.308563405	4.16E-19	32.22841431
RAB31	-0.645371821	4.551032279	-9.303977689	4.32E-19	32.19208756
BLK	-0.507680895	0.67372006	-9.301503604	4.41E-19	32.17249378
ADAMDEC1	-1.003783943	2.030153491	-9.294107463	4.67E-19	32.11394109
MIR8071-2	-1.033268788	2.157624204	-9.290871231	4.80E-19	32.08833126
RP5-887A10.1	-0.481552951	0.627145263	-9.286089646	4.98E-19	32.0505038
TMEM86A	-0.450842583	1.794879523	-9.285097149	5.02E-19	32.0426538
FCHSD2	-0.365469055	3.227744795	-9.28002028	5.23E-19	32.00250834
SUSD6	-0.458669323	4.096146632	-9.276279871	5.39E-19	31.97294086
TIMP3	-0.641584956	1.599424562	-9.267768105	5.77E-19	31.90568765
TRAJ18	-0.338107627	0.33080972	-9.26581993	5.86E-19	31.89030084
HIC1	-0.341488567	1.08726563	-9.265242507	5.89E-19	31.88574075
ELTD1	-0.504038853	2.417425386	-9.262236882	6.03E-19	31.86200764
TXNIP	-0.784445734	7.393150241	-9.258208975	6.23E-19	31.83021087
ZC3H12D	-0.324373786	0.716105116	-9.248794818	6.72E-19	31.75593244
IGJ	-1.398818621	7.581180213	-9.245322451	6.90E-19	31.72854865

ZBTB32	-0.246032268	0.490899064	-9.245010368	6.92E-19	31.72608786
IGKV3-20	-1.545560476	8.219514906	-9.242985763	7.03E-19	31.71012514
PRR5L	-0.537862337	1.596697735	-9.233017927	7.62E-19	31.63157112
CILP	-0.975939632	1.655022014	-9.230176849	7.79E-19	31.60919225
TDRD6	-0.220458427	0.382465596	-9.220278709	8.43E-19	31.53126369
AMPD3	-0.409481979	2.121242974	-9.215909091	8.73E-19	31.49688028
RP5-839B4.8	-0.529479621	0.634632996	-9.213354869	8.91E-19	31.4767871
DEF6	-0.501751454	3.493148156	-9.201817803	9.76E-19	31.38607808
AC006369.2	-0.259899434	0.390615444	-9.20141245	9.79E-19	31.3828925
PPAPDC3	-0.220999334	0.537799517	-9.196816096	1.02E-18	31.34677766
TGFBR2	-0.629413127	5.115464015	-9.196286566	1.02E-18	31.34261783
ZFP36L2	-0.552632004	5.478852515	-9.194526248	1.03E-18	31.32879047
SIGLEC17P	-0.334376944	0.550428402	-9.19051129	1.07E-18	31.29725985
GPR25	-0.322070478	0.488994812	-9.190089348	1.07E-18	31.29394678
CLEC4D	-0.20835879	0.28417806	-9.186845513	1.10E-18	31.26847997
CRISPLD2	-0.62509675	2.867547709	-9.183409722	1.13E-18	31.2415131
PDE1B	-0.31389469	0.636763857	-9.179189431	1.17E-18	31.20839862
CD97	-0.626331933	4.405893729	-9.174137564	1.22E-18	31.16877339
SMAP2	-0.459522857	4.3605505	-9.173729905	1.22E-18	31.16557652
PRCP	-0.415403746	2.983249146	-9.172949121	1.23E-18	31.15945388
OR52K3P	-0.23885254	0.341107587	-9.171319067	1.24E-18	31.14667272
TMEM47	-0.601143855	2.347069502	-9.159243026	1.37E-18	31.05203552
FHL1	-0.822146552	2.327469277	-9.156192574	1.40E-18	31.02814382
KIAA1551	-0.530338937	3.054164052	-9.155124239	1.41E-18	31.01977776
C2orf15	0.404538549	1.599493651	9.153872582	1.43E-18	31.00997701
SOD2	-0.635737613	4.45038248	-9.151850245	1.45E-18	30.99414367
BST1	-0.508166841	1.550710225	-9.151787703	1.45E-18	30.99365405
LEF1	-0.459604428	1.513862101	-9.150458219	1.47E-18	30.98324661
VNN2	-0.651279157	1.500440978	-9.149887568	1.47E-18	30.97877979
KIRREL	-0.617440933	2.652272349	-9.149730066	1.48E-18	30.97754697
CD7	-0.748310548	2.321669531	-9.148840225	1.49E-18	30.97058213
STAC	-0.614137697	1.191693366	-9.144329382	1.54E-18	30.93528292
IGHV4-28	-1.255193032	3.391826377	-9.142879919	1.56E-18	30.9239429

IL17RA	-0.349535292	2.400904641	-9.140710182	1.59E-18	30.90697013
ANGPTL2	-0.756432645	3.71184311	-9.129928342	1.73E-18	30.82267172
CEP85L	-0.239394844	0.764088599	-9.129683405	1.73E-18	30.82075748
PDLIM2	-0.36877322	1.461225491	-9.126813252	1.77E-18	30.7983294
MDFIC	-0.606360747	3.432403727	-9.122712459	1.83E-18	30.7662935
MTA3	0.323753986	2.395952033	9.121752885	1.84E-18	30.75879867
RP11-162J8.3	-0.374032957	0.538080834	-9.120816563	1.86E-18	30.75148599
MOB3A	-0.406046592	3.839683311	-9.117571897	1.90E-18	30.72614928
CD82	-0.675601381	4.135838525	-9.117464562	1.91E-18	30.72531124
HLA-DPA3	-0.49570117	0.780502065	-9.115837649	1.93E-18	30.71260965
RAP1A	-0.336661194	3.889413949	-9.114382492	1.95E-18	30.70125035
VGLL3	-0.57618045	1.345328568	-9.111692138	1.99E-18	30.68025222
RP11-10J5.1	-0.266990405	0.302771023	-9.110364422	2.02E-18	30.66989107
OSM	-0.718091873	1.688218966	-9.105425494	2.10E-18	30.6313584
WDFY4	-0.594409742	1.426172975	-9.10482509	2.11E-18	30.62667516
HLA-A	-0.687638845	9.054537051	-9.104263287	2.11E-18	30.62229322
MMP25	-0.373412484	0.886238979	-9.09466145	2.28E-18	30.54743083
ATP6V0D2	-0.543404802	0.935117018	-9.089180034	2.38E-18	30.50471929
LGALS1	-0.812992718	7.414032437	-9.067857859	2.82E-18	30.33875057
MYL9	-0.688604909	5.629867171	-9.066568087	2.85E-18	30.32872011
CXCR2P1	-0.533331083	0.711078187	-9.066011546	2.86E-18	30.32439225
XAF1	-0.552944902	1.468228656	-9.062232642	2.95E-18	30.29501118
GPR155	-0.327606728	1.187921416	-9.060626246	2.98E-18	30.28252406
PLN	-0.750919287	1.961324669	-9.055779332	3.10E-18	30.24485678
ICAM2	-0.463619488	2.106937473	-9.054722651	3.12E-18	30.23664681
IGLV3-25	-1.585882326	6.76882315	-9.053984824	3.14E-18	30.23091461
RP11-354E11.2	-0.201596112	0.320268149	-9.052637475	3.18E-18	30.22044789
SLC2A6	-0.51812476	1.851175963	-9.051666566	3.20E-18	30.21290618
PIEZO2	-0.386607481	0.798618231	-9.046765884	3.33E-18	30.17484811
RILPL2	-0.394644961	2.322696159	-9.046379615	3.34E-18	30.17184902
IL34	-0.531142338	1.381251805	-9.044015626	3.40E-18	30.15349642
IQGAP2	-0.624820558	1.778291889	-9.040415961	3.50E-18	30.12555738
CALCRL	-0.609169597	2.409375228	-9.038772805	3.54E-18	30.11280656

SULF2	-0.721354597	2.985754156	-9.037981841	3.56E-18	30.10666931
COL3A1	-1.095973057	7.763295993	-9.036461592	3.61E-18	30.09487447
IFIT3	-0.758320952	4.03945133	-9.034906056	3.65E-18	30.08280732
C2CD4D	0.471738041	0.989936511	9.028580724	3.84E-18	30.03375361
LAYN	-0.580463365	1.85334928	-9.028518255	3.84E-18	30.03326928
RP11-1070N10.3	-0.272334677	0.416461428	-9.022433255	4.03E-18	29.98610286
IGHV4-55	-1.096587697	2.700655197	-9.021869382	4.04E-18	29.98173329
ROR2	-0.499243011	1.256701151	-9.01411587	4.30E-18	29.92166957
CPQ	-0.589940155	3.241924195	-9.014019258	4.30E-18	29.92092138
HSPB2	-0.240967772	0.58269042	-9.007025604	4.54E-18	29.86677625
STAT1	-0.694226856	5.791779806	-9.001034959	4.76E-18	29.82042046
ST6GALNAC5	-0.554322398	1.272279918	-8.997799791	4.88E-18	29.79539586
MGAT1	-0.351007168	5.042170445	-8.996121876	4.95E-18	29.78241943
TMOD2	-0.307511584	0.977298041	-8.995549049	4.97E-18	29.77798978
4-Sep	-0.311958024	1.059351209	-8.991400675	5.13E-18	29.7459166
RP11-290F5.1	-0.492382789	0.828216223	-8.989872763	5.20E-18	29.73410622
MYO1G	-0.857839071	2.258768667	-8.986452145	5.34E-18	29.70767093
ADRB2	-0.523242803	1.219024521	-8.978055193	5.70E-18	29.64280814
TRIM21	-0.425598373	3.909086832	-8.977303327	5.73E-18	29.63700244
HDC	-0.437016269	0.727942159	-8.975338366	5.82E-18	29.62183119
GSTP1	0.625801141	8.373886316	8.971702293	5.99E-18	29.59376378
TEK	-0.530287362	1.551339261	-8.969939952	6.07E-18	29.58016294
CCL21	-1.110580746	5.03131305	-8.969630689	6.09E-18	29.57777641
DNM3OS	-0.310338788	0.681165152	-8.966235288	6.25E-18	29.55157855
CADM3	-0.499183482	0.746160435	-8.961766611	6.47E-18	29.51711051
IGKV4-1	-1.598674677	7.87172045	-8.961596127	6.48E-18	29.51579577
IGLV1-44	-1.495894012	6.539242101	-8.958416741	6.64E-18	29.49128024
ANTXR1	-0.800582409	4.085353289	-8.956452804	6.75E-18	29.4761399
SCN7A	-0.642714516	1.327527865	-8.954919135	6.83E-18	29.46431824
ZFPM2	-0.315894702	0.763060055	-8.954535309	6.85E-18	29.4613599
C10orf10	-0.733699229	5.022904015	-8.954098732	6.87E-18	29.45799509
TMEM255A	-0.453601224	0.812340457	-8.954095563	6.87E-18	29.45797067
PPP3CC	-0.319016151	2.501584561	-8.952815058	6.94E-18	29.44810219

EDNRA	-0.649157749	2.681044947	-8.951525346	7.01E-18	29.43816378
CDH23	-0.212544059	0.373043247	-8.95070134	7.06E-18	29.43181461
IFI30	-0.356369178	0.978602647	-8.94969774	7.11E-18	29.42408219
FLVCR2	-0.493709894	2.02345882	-8.946517792	7.29E-18	29.39958582
LAG3	-0.699096463	1.70663399	-8.944179201	7.43E-18	29.38157476
ARPC2	-0.296613398	5.382263896	-8.942733204	7.51E-18	29.37043987
IGHV1-67	-0.951237404	1.728828859	-8.941211369	7.60E-18	29.35872238
MAP3K14	-0.467919015	2.117623198	-8.939141	7.72E-18	29.34278374
RP11-126O1.6	-0.289364158	0.38280867	-8.937305026	7.83E-18	29.32865181
TNFAIP6	-0.680074078	1.95281013	-8.935730449	7.93E-18	29.31653357
CFH	-0.808838173	3.532546525	-8.935187983	7.96E-18	29.31235902
TPSB2	-1.053252718	2.917267626	-8.934449836	8.01E-18	29.30667888
IDO1	-1.139792779	3.094565092	-8.931015863	8.23E-18	29.28025849
KCNA3	-0.614752385	1.264366816	-8.923976209	8.69E-18	29.22611957
CETP	-0.288856494	0.84153291	-8.919849302	8.98E-18	29.19439567
RELT	-0.381932192	1.548371073	-8.919005946	9.03E-18	29.18791402
HECA	-0.402376768	2.898309552	-8.90686858	9.93E-18	29.09468092
IGHV4-61	-1.301843897	3.020970075	-8.904990815	1.01E-17	29.08026509
SLFN5	-0.581960546	3.463245578	-8.902017883	1.03E-17	29.05744603
ANGPTL1	-0.349751859	0.640000318	-8.8938589	1.10E-17	28.99484923
DDO	-0.485410525	1.090841812	-8.892830704	1.11E-17	28.98696372
LOXL1	-0.644422017	2.975190256	-8.890262763	1.13E-17	28.96727239
AL928768.3	-0.7146812	1.266215258	-8.889181387	1.14E-17	28.95898148
ZNF423	-0.227992978	0.466068326	-8.888733452	1.14E-17	28.95554737
IL22RA2	-0.420251947	0.516694315	-8.887790045	1.15E-17	28.94831514
REM1	-0.309577971	0.77436894	-8.885985689	1.17E-17	28.93448434
PCDHGB7	-0.302094021	0.733183793	-8.881166985	1.21E-17	28.89755786
TNFRSF17	-0.830900181	1.790825672	-8.879293497	1.23E-17	28.88320495
THBS2	-1.032602891	4.558041863	-8.877613138	1.25E-17	28.87033349
MRC2	-0.701088065	4.136101846	-8.876671684	1.26E-17	28.86312277
TPST2	-0.333998989	2.681371933	-8.875619369	1.27E-17	28.8550636
MS4A4E	-0.233345313	0.380137744	-8.875067031	1.27E-17	28.8508338
IGFLR1	-0.363137602	1.25792622	-8.87464187	1.28E-17	28.84757805

ADAM8	-0.803220753	3.637088646	-8.874175395	1.28E-17	28.84400605
MYCT1	-0.453801109	1.756216244	-8.8719503	1.30E-17	28.82696944
STARD13	-0.345359849	1.340263373	-8.871596336	1.31E-17	28.82425958
XCR1	-0.274215214	0.426071933	-8.870850007	1.31E-17	28.81854611
EGR3	-0.568904723	1.296852672	-8.866247991	1.36E-17	28.78332344
TCEAL7	-0.395644403	1.044050727	-8.865948058	1.36E-17	28.78102829
HEPH	-0.565427947	1.70698783	-8.863769907	1.39E-17	28.7643623
IGLV5-45	-1.406606761	3.43564786	-8.861464237	1.41E-17	28.74672386
IGDCC4	-0.275820857	0.547714856	-8.860865816	1.42E-17	28.74214646
SELP	-0.562686719	1.511595896	-8.856914656	1.46E-17	28.7119292
VPREB3	-0.772971029	1.646633424	-8.853739886	1.50E-17	28.68765663
IFI27L2	-0.62299723	3.348670308	-8.847676241	1.57E-17	28.64131485
THY1	-0.742491704	3.782556216	-8.844780597	1.61E-17	28.61919287
PCDHGC3	-0.53197022	1.766181665	-8.840401702	1.66E-17	28.58574922
NFATC1	-0.365420299	1.486971653	-8.836562253	1.71E-17	28.55643548
BHLHE40-AS1	-0.236997808	0.724257919	-8.830815187	1.79E-17	28.51257463
UBA7	-0.576291013	3.715606243	-8.82574933	1.86E-17	28.47392991
TRBV10-2	-0.251749024	0.259266697	-8.822375902	1.91E-17	28.44820478
FPR2	-0.409383739	0.693017429	-8.820099634	1.94E-17	28.43085044
POU2AF1	-0.799068854	1.843487774	-8.815357903	2.02E-17	28.3947098
CHRDL1	-0.926629745	2.059210948	-8.815255258	2.02E-17	28.39392762
NAP1L3	-0.350022477	0.779791389	-8.814785502	2.03E-17	28.39034802
CTB-133G6.1	-0.259194767	0.439032731	-8.81323975	2.05E-17	28.37857019
IGHV1OR15-9	-1.039498341	1.869621663	-8.812325313	2.06E-17	28.37160336
IFNG	-0.498484967	0.64945375	-8.811082893	2.08E-17	28.36213857
CX3CR1	-0.561742824	1.061182075	-8.805088409	2.18E-17	28.31648609
OAF	-0.538108098	2.610019936	-8.804814785	2.19E-17	28.31440278
IGKV1-8	-1.217551519	2.696335054	-8.804171926	2.20E-17	28.30950838
IFIT2	-0.648001986	2.735900001	-8.803868723	2.20E-17	28.30720004
MIR4420	-0.360383252	0.470141568	-8.803512621	2.21E-17	28.30448905
INHBA	-0.789822205	2.572738481	-8.802573968	2.23E-17	28.29734349
CORO1C	-0.453936185	4.396154248	-8.799233132	2.28E-17	28.27191566
RN7SL138P	-0.455129231	1.333916632	-8.798878183	2.29E-17	28.26921448

CLEC11A	-0.582791903	2.957706488	-8.798503176	2.30E-17	28.26636075
WISP2	-0.583010452	1.487883947	-8.798062722	2.31E-17	28.26300908
LBH	-0.628210044	4.882661548	-8.787506846	2.50E-17	28.18272011
MSL3	-0.269388403	2.489590219	-8.783140011	2.59E-17	28.14952614
IGLV2-18	-1.319626528	3.131092959	-8.781717767	2.61E-17	28.13871773
IGHV3-15	-1.47080913	6.131669183	-8.779594138	2.66E-17	28.12258148
CD70	-0.452757885	0.720067714	-8.778734579	2.68E-17	28.116051
CMTM3	-0.63354878	4.105859201	-8.776986737	2.71E-17	28.10277324
C6orf136	0.400168861	2.99351946	8.775043534	2.75E-17	28.08801365
PNRC1	-0.356369742	4.994753961	-8.768622674	2.89E-17	28.03926103
TCF21	-0.560190327	1.043550589	-8.761642684	3.05E-17	27.98629264
TIMD4	-0.35255406	0.542346034	-8.759500183	3.10E-17	27.97004023
CYYR1	-0.469737751	2.134276368	-8.749326755	3.35E-17	27.8929072
ABCA1	-0.547748346	2.46034595	-8.748270087	3.38E-17	27.8848995
EMR2	-0.467300225	1.144070321	-8.745795428	3.45E-17	27.86614868
RP11-325F22.2	-0.349140356	0.640079468	-8.741212291	3.57E-17	27.8314319
RP11-147L13.15	-0.334721783	1.158179054	-8.738601719	3.64E-17	27.81166306
HAAO	-0.378270783	1.41021256	-8.73754813	3.67E-17	27.80368587
DKK3	-0.724346916	3.781646654	-8.737276968	3.68E-17	27.80163289
TUBB6	-0.651264045	3.496517526	-8.73557108	3.73E-17	27.78871864
LINC00968	-0.217459215	0.295092179	-8.734659868	3.75E-17	27.78182116
IGHV3-74	-1.398523877	5.157605044	-8.731837693	3.83E-17	27.76046186
CAPZB	-0.290593582	6.198047285	-8.731770832	3.84E-17	27.75995589
FCRL5	-0.555667466	0.969651241	-8.730930062	3.86E-17	27.75359364
IRF2	-0.285341594	3.912772461	-8.729687278	3.90E-17	27.7441901
GRTP1	0.514167189	2.420469478	8.726860891	3.98E-17	27.72280785
IGKJ5	-1.28337377	3.17825593	-8.726771346	3.99E-17	27.72213051
TRBV5-5	-0.266261928	0.307700138	-8.719462207	4.22E-17	27.66685924
PDE5A	-0.402911717	1.499547199	-8.719408012	4.22E-17	27.66644955
KIAA0040	-0.426962164	3.403113069	-8.715406421	4.35E-17	27.63620429
NLRP1	-0.537216603	1.691184755	-8.711873218	4.47E-17	27.60950773
ADAM12	-0.740926145	1.642979554	-8.710033277	4.53E-17	27.59560845
IFNAR2	-0.435042032	2.929156363	-8.709830533	4.54E-17	27.59407701

AC093818.1	-0.252182276	0.692053607	-8.700131755	4.89E-17	27.5208474
CCL11	-0.707179806	1.331189513	-8.69776389	4.98E-17	27.5029782
FAM65B	-0.563273508	1.484986844	-8.696342184	5.03E-17	27.49225094
SERPINF1	-0.856582243	5.195845698	-8.696266102	5.04E-17	27.49167691
IGHV3-52	-0.75009414	1.271567083	-8.692773608	5.17E-17	27.46533049
AKAP7	-0.300650195	1.113015131	-8.691503743	5.22E-17	27.45575291
NOL7	0.284021809	4.788971573	8.683576168	5.55E-17	27.39598477
IGHV3-30	-1.426106075	5.999895606	-8.677633102	5.81E-17	27.35120471
NELFA	0.325625132	2.915563592	8.677206459	5.82E-17	27.34799089
IFIH1	-0.570119834	3.102662118	-8.67709528	5.83E-17	27.34715342
LHFP	-0.533256766	3.580310053	-8.671548599	6.08E-17	27.30538232
IGHV3-71	-0.830184119	1.501470299	-8.67104681	6.10E-17	27.3016044
DYSF	-0.559761374	2.380877404	-8.6697992	6.16E-17	27.29221197
IGHV2-5	-1.39940328	3.681121009	-8.668779163	6.21E-17	27.28453354
IGHGP	-1.356297614	5.359939905	-8.661141116	6.58E-17	27.22705845
RASGRP1	-0.541473193	1.77357556	-8.657323003	6.78E-17	27.19834175
RP11-812E19.9	-0.812232546	1.292474733	-8.657108624	6.79E-17	27.19672964
TOX2	-0.525417918	1.548206465	-8.656189271	6.84E-17	27.18981655
SLFN11	-0.655970384	2.963238325	-8.655512387	6.87E-17	27.18472706
SNORA14	-0.239278977	0.283003725	-8.652544401	7.03E-17	27.16241421
GGT5	-0.638731888	2.863401984	-8.651786704	7.07E-17	27.15671886
ERG	-0.411251046	1.563374511	-8.649354937	7.20E-17	27.1384426
IGLV4-69	-1.525525771	5.345871711	-8.648507077	7.25E-17	27.1320713
PARP15	-0.530330082	1.178306534	-8.641244836	7.66E-17	27.07751751
FCER2	-0.415944102	0.460198498	-8.640073851	7.73E-17	27.06872426
EPS15	-0.26017587	3.475551189	-8.638912319	7.80E-17	27.06000287
KLF12	-0.335568721	0.978393599	-8.6371487	7.90E-17	27.04676234
GPR114	-0.428311972	0.870344228	-8.63091107	8.29E-17	26.99994881
THBS1	-0.881124764	4.945349731	-8.629270673	8.39E-17	26.98764175
SPNS3	-0.278430817	0.597617812	-8.618671751	9.09E-17	26.9081651
IGHV5-51	-1.517377096	6.940633771	-8.617373628	9.19E-17	26.89843601
IGKV1-6	-1.405032723	4.876661535	-8.615701484	9.30E-17	26.88590533
SH3BGRL3	-0.487410034	6.834566731	-8.614311092	9.40E-17	26.8754874

TSPAN18	-0.507466047	1.805689228	-8.608188282	9.85E-17	26.82962522
JAK1	-0.396366378	4.790311635	-8.607324815	9.91E-17	26.82315946
IGHJ3	-1.3688047	4.687091322	-8.60448502	1.01E-16	26.80189808
COL16A1	-0.630977904	2.597346976	-8.598118242	1.06E-16	26.75424925
RAB3IL1	-0.481835284	2.13293801	-8.59097691	1.12E-16	26.70083475
IGHG2	-1.409363581	8.358242596	-8.585875594	1.17E-16	26.66269894
WBP1L	-0.291530781	4.232170892	-8.582207885	1.20E-16	26.63529069
BATF2	-0.645360858	2.510891835	-8.58188096	1.20E-16	26.63284805
C1QTNF7	-0.385132002	0.623440732	-8.573883305	1.28E-16	26.57311453
IGHV3-49	-1.492464695	4.997599723	-8.573485952	1.28E-16	26.57014783
TNFRSF13B	-0.350097494	0.469742739	-8.570380262	1.31E-16	26.54696374
LACC1	-0.336012521	1.564885774	-8.568824485	1.33E-16	26.53535215
PTPLAD2	-0.403027054	1.589863564	-8.568783622	1.33E-16	26.53504719
ZNF671	-0.384108281	1.445668683	-8.567520706	1.34E-16	26.52562256
STK17A	-0.527556834	3.516856833	-8.563135855	1.39E-16	26.49290819
TMEM204	-0.505076499	3.108937485	-8.561551165	1.40E-16	26.48108824
SIGLEC6	-0.322106813	0.441016225	-8.559020343	1.43E-16	26.46221461
LINC01010	-0.208244107	0.253815796	-8.558445087	1.44E-16	26.45792521
DNASE2B	-0.341486735	0.430380479	-8.547591788	1.56E-16	26.37703766
SFXN3	-0.521866485	3.809318287	-8.547028226	1.56E-16	26.37283962
IGLV7-43	-1.342481187	3.896739445	-8.546998473	1.57E-16	26.37261799
PLCB2	-0.593968269	2.332803177	-8.538233228	1.67E-16	26.30735133
IGKV1-5	-1.476302343	7.513218889	-8.537602908	1.68E-16	26.30265985
TRBJ2-2	-0.508122434	0.756247127	-8.533058299	1.74E-16	26.26884181
MMRN1	-0.542894446	1.202834488	-8.531830619	1.75E-16	26.25970851
CYBRD1	-0.779938713	4.402023262	-8.531767684	1.76E-16	26.25924033
CYB561A3	-0.270635732	2.223114169	-8.525423538	1.84E-16	26.21205901
FXYD5	-0.700604563	4.984268702	-8.524425838	1.86E-16	26.20464151
IGLV3-9	-1.476325141	4.1060755	-8.522276111	1.89E-16	26.18866135
FAT4	-0.388734277	0.895779932	-8.52185071	1.89E-16	26.18549945
NFKB1	-0.361906139	3.594661121	-8.518287074	1.94E-16	26.15901646
IGHV1-58	-1.344491618	2.931870102	-8.512959123	2.02E-16	26.11943744
RASGRF2	-0.333979987	0.916629086	-8.507463125	2.11E-16	26.0786294

CASP4	-0.454897466	3.595475875	-8.506908371	2.12E-16	26.07451141
CYR61	-0.845334259	5.863852162	-8.50623434	2.13E-16	26.06950829
MZB1	-1.116506422	3.710095074	-8.501896005	2.20E-16	26.03731328
LIMD2	-0.585461972	2.803550745	-8.501070363	2.21E-16	26.03118754
NAGK	-0.295286565	3.007274567	-8.494190195	2.33E-16	25.98015826
SDC3	-0.553080316	4.06361091	-8.485921984	2.48E-16	25.91887482
GHRL	-0.232903359	0.490719375	-8.485273389	2.49E-16	25.91406936
MYO9B	-0.406401069	3.47417102	-8.481692819	2.56E-16	25.88754574
IGLV3-19	-1.55111812	7.032499527	-8.480701178	2.58E-16	25.88020149
NFKBIE	-0.451035324	3.498236953	-8.477236666	2.65E-16	25.85454778
TRAJ3	-0.401240029	0.467508797	-8.474866844	2.69E-16	25.83700444
RNF166	-0.38568615	2.568328558	-8.470773257	2.78E-16	25.80670903
SH2D2A	-0.503026842	1.858262096	-8.466441596	2.87E-16	25.77466361
COL1A2	-0.954603526	6.942505468	-8.465782094	2.88E-16	25.76978573
CAMK1	-0.329102843	1.588827607	-8.461440963	2.98E-16	25.73768441
GPT2	0.821899781	3.218392666	8.460770207	2.99E-16	25.73272547
IGHV3OR16-9	-0.83615437	1.523796634	-8.456668682	3.09E-16	25.70240907
MAP3K7CL	-0.304559298	1.186018208	-8.455422537	3.12E-16	25.69320037
IGKV3D-20	-1.270617203	3.707965022	-8.45454116	3.14E-16	25.68668783
FLNA	-0.722923509	6.533133879	-8.453599747	3.16E-16	25.67973224
MICB	-0.557636025	2.197576038	-8.452921223	3.18E-16	25.67471935
CLCN2	0.35056618	1.568709022	8.449522066	3.26E-16	25.64961115
FAM129C	-0.277046132	0.317877553	-8.448063546	3.29E-16	25.63883997
CTD-2547L24.3	-0.314639621	0.654068224	-8.447125381	3.32E-16	25.63191235
IGHV3OR16-13	-0.853726258	1.525750874	-8.442906696	3.42E-16	25.60076775
ABCB6	0.479030311	1.419438463	8.441402798	3.46E-16	25.58966798
CCDC102B	-0.312771206	1.130530493	-8.437977558	3.55E-16	25.56439296
IGHV3-23	-1.366143511	7.293754733	-8.437813303	3.56E-16	25.5631811
SLC16A2	-0.562080472	2.100434364	-8.433354806	3.68E-16	25.53029361
SFRP4	-1.055150834	3.702936113	-8.431831248	3.72E-16	25.51905828
MARCKS	-0.557986406	5.167741647	-8.429104588	3.79E-16	25.49895458
LPPR4	-0.320722894	0.643803209	-8.427958604	3.83E-16	25.49050667
STK17B	-0.465188982	3.796397358	-8.421025604	4.03E-16	25.43941672

IGKV1D-8	-1.030965704	2.2155764	-8.420197437	4.06E-16	25.43331599
PML	-0.446299209	3.121560256	-8.418609056	4.10E-16	25.42161635
PRICKLE1	-0.331130783	0.839084231	-8.416870657	4.16E-16	25.40881362
CSTA	-0.768761895	2.436526355	-8.415677003	4.20E-16	25.40002389
ST8SIA1	-0.217640128	0.384124052	-8.415383616	4.20E-16	25.39786362
RP11-493L12.4	-0.250265662	0.497319582	-8.414572816	4.23E-16	25.39189382
SGIP1	-0.218337325	0.526450034	-8.414278581	4.24E-16	25.38972752
CSDC2	-0.423120922	1.166155692	-8.412698594	4.29E-16	25.37809584
SGCA	-0.540083354	1.276501098	-8.411196668	4.34E-16	25.36704036
NAGA	-0.33160728	4.236032988	-8.410207823	4.37E-16	25.35976241
OAS2	-0.744225844	3.521826936	-8.406464595	4.49E-16	25.33221787
RP4-753P9.3	-0.276888907	0.656045004	-8.405070073	4.54E-16	25.32195864
IGKV3OR2-268	-0.950058205	1.945054104	-8.403191655	4.60E-16	25.30814148
MGP	-0.868337024	5.952212771	-8.403092404	4.61E-16	25.30741148
IGLV2-14	-1.438571603	7.376467461	-8.403041234	4.61E-16	25.30703512
NELL2	-0.483407563	0.955068344	-8.402110849	4.64E-16	25.30019241
TRIM14	-0.463760023	3.349234894	-8.398898205	4.75E-16	25.2765687
IGLV3-21	-1.52900637	6.938294511	-8.39838434	4.77E-16	25.2727907
OGN	-0.664159674	1.076054391	-8.393948095	4.93E-16	25.24018206
MSN	-0.557198479	6.65509101	-8.38888173	5.12E-16	25.20295755
IFNG-AS1	-0.234986914	0.269211396	-8.388072685	5.15E-16	25.19701476
GNG8	-0.289646624	0.570038459	-8.387605342	5.17E-16	25.19358211
SYDE1	-0.443829094	1.977474895	-8.387272963	5.18E-16	25.19114086
IGHV3-53	-1.242672052	3.817034788	-8.384407703	5.30E-16	25.17009922
RP11-1166P10.8	-0.773451766	1.417071545	-8.383530335	5.33E-16	25.16365716
LRCH1	-0.328440531	1.917515515	-8.381119744	5.43E-16	25.14596005
IGKV3-11	-1.361963814	7.398602328	-8.380563608	5.45E-16	25.14187777
FAM180A	-0.309097946	0.50472794	-8.379383891	5.50E-16	25.13321883
POSTN	-0.938125724	5.170943028	-8.377779557	5.56E-16	25.12144473
RP5-1171I10.5	-0.564327249	1.05879529	-8.371041871	5.85E-16	25.0720158
PKD2	-0.432065937	2.720347509	-8.363719021	6.18E-16	25.01832792
IGHV1-18	-1.474356417	6.698011097	-8.362467122	6.24E-16	25.0091531
CHI3L1	-1.189857097	4.75611068	-8.361745016	6.27E-16	25.00386145

SNRK	-0.304969114	2.626466489	-8.360469502	6.33E-16	24.99451522
CXorf36	-0.386865145	1.468766938	-8.360078149	6.35E-16	24.99164783
ARRDC4	-0.55734032	2.587174287	-8.358185589	6.44E-16	24.97778272
STON1	-0.306253854	0.993318617	-8.358114467	6.44E-16	24.97726172
PNOC	-0.43690411	0.783830586	-8.356142204	6.54E-16	24.9628153
KIF17	-0.220587068	0.524520653	-8.351795414	6.75E-16	24.93098503
APOL2	-0.513067275	4.012773943	-8.345480722	7.07E-16	24.88476661
AC093850.2	-0.762479466	1.553824472	-8.343979061	7.15E-16	24.87377955
BLNK	-0.454611356	1.964393013	-8.342861327	7.21E-16	24.8656025
TIE1	-0.483328978	2.168745449	-8.339274296	7.41E-16	24.83936628
SEMA4D	-0.373025055	2.213151206	-8.329656927	7.95E-16	24.76906503
OPTN	-0.453191924	4.021046773	-8.325999737	8.17E-16	24.74234768
RP11-291B21.2	-0.664544023	1.005106752	-8.325938952	8.18E-16	24.7419037
IGKV1OR2-6	-0.950433379	1.631007386	-8.315211465	8.85E-16	24.6635863
KLRK1	-0.204680977	0.281212963	-8.31491115	8.87E-16	24.66139491
ECSCR	-0.410536731	1.820942135	-8.31457392	8.89E-16	24.65893422
CTC-231O11.1	-0.438762043	0.742242418	-8.31251243	9.03E-16	24.64389365
PLAU	-1.096301873	5.224925673	-8.304737203	9.57E-16	24.58719118
KIAA0125	-0.397545761	0.610643098	-8.300670325	9.86E-16	24.55754859
ESRP1	0.473195772	4.608076534	8.300549918	9.87E-16	24.55667113
RP11-284N8.3	-0.742260844	1.785661742	-8.295766962	1.02E-15	24.52182363
HAS2	-0.538651164	1.259289852	-8.293529234	1.04E-15	24.50552528
RP11-701P16.5	-0.294663606	0.482641536	-8.292927194	1.04E-15	24.50114093
TRIM69	-0.404636854	2.245347895	-8.291963823	1.05E-15	24.49412568
IGHV3-19	-0.776099518	1.440938506	-8.291556281	1.05E-15	24.49115816
UBE2E2	-0.448122863	2.768413082	-8.289648228	1.07E-15	24.4772661
ADAMTS12	-0.605479818	1.727622255	-8.287993871	1.08E-15	24.4652231
PEAK1	-0.324522756	1.476397178	-8.284252816	1.11E-15	24.43799655
FAM101B	-0.463906011	2.472448032	-8.281799396	1.13E-15	24.42014616
GPBAR1	-0.211545404	0.448479227	-8.281637367	1.13E-15	24.41896742
TMEM51	-0.488138839	3.686530539	-8.281185161	1.14E-15	24.41567778
IGLV2-8	-1.377190577	5.073618769	-8.279287978	1.15E-15	24.40187794
IGHV1-69-2	-1.523373526	5.411171316	-8.277934855	1.17E-15	24.39203697

DERL3	-0.813677608	3.229290976	-8.277056943	1.17E-15	24.38565276
IGLV1-40	-1.380703991	7.35433828	-8.275820188	1.18E-15	24.3766599
IGLV2-23	-1.418137043	6.910224757	-8.274372264	1.20E-15	24.36613285
AC116366.6	-0.28773932	0.69501904	-8.265526851	1.28E-15	24.30185307
CCDC50	-0.381750003	2.678159575	-8.260282917	1.33E-15	24.2637699
PCOLCE	-0.693237316	4.233827759	-8.252757992	1.40E-15	24.20915343
RP11-322E11.5	-0.240244756	0.502714233	-8.250355807	1.43E-15	24.19172614
LOXL3	-0.359618169	1.403773809	-8.250219038	1.43E-15	24.19073402
EPHB6	-0.549182626	0.983672141	-8.246523504	1.47E-15	24.16393164
UBE2J1	-0.3348834	4.581435676	-8.245727842	1.48E-15	24.15816217
MRPS9	0.277316584	3.749057493	8.242957836	1.51E-15	24.13807976
IGKV1D-39	-1.037176918	1.546556878	-8.241939796	1.52E-15	24.1307003
IGKC	-1.38663924	9.800009284	-8.23682669	1.58E-15	24.09364743
ARSI	-0.401458373	1.092528748	-8.235382383	1.60E-15	24.08318421
TNFSF10	-0.789010841	4.998384518	-8.230754299	1.65E-15	24.04966564
IGHV3-48	-1.314900226	4.070129306	-8.229201966	1.67E-15	24.03842619
IGHV3-62	-0.608446984	0.968547732	-8.22914486	1.67E-15	24.03801276
KAL1	-0.692287979	2.195919861	-8.222411632	1.75E-15	23.98928063
SNAI2	-0.68787085	2.197680687	-8.219769742	1.79E-15	23.97016807
IGKV3-15	-1.357209432	6.252110455	-8.218902436	1.80E-15	23.96389464
ITGB2-AS1	-0.647898467	1.469482668	-8.218302697	1.81E-15	23.95955687
KCNN3	-0.296342864	0.65986059	-8.213347404	1.88E-15	23.92372563
CTSC	-0.536083631	4.759645842	-8.205807342	1.98E-15	23.86923571
NABP1	-0.446949109	1.762011078	-8.204497743	2.00E-15	23.85977548
TMEM109	-0.335081311	5.599316459	-8.204288345	2.00E-15	23.85826295
RP11-212I21.2	-0.425160646	0.652921324	-8.20405709	2.01E-15	23.85659257
IGHJ2	-1.29250028	3.682427187	-8.203785411	2.01E-15	23.85463025
MIR7848	-0.346651861	0.503076676	-8.199992836	2.07E-15	23.8272419
ANKRD22	-0.888423009	3.116451731	-8.198675738	2.09E-15	23.81773264
MCEMP1	-0.920622651	1.909999811	-8.198599837	2.09E-15	23.81718467
RP11-588K22.2	-0.541653406	2.557531678	-8.195917894	2.13E-15	23.79782515
IGHV3-21	-1.413949947	6.001235921	-8.191702132	2.20E-15	23.76740355
RP13-297E16.4	-0.363256388	0.616098414	-8.190390998	2.22E-15	23.75794464

PCDH18	-0.342984502	1.106975697	-8.187620981	2.26E-15	23.73796468
LY96	-0.604606563	4.29792439	-8.187177788	2.27E-15	23.73476844
CCL14	-0.300991013	0.45038176	-8.187147062	2.27E-15	23.73454685
IGHV4-59	-1.41323774	5.91809139	-8.186739417	2.28E-15	23.73160709
KLF6	-0.525163684	4.696365534	-8.186528032	2.28E-15	23.73008273
THSD1	-0.336864443	1.13588363	-8.184199938	2.32E-15	23.71329602
UNC5B	-0.538371915	2.775198525	-8.182128637	2.36E-15	23.69836399
NKD2	-0.620283733	2.046591947	-8.178040822	2.43E-15	23.66890331
TBC1D9	-0.412135058	3.147635553	-8.177303889	2.44E-15	23.66359347
AC002331.1	-0.548567521	0.897584816	-8.176842902	2.45E-15	23.66027209
PCED1B	-0.575541742	2.815044362	-8.17454105	2.49E-15	23.64368955
GNA13	-0.371193148	4.007687789	-8.170404646	2.57E-15	23.61389985
MOB3C	-0.307867889	2.334232517	-8.164346071	2.68E-15	23.57028773
NIPSNAP1	0.429159732	4.742040239	8.158264	2.81E-15	23.52653132
IGKV2-28	-0.912543251	1.450863733	-8.156954677	2.83E-15	23.51711487
COL5A1	-0.860430815	4.519873493	-8.155789459	2.86E-15	23.50873577
IL33	-0.800501527	2.694142473	-8.153217837	2.91E-15	23.49024644
GUCY1A3	-0.540509725	2.426389121	-8.152740519	2.92E-15	23.48681514
MAN1C1	-0.411444915	1.366922895	-8.150266154	2.97E-15	23.46903006
SSC5D	-0.563448351	1.952513399	-8.149048154	3.00E-15	23.46027692
FUOM	-0.599146062	2.693214638	-8.148862404	3.01E-15	23.45894211
CASP10	-0.411277226	2.53639909	-8.148844946	3.01E-15	23.45881666
RANBP3L	-0.212771918	0.328856201	-8.146860632	3.05E-15	23.44455882
TRBJ2-4	-0.363911448	0.386474991	-8.145353886	3.08E-15	23.4337342
TCF7	-0.387023869	1.323904071	-8.145119455	3.09E-15	23.43205016
PELP1	0.360751478	3.510591526	8.14444162	3.10E-15	23.42718114
IGLV3-27	-1.433450898	3.688276424	-8.143146304	3.13E-15	23.41787748
OTUD1	-0.354435522	2.77847665	-8.141613365	3.17E-15	23.40686854
TNFAIP2	-0.812930455	4.475802347	-8.132203575	3.39E-15	23.33932599
IQSEC1	-0.382867916	3.201094117	-8.131988593	3.40E-15	23.33778358
FXYD6	-0.469448758	1.955537095	-8.128538816	3.48E-15	23.31303687
IGHV2-70	-1.3784528	3.939345455	-8.126045376	3.55E-15	23.29515538
TAOK3	-0.32179955	2.467825307	-8.11953876	3.72E-15	23.2485135

NECAB3	0.551714396	3.458749892	8.116491575	3.80E-15	23.22667995
IGKV2-30	-1.13427125	2.734931486	-8.114476801	3.86E-15	23.21224723
IGLV2-11	-1.396523458	6.290399077	-8.11103883	3.96E-15	23.18762585
IGHV3-72	-1.185716748	3.400123075	-8.105424735	4.12E-15	23.14743711
CCDC170	-0.564105011	1.047212854	-8.101470921	4.24E-15	23.11914636
IGLV1-51	-1.40049356	6.869324176	-8.095574552	4.43E-15	23.07697568
RIC1	-0.314879149	1.989953732	-8.088416763	4.66E-15	23.02581496
AMPD1	-0.333671021	0.476206626	-8.088351441	4.67E-15	23.02534823
IGHV3-63	-0.692383698	1.388162771	-8.087833237	4.68E-15	23.02164571
GNL3	0.354580948	4.714126827	8.084184059	4.81E-15	22.99557779
AHNAK	-0.76023071	4.749232712	-8.080699127	4.93E-15	22.97069159
IL1R1	-0.581704477	4.286011436	-8.077292179	5.06E-15	22.94637024
SPATA20P1	-0.424540027	1.027509416	-8.077141553	5.06E-15	22.94529514
EPB41L3	-0.512528636	1.668363617	-8.07695731	5.07E-15	22.94398012
KCNJ5	-0.515484468	1.019709827	-8.075227413	5.13E-15	22.93163422
TRAJ5	-0.294701399	0.296116132	-8.07445585	5.16E-15	22.92612839
IGLV3-1	-1.388097222	6.104717514	-8.074167454	5.17E-15	22.92407053
F2R	-0.551115987	3.515994661	-8.060056485	5.73E-15	22.82344958
SULF1	-0.926380129	3.7754056	-8.055410619	5.92E-15	22.79035093
AC104699.1	-0.535831157	0.976841924	-8.053581123	6.00E-15	22.77732104
CD300E	-0.429642307	0.935702912	-8.053142815	6.02E-15	22.77419969
PARVB	-0.386439861	2.066116734	-8.046361168	6.32E-15	22.72592183
IGSF21	-0.341480606	0.634241356	-8.043181579	6.47E-15	22.70329743
LYST	-0.334186022	1.46781037	-8.042767906	6.49E-15	22.70035444
MOB3B	-0.423963283	1.587838807	-8.04275677	6.49E-15	22.70027521
IL18	-0.63387365	3.048089695	-8.04274225	6.49E-15	22.70017192
CREBRF	-0.297730808	1.686919114	-8.03543561	6.84E-15	22.64820967
CTB-41I6.2	-0.328315221	0.751386141	-8.033953244	6.92E-15	22.63767204
STX12	-0.26032406	4.138678369	-8.032210213	7.00E-15	22.62528335
RSAD2	-0.637464385	2.22526839	-8.031173587	7.06E-15	22.61791645
EFEMP2	-0.517382878	3.041715567	-8.01502111	7.93E-15	22.50322165
EBF1	-0.274142303	0.801177735	-8.013951896	7.99E-15	22.4956357
FGF2	-0.318767936	0.619036993	-8.013233856	8.03E-15	22.49054173

RP11-455F5.5	-0.30397368	0.647905577	-8.010004228	8.22E-15	22.4676342
IGHV4-31	-1.380510223	4.502429463	-8.008608389	8.30E-15	22.45773581
TRBV23-1	-0.248417332	0.315929569	-8.003991029	8.58E-15	22.42500197
IGKV3D-15	-1.128697305	2.643628584	-8.003853446	8.59E-15	22.42402682
C1orf56	0.443923833	2.462260925	8.002660095	8.67E-15	22.41556927
CRTAP	-0.340326959	4.490812922	-7.998064272	8.96E-15	22.38300674
SLC11A1	-0.568670755	2.061046224	-7.993024688	9.29E-15	22.34731664
ICA1	0.465609954	3.399141346	7.992438265	9.33E-15	22.34316474
NR2F6	0.44427181	4.509760898	7.991491797	9.39E-15	22.33646421
GRHL2	0.434987353	3.329382382	7.986090006	9.76E-15	22.29823391
TNIP1	-0.327951366	4.866018199	-7.985196947	9.83E-15	22.29191535
MR1	-0.418825106	2.842700076	-7.984590193	9.87E-15	22.28762276
CCL3L3	-0.489845027	1.184961467	-7.982280118	1.00E-14	22.27128204
CEACAM4	-0.485952401	0.956090912	-7.98160352	1.01E-14	22.26649669
APLNR	-0.558827598	2.037265003	-7.977659028	1.04E-14	22.2386049
TLR6	-0.340345666	1.061327441	-7.977244732	1.04E-14	22.235676
IGHV3-66	-1.136752158	3.174875698	-7.976617735	1.05E-14	22.23124362
MCTP1	-0.3465538	1.085502288	-7.9739838	1.07E-14	22.21262667
IGLV1-50	-0.764646184	1.444494413	-7.973852223	1.07E-14	22.2116968
IGKV1-12	-1.033827759	2.524669461	-7.972181684	1.08E-14	22.19989186
VDR	-0.567230199	3.31451585	-7.965426426	1.13E-14	22.15217501
BLOC1S4	0.33414388	3.58874651	7.960391922	1.17E-14	22.11663332
SLFN12	-0.376970247	1.565077131	-7.955255963	1.22E-14	22.08039329
IGLV8-61	-1.472243991	4.680302606	-7.949477867	1.27E-14	22.03964389
AL359753.1	-0.467071566	0.561670842	-7.944129418	1.32E-14	22.00194495
HMHA1	-0.49777446	3.519660548	-7.942868104	1.33E-14	21.99305735
GBGT1	-0.394373859	1.951603995	-7.940858486	1.35E-14	21.97889921
AC027319.1	-0.284498412	0.395056589	-7.940562606	1.35E-14	21.97681492
ACKR1	-0.850747375	2.339769333	-7.939764164	1.36E-14	21.97119068
IGLV1-47	-1.336299727	6.286524819	-7.938147253	1.38E-14	21.95980248
U62631.5	-0.261504716	0.325710052	-7.937395121	1.38E-14	21.95450569
LPCAT2	-0.435393784	2.145992902	-7.934799449	1.41E-14	21.93622898
SCARF1	-0.515251697	2.135993758	-7.92862421	1.47E-14	21.89276636

ANO6	-0.384712726	3.423577196	-7.928312224	1.48E-14	21.89057124
CNN1	-0.63121618	2.230707894	-7.9266229	1.50E-14	21.87868635
GATA3	-0.490992178	1.255220876	-7.925512627	1.51E-14	21.87087633
TPP1	-0.365523132	5.621189734	-7.925232472	1.51E-14	21.86890576
HLA-H	-0.703043411	5.173463758	-7.921041893	1.56E-14	21.8394363
PET117	0.316927313	2.38928623	7.919067219	1.58E-14	21.82555397
MAP1LC3C	-0.627063903	1.228420706	-7.918097563	1.59E-14	21.81873808
FAM86JP	0.298842397	1.249127591	7.912548476	1.65E-14	21.77974501
LYSMD2	-0.366855777	2.700373478	-7.912396819	1.65E-14	21.77867963
IDS	-0.538744199	4.107690466	-7.911025502	1.67E-14	21.76904688
HCG4P7	-0.556140025	1.960804377	-7.909370867	1.69E-14	21.75742571
PRR16	-0.324801461	0.945896941	-7.908087059	1.71E-14	21.7484103
SYNDIG1	-0.479014544	0.938454185	-7.905579468	1.74E-14	21.73080429
FOXF1	-0.446813135	1.6177577	-7.901303562	1.79E-14	21.70079277
NELFCD	0.351549304	3.909058052	7.897684337	1.84E-14	21.67540019
IGHV3-13	-1.291877127	3.323153136	-7.893152906	1.90E-14	21.64362027
CCDC88A	-0.387588185	1.744571964	-7.891622511	1.92E-14	21.63289047
RP4-575N6.4	-0.228739389	0.526247243	-7.891225085	1.92E-14	21.63010434
DDX60	-0.603285955	2.710334131	-7.887717368	1.97E-14	21.60551834
XXbac-BPG170G13.31	-0.361077067	0.536045636	-7.886636278	1.99E-14	21.59794257
RCCD1	0.329394934	1.859633693	7.886299969	1.99E-14	21.59558603
GPR176	-0.514497431	1.578645806	-7.878978002	2.10E-14	21.54430005
TRGC2	-0.442470897	0.899854865	-7.87860781	2.11E-14	21.54170807
CREBL2	-0.341763882	4.196593339	-7.878479622	2.11E-14	21.54081055
ASPN	-0.850875607	3.321437021	-7.878167757	2.11E-14	21.53862705
PLAC9	-0.496087041	1.920139053	-7.872894917	2.19E-14	21.50171987
NIN	-0.328162343	2.051387141	-7.872187162	2.20E-14	21.4967674
CPPED1	-0.412446236	2.184640818	-7.871884653	2.21E-14	21.49465073
RP11-672A2.4	-0.284226531	0.694079283	-7.868657337	2.26E-14	21.4720729
RP6-159A1.4	-0.39963786	0.818708479	-7.868045638	2.27E-14	21.46779435
SHMT1	0.425148217	2.579675228	7.867738588	2.27E-14	21.46564678
ARHGAP6	-0.236779603	0.590655481	-7.866694097	2.29E-14	21.45834188
TBXAS1	-0.476857807	2.406370313	-7.861596502	2.38E-14	21.42270145

RTP4	-0.62013195	2.332194852	-7.861586537	2.38E-14	21.42263179
IGKV6D-21	-1.047634665	1.711752328	-7.86131897	2.38E-14	21.42076157
LIMS2	-0.409521347	1.4767578	-7.859171636	2.42E-14	21.40575402
TMEM156	-0.649317083	1.535652587	-7.8509925	2.56E-14	21.34861993
IGHV3-76	-0.573764326	0.863099152	-7.850079366	2.58E-14	21.34224425
POLR2H	0.359064011	4.135644403	7.849560984	2.59E-14	21.33862506
RNF130	-0.314339254	2.991706404	-7.84951	2.59E-14	21.33826912
EMP1	-0.629460449	3.971701714	-7.849409501	2.59E-14	21.33756749
IGKV1OR2-108	-1.137422512	2.955180111	-7.842947199	2.71E-14	21.29246592
IGKV3D-11	-0.975917894	2.480684828	-7.838691288	2.79E-14	21.26277897
ZNF25	-0.266996578	1.829493792	-7.835717692	2.85E-14	21.24204421
IGLL5	-1.185543903	4.955561664	-7.835327548	2.86E-14	21.2393242
IGHV3OR16-10	-0.527653324	0.780832525	-7.832847144	2.91E-14	21.22203381
CARD17	-0.301083774	0.450677579	-7.827979667	3.02E-14	21.18811602
ALDH1A3	-0.634398289	1.906411586	-7.825815869	3.06E-14	21.17304342
TXLNB	-0.245709414	0.459263016	-7.824971241	3.08E-14	21.16716079
ARHGAP17	-0.259544953	2.538141722	-7.822427293	3.14E-14	21.14944578
TIMP1	-0.59611403	8.149015717	-7.81975671	3.20E-14	21.13085378
FRMD6	-0.549463097	1.770623807	-7.819443668	3.20E-14	21.12867478
LIX1L	-0.469238519	3.104227482	-7.814545739	3.32E-14	21.09459043
CTB-41I6.1	-0.305610272	0.648074275	-7.811840398	3.38E-14	21.0757713
CPXM1	-0.707998242	2.522449324	-7.808294268	3.47E-14	21.05111109
CTSG	-0.497229889	0.802820448	-7.805499166	3.53E-14	21.03167979
PACSIN3	0.534110487	3.692956634	7.805269406	3.54E-14	21.03008276
IGHV3-7	-1.024699442	2.633123373	-7.804946933	3.55E-14	21.02784136
PATL2	-0.276583336	0.599487897	-7.80490717	3.55E-14	21.02756498
FOXP4-AS1	0.361044626	0.723826747	7.804091236	3.57E-14	21.02189404
HERC5	-0.479594093	1.753825875	-7.802724307	3.60E-14	21.01239459
IGHV6-1	-1.036460283	2.234489475	-7.801026784	3.65E-14	21.00059948
RP11-81H14.2	-0.255822115	0.4052953	-7.797476197	3.74E-14	20.975935
RGS16	-0.673570685	3.607519848	-7.790227785	3.94E-14	20.92561047
SAMD4A	-0.45558825	1.621179163	-7.786984537	4.03E-14	20.90310498
IGHV3-60	-0.628071994	1.144276962	-7.786367153	4.05E-14	20.89882167

PPME1	0.301038672	3.236911903	7.786171642	4.05E-14	20.8974653
PRR36	0.664010992	2.408827372	7.785222909	4.08E-14	20.89088379
COL5A2	-0.823679444	4.468301001	-7.783914711	4.12E-14	20.88180964
IGLC3	-1.302600987	8.147919868	-7.778964182	4.26E-14	20.84748175
IRAK3	-0.56699864	1.782613824	-7.778867267	4.27E-14	20.84680989
C8orf82	0.424950795	3.089844945	7.778156805	4.29E-14	20.84188486
FAM83H	0.524903651	4.529521739	7.775148495	4.38E-14	20.82103471
GNLY	-0.818256397	2.077198126	-7.772857933	4.45E-14	20.8051634
PFDN6	0.362807807	3.776352151	7.772289595	4.47E-14	20.80122595
NALCN	-0.217615716	0.454276144	-7.769954532	4.54E-14	20.78505099
MCOLN1	-0.309714503	2.901718274	-7.769868045	4.54E-14	20.78445197
ANXA5	-0.352340011	6.849056826	-7.769452231	4.56E-14	20.78157205
EXOC3L4	-0.296378042	0.480488031	-7.769372435	4.56E-14	20.7810194
SMOC2	-0.733382491	2.563337974	-7.761823866	4.81E-14	20.72875961
HAMP	-0.355734842	0.604658912	-7.761799698	4.81E-14	20.72859235
RP11-876N24.3	-0.581113435	1.739577158	-7.760949598	4.84E-14	20.7227095
ETV7	-0.561680486	2.462385349	-7.760708179	4.85E-14	20.72103892
WARS	-0.665801627	5.347515951	-7.758813611	4.91E-14	20.70793029
IGKV3-7	-0.997636036	2.537371793	-7.756754084	4.98E-14	20.69368313
CDC42SE2	-0.335376346	4.07142576	-7.755459705	5.03E-14	20.68473055
EPCAM	0.513936347	7.474375776	7.75477447	5.05E-14	20.67999159
RP11-731F5.2	-0.360489839	0.56926547	-7.750745267	5.20E-14	20.65213299
NXPE3	-0.360380263	1.541184641	-7.750343564	5.21E-14	20.64935616
HERC3	-0.324714183	1.937077734	-7.749004322	5.26E-14	20.64009931
KLRC1	-0.240256873	0.353738721	-7.748249427	5.29E-14	20.63488201
IGHV1-2	-1.428782294	5.501475189	-7.74663353	5.35E-14	20.62371543
NDN	-0.603663435	3.083997359	-7.746578703	5.35E-14	20.62333658
HSPA12B	-0.378998297	1.445757488	-7.740380213	5.59E-14	20.58051938
CARD9	-0.323820134	0.906922925	-7.738201071	5.68E-14	20.56547294
IGHV1OR15-2	-0.995875223	2.068588134	-7.737486553	5.71E-14	20.56054009
CTGF	-0.765903155	5.978259232	-7.736905731	5.73E-14	20.55653051
KIAA1549	0.429497364	1.366539593	7.735803327	5.77E-14	20.54892095
HMCN1	-0.498810476	1.384531265	-7.734212443	5.84E-14	20.53794106

IGHV2-26	-1.343645183	3.944115228	-7.729850789	6.02E-14	20.50784708
IGLC2	-1.233842727	8.878279629	-7.727155179	6.13E-14	20.48925491
CCDC58	0.38865046	3.091445414	7.725205953	6.22E-14	20.47581388
SGK1	-0.575250139	3.351611877	-7.723732567	6.28E-14	20.4656558
IGKV1OR22-5	-0.591315409	0.896715882	-7.722308501	6.35E-14	20.4558392
IGKV1-9	-1.34182777	5.597203232	-7.71965237	6.47E-14	20.43753334
TBC1D30	0.40472138	1.751956931	7.717665508	6.56E-14	20.42384325
SRPX	-0.619228789	2.090651983	-7.717593774	6.56E-14	20.42334903
AC103563.5	-0.897228723	1.545412412	-7.717323515	6.57E-14	20.42148709
SETDB2	-0.252694106	1.687340972	-7.715633437	6.65E-14	20.40984453
AAED1	-0.307924366	2.604763145	-7.71387833	6.73E-14	20.39775612
CEP120	-0.281667341	2.167795237	-7.712277495	6.81E-14	20.38673214
RP11-686D22.7	-0.384144872	1.646802602	-7.711074361	6.87E-14	20.37844808
GYLTL1B	0.776538609	3.339040331	7.711062073	6.87E-14	20.37836347
IGKV2D-29	-1.32513414	3.594413077	-7.707714464	7.03E-14	20.3553192
EEF1E1	0.352626854	2.60692603	7.707029335	7.06E-14	20.35060388
IGHJ1	-1.086229564	2.284912793	-7.706095424	7.11E-14	20.34417687
IGLV3-16	-0.908825497	1.661395082	-7.701081665	7.36E-14	20.30968356
ENOX1	-0.242284647	0.647606976	-7.698305968	7.51E-14	20.2905951
IGKV2OR22-4	-0.754781	1.369212254	-7.697772197	7.53E-14	20.28692498
APOA1BP	0.447594123	5.967346612	7.695608786	7.65E-14	20.27205177
HS3ST2	-0.688159496	1.660598693	-7.6943042	7.72E-14	20.26308448
ROR1	-0.405388263	1.141841167	-7.69355764	7.76E-14	20.25795342
MTMR6	-0.326357914	2.914260647	-7.693504606	7.76E-14	20.25758893
SH3KBP1	-0.394936779	3.601661012	-7.692546238	7.81E-14	20.25100272
PITPNA-AS1	0.432757705	2.50283943	7.686711068	8.14E-14	20.21091554
ATXN1	-0.290319469	2.001144238	-7.686578321	8.15E-14	20.21000387
PPAP2B	-0.405765664	3.664094795	-7.686485377	8.15E-14	20.20936555
ZBTB42	0.399009944	2.828784703	7.685829588	8.19E-14	20.20486193
NOP58	0.298364141	4.540349317	7.684161469	8.29E-14	20.19340751
CLIP3	-0.494476442	2.257094676	-7.680875241	8.48E-14	20.1708478
AC007386.4	-0.296146235	0.514801048	-7.679950477	8.53E-14	20.16450073
IGKV1-39	-1.077890491	2.156685734	-7.678730835	8.61E-14	20.15613071

ADH1B	-1.017200292	2.176568103	-7.677868175	8.66E-14	20.15021117
IL4R	-0.391206098	4.088521942	-7.67775616	8.66E-14	20.14944257
ANKRD39	0.240399889	1.721515334	7.677361916	8.69E-14	20.14673749
TSPAN33	-0.537089024	2.598587594	-7.675868679	8.78E-14	20.13649273
FAM124B	-0.203301954	0.521168844	-7.671880799	9.03E-14	20.10914049
PIK3R1	-0.382330702	2.383593139	-7.666063353	9.40E-14	20.06925961
BDH1	0.400280025	1.772398534	7.665785352	9.42E-14	20.0673544
CLIC2	-0.662013946	3.526491113	-7.665056467	9.47E-14	20.06235943
RBBP8NL	0.401555677	1.481715058	7.65962783	9.83E-14	20.02516936
IGLC6	-0.694235739	1.543987966	-7.658039861	9.94E-14	20.01429455
RNF125	-0.307627592	1.206298517	-7.655066514	1.01E-13	19.99393711
LYVE1	-0.508828599	1.381359567	-7.654234336	1.02E-13	19.9882406
IGHV4-34	-1.31759031	5.865910377	-7.652588293	1.03E-13	19.97697437
TRAJ4	-0.283583507	0.311270522	-7.651496236	1.04E-13	19.96950093
PSTPIP2	-0.426862079	2.068662584	-7.649966313	1.05E-13	19.95903237
C1orf186	-0.427570028	0.87750773	-7.648267434	1.06E-13	19.94740967
TNFRSF14	-0.515791695	3.409452585	-7.647041096	1.07E-13	19.93902107
LL22NC03-88E1.18	-0.426580688	0.571358603	-7.643140054	1.10E-13	19.91234358
MYBPC2	-0.312163363	0.484288978	-7.64288963	1.10E-13	19.9106314
HLA-DRB6	-1.051368249	4.853580029	-7.642647688	1.11E-13	19.90897727
IGHJ3P	-1.163237604	3.056678734	-7.642600947	1.11E-13	19.9086577
EIF4E3	-0.329469585	1.805881887	-7.641115599	1.12E-13	19.89850346
IPO4	0.266228595	1.21414427	7.637357733	1.15E-13	19.87282061
ADAMTS8	-0.507982465	0.863161099	-7.627343895	1.23E-13	19.80443051
TNFSF13	-0.454914281	3.280939884	-7.623309266	1.27E-13	19.7768958
IMP4	0.258376226	3.965229209	7.620538064	1.29E-13	19.75799013
LINC00926	-0.427043772	0.861950049	-7.618388423	1.31E-13	19.74332861
VSTM4	-0.437631405	1.53257957	-7.616042238	1.33E-13	19.7273303
CAP1	-0.275259876	6.679441283	-7.613931049	1.35E-13	19.71293774
KCNMA1	-0.329467005	0.739596536	-7.609204354	1.40E-13	19.68072596
TNFRSF13C	-0.518180993	1.10497771	-7.608176014	1.41E-13	19.67372006
DNASE2	-0.274913785	5.082187642	-7.607356841	1.41E-13	19.66813971
SPIRE2	0.474388524	1.816800678	7.606425484	1.42E-13	19.66179573

MCC	-0.337137785	1.094417935	-7.600866277	1.48E-13	19.62394168
ITGA5	-0.612427733	3.634919969	-7.598580676	1.50E-13	19.6083848
NR1H3	-0.400975006	2.911003464	-7.597287232	1.52E-13	19.59958264
TM7SF2	0.554323103	3.413559498	7.591552294	1.58E-13	19.5605695
CD163L1	-0.399762338	1.032664452	-7.590048745	1.59E-13	19.55034514
IGHV3-33	-1.337578912	5.228329803	-7.589748198	1.60E-13	19.54830157
NAV3	-0.252282251	0.423626699	-7.589444385	1.60E-13	19.54623586
IGHV3OR16-8	-0.69768906	1.290887309	-7.589338599	1.60E-13	19.5455166
MYH11	-0.767716546	2.42178609	-7.589185806	1.60E-13	19.54447776
PAMR1	-0.413820627	1.172327354	-7.581763538	1.69E-13	19.49403327
RP11-148O21.2	-0.389698345	0.533466401	-7.581638185	1.69E-13	19.49318166
ATP10A	-0.55141362	1.785180905	-7.577547549	1.74E-13	19.46539726
IGKV1-33	-0.841033017	1.365866729	-7.57704223	1.74E-13	19.46196586
COL15A1	-0.730550406	3.027562149	-7.567515413	1.86E-13	19.39730733
RTKN	0.346112321	3.17641646	7.565923327	1.88E-13	19.38650813
CCT3	0.383734024	6.350890171	7.562967687	1.92E-13	19.36646465
LNPEP	-0.294995053	2.231080223	-7.56294247	1.92E-13	19.36629367
IGHV3OR16-6	-0.595047802	0.928583424	-7.561027154	1.95E-13	19.35330843
IGKV1OR9-2	-0.381569153	0.463415537	-7.560008218	1.96E-13	19.34640142
PLB1	-0.230841604	0.694159079	-7.558221079	1.98E-13	19.33428883
PLCL1	-0.202901344	0.598104162	-7.55482787	2.03E-13	19.31129711
IGHV4-39	-1.362779076	6.603866731	-7.55305606	2.06E-13	19.29929493
MRPS18A	0.328922787	4.113655372	7.546647979	2.15E-13	19.25590547
PYCARD	-0.572013878	3.619660603	-7.54074265	2.24E-13	19.21594605
EIF2B4	0.214567078	3.097428476	7.540667882	2.24E-13	19.21544027
WNT10A	-0.615379442	1.17208193	-7.537156879	2.29E-13	19.19169443
AL122127.2	-0.790814457	1.93914198	-7.535695954	2.32E-13	19.1818164
TNC	-1.096069224	4.296516838	-7.532168945	2.37E-13	19.15797482
IGKV1D-16	-1.104288526	2.563999966	-7.531386889	2.39E-13	19.15268954
RP11-212I21.5	-0.2018995	0.265749983	-7.528377898	2.44E-13	19.13235831
HTRA3	-0.805184047	4.031220963	-7.525742058	2.48E-13	19.11455369
TRAJ1	-0.329238318	0.399728176	-7.518096871	2.62E-13	19.0629399
AP000892.6	-0.278235882	0.733241066	-7.517547423	2.63E-13	19.0592321

LINC00582	-0.332434376	0.568714077	-7.512942383	2.71E-13	19.02816473
IGHV3-42	-0.453158314	0.636989582	-7.508865116	2.79E-13	19.00067056
MAML2	-0.486410239	2.211276206	-7.508572755	2.79E-13	18.99869954
RP1-266L20.2	0.414274149	1.996752152	7.508354915	2.80E-13	18.99723097
TCAF2	-0.254765945	0.732883759	-7.507137684	2.82E-13	18.98902558
C3	-0.988679031	6.534657546	-7.507012923	2.82E-13	18.98818462
NCEH1	-0.523099205	3.350905457	-7.505213839	2.86E-13	18.97605904
SFRP2	-1.171771158	5.2218016	-7.49801286	3.00E-13	18.9275486
ZBTB12	0.435913554	2.138294327	7.497822897	3.01E-13	18.92626938
SLC25A10	0.492705067	2.87649836	7.4958468	3.05E-13	18.91296387
TNF	-0.45214367	0.993313576	-7.487296338	3.23E-13	18.85542384
TBC1D27	-0.232373584	0.268172873	-7.486753465	3.24E-13	18.85177236
LINC01270	0.374150576	1.007724071	7.485253728	3.28E-13	18.84168591
AC246787.3	-0.794588993	1.50295347	-7.483628733	3.31E-13	18.83075886
PSMB8	-0.500495728	5.808777612	-7.478077132	3.44E-13	18.79344215
OSTN-AS1	-0.211400032	0.259476783	-7.476550644	3.48E-13	18.78318529
KDF1	0.3446946	3.707122232	7.470774736	3.62E-13	18.74439059
METTL21A	0.241140957	1.799602247	7.46931506	3.65E-13	18.73459026
TSPAN4	-0.490214073	3.413144594	-7.468601532	3.67E-13	18.72980015
IGHV1-12	-0.911230509	1.953876666	-7.468486911	3.67E-13	18.7290307
IGHV3-38	-0.610855685	1.168385065	-7.467169494	3.71E-13	18.72018759
CDO1	-0.370014537	0.770996783	-7.465102916	3.76E-13	18.70631828
MAP7D3	-0.300313555	1.048203336	-7.464683894	3.77E-13	18.70350649
POLR1C	0.307722718	3.475524539	7.462949973	3.82E-13	18.69187261
TPM4	-0.423011641	5.880043561	-7.462424996	3.83E-13	18.68835066
EMR3	-0.202480011	0.351175024	-7.461094885	3.86E-13	18.67942813
VENTX	-0.259842238	0.605264111	-7.460805697	3.87E-13	18.67748839
SEMA3C	-0.684745671	3.182238786	-7.460580634	3.88E-13	18.67597882
hsa-mir-4538	-0.642918892	0.97742919	-7.458500388	3.93E-13	18.66202762
CH25H	-0.834778527	2.457696203	-7.456133984	4.00E-13	18.64616107
IGHA1	-1.250244471	10.61278728	-7.455899159	4.00E-13	18.64458681
HMOX1	-0.569668963	4.590888101	-7.444481823	4.33E-13	18.56809291
SENCR	-0.223119687	0.526638442	-7.442265512	4.39E-13	18.55325492

RENBP	-0.588875534	3.067134198	-7.441853937	4.41E-13	18.55049985
ARFIP2	0.298977017	3.65510568	7.440490307	4.45E-13	18.54137263
HCFC2	-0.241430432	1.626977738	-7.438797935	4.50E-13	18.53004689
ARHGEF15	-0.378727369	1.465056036	-7.43710339	4.55E-13	18.51870868
CFLAR	-0.375233962	3.112622837	-7.436460928	4.57E-13	18.51441049
SLC29A2	0.509583659	3.355737468	7.430539369	4.76E-13	18.47480824
MMP14	-0.644779263	5.97839276	-7.424656693	4.95E-13	18.435491
IGHV3-41	-0.661126533	1.310839044	-7.422444743	5.03E-13	18.42071374
ITPRIPL1	-0.249205305	0.670917022	-7.420905384	5.08E-13	18.41043189
SERINC1	-0.331239957	5.885110322	-7.419344651	5.14E-13	18.40000903
GREM1	-0.799158622	1.859482881	-7.418824222	5.15E-13	18.3965339
IGHM	-1.378238535	7.473838611	-7.417150556	5.21E-13	18.38535943
DHRS9	-0.727999424	1.492021112	-7.412599318	5.38E-13	18.35498263
IGKV1OR2-3	-0.541805993	0.797904696	-7.411814903	5.40E-13	18.34974863
AP001469.9	0.279228962	0.857382754	7.409870171	5.48E-13	18.33677435
HYKK	0.245578248	0.779150353	7.40848787	5.53E-13	18.32755399
NTM	-0.508840174	1.489942405	-7.407454645	5.57E-13	18.32066297
IGHV3-47	-0.6294387	1.2716445	-7.405489382	5.64E-13	18.30755791
RALB	-0.350673396	4.729338607	-7.403449427	5.72E-13	18.29395772
AC103563.2	-0.671606323	1.102930565	-7.402238775	5.77E-13	18.28588784
IGKV6-21	-1.197511158	2.895110157	-7.39901928	5.90E-13	18.26443269
IGLV3-10	-1.434468834	5.42635994	-7.398635005	5.91E-13	18.26187233
MRPS5	0.242638449	3.220627127	7.396731448	5.99E-13	18.24919081
IL27RA	-0.586546383	3.615585003	-7.393999083	6.10E-13	18.23099233
AF131215.2	-0.325191473	0.755159448	-7.393328892	6.13E-13	18.22652945
MTX2	0.273970166	4.581796725	7.391746708	6.19E-13	18.2159948
IGHD3-3	-0.940362782	1.72199914	-7.389310414	6.30E-13	18.19977676
ZNF385A	-0.475933638	3.711982146	-7.38804173	6.35E-13	18.19133302
LRRN3	-0.293469014	0.509803621	-7.386250337	6.43E-13	18.17941237
MYADM	-0.541019862	5.110675435	-7.385965475	6.44E-13	18.17751699
FXYD1	-0.209800602	0.407177585	-7.383506051	6.55E-13	18.16115527
PI4K2A	-0.277890687	3.250966402	-7.382605394	6.59E-13	18.15516459
IGHV5-78	-0.684457889	1.656912682	-7.381993764	6.62E-13	18.15109669

RHBDF2	-0.42486737	2.999936482	-7.3789721	6.75E-13	18.13100386
CHSY3	-0.353155964	0.995170014	-7.377862681	6.80E-13	18.12362833
BCL6B	-0.382774081	1.910269517	-7.376588311	6.86E-13	18.11515729
ANP32A	0.257069183	4.077560136	7.373674507	7.00E-13	18.09579294
PAICS	0.420217604	4.135953382	7.370346181	7.16E-13	18.07368131
CLEC4G	-0.20553414	0.296906654	-7.369521192	7.20E-13	18.06820176
IGHV3OR15-7	-0.737116075	1.44122658	-7.368739134	7.24E-13	18.06300781
DBNDD1	0.485918071	2.884527865	7.368672382	7.24E-13	18.0625645
RP11-166D19.1	-0.470497672	1.250019995	-7.365875807	7.38E-13	18.04399512
CCDC71L	-0.444116686	3.203789177	-7.362345021	7.56E-13	18.02055863
CORIN	-0.217463527	0.406239396	-7.348640313	8.29E-13	17.92967561
ZNF101	-0.268750251	1.537755662	-7.345220935	8.48E-13	17.90702114
CAV1	-0.821105612	4.463198969	-7.342452411	8.64E-13	17.888685
IKBKE	-0.348927106	2.651975076	-7.34136524	8.71E-13	17.88148611
LAMP5	-0.577327148	1.741584376	-7.340768657	8.74E-13	17.87753609
FFAR4	-0.337386031	0.658782381	-7.339543805	8.81E-13	17.86942708
PDHX	0.282908726	3.148203888	7.339503858	8.82E-13	17.86916263
RRAS	-0.466784318	5.285141214	-7.338139401	8.90E-13	17.86013069
HSPD1	0.40893053	6.253156016	7.337790838	8.92E-13	17.85782362
SAMD1	0.336487714	4.128833536	7.336081061	9.02E-13	17.84650819
RSPO1	-0.229166616	0.262695414	-7.334488261	9.12E-13	17.83596883
IL1RN	-0.647898326	2.505838313	-7.333432175	9.18E-13	17.82898186
IGHD3-9	-0.780182192	1.330661942	-7.332494438	9.24E-13	17.82277855
TUBA1A	-0.577022671	5.243154466	-7.331770568	9.29E-13	17.81799045
IGHV3-35	-0.756611918	1.551128807	-7.330819795	9.35E-13	17.81170206
IGHV1-45	-1.020741722	2.182751972	-7.321351124	9.96E-13	17.74911226
P2RY1	-0.274915337	0.879618052	-7.320249646	1.00E-12	17.7418355
ZBTB47	-0.328383188	2.185883503	-7.316033315	1.03E-12	17.71398903
AP001434.2	-0.336441197	0.816060209	-7.314242891	1.04E-12	17.70216821
TWIST2	-0.404554029	1.049117163	-7.308631916	1.09E-12	17.66513825
NOL10	0.255005424	3.340690649	7.306616262	1.10E-12	17.65184142
FAM136A	0.303014473	4.061409061	7.304376296	1.12E-12	17.6370683
RP11-294C11.2	-0.647694962	1.192883671	-7.303267092	1.12E-12	17.62975419

IGHV3-37	-0.343138612	0.483781869	-7.302681295	1.13E-12	17.62589179
SMARCA2	-0.431027911	3.440377535	-7.302155916	1.13E-12	17.62242797
CNOT6L	-0.264171614	2.487489181	-7.296748991	1.18E-12	17.58679177
LYSMD1	0.3554703	2.535499098	7.296727034	1.18E-12	17.5866471
GUCY1B3	-0.427041497	2.469410445	-7.296439663	1.18E-12	17.58475368
VCAN	-0.775865039	3.710311035	-7.295652984	1.18E-12	17.57957075
IGHV1OR16-1	-0.442980371	0.601486584	-7.293453139	1.20E-12	17.56507977
TPSD1	-0.613418335	1.042995394	-7.292021769	1.21E-12	17.55565283
FLVCR1	0.372839448	2.198723817	7.291968429	1.21E-12	17.55530156
RAB42	-0.494019096	2.039015039	-7.285160255	1.27E-12	17.51048392
MXRA8	-0.681567993	4.543724636	-7.283866741	1.28E-12	17.50197263
DBH-AS1	-0.376022366	0.825727779	-7.282756428	1.29E-12	17.49466778
HERPUD1	-0.397233228	4.963669763	-7.281778736	1.30E-12	17.4882362
IGHG1	-1.344388716	9.072148864	-7.281201899	1.30E-12	17.4844419
IGLC7	-1.253619253	3.424107791	-7.28090472	1.31E-12	17.48248722
APOL1	-0.702578227	5.384083286	-7.280647549	1.31E-12	17.48079575
CABYR	0.800119768	1.57470367	7.280225512	1.31E-12	17.47802001
RP11-22C11.2	0.328885507	1.118712601	7.277572105	1.34E-12	17.46057154
PCDHGA12	-0.205666904	0.453989044	-7.272910559	1.38E-12	17.42993022
TMSB4X	-0.476089223	9.947788495	-7.272739811	1.38E-12	17.42880816
PTPRJ	-0.401611695	2.720381163	-7.272736909	1.38E-12	17.42878909
MEF2A	-0.258836387	2.956847547	-7.272272553	1.38E-12	17.42573771
TMEM9	0.424389119	5.191421815	7.272202342	1.39E-12	17.42527635
HOOK1	0.406046117	2.75922016	7.272060358	1.39E-12	17.42434339
RP3-460G2.2	-0.467800406	1.039811067	-7.27140733	1.39E-12	17.42005256
IGHV1-17	-0.495714484	0.772153344	-7.271249943	1.39E-12	17.41901847
MMP7	-1.233244395	4.292428984	-7.269711775	1.41E-12	17.40891312
EFEMP1	-0.702428034	4.518698235	-7.26740505	1.43E-12	17.39376177
ARAP2	-0.340238115	1.660157989	-7.266057771	1.44E-12	17.38491419
HSPE1	0.400439988	5.444209881	7.264744544	1.46E-12	17.3762915
PPM1K	-0.341303786	1.654220028	-7.264443573	1.46E-12	17.37431549
CD248	-0.564400838	3.622264144	-7.263175805	1.47E-12	17.36599276
NTN4	-0.610417475	3.506674958	-7.258859879	1.51E-12	17.33766806

LTBP1	-0.587043701	3.411985465	-7.257596838	1.53E-12	17.32938151
ACTG2	-0.658201205	2.11577933	-7.255861265	1.55E-12	17.31799666
NRP2	-0.542705787	2.695374233	-7.250995473	1.60E-12	17.28609025
CBX7	-0.417870622	2.072988888	-7.250281654	1.60E-12	17.28141099
C12orf73	0.261692721	1.7789114	7.249633151	1.61E-12	17.27716021
RP11-170L3.7	-0.652356349	1.130555787	-7.248842612	1.62E-12	17.27197884
SERPINB8	-0.333867709	2.036929603	-7.248835077	1.62E-12	17.27192946
SCRIB	0.499129357	4.26513939	7.244678367	1.66E-12	17.24469296
GPRIN3	-0.395793336	1.438443272	-7.243719613	1.68E-12	17.2384126
RP11-435O5.2	0.262417539	1.009565106	7.243184447	1.68E-12	17.23490726
RORA	-0.285337636	1.038369908	-7.241660188	1.70E-12	17.22492451
SNCA	-0.282836464	0.70905093	-7.239357579	1.73E-12	17.20984738
C1QTNF2	-0.248599533	0.623168002	-7.238347874	1.74E-12	17.20323721
STARD10	0.544183641	4.742563423	7.23720494	1.75E-12	17.19575573
CD34	-0.426508013	2.537394108	-7.233252568	1.80E-12	17.16989147
DDX1	0.256015028	4.825575818	7.232941329	1.80E-12	17.16785521
IFI35	-0.514965288	4.491504892	-7.232932208	1.80E-12	17.16779554
PLEKHG1	-0.406013346	2.142535042	-7.232599521	1.80E-12	17.16561904
RP4-564M11.2	-0.200018514	0.363959402	-7.230103669	1.83E-12	17.1492933
ESYT1	-0.286357538	4.656311914	-7.224986085	1.90E-12	17.11583268
SCARF2	-0.4725125	2.02623675	-7.222694549	1.93E-12	17.10085601
IGKV5-2	-1.027220195	2.301583098	-7.219816994	1.96E-12	17.08205476
CARD6	-0.404800188	2.288691216	-7.218658639	1.98E-12	17.07448806
ACP1	0.256255901	4.450590195	7.217533004	2.00E-12	17.06713604
AC246787.4	-0.907867859	1.936459543	-7.216379198	2.01E-12	17.05960099
ENO3	0.735487687	1.459524528	7.213357142	2.05E-12	17.03986976
NUDT15	0.315550727	3.451133942	7.2108484	2.09E-12	17.02349507
FOXF2	-0.533084724	1.897876471	-7.203252874	2.19E-12	16.97394683
SFXN4	0.354288458	3.873643264	7.203224562	2.19E-12	16.97376222
ODC1	0.912544464	5.708679375	7.196999046	2.29E-12	16.93318272
PAG1	-0.500794755	2.244807136	-7.193061756	2.35E-12	16.90753314
FCRL2	-0.407792466	0.737366596	-7.189896632	2.40E-12	16.88692211
HBEGF	-0.593165419	2.9464706	-7.186191487	2.46E-12	16.86280386

OARD1	0.239774487	2.416994029	7.185117716	2.47E-12	16.85581615
TGFB1I1	-0.410000809	2.353665899	-7.184589876	2.48E-12	16.85238147
PRKCQ	-0.395209463	1.207804383	-7.183588821	2.50E-12	16.84586811
FN3K	0.454675849	2.916963224	7.183549383	2.50E-12	16.84561152
TIAM1	-0.390835933	1.277984475	-7.177212983	2.61E-12	16.80440106
CSK	-0.297614897	4.072128521	-7.176285648	2.62E-12	16.79837238
MMRN2	-0.418102262	2.291027508	-7.17231544	2.69E-12	16.77256882
CEBPZOS	0.281400567	3.55677554	7.170549267	2.73E-12	16.76109368
TXNDC15	-0.28839544	3.501375152	-7.169638151	2.74E-12	16.75517488
AC007249.3	0.335752225	0.546854836	7.165220525	2.82E-12	16.72648576
IGHV3-43	-1.135712873	3.404870306	-7.162716468	2.87E-12	16.71023019
CCDC183	0.243729234	0.579381659	7.160941414	2.90E-12	16.69870988
ACER3	-0.256581353	2.241136445	-7.157647209	2.97E-12	16.67733625
SNX29	-0.288588579	1.950364832	-7.154279453	3.03E-12	16.65549367
IGHV1OR21-1	-0.63800038	1.160307577	-7.150406981	3.11E-12	16.63038792
JAM3	-0.450588	2.159716406	-7.148290277	3.16E-12	16.61666972
COL10A1	-0.921325746	3.455970957	-7.147795593	3.17E-12	16.61346419
EDIL3	-0.713626186	2.90803671	-7.143331979	3.26E-12	16.58454829
SSH1	-0.301800759	2.347579688	-7.142090189	3.29E-12	16.57650643
SIGLEC16	-0.207313735	0.385557199	-7.140696003	3.32E-12	16.56747899
AACS	0.300769707	1.886898761	7.139748398	3.34E-12	16.561344
TRAJ10	-0.225148474	0.225632528	-7.136135265	3.42E-12	16.53795795
GLDN	-0.341508865	0.780654147	-7.132250937	3.51E-12	16.51282733
UQCC2	0.399410363	3.274073769	7.129788978	3.57E-12	16.49690484
STOM	-0.488559858	6.244500431	-7.127596127	3.62E-12	16.48272655
MAGEF1	0.363653409	4.885755456	7.127207266	3.63E-12	16.48021266
RP11-295P22.2	-0.709548254	1.292585391	-7.124116415	3.70E-12	16.4602351
PGAP2	0.266116702	3.001888234	7.122809955	3.73E-12	16.45179298
IGHV3OR16-16	-0.343368906	0.445592328	-7.11995225	3.81E-12	16.43333138
ITGAV	-0.517898178	4.496529438	-7.119119012	3.83E-12	16.42794956
GNG7	-0.392129338	1.428616511	-7.118042466	3.85E-12	16.42099698
HLA-L	-0.419060942	1.341643514	-7.118007285	3.85E-12	16.42076979
SLC22A4	-0.39091329	1.107583756	-7.115532119	3.92E-12	16.40478794

AFAP1L1	-0.362151311	1.788760537	-7.115022945	3.93E-12	16.40150082
SUCNR1	-0.566563672	1.177060486	-7.111922079	4.01E-12	16.38148645
HID1	0.510430911	3.802925466	7.111831011	4.01E-12	16.38089877
PKNOX2	-0.271547376	0.568407397	-7.107799124	4.12E-12	16.35488608
IGLV7-46	-1.154054846	4.076457525	-7.105257947	4.19E-12	16.33849724
SAA1	-1.21540702	3.108153125	-7.104231365	4.22E-12	16.33187785
ATIC	0.295349203	4.727434045	7.103793529	4.23E-12	16.32905492
GNA15	-0.469416212	3.541354855	-7.103645306	4.24E-12	16.32809929
TNNI2	-0.563045432	1.267657184	-7.100135451	4.34E-12	16.30547519
AC104654.2	-0.211564619	0.396621403	-7.099785736	4.35E-12	16.30322147
CHRNA5	0.491284905	0.996133793	7.099379065	4.36E-12	16.30060081
NDUFA10	0.243030299	3.247252614	7.093418602	4.53E-12	16.26220461
ATG4D	0.307576461	2.996140153	7.091419754	4.59E-12	16.24933429
TRAJ17	-0.222870517	0.213504992	-7.090779299	4.61E-12	16.24521111
IGLV1-36	-1.203057541	3.349190611	-7.089603286	4.65E-12	16.23764086
ZNFX1	-0.359832577	3.474130234	-7.089129196	4.66E-12	16.23458933
ARHGEF16	0.42136485	2.963874034	7.087808227	4.70E-12	16.22608765
KLHDC7B	-0.565366917	1.383043706	-7.085337188	4.78E-12	16.21018765
ITGAE	-0.318068043	1.955960234	-7.083783571	4.83E-12	16.20019316
BCCIP	0.275003994	3.600694612	7.083050393	4.85E-12	16.19547721
ITGA1	-0.423097409	2.271044708	-7.077987791	5.01E-12	16.16292442
RBM43	-0.308732259	1.946001268	-7.077777921	5.02E-12	16.16157535
MEIS3	-0.319317747	1.044621244	-7.069725375	5.29E-12	16.10983747
IGLV6-57	-1.265648663	5.565765636	-7.067768209	5.36E-12	16.09726988
KCNJ11	0.513911341	1.34137512	7.064247259	5.49E-12	16.07466789
IGHV1-69	-1.275389074	4.04388804	-7.063399517	5.52E-12	16.06922735
IGKV1D-42	-0.671139231	1.207616476	-7.061443381	5.59E-12	16.05667556
C1orf35	0.304659456	2.663476234	7.05988149	5.64E-12	16.04665553
MAP7D1	-0.349351984	4.388116822	-7.058353646	5.70E-12	16.03685567
RP11-650L12.2	0.395934103	0.64370187	7.057586451	5.73E-12	16.0319354
AC016700.5	0.389290203	1.020271715	7.052854237	5.91E-12	16.00159585
ADTRP	-0.410075028	0.916753205	-7.04570055	6.19E-12	15.95576316
MAP3K5	-0.400962463	2.999895438	-7.045207075	6.21E-12	15.95260293

KCTD10	-0.208860286	3.0732105	-7.045018476	6.22E-12	15.95139519
OPLAH	0.521657756	2.828455528	7.04034896	6.41E-12	15.92150112
GALNT15	-0.339885941	0.741756937	-7.038039397	6.51E-12	15.90672138
FCER1A	-0.848361852	1.900700379	-7.034319844	6.67E-12	15.88292694
ESRP2	0.44297362	3.841871388	7.033154781	6.72E-12	15.875476
TXNL4A	0.283243063	3.169872778	7.032449215	6.75E-12	15.87096418
ZDHHC14	-0.215012586	0.794827151	-7.031076821	6.81E-12	15.86218932
ZBTB46	-0.274504404	1.549852985	-7.027495199	6.97E-12	15.83929561
RP11-283G6.3	-0.28011261	0.556120686	-7.026231567	7.03E-12	15.83122076
NRP1	-0.484671364	3.910798536	-7.024797398	7.10E-12	15.82205759
RP11-134L10.1	-0.204200954	0.756564168	-7.023948455	7.14E-12	15.81663425
CTD-2033D15.2	-0.34012813	0.680313488	-7.022065063	7.22E-12	15.80460443
SLC38A5	-0.546895421	2.261659687	-7.020522611	7.30E-12	15.79475426
RCOR2	0.562232673	1.255831159	7.019264171	7.36E-12	15.78671912
RP11-1399P15.1	-0.264070606	0.563687547	-7.017164859	7.46E-12	15.77331765
ACE	-0.458242738	2.367870341	-7.016273173	7.50E-12	15.76762634
MICAL1	-0.433516331	2.918139605	-7.011984972	7.71E-12	15.7402646
NOX4	-0.302747985	0.837500663	-7.009382669	7.85E-12	15.72366676
MIR4539	-0.620731971	1.041112985	-7.009274609	7.85E-12	15.72297765
BRIX1	0.377976782	3.241438641	7.00505489	8.07E-12	15.69607478
PPP1R16A	0.412835401	2.700012231	7.001655159	8.25E-12	15.67440942
IGKV1OR2-11	-0.503022997	0.761291747	-7.000492676	8.31E-12	15.66700328
RP11-705C15.2	-0.351577238	1.70149668	-6.998777204	8.41E-12	15.6560759
COL1A1	-0.946778055	7.654327923	-6.998403834	8.43E-12	15.65369787
TCN2	-0.504110309	4.125982861	-6.99604748	8.56E-12	15.63869238
PRPF40B	0.235650001	1.077521093	6.995394868	8.59E-12	15.63453721
RELB	-0.382877559	3.320846465	-6.995017	8.61E-12	15.63213148
ERAP2	-0.790364014	2.608569129	-6.993499198	8.70E-12	15.62246934
VASN	-0.633350668	3.748564418	-6.991140224	8.83E-12	15.6074558
FAM65A	-0.388981093	3.127233754	-6.990998189	8.84E-12	15.60655196
PAX5	-0.362351894	0.589410727	-6.989821114	8.91E-12	15.59906223
HCG4P11	-0.206322575	0.382124623	-6.987697813	9.03E-12	15.58555427
HSPA7	-0.636472185	2.129177081	-6.987100451	9.07E-12	15.58175459

VSTM1	-0.208412302	0.291815654	-6.981828572	9.38E-12	15.548233
GRN	-0.372284039	7.445910374	-6.981717034	9.39E-12	15.547524
PPT1	-0.355608628	6.001479463	-6.981653999	9.39E-12	15.54712333
S1PR2	-0.323329625	2.674613122	-6.977569068	9.65E-12	15.52116383
IGLV2-5	-0.541169125	0.992970514	-6.977382951	9.66E-12	15.51998137
RRS1	0.415376151	3.992329973	6.975435745	9.78E-12	15.50761166
SETD6	0.263749818	1.974251872	6.973398719	9.91E-12	15.49467439
KLF2	-0.594147155	3.439776663	-6.971028182	1.01E-11	15.47962289
SESN1	-0.351692087	2.172846772	-6.970460433	1.01E-11	15.47601864
SH3BP1	-0.339142125	2.225204896	-6.96834726	1.02E-11	15.46260568
ADAMTSL2	-0.510544638	2.226938416	-6.968230271	1.02E-11	15.46186322
PARP12	-0.357351813	3.318036322	-6.965678806	1.04E-11	15.44567296
S100A3	-0.439854952	1.309917691	-6.964895001	1.05E-11	15.44070033
IGHV1-24	-1.35178899	5.289030212	-6.960427634	1.08E-11	15.41236713
TLN1	-0.364599084	5.208713307	-6.956325663	1.11E-11	15.38636452
IGLV3-13	-0.403310753	0.596008223	-6.955508628	1.11E-11	15.3811868
NETO1	-0.240388688	0.293418672	-6.954136704	1.12E-11	15.37249375
IGKV1OR22-1	-0.683798809	1.205409537	-6.953572522	1.13E-11	15.36891928
TRAF4	0.360785414	4.652174207	6.949193346	1.16E-11	15.34118239
RP3-325F22.5	0.310567033	0.680526736	6.94903785	1.16E-11	15.34019777
OTX1	0.377282493	1.105015096	6.946036999	1.18E-11	15.3211996
GRASP	-0.421886292	1.907438033	-6.945163209	1.19E-11	15.31566896
N4BP1	-0.247386409	3.228903799	-6.943278974	1.20E-11	15.30374468
G0S2	-0.860537158	4.358772471	-6.943143251	1.21E-11	15.30288587
GMCL1	0.253037761	3.157897143	6.941074879	1.22E-11	15.28979956
RP11-673E1.3	-0.219539162	0.460495273	-6.940532896	1.23E-11	15.28637104
MPHOSPH10	0.211700356	3.311387608	6.94024565	1.23E-11	15.28455404
TACC1	-0.459639625	3.022248468	-6.940037592	1.23E-11	15.283238
LINC00944	-0.244210405	0.442455425	-6.934492952	1.27E-11	15.24817796
FRZB	-0.570241986	2.386261106	-6.934457788	1.27E-11	15.24795569
AC096579.13	-0.269330508	0.432042596	-6.933188422	1.28E-11	15.23993247
RHBDD3	0.351803828	3.114595749	6.933080868	1.29E-11	15.23925271
DANCR	0.453618741	3.521617231	6.932353578	1.29E-11	15.23465637

UNC13D	-0.615467482	2.995778757	-6.928796518	1.32E-11	15.21218212
PCDH12	-0.355665144	1.832398313	-6.927606714	1.33E-11	15.20466681
RGCC	-0.536483629	5.241389482	-6.927223391	1.34E-11	15.2022458
LRPPRC	0.298081521	4.056391538	6.927070438	1.34E-11	15.2012798
BYSL	0.374815492	3.621213619	6.926246135	1.34E-11	15.19607411
CX3CL1	-0.859628026	3.557757168	-6.925388841	1.35E-11	15.19066061
AFAP1L2	-0.539352838	1.958012779	-6.924053472	1.36E-11	15.18222933
TNFSF18	-0.207673752	0.436092702	-6.917439158	1.42E-11	15.14048752
CLUH	0.37426815	3.664036851	6.914981391	1.44E-11	15.12498534
DCHS1	-0.390415362	1.683118399	-6.91462224	1.45E-11	15.1227204
1-Mar	0.525332211	1.57869471	6.913962517	1.45E-11	15.1185602
OSBPL8	-0.315384984	3.071042891	-6.912048458	1.47E-11	15.10649203
SYNC	-0.218544833	0.695153512	-6.909768489	1.49E-11	15.09212039
PXN-AS1	0.237265376	1.259893942	6.906796464	1.52E-11	15.07339228
IGHVIII-51-1	-0.251196687	0.335234663	-6.90624213	1.53E-11	15.0698999
GALC	-0.342930011	2.784670217	-6.905084111	1.54E-11	15.06260496
IGHG3	-1.190669152	7.590767077	-6.903508443	1.56E-11	15.05268067
MORC3	-0.241255879	2.558716118	-6.89918582	1.60E-11	15.02546437
MOSPD2	-0.250339098	2.123546251	-6.89606153	1.63E-11	15.00580182
CCDC88B	-0.431470787	2.006990345	-6.894110553	1.65E-11	14.99352717
SKAP2	-0.414133473	3.58006395	-6.893844678	1.66E-11	14.99185463
RP11-465N4.4	0.362323898	1.714674468	6.892354111	1.67E-11	14.9824789
AGRP	-0.323375911	0.586559454	-6.886176922	1.74E-11	14.94364196
IGKV2D-28	-0.64361593	0.823460839	-6.882455915	1.78E-11	14.92026129
RRAD	-0.8950507	3.377252591	-6.878745165	1.82E-11	14.89695545
SLC25A46	-0.280348367	2.63527875	-6.878002782	1.83E-11	14.89229407
IGHV4-4	-1.043022388	2.534728383	-6.877481459	1.84E-11	14.88902095
HLA-DRB9	-0.361874105	0.886276581	-6.877438751	1.84E-11	14.88875282
ANKRD33B	-0.25111445	0.474001858	-6.876798657	1.85E-11	14.88473431
SNX3	-0.299101334	6.05797517	-6.870466532	1.92E-11	14.84499785
ANGPT1	-0.431132573	1.203529304	-6.868889623	1.94E-11	14.83510686
PDCL3	0.269731405	3.7673922	6.868122686	1.95E-11	14.83029701
CSF2	-0.627325932	1.345401384	-6.867427732	1.96E-11	14.82593898

ICAM1	-0.758889852	6.082233247	-6.865752509	1.98E-11	14.81543523
IGHD2-2	-0.643595694	0.989816269	-6.863503653	2.01E-11	14.80133809
TBC1D1	-0.384032306	2.402435843	-6.860762882	2.05E-11	14.7841625
IGKV1OR10-1	-0.345006234	0.540370928	-6.860699235	2.05E-11	14.78376371
PCAT6	0.515235889	2.495952776	6.859510232	2.06E-11	14.77631441
FAM110D	-0.268818067	0.844733777	-6.856992167	2.10E-11	14.76054183
STX7	-0.219992694	2.256774448	-6.856471384	2.10E-11	14.75728036
GMEB2	0.270869786	2.674196684	6.852673779	2.15E-11	14.73350359
CYTL1	-0.430813127	0.975584568	-6.851472429	2.17E-11	14.72598422
F8	-0.295015816	1.409791011	-6.84710413	2.23E-11	14.69865177
IGHG4	-1.315656198	7.049065058	-6.841770739	2.31E-11	14.66530029
PARP8	-0.287259267	2.238885207	-6.839917273	2.34E-11	14.65371498
C19orf45	0.218011058	0.325625861	6.839658094	2.34E-11	14.65209516
SNRPE	0.329696206	5.134647075	6.838842786	2.35E-11	14.64699996
LAMB1	-0.517793886	4.088714036	-6.837356012	2.38E-11	14.63770979
VAMP3	-0.239066354	5.353901291	-6.834609237	2.42E-11	14.62055085
FAM83H-AS1	0.515347228	3.579962725	6.832766401	2.45E-11	14.60904195
EPN3	0.502544851	2.248889542	6.83101218	2.47E-11	14.59808887
SOX12	0.447657576	2.954950641	6.828206543	2.52E-11	14.58057574
GLI1	-0.263247765	0.561935144	-6.824686436	2.58E-11	14.55861124
CREB3L4	0.433302713	3.247013587	6.821152693	2.63E-11	14.53657108
IGHVIII-67-2	-0.44543405	0.55203561	-6.819366107	2.66E-11	14.52543164
NUB1	-0.246309373	4.122994009	-6.818708433	2.68E-11	14.52133162
IGLV3-12	-0.693243932	1.322447788	-6.817655944	2.69E-11	14.51477096
PDE2A	-0.263937203	0.846750344	-6.815764251	2.73E-11	14.50298126
DDX27	0.290825572	3.833235139	6.813838038	2.76E-11	14.4909792
TMEM255B	-0.253123921	0.944685588	-6.813805401	2.76E-11	14.49077587
RGL3	0.52760555	2.746539782	6.812700609	2.78E-11	14.48389331
CD1A	-0.848605976	1.664552261	-6.812237959	2.79E-11	14.4810114
HHEX	-0.373020214	2.08002871	-6.812185903	2.79E-11	14.48068714
CTD-2319I12.2	-0.222818899	0.699043714	-6.812122993	2.79E-11	14.48029528
P2RX5	-0.280102614	0.626872351	-6.809379164	2.84E-11	14.46320715
IGKV2-26	-0.479437779	0.691972465	-6.804071	2.94E-11	14.43016496

TPD52	0.450291515	3.7983584	6.801495514	2.98E-11	14.4141408
C14orf93	0.203707306	1.871176796	6.800838788	3.00E-11	14.41005559
RP11-876N24.4	-0.244798194	0.818053437	-6.800650488	3.00E-11	14.40888431
CHTOP	0.234928944	4.180462129	6.794541043	3.12E-11	14.37089661
SORBS1	-0.375902939	1.594721132	-6.790300221	3.20E-11	14.34454441
IGHV3OR16-11	-0.431634653	0.603413533	-6.788431001	3.24E-11	14.33293353
SLC2A3	-0.623823028	3.160499798	-6.788299924	3.25E-11	14.33211943
AC133528.2	0.381261198	3.12179364	6.787280437	3.27E-11	14.32578798
HNMT	-0.470517905	3.446755582	-6.787182933	3.27E-11	14.32518249
ID2	-0.511774278	4.191295645	-6.786653421	3.28E-11	14.32189434
WDR74	0.230040088	2.916067597	6.78640614	3.28E-11	14.32035886
LIPT2	0.26959227	1.268586929	6.785540594	3.30E-11	14.31498465
FRMD4B	-0.304285772	1.977522442	-6.785198967	3.31E-11	14.31286363
TLR3	-0.411510254	1.670425309	-6.783872271	3.34E-11	14.30462756
PIM1	-0.392360082	4.266878117	-6.780217402	3.42E-11	14.28194523
DTHD1	-0.340288625	0.438677988	-6.779075509	3.44E-11	14.27486066
AC112721.2	-0.253229393	0.341518474	-6.778186631	3.46E-11	14.26934653
KCNJ10	-0.241586555	0.354859406	-6.777773832	3.47E-11	14.26678595
NOTCH2	-0.443736433	3.384928987	-6.776659947	3.49E-11	14.2598772
FAM198A	-0.203593543	0.52605089	-6.776559772	3.50E-11	14.25925592
THPO	0.556671834	0.857271309	6.772061584	3.60E-11	14.23136634
EHD1	-0.369949039	3.635430994	-6.769808056	3.65E-11	14.21739984
PNPLA6	-0.316656951	3.365258133	-6.767852525	3.69E-11	14.20528334
RP2	-0.291969762	2.784710245	-6.76683287	3.72E-11	14.19896669
TKT	0.424239869	5.813937729	6.766694845	3.72E-11	14.19811171
S100A4	-0.74741775	6.928383455	-6.763933809	3.79E-11	14.18101166
RP11-20G13.3	-0.229767859	0.31050444	-6.763293475	3.80E-11	14.17704668
IGHV4OR15-8	-0.319044715	0.45674211	-6.761518886	3.84E-11	14.16605997
AGPAT4	-0.263883099	0.963160229	-6.759077283	3.90E-11	14.15094761
PTPRD	-0.267091955	0.6613998	-6.757275095	3.95E-11	14.13979583
MCAT	0.239793533	2.873437338	6.756223398	3.98E-11	14.13328917
TMEM173	-0.510355054	4.742482618	-6.756112681	3.98E-11	14.13260423
PCDH17	-0.377142561	1.679320795	-6.75384284	4.04E-11	14.11856419

ZMYND19	0.299599204	3.167930373	6.753137285	4.05E-11	14.11420079
STS	-0.511276816	2.737831076	-6.7527064	4.07E-11	14.11153624
NEDD4L	0.45186716	2.847487181	6.75028389	4.13E-11	14.09655828
IGLJ2	-0.563380349	1.268727684	-6.748905031	4.16E-11	14.08803503
ACTR3B	0.214772623	1.139552737	6.746854722	4.22E-11	14.07536398
SEMA6B	-0.386719099	1.767688245	-6.746441692	4.23E-11	14.07281181
MAX	-0.208591433	3.942005596	-6.746414599	4.23E-11	14.0726444
GCSAM	-0.205216876	0.738263593	-6.746041704	4.24E-11	14.07034036
LEPROT	-0.253153305	4.685201907	-6.74577207	4.25E-11	14.0686744
RP11-353N14.2	0.371584067	0.812624697	6.744525531	4.28E-11	14.0609733
IFI44	-0.720919881	3.919612191	-6.744483265	4.28E-11	14.0607122
FHL3	-0.344706694	3.576739666	-6.743439422	4.31E-11	14.05426429
CLDN7	0.455823318	5.938220023	6.743404057	4.31E-11	14.05404585
EHMT2	0.328265703	3.515246308	6.739869593	4.41E-11	14.03221942
DFNA5	-0.40911993	1.293902306	-6.739354563	4.42E-11	14.02903975
IGHV1-14	-0.604063275	1.085233644	-6.734000494	4.57E-11	13.99599693
TYMP	-0.51238589	5.342479278	-6.732150818	4.63E-11	13.98458666
NXPH3	-0.207471467	0.515876296	-6.726657647	4.79E-11	13.95071584
EPYC	-0.453726079	0.493987443	-6.726051896	4.81E-11	13.9469822
KLHL4	-0.210628906	0.307166777	-6.722299612	4.92E-11	13.92386063
CNPY2	0.314623819	3.605616408	6.720320082	4.99E-11	13.9116671
CDH13	-0.319908134	0.918950628	-6.719203317	5.02E-11	13.90478935
DXO	0.319842251	2.843117357	6.7125123	5.24E-11	13.8636018
MLF2	0.300249065	5.646519169	6.709165394	5.35E-11	13.84301226
NID2	-0.448807725	1.543300192	-6.707972942	5.39E-11	13.83567858
PCNX	-0.260448308	2.018901191	-6.702781744	5.57E-11	13.80376495
CTD-2033A16.3	0.253888766	0.569114129	6.694602433	5.86E-11	13.75352328
PAXIP1-AS2	-0.242112061	1.430070711	-6.693564748	5.90E-11	13.74715292
KRTCAP3	0.591051579	4.978813632	6.693480284	5.90E-11	13.74663444
GP1BA	-0.243641239	0.641491768	-6.692143499	5.95E-11	13.7384292
PTX3	-0.413900592	0.946029501	-6.691781936	5.96E-11	13.73621016
TMTC4	0.341248768	2.448979294	6.690929156	6.00E-11	13.73097671
RP11-713C5.1	-0.224707819	0.283445018	-6.686995667	6.15E-11	13.70684441

ATOX1	-0.253343987	2.941253276	-6.686741781	6.16E-11	13.7052872
FIG4	-0.246152223	2.711797416	-6.686218916	6.18E-11	13.70208037
MAP3K1	-0.302962314	2.523360223	-6.686102243	6.18E-11	13.70136482
RAB40B	0.354520401	2.00656147	6.683788422	6.27E-11	13.68717644
LSM14B	0.315969264	3.99744366	6.683102696	6.30E-11	13.68297235
KCTD17	-0.365705598	2.587736816	-6.682424497	6.32E-11	13.67881475
PUF60	0.31614005	4.885343191	6.681662392	6.35E-11	13.67414321
CYTH1	-0.296029895	3.545705399	-6.679916731	6.42E-11	13.66344435
RELL1	-0.38286835	1.645856309	-6.67859431	6.48E-11	13.65534101
HLA-J	-0.554287951	1.857627647	-6.673340959	6.69E-11	13.62316351
VAX2	0.376210707	0.874077503	6.672536487	6.73E-11	13.61823787
ACTA2-AS1	-0.216642184	0.539321146	-6.670956773	6.79E-11	13.60856701
BBS12	-0.21119331	1.299637085	-6.667500771	6.94E-11	13.58741637
TSACC	0.281986639	0.749327579	6.665705003	7.02E-11	13.57642993
ANKRD36BP2	-0.392338593	0.713145504	-6.664215332	7.09E-11	13.56731805
CHN1	-0.392998722	1.984617214	-6.662085522	7.18E-11	13.55429359
PDGFC	-0.441043066	2.809708604	-6.661861023	7.19E-11	13.55292092
HGH1	0.348177725	3.742090521	6.660886569	7.24E-11	13.54696315
GNPTAB	-0.285215067	3.155171325	-6.659303108	7.31E-11	13.5372835
PMF1	0.338847711	3.85371696	6.658871091	7.33E-11	13.53464292
RPL7L1	0.248002648	3.965643465	6.656872538	7.42E-11	13.52242923
CYC1	0.423769926	5.758076909	6.656017829	7.46E-11	13.51720681
AKT3	-0.392734034	1.671686542	-6.655495119	7.48E-11	13.51401324
COQ9	0.269192287	3.643254828	6.653303792	7.59E-11	13.5006273
IER3IP1	0.333177572	4.054006851	6.653176896	7.59E-11	13.49985226
FAM198B	-0.553249442	2.656418571	-6.649817617	7.75E-11	13.47933924
IGHV3OR16-12	-0.38585963	0.647764781	-6.649628526	7.76E-11	13.47818484
C10orf55	-0.274295538	0.576084241	-6.648823685	7.80E-11	13.47327158
PID1	-0.558626672	2.206423053	-6.647872012	7.85E-11	13.46746261
SDCBP	-0.307977562	5.465220656	-6.647496739	7.87E-11	13.46517215
GJA4	-0.421099398	2.48205468	-6.647233124	7.88E-11	13.46356325
MARVELD3	0.3185082	1.975745139	6.646919903	7.90E-11	13.46165167
DES	-0.724317748	1.928124216	-6.646719554	7.91E-11	13.46042898

IGKV1D-33	-0.647781753	0.857891084	-6.646016571	7.94E-11	13.45613907
RP11-148B3.2	0.584149124	0.646011237	6.63993164	8.25E-11	13.41902203
RP11-27N21.3	0.365482648	0.963457403	6.639339493	8.28E-11	13.41541155
UACA	-0.351618766	2.143947426	-6.637969293	8.35E-11	13.40705811
DCP2	-0.258607754	2.08392936	-6.637355299	8.38E-11	13.40331535
MCHR1	-0.206213626	0.35720011	-6.635099429	8.50E-11	13.38956662
NDUFS1	0.238273835	3.102337648	6.630297208	8.76E-11	13.36031181
SLC39A8	-0.562989527	3.97477	-6.624820036	9.06E-11	13.32696688
IGHA2	-1.131856295	7.202908233	-6.621017776	9.28E-11	13.30383235
ABCA8	-0.346131662	0.699950839	-6.620174111	9.33E-11	13.29870065
FUCA1	-0.43258395	5.737394016	-6.618690952	9.41E-11	13.28968048
FERMT2	-0.336264766	2.617036525	-6.618424467	9.43E-11	13.28805997
ABLIM3	-0.462678669	1.603693605	-6.617569476	9.48E-11	13.28286109
WRNIP1	0.253216796	4.077124031	6.617148351	9.50E-11	13.28030059
CCAR1	0.23090012	3.712038277	6.615788171	9.58E-11	13.27203144
UGDH	0.675226972	4.888623227	6.613798667	9.70E-11	13.25993891
ALPK1	-0.237990744	1.669186883	-6.613460533	9.72E-11	13.25788398
RETN	-0.732111373	1.570184802	-6.613403783	9.73E-11	13.25753911
GTPBP4	0.329973115	3.361161196	6.613248833	9.74E-11	13.25659747
CCDC3	-0.512204863	2.429997487	-6.6132171	9.74E-11	13.25640463
IGKV1OR2-118	-0.232058391	0.338470335	-6.61155514	9.84E-11	13.24630605
ZMYM6NB	-0.327480962	2.91216958	-6.609688062	9.95E-11	13.23496364
ARHGAP39	0.383189601	2.167484081	6.607160224	1.01E-10	13.21961143
RP11-314A20.2	0.205422175	0.434111129	6.606210667	1.02E-10	13.2138458
MRPL48	0.289593602	2.574944584	6.604810363	1.03E-10	13.20534453
CLEC14A	-0.454580773	3.357062663	-6.604648148	1.03E-10	13.20435982
NT5C	0.328616117	3.910844992	6.603208815	1.04E-10	13.19562337
CYP7B1	-0.424149545	1.756961607	-6.602703892	1.04E-10	13.19255896
PLK3	-0.330809389	2.780061513	-6.6019398	1.04E-10	13.18792203
GBP3	-0.659350262	3.314203431	-6.597474735	1.07E-10	13.16083452
EXOSC4	0.390771705	4.028195766	6.597255589	1.08E-10	13.15950546
RAP2B	-0.355533364	3.667914032	-6.594425645	1.09E-10	13.14234592
MEOX1	-0.266095041	0.468159141	-6.593316776	1.10E-10	13.13562391

CDH5	-0.445926255	2.94865313	-6.593299444	1.10E-10	13.13551884
CAST	-0.303573021	4.400163296	-6.593282994	1.10E-10	13.13541913
RP11-26J3.3	0.297439804	1.130719596	6.590247588	1.12E-10	13.1170233
CD320	0.415810253	4.015114868	6.58762697	1.14E-10	13.10114697
IGHJ2P	-0.427546133	0.592117224	-6.587212127	1.14E-10	13.09863424
AL122127.25	-0.239294512	0.35347824	-6.586911967	1.15E-10	13.09681623
MFSD9	0.266483671	2.382014572	6.579379382	1.20E-10	13.05121569
NPR3	-0.430056121	1.419670315	-6.57904787	1.20E-10	13.0492098
PADI2	-0.520762881	1.538898335	-6.578727655	1.21E-10	13.04727234
COA4	0.303073762	4.474751324	6.57773488	1.21E-10	13.04126608
IGHV3-73	-1.278367561	4.397344178	-6.57772469	1.21E-10	13.04120443
TLCD1	0.462318758	4.001789314	6.577625599	1.21E-10	13.04060497
LSM4	0.31014658	4.800517631	6.57683868	1.22E-10	13.03584474
CTD-2288O8.1	-0.215324535	0.33668975	-6.574466422	1.24E-10	13.02149736
CTD-2020K17.1	-0.413188483	1.133714047	-6.573407952	1.25E-10	13.01509715
CYB5R4	-0.241281733	1.7650331	-6.573056765	1.25E-10	13.01297383
METRNL	-0.438687893	3.72755823	-6.5726533	1.25E-10	13.01053455
RAMP3	-0.504755339	3.131812419	-6.572274216	1.25E-10	13.00824279
MAMDC2	-0.562573536	1.518617285	-6.569907339	1.27E-10	12.99393628
RP11-283G6.4	-0.223949157	0.598215958	-6.56844028	1.29E-10	12.98507087
ITM2B	-0.343576449	5.822466413	-6.565516522	1.31E-10	12.96740762
TALDO1	0.443223168	6.243689995	6.564735772	1.31E-10	12.96269199
ACTB	-0.283591049	10.76125893	-6.564320978	1.32E-10	12.96018689
TMEM177	0.248951298	2.450775851	6.564054006	1.32E-10	12.95857462
CELF2	-0.485529217	2.919451966	-6.563621917	1.32E-10	12.95596529
RP11-335K5.3	0.233941859	0.23571825	6.562777095	1.33E-10	12.95086394
TFB2M	0.306346461	3.797486605	6.56153574	1.34E-10	12.94336919
IGKV2D-30	-0.594358104	0.908006033	-6.560651563	1.35E-10	12.93803164
CAMSAP3	0.390557883	2.85060184	6.557175408	1.38E-10	12.91705286
MAST3	-0.271073299	1.945079344	-6.556434101	1.38E-10	12.91258024
HRH1	-0.31220554	1.76926212	-6.554972175	1.40E-10	12.90376107
ALDH18A1	0.31054144	4.924266162	6.554821038	1.40E-10	12.90284942
BNIP2	-0.235661449	3.02025572	-6.554647894	1.40E-10	12.90180504

CPXM2	-0.660918611	2.132979582	-6.554000514	1.40E-10	12.89790037
PHLDB2	-0.418741542	1.499733266	-6.55241049	1.42E-10	12.88831151
PTCD1	0.200984495	1.256336547	6.551239926	1.43E-10	12.88125351
CASQ2	-0.235001536	0.382651501	-6.549195685	1.45E-10	12.86893015
IGKV1-27	-1.12505419	4.93920293	-6.547715971	1.46E-10	12.86001197
TAPBPL	-0.416065234	3.64144392	-6.547704582	1.46E-10	12.85994334
BAG6	0.266753188	5.738405166	6.547642787	1.46E-10	12.85957094
MANEA	-0.3076659	2.265892277	-6.547205857	1.46E-10	12.85693792
FNBP1L	0.337482303	3.956176074	6.546617972	1.47E-10	12.85339546
PEMT	0.358662581	3.391948536	6.546356478	1.47E-10	12.85181984
RP11-799B12.4	0.365574593	0.720534347	6.546198224	1.47E-10	12.85086632
HRH2	-0.26347164	0.580043046	-6.546147569	1.47E-10	12.85056111
THBD	-0.541141241	3.193855753	-6.544202535	1.49E-10	12.8388434
THEM6	0.438785854	3.819012595	6.538426484	1.55E-10	12.80406327
TRUB2	0.239216086	2.902060772	6.53583475	1.57E-10	12.7884657
RP11-148O21.4	-0.207193295	0.240175103	-6.5302872	1.63E-10	12.75509693
TXK	-0.202668426	0.556984136	-6.5257666	1.67E-10	12.72792295
ATP8B1	0.481166946	3.420771006	6.525482452	1.67E-10	12.72621542
MB21D2	-0.368684252	1.492883827	-6.523890949	1.69E-10	12.71665278
RP11-294C11.4	-0.399784914	0.480981423	-6.523852664	1.69E-10	12.71642277
RP11-295G20.2	0.651910329	2.627511724	6.523249833	1.70E-10	12.71280116
HLA-F-AS1	-0.235523414	0.671219616	-6.522965094	1.70E-10	12.71109064
MAPK11	-0.404740461	2.053671569	-6.522825829	1.70E-10	12.71025405
RPP21	0.280540802	2.491273352	6.522778852	1.70E-10	12.70997185
ZFP36L1	-0.474755222	6.437567747	-6.522266772	1.71E-10	12.70689585
CCHCR1	0.354518223	2.760302864	6.521970652	1.71E-10	12.70511718
SKAP1	-0.551869577	2.144748326	-6.517622172	1.76E-10	12.67900559
SNHG17	0.376680502	2.517238646	6.517265041	1.76E-10	12.67686175
MIR6774	-0.271480151	0.36832561	-6.515217013	1.78E-10	12.66456947
IGKV2OR22-3	-0.497405581	0.765164578	-6.514656201	1.79E-10	12.66120404
NOP56	0.329406101	4.178371619	6.51445733	1.79E-10	12.66001067
AAMP	0.203827132	5.287157896	6.5136811	1.80E-10	12.65535304
GTF3C2	0.219179035	3.722751845	6.513251111	1.80E-10	12.65277317

DUSP3	-0.256998745	4.278498568	-6.511266276	1.83E-10	12.64086631
RCAN1	-0.366569212	2.531244475	-6.509273558	1.85E-10	12.62891523
GKAP1	0.244446603	1.235248587	6.508165069	1.86E-10	12.62226854
PIR	0.620744762	2.801054054	6.506402212	1.88E-10	12.61170012
TACC2	0.582650236	2.637883704	6.505830031	1.89E-10	12.60827038
MCUR1	0.244702132	3.083085479	6.505505436	1.89E-10	12.60632482
MLKL	-0.330610408	2.144269724	-6.503594864	1.92E-10	12.59487489
ARMCX1	-0.408534415	2.6168085	-6.502615231	1.93E-10	12.58900511
FBLN5	-0.555757716	3.120536407	-6.501221023	1.94E-10	12.58065256
TAPBP	-0.355437707	6.138054357	-6.49860821	1.97E-10	12.56500355
AIFM2	0.372194872	2.795787953	6.495035782	2.02E-10	12.54361566
TRAJ38	-0.203595114	0.187135319	-6.494919543	2.02E-10	12.54291991
MTPAP	0.21421082	2.002316574	6.492852023	2.05E-10	12.53054652
NMI	-0.338036159	3.238990004	-6.492257556	2.05E-10	12.52698946
RP11-524D16__A.3	-0.50800739	1.732451735	-6.488487305	2.10E-10	12.50443611
OLFML3	-0.65830545	3.968219767	-6.487025874	2.12E-10	12.49569692
2-Mar	-0.265938876	4.006574563	-6.486074306	2.13E-10	12.49000754
HMBS	0.318219916	2.958604358	6.483216225	2.17E-10	12.47292345
MAOB	-0.59220698	2.585916165	-6.481242071	2.20E-10	12.46112672
AHR	-0.540685781	4.856997019	-6.48097805	2.20E-10	12.45954926
LTBP4	-0.513494059	3.519665479	-6.479863464	2.21E-10	12.45289052
SYTL2	-0.462012118	2.169726867	-6.479536373	2.22E-10	12.45093661
RP11-493L12.5	-0.33282579	0.750459539	-6.479099687	2.22E-10	12.44832814
CDK2AP2	0.460802858	5.635411723	6.479061354	2.23E-10	12.44809917
IRF2BP1	0.308370746	3.502919932	6.478683125	2.23E-10	12.44584002
CBR1	0.758831986	5.200115021	6.477141874	2.25E-10	12.43663531
TMEM176B	-0.630388743	5.75399191	-6.476311137	2.26E-10	12.43167472
AC246787.1	-0.646986737	1.199388143	-6.475372957	2.28E-10	12.4260732
TLE4	-0.320407249	1.599499857	-6.474362293	2.29E-10	12.42003967
APITD1	0.271558286	1.718138133	6.473297851	2.31E-10	12.41368596
C19orf48	0.414052705	3.783638649	6.46873561	2.37E-10	12.38646367
IGKV1D-12	-0.718664782	1.373359031	-6.468137368	2.38E-10	12.38289525
OASL	-0.54778826	2.033704692	-6.466351915	2.40E-10	12.37224693

IGLV2-34	-0.604176328	1.286321687	-6.464907396	2.43E-10	12.36363374
OSBPL11	-0.235666267	2.771115059	-6.459199687	2.51E-10	12.32961644
SLC9A6	-0.255056119	2.522719719	-6.455937016	2.56E-10	12.31018269
FLAD1	0.321676132	3.847236399	6.455869177	2.56E-10	12.3097787
FAM46C	-0.545402999	3.35050453	-6.455783461	2.56E-10	12.30926826
CTD-2547L24.4	-0.253102613	0.602658856	-6.454775331	2.58E-10	12.30326524
SHARPIN	0.33912909	4.863022616	6.454643427	2.58E-10	12.30247986
GYG2	0.480467413	2.025991222	6.453598021	2.60E-10	12.29625582
TNFSF14	-0.321107186	0.845739235	-6.452518699	2.62E-10	12.28983075
11-Sep	-0.392148052	3.500767198	-6.450868726	2.64E-10	12.28001041
RUVBL1	0.303451346	3.188512681	6.450111879	2.66E-10	12.27550651
SEC14L1	-0.348468928	3.096485173	-6.448908594	2.67E-10	12.26834683
RP11-320M2.1	0.273663227	0.770863177	6.447999839	2.69E-10	12.26294039
MKNK1	-0.218823768	2.41839918	-6.447314898	2.70E-10	12.2588659
ELN	-0.758486347	3.614728313	-6.447223295	2.70E-10	12.25832101
IGHV1OR16-3	-0.482078456	0.725789639	-6.44677079	2.71E-10	12.25562944
IGHV3-25	-0.392608337	0.665497741	-6.444439892	2.75E-10	12.24176743
ABCB1	-0.25379819	0.647954627	-6.442634127	2.78E-10	12.23103135
C10orf2	0.295761564	2.347115404	6.441741031	2.79E-10	12.22572242
TMEM52	0.404784876	1.261529891	6.441345129	2.80E-10	12.22336922
SLC25A30	-0.218244601	1.699108889	-6.440985938	2.81E-10	12.22123433
PHF11	-0.234959511	2.751229602	-6.440757236	2.81E-10	12.21987507
PVALB	-0.270255448	0.42707275	-6.440372039	2.82E-10	12.21758579
COL13A1	-0.318151171	0.984335242	-6.439204975	2.84E-10	12.21065048
AP000688.14	0.205063731	0.316771259	6.43833953	2.85E-10	12.20550824
LRRC17	-0.406336667	1.303879597	-6.432847424	2.95E-10	12.17288922
SERPINE1	-0.777934203	4.538856743	-6.431097802	2.98E-10	12.16250271
IGLJ1	-0.426610126	0.764530016	-6.430072142	3.00E-10	12.15641506
PATZ1	0.289831634	3.374765648	6.429655595	3.01E-10	12.15394294
WDR34	0.377315705	4.948284029	6.429572601	3.01E-10	12.15345041
ESRRA	0.311316063	4.09149198	6.423919046	3.11E-10	12.11991155
CNN3	-0.425377007	5.787380682	-6.422256279	3.14E-10	12.1100522
SWI5	0.273424692	3.505196351	6.422068123	3.15E-10	12.10893666

UBE2V2	0.27646153	3.188601752	6.420714877	3.17E-10	12.1009144
AC016831.7	0.350686902	0.563180497	6.419933679	3.19E-10	12.09628398
HRAS	0.316990654	3.836698959	6.417517954	3.24E-10	12.08196819
ARHGAP20	-0.397067653	1.01400017	-6.416886589	3.25E-10	12.07822743
IGFBP4	-0.465438688	7.568184579	-6.414880389	3.29E-10	12.06634298
MTM1	-0.264439975	2.313728196	-6.414590898	3.29E-10	12.06462833
MAL	-0.583587683	1.936713576	-6.413259321	3.32E-10	12.0567423
COPZ2	-0.44477706	2.546548382	-6.407426303	3.44E-10	12.02221358
SUPV3L1	0.212365861	2.763030565	6.404220953	3.51E-10	12.00325075
OLFM1	-0.476382786	1.26377703	-6.402240741	3.55E-10	11.99153984
PNPT1	0.297608401	3.104325078	6.401598172	3.56E-10	11.98774037
RNA5SP39	-0.205739149	0.345563297	-6.400177029	3.59E-10	11.97933839
SLC6A12	-0.242376035	0.628993809	-6.396069219	3.69E-10	11.95506136
DIO2	-0.498767493	1.249583463	-6.393513169	3.74E-10	11.93996186
NMB	0.54223832	3.084678654	6.393470613	3.74E-10	11.93971051
IGHV3OR16-15	-0.301429237	0.518522639	-6.390796401	3.80E-10	11.92391854
TSC22D3	-0.586188162	5.273088799	-6.390264265	3.82E-10	11.92077679
CR2	-0.623889645	1.353646425	-6.389809572	3.83E-10	11.91809245
IGKV2D-40	-1.021772195	2.6006521	-6.389774923	3.83E-10	11.9178879
LHX4-AS1	0.203930466	2.620281336	6.388751911	3.85E-10	11.91184903
PICK1	0.303666705	2.869545145	6.385722861	3.92E-10	11.89397326
CFD	-0.701436876	3.868102678	-6.38417875	3.96E-10	11.88486355
8-Sep	-0.254871139	2.890419719	-6.383408084	3.98E-10	11.88031758
FXR2	0.245465613	3.398524198	6.38288596	3.99E-10	11.87723797
NPR2	-0.279274398	1.640697834	-6.380569424	4.05E-10	11.86357706
RP11-798K23.5	-0.406581674	1.624014856	-6.376507589	4.15E-10	11.83963406
PANK1	0.245456682	1.021089802	6.376450509	4.15E-10	11.83929769
ROS1	-0.820590752	2.807974774	-6.376164295	4.16E-10	11.83761106
DPM2	0.293114351	4.36486117	6.375225771	4.18E-10	11.83208091
AC013264.2	-0.566461727	0.984660956	-6.372763235	4.24E-10	11.81757395
ITPR2	-0.383136197	2.04972753	-6.372227567	4.26E-10	11.81441892
MARVELD1	-0.425566305	4.130933433	-6.369724448	4.32E-10	11.79967881
ADAMTS16	-0.384108253	0.924674432	-6.369149077	4.34E-10	11.79629132

TRAJ2	-0.273712013	0.374189583	-6.368349373	4.36E-10	11.79158351
USP21	0.256831061	3.372282846	6.367926448	4.37E-10	11.78909397
RP11-480I12.5	0.451336075	1.477957066	6.366184962	4.41E-10	11.77884424
FAM212A	-0.290916623	1.326011674	-6.365747293	4.43E-10	11.77626866
ANXA1	-0.781383716	5.797547682	-6.365537869	4.43E-10	11.7750363
SRPX2	-0.707750265	3.250988568	-6.363265647	4.49E-10	11.7616676
MRPL30	0.233597504	3.503188603	6.362472896	4.51E-10	11.75700436
DOK1	-0.288424943	2.815126717	-6.362352704	4.52E-10	11.7562974
RP11-77A13.1	-0.396944694	0.39333358	-6.361262436	4.55E-10	11.74988498
PUSL1	0.319905438	2.79429066	6.359977034	4.58E-10	11.74232609
MACROD1	0.423637656	3.067738642	6.356311828	4.68E-10	11.7207797
SECTM1	-0.574466063	3.536114028	-6.352673994	4.79E-10	11.69940466
IGHV3-65	-0.340022276	0.470478196	-6.352629189	4.79E-10	11.69914146
BOP1	0.44538093	4.019201829	6.351497016	4.82E-10	11.69249125
NDUFS2	0.246255088	4.916337304	6.351451585	4.82E-10	11.69222441
CD59	-0.439380276	6.198073547	-6.349949931	4.87E-10	11.68340553
C14orf132	-0.404767768	1.415417278	-6.349343904	4.88E-10	11.67984696
MRPL2	0.272854666	3.638168537	6.349112947	4.89E-10	11.67849087
IGKV2-24	-1.05778449	4.351313937	-6.347019797	4.95E-10	11.66620259
CBX8	0.25986942	2.029405611	6.346252106	4.98E-10	11.66169656
STAT5B	-0.236875854	3.394847992	-6.345491996	5.00E-10	11.65723548
EBPL	0.367407837	4.237987667	6.344469009	5.03E-10	11.6512323
ITIH5	-0.404425802	1.000249928	-6.342937705	5.08E-10	11.6422477
MT2A	-0.719031744	6.113899113	-6.341879677	5.11E-10	11.63604103
ST6GALNAC6	-0.321857801	3.502458447	-6.340979795	5.14E-10	11.63076278
CLIP2	-0.384458904	1.906501161	-6.337309151	5.25E-10	11.60923922
ZNF696	0.25805311	1.60903558	6.334647328	5.34E-10	11.59363771
AC074117.10	0.272813847	1.416623148	6.331518056	5.44E-10	11.57530352
MYCBP2	-0.305895858	2.176185757	-6.330519977	5.47E-10	11.56945746
RP11-20G13.2	-0.246898046	0.339188251	-6.326533386	5.60E-10	11.54611458
ARHGEF39	0.26908008	1.048578286	6.323766378	5.69E-10	11.52992014
VOPP1	-0.36514191	3.459337876	-6.32315586	5.72E-10	11.52634777
WI2-1896O14.1	-0.382981127	1.460938484	-6.321476824	5.77E-10	11.51652464

TTYH2	-0.311660674	1.26778444	-6.320792069	5.80E-10	11.51251914
VCL	-0.351953352	3.932212476	-6.319239754	5.85E-10	11.50344019
RHOA	-0.205880655	7.714338151	-6.318026785	5.89E-10	11.49634728
IGHV1-3	-0.933418395	2.093255948	-6.317478113	5.91E-10	11.49313927
CTSD	-0.447683503	8.817517966	-6.316913096	5.93E-10	11.48983594
BACE1	-0.312783506	2.514769404	-6.313037215	6.07E-10	11.46718265
RAP1GAP2	0.437588457	2.255096996	6.31049216	6.17E-10	11.45231406
EIF2AK1	0.257419756	5.511425112	6.309871371	6.19E-10	11.44868809
BFSP1	0.249116473	0.970617882	6.307717271	6.27E-10	11.43610855
NMT2	-0.232079913	1.660713121	-6.3054489	6.35E-10	11.42286565
RASA2	-0.263213927	2.331897659	-6.305348496	6.36E-10	11.42227958
MRPS16	0.240080024	4.830074809	6.304031422	6.41E-10	11.41459238
SLC40A1	-0.617198107	5.181563343	-6.303205842	6.44E-10	11.40977451
MRPL24	0.342229988	5.213342239	6.299188504	6.60E-10	11.38633807
ROBO1	-0.410679921	1.959991456	-6.298521181	6.62E-10	11.38244626
DMXL1	-0.21479747	1.66135468	-6.297734592	6.65E-10	11.37785935
DDT	0.329002338	3.717492102	6.297575786	6.66E-10	11.37693334
STAU2	0.308130311	2.840062264	6.296777836	6.69E-10	11.37228077
ZNF469	-0.237124835	0.705301867	-6.296028436	6.72E-10	11.36791174
RIN2	-0.305336445	3.78605067	-6.295601185	6.74E-10	11.36542105
PTGER2	-0.479732655	1.602923505	-6.294335372	6.79E-10	11.35804274
AC244250.4	-0.727778969	1.217021494	-6.293336713	6.83E-10	11.35222254
IGHV7-56	-0.467420999	0.850741598	-6.291811674	6.89E-10	11.3433361
MIR4537	-0.556150281	0.973378391	-6.290918745	6.93E-10	11.33813384
CCDC24	0.353630168	2.410699156	6.290211214	6.96E-10	11.33401216
CNOT11	0.257236866	4.872033678	6.290108047	6.96E-10	11.3334112
FASN	0.61915112	5.016296641	6.288784925	7.02E-10	11.32570461
PDHA1	0.242927039	3.765307077	6.288233375	7.04E-10	11.32249248
ETV1	-0.502634869	2.128038448	-6.287329258	7.08E-10	11.31722759
JPH2	-0.20665758	0.495654196	-6.286739317	7.11E-10	11.31379257
HES6	0.751101267	2.894688067	6.286486724	7.12E-10	11.31232189
RASSF8	-0.429047429	1.861945825	-6.282444202	7.29E-10	11.2887919
IL13RA2	-0.483343009	0.728512885	-6.281942172	7.31E-10	11.28587068

METTL7A	-0.533763082	4.012325863	-6.281215903	7.34E-10	11.281645
RNF208	0.430534805	2.95328371	6.280721863	7.37E-10	11.27877074
DEGS1	-0.261382465	4.926829442	-6.275263206	7.61E-10	11.24702586
GLRX	-0.410198526	2.685610647	-6.274317504	7.65E-10	11.24152852
PI16	-0.31067279	0.436440585	-6.273903166	7.67E-10	11.2391202
IGKV7-3	-0.425524897	0.776405826	-6.27144455	7.78E-10	11.22483242
IFI44L	-0.630040467	2.012828535	-6.269724143	7.86E-10	11.21483745
C9orf72	-0.275157166	1.757576119	-6.266663868	8.01E-10	11.19706408
TBCE	0.274905423	3.710548983	6.265285405	8.07E-10	11.1890607
FRAT2	0.336875466	3.010340047	6.265129905	8.08E-10	11.18815796
SNRPA	0.229876193	4.63220995	6.264832985	8.10E-10	11.18643428
ITGA11	-0.604497459	1.96909731	-6.2643807	8.12E-10	11.18380879
PCAT19	-0.307754962	1.077221424	-6.264014833	8.14E-10	11.18168507
TRIM28	0.290717669	6.078085572	6.263986009	8.14E-10	11.18151777
DHCR7	0.403330518	4.153435061	6.263515346	8.16E-10	11.17878592
VPS52	0.236834427	3.760891542	6.259805524	8.34E-10	11.15725929
SF3B6	0.253870988	5.769140895	6.259008789	8.38E-10	11.15263758
YDJC	0.364686742	3.729157347	6.25893971	8.38E-10	11.15223689
MEIS1	-0.255478469	1.145739826	-6.256235862	8.52E-10	11.13655624
GPRASP1	-0.267581421	0.925131356	-6.255225367	8.57E-10	11.13069748
IGHD4-23	-0.428047305	0.47187415	-6.255165821	8.57E-10	11.13035226
FABP3	-0.794896648	3.414630159	-6.253602123	8.65E-10	11.12128774
SAA2	-0.705680231	1.327802294	-6.25297921	8.69E-10	11.11767735
RP11-686D22.8	-0.248504319	1.109763284	-6.252808486	8.70E-10	11.11668789
TM4SF18	-0.436221541	1.440821223	-6.252047795	8.74E-10	11.11227945
EFHD2	-0.413018183	5.437911305	-6.249117451	8.89E-10	11.09530152
ZNF620	0.200986592	1.053768782	6.248793809	8.91E-10	11.0934268
ROBO4	-0.37744054	1.862660055	-6.246836423	9.01E-10	11.08209031
THAP4	0.222664391	4.085001151	6.245784065	9.07E-10	11.07599668
ADCK5	0.393812535	2.535183811	6.245653351	9.07E-10	11.07523984
FOXN3	-0.317621016	2.577012978	-6.241028758	9.33E-10	11.04847221
MAPKAPK3	-0.335806851	4.107241393	-6.238106835	9.49E-10	11.03156854
LZTS3	0.473346625	3.34763236	6.237943709	9.50E-10	11.03062504

C11orf49	0.273256354	2.709100326	6.232488656	9.81E-10	10.99908572
ADAMTS1	-0.505591438	2.463199555	-6.23137309	9.87E-10	10.99263879
KLF10	-0.336478535	4.204318948	-6.228339031	1.01E-09	10.97510974
AC108463.2	-0.236115347	0.90269411	-6.225894246	1.02E-09	10.9609905
GCAT	0.426702	2.878210993	6.221245723	1.05E-09	10.93415718
FOXO1	-0.372165674	2.453178091	-6.220495919	1.05E-09	10.92983059
ZSCAN16	0.296357379	2.694477844	6.22019193	1.05E-09	10.92807662
DHTKD1	0.32306006	3.33362997	6.218472855	1.07E-09	10.91815914
ADCY4	-0.246763244	1.009222132	-6.218342221	1.07E-09	10.9174056
PAXIP1	0.213137376	1.636878451	6.21771153	1.07E-09	10.91376774
INMT	-0.706382526	2.563963061	-6.216764819	1.08E-09	10.90830767
PLA2R1	-0.226952723	0.917222043	-6.216753397	1.08E-09	10.9082418
CARKD	0.327125356	4.028984768	6.213365624	1.10E-09	10.88870896
YPEL2	-0.238262216	2.050960083	-6.211537415	1.11E-09	10.87817186
HSF1	0.288083858	4.319585831	6.208736741	1.13E-09	10.86203499
C12orf4	-0.222492938	2.221908025	-6.20797472	1.13E-09	10.85764547
PCCB	0.280105031	2.719924351	6.207675474	1.14E-09	10.85592183
PAFAH1B3	0.452918925	4.62569648	6.205854496	1.15E-09	10.84543463
FXYD4	0.781179492	1.042183758	6.203845262	1.16E-09	10.8338663
PA2G4	0.259938571	5.110610667	6.203467312	1.16E-09	10.83169058
GPR1	-0.221478432	0.313426561	-6.202214405	1.17E-09	10.82447886
RP11-517I3.2	-0.212461943	0.834670682	-6.20132518	1.18E-09	10.81936125
SYT7	0.74765119	3.11424363	6.201290469	1.18E-09	10.81916149
FAM13B	-0.26464048	2.147133025	-6.198608353	1.20E-09	10.80372943
RP11-251M1.1	-0.229937296	0.529314967	-6.196529363	1.21E-09	10.7917715
PPFIBP1	-0.325164473	2.200078459	-6.196141976	1.22E-09	10.78954371
SMG5	0.297506323	4.82911262	6.192856173	1.24E-09	10.77065244
MID2	-0.298634843	1.707326178	-6.190733036	1.26E-09	10.75845033
FARSB	0.252491797	3.875532674	6.189392379	1.27E-09	10.75074713
PEX13	0.209151792	3.266184666	6.188929255	1.27E-09	10.74808644
RABL6	0.267052866	3.431191695	6.18822961	1.27E-09	10.74406721
ZNF154	-0.275362099	0.899702062	-6.185506722	1.29E-09	10.72842887
FAM20C	-0.446616889	3.376132286	-6.185390423	1.30E-09	10.72776106

PTGES2	0.276041077	3.961660869	6.184551684	1.30E-09	10.72294521
HSBP1L1	0.36654876	3.296880069	6.18166723	1.32E-09	10.70638756
ABHD6	-0.238790458	1.62903097	-6.180875095	1.33E-09	10.70184162
DDX60L	-0.336328854	1.76210836	-6.180289317	1.33E-09	10.69848026
IGHV3-6	-0.335625176	0.50761793	-6.18009696	1.34E-09	10.69737652
LARP1B	0.200863038	1.853615207	6.179349872	1.34E-09	10.69309002
WDR12	0.231650659	2.070613227	6.178835639	1.35E-09	10.6901398
IGFBP6	-0.713972173	2.911893494	-6.174915065	1.38E-09	10.66765396
RBMS3	-0.3258318	1.266041479	-6.174179369	1.38E-09	10.66343585
ERP29	0.30678531	6.115033694	6.172553768	1.40E-09	10.65411701
WDFY1	-0.256937025	3.581736389	-6.17204111	1.40E-09	10.65117861
3-Mar	-0.253485583	0.999331785	-6.170699601	1.41E-09	10.64349047
SSB	0.228734454	4.330722501	6.169880674	1.42E-09	10.63879793
PDLIM4	-0.622529374	2.536994552	-6.16974325	1.42E-09	10.63801053
PYGO2	0.250891676	4.557688882	6.169641071	1.42E-09	10.63742509
TMED4	0.248369825	5.384580951	6.169108097	1.43E-09	10.63437148
PSMD4	0.275446744	5.813843865	6.168958866	1.43E-09	10.63351652
DCBLD1	-0.364567454	2.388096007	-6.166656224	1.45E-09	10.6203267
HS3ST3A1	-0.261877126	0.527893027	-6.162910021	1.48E-09	10.59887699
BPHL	0.291881294	2.152781191	6.160171934	1.50E-09	10.58320655
ACKR4	-0.248388412	0.490337934	-6.16006411	1.50E-09	10.58258958
HLA-DPB2	-0.453073016	1.202766222	-6.159513394	1.51E-09	10.57943853
LRP5	0.395471021	4.557962968	6.159315189	1.51E-09	10.57830451
POLK	-0.215210711	1.755637148	-6.156181508	1.54E-09	10.5603795
FABP4	-0.667394237	1.497035843	-6.155837036	1.54E-09	10.55840956
TMEM55A	-0.297710145	1.737035432	-6.155777588	1.54E-09	10.5580696
PLAUR	-0.542334155	4.135161107	-6.154057635	1.56E-09	10.54823516
CRLF3	-0.228513763	2.640561375	-6.152943434	1.57E-09	10.54186557
FAM102B	-0.314682982	1.671692811	-6.152433791	1.57E-09	10.53895241
RNF144B	-0.411314091	2.764429271	-6.146702865	1.63E-09	10.50620824
BEND6	-0.225836291	0.517264691	-6.143975323	1.65E-09	10.49063337
MT1M	-0.599877548	1.620117338	-6.143766595	1.65E-09	10.48944173
GPX8	-0.474047381	2.915788007	-6.141994631	1.67E-09	10.4793269

SLC28A3	-0.40749762	0.936394563	-6.140422633	1.69E-09	10.47035563
RBMS1	-0.243131947	3.000014522	-6.138086227	1.71E-09	10.45702558
DPY30	0.271936816	4.169839167	6.137792835	1.71E-09	10.45535197
ECE2	0.315816894	1.51470069	6.136308075	1.73E-09	10.44688349
RSU1	-0.226278275	4.237193164	-6.135064318	1.74E-09	10.43979094
MXRA7	-0.418624114	2.978692317	-6.134507299	1.75E-09	10.43661492
C10orf35	0.337770802	3.311878088	6.133342463	1.76E-09	10.42997407
DOK5	-0.415507641	0.989376034	-6.130286489	1.79E-09	10.41255676
MIS18A	0.334929693	3.041650011	6.129781147	1.79E-09	10.40967732
TMEM141	0.422525885	5.07964083	6.129649351	1.80E-09	10.40892638
XXyac-YX65C7_A.2	-0.216195764	0.643143334	-6.128843245	1.80E-09	10.40433369
TOP1MT	0.306787339	2.565331177	6.128165728	1.81E-09	10.40047402
TSPAN9	-0.357212483	3.207546921	-6.12735471	1.82E-09	10.39585431
ZNF692	0.435022762	3.01743678	6.127178784	1.82E-09	10.39485227
NOL3	0.389819094	3.780264925	6.126462492	1.83E-09	10.39077267
RP11-9E17.1	0.314755025	1.10536765	6.123627464	1.86E-09	10.37462996
CBFA2T3	-0.322556844	0.993352675	-6.122981259	1.87E-09	10.37095135
MFAP5	-0.558540227	1.054965316	-6.121881406	1.88E-09	10.36469106
EOGT	-0.249160461	2.400212621	-6.120513788	1.89E-09	10.35690802
SYNE1	-0.357727167	1.510512032	-6.116586342	1.94E-09	10.33456544
FHAD1	-0.239014318	0.501964734	-6.114292805	1.96E-09	10.32152359
RP11-566K19.6	0.337644475	0.539694745	6.113590206	1.97E-09	10.31752921
TOR1AIP1	-0.211511695	3.19644134	-6.111974028	1.99E-09	10.3083425
TMEM71	-0.331728051	1.0222563	-6.111317065	2.00E-09	10.30460877
HPN-AS1	0.21593416	0.501099241	6.111214875	2.00E-09	10.30402803
COPG1	0.241223286	6.029841608	6.108649555	2.03E-09	10.28945202
MOB1A	-0.20982575	4.75957986	-6.106057597	2.06E-09	10.27473
VAPA	0.286021639	4.165880605	6.105984398	2.06E-09	10.27431433
E2F6	0.212940812	2.142833657	6.103160113	2.10E-09	10.258279
DHRS13	0.250209121	1.507878942	6.101694987	2.11E-09	10.24996303
SLC39A7	0.307076481	6.384520103	6.100867673	2.12E-09	10.24526801
SEPP1	-0.588754348	3.842830607	-6.099125066	2.15E-09	10.23538047
CDK16	0.282008392	4.164802599	6.098642175	2.15E-09	10.23264097

LATS2	-0.271911256	2.514660446	-6.097465948	2.17E-09	10.2259689
SERTAD1	-0.356592889	3.608193581	-6.097075868	2.17E-09	10.22375643
BTBD19	-0.30934036	1.285173535	-6.096866941	2.17E-09	10.22257149
PTGES3P1	-0.292292486	3.035274932	-6.095523182	2.19E-09	10.2149511
TMEM254	0.289202388	3.443954063	6.093863707	2.21E-09	10.20554228
ACOT13	0.283170671	3.063937021	6.092607913	2.23E-09	10.19842371
IGHV7-27	-0.407591677	0.738649942	-6.09028425	2.26E-09	10.18525516
NUDT8	0.395759931	2.669661908	6.088453693	2.28E-09	10.17488417
FLT4	-0.323639669	1.618059253	-6.08760653	2.29E-09	10.17008548
IGKV1OR2-9	-0.287427702	0.434035595	-6.087056626	2.30E-09	10.16697091
AC104667.3	0.274327313	1.033440757	6.084138438	2.34E-09	10.15044676
GFRA1	-0.293127331	0.511017371	-6.082358189	2.37E-09	10.14036951
ACSL4	-0.448621996	3.847546361	-6.079426033	2.41E-09	10.12377733
PHACTR2	-0.312115955	2.007188557	-6.078507078	2.42E-09	10.11857866
CDC14A	-0.252355654	1.239885717	-6.077069423	2.44E-09	10.11044698
IL5RA	-0.237822248	0.339777389	-6.073370252	2.49E-09	10.08953132
TRAJ6	-0.225507201	0.279901803	-6.07335204	2.49E-09	10.08942837
CTD-2260A17.1	-0.204631827	0.787995063	-6.070246847	2.54E-09	10.07187966
RP3-406A7.7	0.405309255	2.013569822	6.070050893	2.54E-09	10.0707725
TIMM17B	0.330210567	4.244366818	6.068976064	2.56E-09	10.06470017
IFNAR1	-0.228768429	3.998276018	-6.068576615	2.56E-09	10.06244369
SDC2	-0.481112503	3.345408505	-6.068161821	2.57E-09	10.06010066
RP11-388C12.8	0.288644164	0.878455363	6.063708693	2.64E-09	10.03495516
IFIT5	-0.325952156	3.098845462	-6.063497238	2.64E-09	10.03376154
RP11-168F9.2	0.295873681	0.725217256	6.061287948	2.67E-09	10.0212926
IGHD3-22	-0.587611292	1.000046121	-6.059981785	2.69E-09	10.01392264
SNRPD1	0.302291497	3.338537918	6.059146521	2.71E-09	10.00921042
ITPA	0.293931377	4.526472535	6.058856318	2.71E-09	10.00757334
FGF1	-0.300397496	0.836116064	-6.05838282	2.72E-09	10.00490242
KIF21B	-0.289893317	1.160012535	-6.056398467	2.75E-09	9.993710973
PFDN2	0.328036368	6.251766247	6.056382923	2.75E-09	9.993623321
EPT1	0.343249051	2.853719665	6.056311828	2.75E-09	9.993222411
SUV420H1	0.212413334	2.969495399	6.05592977	2.76E-09	9.991068052

TGFBR3	-0.371205666	1.319169552	-6.054716817	2.78E-09	9.984229197
ELF4	-0.358249638	3.348696014	-6.054527894	2.78E-09	9.983164117
RP11-983P16.4	0.335229145	1.578751937	6.053679948	2.79E-09	9.978384071
C3orf80	-0.264095004	0.864979036	-6.053553125	2.80E-09	9.97766919
HEXB	-0.284598057	4.812579138	-6.052460688	2.81E-09	9.971511854
AC244157.1	-0.474163144	0.747715792	-6.052367675	2.81E-09	9.970987651
RP11-10A14.4	0.284324431	0.564532263	6.05027627	2.85E-09	9.959202632
PEAR1	-0.277535865	1.106575518	-6.05022041	2.85E-09	9.958887909
FZD5	0.355100151	2.830149613	6.049163806	2.87E-09	9.952935355
IGKV3OR2-5	-0.305097967	0.344961368	-6.048787192	2.87E-09	9.950813856
DPYSL3	-0.579720028	4.556356751	-6.048600878	2.88E-09	9.949764375
PSMB5	0.302844185	6.032593911	6.046601915	2.91E-09	9.938506237
AIMP2	0.294811509	3.689217548	6.045464344	2.93E-09	9.932100887
COX6C	0.325814924	4.979051303	6.044078057	2.95E-09	9.924296486
NCK1	-0.281133313	3.225417852	-6.043900603	2.96E-09	9.923297582
AQP1	-0.949755983	5.942978519	-6.043405426	2.96E-09	9.92051032
ARHGAP10	-0.23991391	1.281576632	-6.041551893	3.00E-09	9.910078877
MFSD3	0.433606463	3.468405144	6.0403122	3.02E-09	9.903103583
IGFL2	-0.436441689	0.806132486	-6.039899936	3.02E-09	9.9007842
GS1-600G8.5	-0.255269337	0.349864871	-6.039296232	3.04E-09	9.897388029
VAPB	0.238040297	2.839465747	6.039053341	3.04E-09	9.896021712
VWF	-0.582253537	4.035466879	-6.038645885	3.05E-09	9.893729789
MFAP3	-0.280141094	2.082272033	-6.037711935	3.06E-09	9.888476867
RERG	-0.404840419	1.621539677	-6.037240862	3.07E-09	9.885827625
MLK4	0.279815457	1.66157334	6.036033598	3.09E-09	9.879038965
PYCR1	0.494450091	5.245194268	6.033622142	3.14E-09	9.865482426
TTC39A	0.444868637	2.355680553	6.031028033	3.18E-09	9.850904288
DDX58	-0.379151583	2.999535974	-6.029917787	3.20E-09	9.844666677
COL12A1	-0.760606898	2.918826259	-6.027741223	3.24E-09	9.832441138
C2orf68	0.238631454	3.504325713	6.027556878	3.25E-09	9.831405869
CHRAC1	0.249005082	3.826606235	6.027535449	3.25E-09	9.831285524
NFKBIL1	0.269807608	3.651695412	6.024706394	3.30E-09	9.815401173
OSBPL2	0.254761621	2.759529574	6.023536365	3.32E-09	9.808833668

CTB-134H23.3	-0.297403316	0.454419593	-6.021301762	3.37E-09	9.796293654
M6PR	-0.29812372	4.428583067	-6.020499844	3.38E-09	9.791794478
NECAB1	-0.216059987	0.463782034	-6.01813271	3.43E-09	9.778516649
RGS3	-0.255370391	2.532566854	-6.013540677	3.52E-09	9.752771674
LCN12	0.334911613	0.754348372	6.01167426	3.56E-09	9.742312565
GCSH	0.237863587	1.617716984	6.010956547	3.57E-09	9.738291364
GLRX5	0.226072231	3.774931907	6.007650377	3.64E-09	9.719772901
TMCC3	-0.330134378	1.606592391	-6.007367425	3.65E-09	9.718188444
LAMP2	-0.305577435	5.288833662	-6.007217375	3.65E-09	9.71734823
CST6	-0.90173056	3.267184669	-6.00328378	3.74E-09	9.695328294
TYMSOS	0.281202879	0.805187398	6.002337782	3.76E-09	9.690034532
ARHGAP24	-0.263295561	1.443252062	-6.001347408	3.78E-09	9.684493223
CD58	-0.301059941	3.235586659	-6.000011232	3.81E-09	9.677018338
CKMT1B	0.372657824	0.799418591	5.99675343	3.88E-09	9.658799465
MATN3	-0.497331008	1.75663143	-5.99503096	3.92E-09	9.649170215
C16orf59	0.355862698	1.611915141	5.993045175	3.96E-09	9.638071899
SH3GLB1	-0.207463124	4.196548722	-5.992919439	3.96E-09	9.637369282
CXCR2	-0.231218567	0.543241962	-5.991835424	3.99E-09	9.631312306
COL25A1	0.574039344	0.592104578	5.990915498	4.01E-09	9.626172926
BMP1	-0.3857604	2.696750366	-5.990217746	4.03E-09	9.622275229
MZT2A	0.304783072	2.84859405	5.989970156	4.03E-09	9.620892261
WDR45B	0.314693462	5.41086191	5.98673557	4.11E-09	9.602829381
KANK3	-0.285775061	1.117490177	-5.986174901	4.12E-09	9.599699297
VARS	0.300384205	4.495374408	5.986099039	4.12E-09	9.599275802
MCF2L-AS1	0.435821891	1.503444696	5.98483725	4.15E-09	9.592232537
PDLIM7	-0.398704814	3.711559991	-5.980415491	4.26E-09	9.567560558
GPR125	0.284942745	2.187460734	5.980090617	4.27E-09	9.565748489
LTBR	0.332774388	4.617097663	5.979388538	4.28E-09	9.561832754
SPHK1	-0.469893068	1.993607179	-5.979100343	4.29E-09	9.560225506
RLTPR	-0.317132568	0.939253247	-5.978816052	4.30E-09	9.558640098
COMP	-0.900991787	3.076531764	-5.975983179	4.37E-09	9.542845538
NCAM2	-0.291372762	0.83264056	-5.972692941	4.45E-09	9.5245091
CMPK2	-0.408936102	2.195410791	-5.969880658	4.52E-09	9.508843223

PRKD3	-0.300209711	2.450725664	-5.96922803	4.54E-09	9.505208655
FAM129A	-0.512470129	3.116942778	-5.9688504	4.55E-09	9.503105745
CBX2	0.530691062	1.764689193	5.968379268	4.56E-09	9.500482317
RAPGEF1	-0.294660239	3.856171688	-5.967570093	4.58E-09	9.495976957
CYHR1	0.275735541	2.839878588	5.967254891	4.59E-09	9.494222102
DIAPH2	-0.259982782	1.783134034	-5.966097355	4.62E-09	9.487778334
APOBEC3F	-0.285339511	1.636310024	-5.965727229	4.63E-09	9.485718147
CORO7	-0.225454781	1.458416226	-5.963024262	4.70E-09	9.470676309
POLD2	0.301431838	4.676500058	5.961111152	4.76E-09	9.460033542
ACAD10	0.236312134	2.114499889	5.96039299	4.78E-09	9.456039119
ATXN2	0.211914303	2.159198621	5.9592237	4.81E-09	9.44953641
MRPS28	0.343252157	3.136705886	5.958426113	4.83E-09	9.44510147
NCL	0.261981363	6.347351878	5.956745048	4.88E-09	9.435755672
LETM1	0.241646582	2.975203749	5.955194704	4.92E-09	9.427138644
MBNL1	-0.292846595	4.195660811	-5.954915484	4.93E-09	9.425586904
FAM92B	-0.545831296	0.994979348	-5.953865939	4.96E-09	9.419754725
NUP35	0.207870787	2.052762914	5.952756479	4.99E-09	9.41359057
GLTPD2	0.546244791	1.168862198	5.95193629	5.01E-09	9.409034246
TNXB	-0.437283048	1.093859006	-5.949546456	5.08E-09	9.395761314
TCTEX1D1	-0.258862446	0.445028817	-5.949302044	5.09E-09	9.394404131
C21orf59	0.230429734	2.865404085	5.947573586	5.14E-09	9.384807649
COASY	0.229036756	4.521794452	5.944791927	5.22E-09	9.369368809
IMPA2	0.512294131	4.234897611	5.941409682	5.32E-09	9.350605022
NT5M	0.284372343	1.146605093	5.939611072	5.38E-09	9.340630585
SAP30L	-0.218579543	2.439416912	-5.937825935	5.43E-09	9.330733452
HMGB3	0.71573995	5.689680536	5.93704871	5.45E-09	9.32642518
ASPHD2	-0.355939775	1.404293096	-5.936367002	5.48E-09	9.322646773
VARS2	0.259488212	2.605530668	5.936103036	5.48E-09	9.321183828
RAB29	-0.323102692	3.008563778	-5.934803955	5.53E-09	9.313984914
MRPL3	0.262570942	5.11602133	5.934581513	5.53E-09	9.31275238
CXXC4	0.405884504	1.077658289	5.931930192	5.62E-09	9.298064692
TRIM16L	0.59626473	1.746941509	5.931832054	5.62E-09	9.297521141
GNG10	-0.264273769	2.854470853	-5.926850888	5.78E-09	9.26994246

YWHAH	-0.236273395	6.062049865	-5.926445087	5.79E-09	9.26769659
GSN	-0.408783042	5.270959739	-5.921443122	5.96E-09	9.24002462
ETNPPL	0.372859296	0.324026658	5.920661096	5.99E-09	9.235700118
PRKCDBP	-0.461652074	3.290292237	-5.920279704	6.00E-09	9.233591247
HCG4P3	-0.259660637	0.945370113	-5.919583268	6.02E-09	9.229740675
RP11-720N19.1	0.420684119	0.773131045	5.919183752	6.04E-09	9.227531943
KIAA0922	-0.313158869	2.229661019	-5.919043087	6.04E-09	9.226754303
KIAA1958	0.206196872	1.064629385	5.917228929	6.11E-09	9.216726521
BAG1	0.366538087	3.673028749	5.917192408	6.11E-09	9.216524678
RP11-59D5__B.2	0.509638962	1.182624254	5.914845801	6.19E-09	9.203557812
RAB37	-0.41405526	1.295268416	-5.910146188	6.36E-09	9.177602135
CORO2B	-0.261773408	0.769121551	-5.909736922	6.37E-09	9.175342633
TMEM68	0.245912714	2.011531786	5.908954047	6.40E-09	9.171020865
TMEM37	-0.54021651	2.989061656	-5.9069697	6.47E-09	9.160068735
SLCO3A1	-0.364874391	2.141523609	-5.906946813	6.47E-09	9.159942437
PLBD1	-0.575675428	3.743513198	-5.906001446	6.51E-09	9.15472585
SEL1L	-0.30511613	4.012905893	-5.905868681	6.51E-09	9.153993304
SNRPB	0.357883701	6.978152516	5.904742307	6.55E-09	9.147778978
LINC00473	0.569091908	0.601575217	5.904064818	6.58E-09	9.144041697
GPATCH4	0.290163296	3.230869578	5.902514223	6.64E-09	9.13548944
DAK	0.248688639	2.699092344	5.902109383	6.65E-09	9.133256877
GAB1	-0.249752244	1.660901946	-5.901880223	6.66E-09	9.13199319
IGHD2-15	-0.481316401	0.614055846	-5.901074576	6.69E-09	9.127550854
TARBP2	0.253310797	3.045652203	5.900240491	6.72E-09	9.122952266
FCGBP	-0.735044361	2.182540269	-5.894752368	6.94E-09	9.0927085
RP11-539L10.3	0.400523851	1.94833341	5.894582414	6.94E-09	9.091772313
IGLV5-48	-0.515884906	0.919610499	-5.893735088	6.98E-09	9.087105196
SFMBT2	-0.319858917	1.24929931	-5.891014349	7.08E-09	9.072123152
CRACR2B	0.536888891	3.104039957	5.890989163	7.08E-09	9.071984486
PWWP2B	0.322048379	3.506710797	5.88888536	7.17E-09	9.060403837
PCBD1	0.298124796	5.52499843	5.887802706	7.21E-09	9.054445627
RP11-111M22.3	0.364240746	2.111970016	5.88697261	7.25E-09	9.049877974
JUNB	-0.42536461	6.502489729	-5.886176354	7.28E-09	9.045497058

NDUFB9	0.364115508	5.572775864	5.886030382	7.29E-09	9.044693993
PUS1	0.283866256	2.340315745	5.88548424	7.31E-09	9.041689537
ANKRD9	0.335052367	1.935253741	5.884485158	7.35E-09	9.036193984
IGKV1D-43	-0.576451659	1.125264224	-5.883407296	7.39E-09	9.030266001
H1F0	0.370472916	7.047505612	5.881758412	7.46E-09	9.021199357
AC244250.1	-0.4165804	0.575278002	-5.880644332	7.51E-09	9.015074668
SEMA3D	-0.233288273	0.491927996	-5.878099283	7.62E-09	9.001086968
LTF	-1.051641429	3.501644976	-5.878068876	7.62E-09	9.000919884
IGKV2D-24	-0.633436994	1.363326082	-5.874665622	7.77E-09	8.982223767
NARS	0.253714157	5.079986449	5.874365847	7.78E-09	8.980577375
C2orf47	0.209958087	3.386352201	5.870977656	7.93E-09	8.961974187
FAM20A	-0.522838128	3.149944286	-5.869584097	7.99E-09	8.954325428
PYCR2	0.240382326	4.459776897	5.86886078	8.03E-09	8.95035602
MRPL12	0.384311467	4.504924513	5.868411617	8.05E-09	8.947891319
FAM101A	-0.502418243	1.21031117	-5.866566276	8.13E-09	8.937767076
NINL	0.376813843	2.103170655	5.864911809	8.21E-09	8.928692403
KANK2	-0.309949345	3.526206099	-5.864533192	8.23E-09	8.926616013
NUPR1	-0.52102867	3.718918216	-5.864405986	8.23E-09	8.925918428
DNMT3A	0.290634421	2.41355218	5.863990463	8.25E-09	8.923639822
MTG2	0.256814252	2.838258879	5.863176357	8.29E-09	8.919175914
NUAK1	-0.386102169	2.150836805	-5.863008507	8.30E-09	8.918255629
PIM3	0.385273665	5.598900525	5.860289889	8.42E-09	8.903353178
CARD11	-0.556745114	2.729979775	-5.859029242	8.48E-09	8.896444822
TXNRD2	0.270570063	2.351102378	5.858441386	8.51E-09	8.893223805
SIPA1	-0.278031135	3.895504986	-5.857571451	8.55E-09	8.888457727
TK2	-0.278991415	2.618280359	-5.857316466	8.57E-09	8.887060867
GPX2	1.532833758	3.487614538	5.855429968	8.66E-09	8.876727892
TRPM2	-0.371352082	1.725907322	-5.855194518	8.67E-09	8.875438457
ASPSCR1	0.315429985	2.144047936	5.854801383	8.69E-09	8.873285563
PTS	0.261917438	2.637946609	5.852132545	8.82E-09	8.858673769
GNL2	0.215994205	3.683643347	5.851960788	8.83E-09	8.857733599
RP11-289I10.2	-0.37239556	2.398243954	-5.851893582	8.83E-09	8.857365735
SNAI1	-0.41578661	1.980738193	-5.850828286	8.89E-09	8.851535103

MFAP2	-0.536819184	3.193435461	-5.849705136	8.94E-09	8.845388821
PLAGL1	-0.335182598	1.406044555	-5.848139142	9.02E-09	8.836820856
RAB3D	0.326864749	3.254006113	5.846641731	9.10E-09	8.828629991
MICAL2	-0.433073788	3.140911012	-5.846639651	9.10E-09	8.828618617
ANAPC7	0.2221945	3.146421339	5.846350698	9.11E-09	8.827038247
TIGD5	0.24416952	1.746517739	5.845291004	9.17E-09	8.82124306
NPM1P25	-0.230516984	0.882959211	-5.844654126	9.20E-09	8.817760583
RNF122	-0.286971739	2.615183786	-5.843944256	9.24E-09	8.813879368
SNRPC	0.260493121	5.895637969	5.841761531	9.35E-09	8.801947898
ACP2	-0.229415431	4.259280294	-5.841612535	9.36E-09	8.801133581
ANKH	-0.366845582	2.740719181	-5.841602736	9.36E-09	8.801080025
GPC6	-0.520182247	1.657102281	-5.841484793	9.36E-09	8.800435438
NDUFV1	0.244647334	4.925535716	5.841074674	9.39E-09	8.798194119
TIMM23	0.233567968	5.353202802	5.840472299	9.42E-09	8.79490237
SS18L1	0.311292032	2.471733369	5.840161628	9.43E-09	8.793204782
RP13-582O9.7	0.311594191	1.335796219	5.838109834	9.54E-09	8.781995237
ELMO1	-0.336268622	1.728098685	-5.837685901	9.57E-09	8.779679597
BMPR2	-0.248625783	3.231958519	-5.833926997	9.77E-09	8.759153819
DARS2	0.320725757	3.383056425	5.833472225	9.80E-09	8.756671286
MRPL42	0.209830751	2.057767023	5.830030037	9.99E-09	8.73788634
RP11-44K6.2	-0.525216642	1.02773898	-5.829722169	1.00E-08	8.736206691
PROSER1	0.281979982	2.979595181	5.829591799	1.00E-08	8.735495452
IGLV4-3	-0.539837096	0.922750791	-5.828743309	1.01E-08	8.730866804
DVL2	0.227956262	2.85163948	5.828545323	1.01E-08	8.729786841
TIGD3	0.20152403	0.621147116	5.827486045	1.01E-08	8.724009303
PYCRL	0.343915246	2.975893498	5.821834106	1.05E-08	8.693197852
RP3-407E4.2	0.510273701	1.005502813	5.819880515	1.06E-08	8.682553946
COX5A	0.283216888	5.661508599	5.815239511	1.08E-08	8.657280485
RP11-344B5.2	-0.430747931	1.72741614	-5.814790243	1.09E-08	8.654834844
IGHV3-16	-0.229513645	0.294454667	-5.814123402	1.09E-08	8.651205125
RNY4P19	0.409787568	0.741749296	5.813805822	1.09E-08	8.649476618
RCN2	0.261327922	2.973198458	5.813805135	1.09E-08	8.649472878
SLC7A11	0.666848007	1.83079022	5.812629245	1.10E-08	8.643073518

RP5-963E22.6	0.26102174	0.975772224	5.812060995	1.10E-08	8.639981427
METAP1D	0.211277885	1.244502375	5.810998974	1.11E-08	8.634203218
FBRSL1	0.310786645	2.844172411	5.810855163	1.11E-08	8.63342085
GSAP	-0.324402018	2.173076683	-5.810610925	1.11E-08	8.632092165
ALKBH2	0.280977654	2.559231167	5.809937606	1.12E-08	8.628429484
XRN1	-0.237087813	2.225288903	-5.809805399	1.12E-08	8.62771036
SEMA5A	-0.392229787	1.32694043	-5.808920892	1.12E-08	8.62289953
MRPS15	0.299265307	4.572724911	5.807544457	1.13E-08	8.615414379
TCEA1	0.284841426	4.314238433	5.807403033	1.13E-08	8.614645391
TAPT1-AS1	0.218798924	1.009057214	5.806284136	1.14E-08	8.608562021
MRPS2	0.256562998	4.002122705	5.805787414	1.14E-08	8.605861702
ZNF48	0.240424949	2.368124412	5.803915937	1.16E-08	8.595689646
PPP1R14B	0.372867135	5.783479931	5.80359777	1.16E-08	8.593960594
SNRPEP2	0.239712944	1.592148009	5.802325474	1.17E-08	8.587047239
DDX55	0.239329482	2.342046061	5.800798896	1.18E-08	8.578753922
TUFT1	0.346483784	3.603624228	5.799985783	1.18E-08	8.574337363
PTEN	-0.211029178	2.997832862	-5.798659974	1.19E-08	8.567137173
LONP1	0.265148138	4.257865604	5.796954939	1.20E-08	8.557879599
GGA2	-0.254893906	3.889425948	-5.796431321	1.21E-08	8.555037065
ENSA	0.243060098	5.359845964	5.794334656	1.22E-08	8.543657267
DNA2	0.309906113	1.42629113	5.792546541	1.23E-08	8.533954988
IGLJ3	-0.301028539	0.484980982	-5.789655748	1.25E-08	8.518275134
NDUFAF6	0.267220755	1.924768844	5.789359459	1.25E-08	8.516668427
ARTN	0.364509753	0.78636691	5.788658873	1.26E-08	8.512869602
VPS72	0.261504346	3.829214446	5.787309668	1.27E-08	8.505554863
SETX	-0.242835375	2.941223828	-5.787140994	1.27E-08	8.5046405
PHLDB1	-0.270502632	1.941073292	-5.78703265	1.27E-08	8.504053186
MDH2	0.258768758	5.840063651	5.786908736	1.27E-08	8.503381488
BEND3	0.203916354	1.250795166	5.785640415	1.28E-08	8.496507036
SARS2	0.248413212	1.523701382	5.783728957	1.29E-08	8.486149195
IGHV3-22	-0.382538251	0.724254609	-5.780982381	1.31E-08	8.471271229
CSNK2B	0.229512052	4.232708895	5.780441639	1.32E-08	8.468342808
SMPD4	0.236199591	3.337763912	5.77963003	1.32E-08	8.463947929

PDE1A	-0.255593585	0.933713881	-5.778261299	1.33E-08	8.456537457
PGAM5	0.306656666	3.828543889	5.777022644	1.34E-08	8.449832546
BNIP3	0.421321235	4.015104122	5.776510502	1.35E-08	8.447060662
SMUG1	0.251292907	2.939712616	5.776415911	1.35E-08	8.446548727
ATAT1	0.314944546	2.213049559	5.775965249	1.35E-08	8.444109807
SOCS7	0.308711731	2.091328977	5.773722623	1.37E-08	8.431975483
SLC16A14	0.767126905	2.186201737	5.773222711	1.37E-08	8.429271138
MAN2A1	-0.309908652	2.682845336	-5.772685546	1.38E-08	8.426365498
LIPM	-0.306092307	0.830294919	-5.768999625	1.40E-08	8.406433932
PKP3	0.385892464	4.437684973	5.768181736	1.41E-08	8.402012712
RP11-116O18.1	0.799945551	0.79307119	5.766948681	1.42E-08	8.395348296
ZNF768	0.285224536	4.258976978	5.766264851	1.43E-08	8.391652867
RPS10L	0.214116636	0.751943511	5.766107324	1.43E-08	8.390801641
ATAD3A	0.312315802	3.261938751	5.7646592	1.44E-08	8.382977408
PRICKLE2	-0.250079514	1.360582146	-5.763514974	1.45E-08	8.376796352
RP11-973H7.3	0.200428303	1.194734456	5.763145009	1.45E-08	8.374798046
PTP4A1	0.433005193	5.729741672	5.762246507	1.46E-08	8.369945405
TAB2	-0.231925989	4.023454861	-5.759845177	1.48E-08	8.356979521
GALNT10	-0.427020596	3.603884806	-5.759372269	1.48E-08	8.354426628
VEGFC	-0.459491195	1.973234406	-5.758828607	1.49E-08	8.351492014
HDGF	0.287331094	6.73346424	5.75827025	1.49E-08	8.348478333
KDM1A	0.239727044	4.115350768	5.757741312	1.49E-08	8.34562367
RALY-AS1	0.247335447	1.525363328	5.756782253	1.50E-08	8.340448244
DCAF13	0.28510824	2.789566841	5.755897182	1.51E-08	8.33567275
IGLV10-54	-1.030244658	3.234007325	-5.755635834	1.51E-08	8.334262743
PTPN1	-0.28105197	4.512779781	-5.754304927	1.52E-08	8.327083194
PRR3	0.227404737	2.285452044	5.751820005	1.54E-08	8.313682215
LRRC1	0.272355446	2.638117031	5.751579771	1.55E-08	8.312386923
SNRPF	0.305041109	3.773680153	5.750723897	1.55E-08	8.307772608
KCNJ2	-0.325112099	1.117016331	-5.750557209	1.56E-08	8.306874003
C1orf210	0.409441697	3.927918234	5.749800144	1.56E-08	8.302793009
MZT2B	0.337964956	4.759190782	5.747463763	1.58E-08	8.290201592
PARP9	-0.291259202	3.64585117	-5.746325389	1.59E-08	8.284068196

HOXA5	-0.355505982	1.883475354	-5.746227619	1.59E-08	8.283541476
NOC2L	0.276591087	4.181590836	5.74406594	1.61E-08	8.271897758
ZNF74	0.232628852	1.746691353	5.743979811	1.61E-08	8.271433909
GLS	-0.573171775	3.941240608	-5.7422459	1.63E-08	8.262097226
SAC3D1	0.289258019	2.825848313	5.741547438	1.64E-08	8.258336877
TSEN54	0.257104386	3.66985909	5.739976706	1.65E-08	8.249881899
RNASEL	-0.242803212	2.014811343	-5.737308719	1.67E-08	8.235525235
BZW2	0.311433876	4.72280351	5.736705653	1.68E-08	8.232280895
RP13-977J11.2	-0.242170189	0.93326022	-5.734128436	1.70E-08	8.218419493
RB1	-0.264637943	3.048754226	-5.732578542	1.72E-08	8.210086114
GSR	0.488931671	5.064474775	5.732111903	1.72E-08	8.207577503
RRP12	0.266521129	2.801035445	5.731845166	1.73E-08	8.206143625
ARHGEF25	-0.322831714	1.852336252	-5.731832955	1.73E-08	8.206077987
RHPN1-AS1	0.269605916	1.157183464	5.731258486	1.73E-08	8.202990066
POP5	0.253630894	3.373250677	5.730682759	1.74E-08	8.199895657
EFNB1	-0.453585524	3.156737876	-5.728815465	1.75E-08	8.189861227
PPM1F	-0.227928937	2.003643146	-5.728528196	1.76E-08	8.18831776
RNFT2	0.321356239	0.896121652	5.728161888	1.76E-08	8.186349719
KLHL23	0.346680904	2.402249014	5.727855159	1.76E-08	8.184701864
IGHV3OR16-7	-0.22198176	0.315428183	-5.727731467	1.77E-08	8.184037371
AHCY	0.311459548	5.432709465	5.727276006	1.77E-08	8.181590658
UCHL5	0.206982987	2.952326633	5.724924386	1.79E-08	8.168960612
FH	0.238599558	4.934538501	5.722855984	1.81E-08	8.157855428
PTOV1	0.248048953	4.183838112	5.722188683	1.82E-08	8.154273459
TSEN34	0.224389662	4.067891282	5.721919538	1.82E-08	8.15282883
IGLV2-28	-0.512251036	1.248659802	-5.721880351	1.82E-08	8.152618501
GSTO1	-0.335790631	5.606682443	-5.721858389	1.82E-08	8.152500626
PROS1	-0.457039179	3.255960062	-5.721354868	1.83E-08	8.149798181
TMEM158	-0.460422982	1.442564961	-5.718864353	1.85E-08	8.136434415
NAT8L	0.358835174	0.575640136	5.717032581	1.87E-08	8.126608632
RP11-98G7.1	0.255044511	0.467952312	5.712887313	1.92E-08	8.104383243
OMG	-0.381755038	0.691316393	-5.712271191	1.92E-08	8.101081027
RP11-379F4.7	-0.224587941	1.122457557	-5.711756158	1.93E-08	8.098320861

FAM213A	0.244868884	3.589207275	5.710784952	1.94E-08	8.09311656
AKR1C2	1.429761519	2.635491437	5.71064036	1.94E-08	8.092341817
PABPN1	0.292907837	4.472975873	5.706948865	1.98E-08	8.072568098
CTPS2	0.244137759	2.757888364	5.706262292	1.99E-08	8.068891669
RBFA	0.229807856	2.479356524	5.704895073	2.00E-08	8.061571693
C12orf56	0.285203692	0.387858968	5.704079249	2.01E-08	8.057204573
PURA	-0.219611882	1.856400437	-5.701758552	2.04E-08	8.044784841
TBX3	-0.319865676	1.373703903	-5.701474147	2.04E-08	8.043263087
RP1-63M2.7	0.330805155	1.047895564	5.699174804	2.07E-08	8.030962542
NDUFV2	0.261788363	2.510417835	5.698044929	2.08E-08	8.024919765
RAB26	0.387690524	1.150040489	5.697077726	2.09E-08	8.019747822
IGLV9-49	-0.989958684	3.085032712	-5.696928987	2.09E-08	8.018952535
ZNF219	0.355240787	2.187302176	5.696655067	2.10E-08	8.017487973
AC010136.2	0.313287129	0.824905789	5.696072533	2.10E-08	8.014373552
BCL2	-0.374184117	1.453047454	-5.69598541	2.10E-08	8.01390779
NSMF	0.359163074	3.520616803	5.695697874	2.11E-08	8.01237065
RP3-407E4.3	0.373490763	0.576327475	5.693944823	2.13E-08	8.003000482
MAGIX	0.294589578	1.47302491	5.689995662	2.17E-08	7.981901245
WDR77	0.221463522	3.487149891	5.688601802	2.19E-08	7.974457318
GAS8	0.263847016	2.303532779	5.68302814	2.26E-08	7.944707092
ALG3	0.278002278	4.084307635	5.681981823	2.27E-08	7.939125075
KPTN	0.283666933	2.286958621	5.680403742	2.29E-08	7.930707853
ATM	-0.23330455	1.691886906	-5.679944647	2.30E-08	7.928259498
PARP11	-0.214982638	1.316029853	-5.6798341	2.30E-08	7.927669978
PPM1G	0.223944112	5.202171282	5.67966716	2.30E-08	7.926779748
RP11-286N22.16	0.323945803	0.603417332	5.677622931	2.33E-08	7.915880453
AGBL5	0.241946144	2.896878148	5.677086094	2.33E-08	7.913018754
FAM216B	-0.561349354	0.898158121	-5.677007146	2.33E-08	7.912597928
6-Mar	0.288277623	4.13021743	5.676710081	2.34E-08	7.911014492
CD36	-0.477179635	1.494335329	-5.673665793	2.38E-08	7.894791826
ATP6V1B1	0.51836078	1.141056855	5.671653471	2.40E-08	7.884072586
DENND2D	-0.25029165	3.164301982	-5.670352785	2.42E-08	7.877145864
TMEM69	0.211877293	3.610430724	5.666023198	2.48E-08	7.854098974

ADPRHL1	0.385366346	1.643354633	5.66265804	2.52E-08	7.836196528
KAZALD1	0.365740658	1.800237713	5.660115824	2.56E-08	7.822678287
BST2	-0.564786257	6.752122824	-5.658280607	2.59E-08	7.812922825
SLC25A19	-0.230211541	2.044967376	-5.657385164	2.60E-08	7.808163927
RPL7P9	0.391755546	3.256078306	5.655407221	2.63E-08	7.797654356
ARHGAP22	-0.20230623	0.815080676	-5.654800206	2.64E-08	7.794429696
ZNF76	0.237522327	2.81600746	5.654782175	2.64E-08	7.794333915
APOBEC3A	-0.289430031	0.696836666	-5.653764407	2.65E-08	7.788927904
IGKV1OR1-1	-0.235955369	0.306036034	-5.653022662	2.66E-08	7.784988568
PDLIM5	-0.330026129	3.156223813	-5.652385924	2.67E-08	7.781607275
AAAS	0.202581316	3.352183565	5.649898363	2.71E-08	7.76840069
POLR2J	0.288459771	4.731074266	5.648668476	2.73E-08	7.761873052
SLMO2	0.320637317	5.04992001	5.648409786	2.73E-08	7.760500209
ECHS1	0.238330621	6.209958596	5.647997077	2.74E-08	7.758310118
RBM47	0.270170655	4.178936407	5.647941145	2.74E-08	7.758013319
ATL2	0.288468174	4.231642132	5.647864242	2.74E-08	7.757605244
RP11-443B20.1	0.244230049	1.074134899	5.647041831	2.75E-08	7.753241528
ITGA2	-0.654875582	3.395897618	-5.645547589	2.77E-08	7.745314508
MMP12	-0.919934434	3.232174991	-5.644978513	2.78E-08	7.742296019
MYO19	0.274808656	2.242734411	5.64476344	2.78E-08	7.741155302
VPS37D	0.365640501	1.604155114	5.643711053	2.80E-08	7.73557413
ANO1	-0.525453436	2.155735243	-5.642577243	2.82E-08	7.72956217
PIGU	0.247773269	3.732655691	5.641757368	2.83E-08	7.725215489
EIF5A2	-0.247398622	1.079695523	-5.640057525	2.86E-08	7.716205312
RPH3AL	0.385785579	2.367516559	5.639375259	2.87E-08	7.712589565
NR2F1-AS1	-0.24115397	0.884872243	-5.637318977	2.90E-08	7.701694403
ILF2	0.281007402	6.67430081	5.637235818	2.90E-08	7.701253857
RPL7P23	0.238971635	0.909924042	5.637009998	2.90E-08	7.700057584
RP3-512B11.3	0.38754848	1.29139258	5.635494188	2.93E-08	7.692028723
CTA-126B4.7	-0.251019208	0.535415141	-5.635389851	2.93E-08	7.691476146
TWF2	-0.249436882	4.705040955	-5.634209198	2.95E-08	7.685223944
IGKV3D-7	-0.408242741	0.618328169	-5.633195546	2.97E-08	7.679857021
EHD2	-0.46675953	5.127253839	-5.631861352	2.99E-08	7.672794239

SUDS3	0.253130985	3.492758089	5.631769208	2.99E-08	7.672306515
DHX34	0.267679493	2.509635859	5.631441411	2.99E-08	7.670571518
ATP11C	-0.273473685	2.008553582	-5.630956578	3.00E-08	7.668005506
IGHD4-11	-0.320474639	0.325437413	-5.630315682	3.01E-08	7.664613815
TARS2	0.240708249	3.485851208	5.629615235	3.02E-08	7.660907364
DCXR	0.361875088	4.759528576	5.629606112	3.02E-08	7.660859093
NT5E	-0.724614062	3.476311925	-5.628845498	3.04E-08	7.65683473
C1orf27	0.238063312	3.541098181	5.626997939	3.07E-08	7.647061403
JADE2	-0.301336798	2.754092696	-5.625332303	3.10E-08	7.638252842
PTPRG	-0.339650397	1.927012488	-5.625067216	3.10E-08	7.636851166
RP11-686D22.4	-0.298743756	1.373869803	-5.624045115	3.12E-08	7.631447238
ZNF517	0.245809533	1.816466672	5.623954172	3.12E-08	7.630966459
PLSCR1	-0.352108585	4.100716595	-5.621419643	3.16E-08	7.617570131
UBQLN4	0.267328087	4.172977378	5.620710558	3.17E-08	7.613823194
DPYSL2	-0.436961273	4.32469104	-5.620182169	3.18E-08	7.611031356
TSHZ2	-0.22881953	0.691313529	-5.618810745	3.21E-08	7.603786276
SLC14A2	0.538633701	0.520010127	5.618434943	3.21E-08	7.601801224
DST	-0.403226817	1.823263115	-5.617432835	3.23E-08	7.596508496
PCSK5	-0.245696936	0.913948091	-5.616735353	3.24E-08	7.592825168
AC245028.1	-0.425769162	0.815276052	-5.616522073	3.25E-08	7.591698939
KRTCAP2	0.27866208	3.111086171	5.614705188	3.28E-08	7.582106371
MSI1	0.505083825	0.854307101	5.61376523	3.30E-08	7.577144771
MIF	0.410932269	5.300621617	5.6103109	3.36E-08	7.558917251
EVC	-0.289653081	1.921245197	-5.609822178	3.37E-08	7.556339197
PROCR	-0.446639241	3.842910535	-5.606480819	3.43E-08	7.538718546
CTD-2134A5.4	0.297285006	1.422387336	5.606030745	3.44E-08	7.536345787
GLUL	-0.34023597	6.371384383	-5.603993356	3.48E-08	7.525606916
COL11A1	-0.836391302	2.154123664	-5.60370763	3.48E-08	7.52410116
TMEM41B	0.263897079	3.891305657	5.60368034	3.48E-08	7.523957346
IGKV1D-17	-0.726961419	1.388005359	-5.603285283	3.49E-08	7.521875539
DCUN1D3	-0.241806649	2.053396586	-5.601915777	3.52E-08	7.514659748
RP11-589N15.2	-0.354039588	2.374839492	-5.601185316	3.53E-08	7.510811656
PTK2	0.240342619	3.462946174	5.60100107	3.53E-08	7.509841111

RP1-92O14.3	0.30981244	1.140343475	5.597810447	3.60E-08	7.493038482
BARX1	0.990025503	1.615215156	5.597371802	3.60E-08	7.490729125
LGALS3	-0.42081089	7.074171937	-5.595460385	3.64E-08	7.480667853
MRM1	0.264025916	2.535193637	5.594129748	3.67E-08	7.473665462
C21orf91	-0.286818609	1.921935015	-5.592551993	3.70E-08	7.465364532
MYL6B	0.30193886	3.426507508	5.589513681	3.76E-08	7.449385094
MIR8071-1	-0.601573807	1.62469877	-5.58826489	3.79E-08	7.442819528
PPAPDC1A	-0.427911716	0.973454093	-5.586452024	3.82E-08	7.433290617
RP11-705C15.3	-0.253054987	1.36788624	-5.585861276	3.84E-08	7.430186075
RP11-5C23.1	-0.254674201	1.763532005	-5.585818422	3.84E-08	7.429960879
SCT	-0.328359284	1.031104429	-5.585774911	3.84E-08	7.429732228
RAP1B	-0.238917408	1.987626945	-5.584701913	3.86E-08	7.424094152
PDF	0.203125924	1.33150188	5.583804647	3.88E-08	7.419380189
MMD	-0.427418546	3.166598635	-5.583706593	3.88E-08	7.418865087
SMURF2	-0.343443405	3.297174282	-5.583441672	3.89E-08	7.417473422
MGAT4A	-0.322453611	2.70271264	-5.583187203	3.89E-08	7.416136716
RAB3IP	0.347375568	1.83962436	5.583114591	3.89E-08	7.4157553
XXbac-BPG248L24.12	-0.317427987	1.689943339	-5.580363409	3.95E-08	7.401307171
IGLV4-60	-0.975079376	2.948234859	-5.579630144	3.97E-08	7.397457412
MIAT	-0.466150774	1.163916712	-5.578644141	3.99E-08	7.39228144
ZC3H3	0.274768728	3.322912338	5.578345512	4.00E-08	7.390713964
GPX1	-0.346813635	7.491134282	-5.577180567	4.02E-08	7.384599972
TNIP3	-0.36652554	0.799573653	-5.577067687	4.02E-08	7.384007601
MSTO1	0.241949266	2.257489525	5.576951773	4.03E-08	7.383399322
SLC1A5	0.311661259	5.470026576	5.576789321	4.03E-08	7.382546842
CCL7	-0.416493309	0.91657669	-5.576729215	4.03E-08	7.382231437
FAM71E1	0.287585629	1.370821956	5.57645359	4.04E-08	7.380785141
SCN4B	-0.306699556	0.936011738	-5.57584472	4.05E-08	7.377590421
ANKRD13A	-0.266433336	3.394389692	-5.575343539	4.06E-08	7.374960968
TMEM154	-0.232435067	1.205969515	-5.574207309	4.09E-08	7.3690005
PDIA3	0.33380729	7.268580511	5.573892117	4.09E-08	7.367347247
UBE2K	0.215007597	4.294185573	5.573279639	4.11E-08	7.364134893
CTD-2555O16.2	-0.20337279	1.096674046	-5.572137532	4.13E-08	7.358145555

TBX4	-0.403083946	1.585435763	-5.571767428	4.14E-08	7.356204921
YWHAE	0.226611965	6.788973199	5.571460778	4.15E-08	7.354597091
SLC25A33	0.237567064	1.826684905	5.570469444	4.17E-08	7.349399857
RASAL2	-0.244434173	1.672783252	-5.569872485	4.18E-08	7.346270597
SLC16A6	-0.265551792	0.901973649	-5.56788727	4.23E-08	7.335866209
RGS2	-0.534536918	4.445116752	-5.567204921	4.24E-08	7.332290819
DDX51	0.23133761	2.270974854	5.566194741	4.27E-08	7.32699836
TMEM223	0.25323909	3.265676535	5.564626984	4.30E-08	7.318786359
TAF4	0.207524026	1.914417085	5.561958844	4.36E-08	7.30481519
AC246787.5	-0.387660716	0.650637321	-5.557803003	4.46E-08	7.283065756
CXCL14	-1.19952236	4.218432979	-5.556629712	4.49E-08	7.276927978
GUCY1A2	-0.20978328	0.700423004	-5.556281612	4.50E-08	7.2751072
GSE1	0.338258032	3.608774386	5.555937212	4.51E-08	7.273305876
ATP8B2	-0.364419903	2.660735037	-5.555897172	4.51E-08	7.273096459
NFIX	-0.5125544	3.457769478	-5.55485295	4.54E-08	7.267635474
C4B	-0.471771668	1.714604541	-5.554542043	4.54E-08	7.266009691
DUS1L	0.291353175	4.248199046	5.554333663	4.55E-08	7.264920085
MCCC2	0.251273593	3.791328941	5.553880691	4.56E-08	7.262551648
IGKV1D-27	-0.633774062	1.206918253	-5.553488536	4.57E-08	7.260501337
SOX7	-0.318262069	1.147987366	-5.553394692	4.57E-08	7.26001071
IGLV3-30	-0.211868434	0.290860217	-5.551711363	4.61E-08	7.251211321
FBXL3	-0.221437528	2.938082634	-5.547758881	4.71E-08	7.230559484
GTF2IRD1	0.253279277	2.641014667	5.5464105	4.75E-08	7.223517121
MRPS17	0.243908141	2.5901131	5.544810575	4.79E-08	7.215162952
AKR7A3	0.779280925	1.743734483	5.544449833	4.80E-08	7.213279595
ECI1	0.283744147	3.8128464	5.543551802	4.82E-08	7.208591636
RIOK1	0.236012886	3.11280267	5.542910878	4.84E-08	7.205246254
FAM86C2P	0.202865728	1.412532515	5.541072566	4.88E-08	7.195652856
MRPS18B	0.250497611	5.306539883	5.539361978	4.93E-08	7.186728521
DMXL2	-0.254082518	1.825830275	-5.537093245	4.99E-08	7.174896034
GPD1	-0.389213488	0.906123543	-5.536377492	5.01E-08	7.171163944
ENTPD8	0.467273207	1.164650267	5.533457794	5.09E-08	7.155944411
TUBG1	0.314799272	4.250617631	5.530722119	5.16E-08	7.141690581

AP1M2	0.309519	5.107407476	5.529230326	5.21E-08	7.133920437
XYLT1	-0.328120729	1.3293829	-5.528241068	5.23E-08	7.128768816
MTFR1	0.333457964	3.135142316	5.527024361	5.27E-08	7.122433858
SBK1	0.552804316	2.618044	5.525006311	5.32E-08	7.111929306
PTRHD1	0.298278385	3.519837566	5.52363937	5.36E-08	7.1048159
SNRPA1	0.246997848	3.196443902	5.523121835	5.38E-08	7.102123111
MRPL37	0.215311326	4.734265626	5.522954476	5.38E-08	7.101252372
SLC25A4	0.279233483	3.108387945	5.522645552	5.39E-08	7.09964516
STX17-AS1	0.248206004	1.059375481	5.521118809	5.44E-08	7.091703267
MBNL3	-0.260335153	1.465764872	-5.520709158	5.45E-08	7.089572652
TMEM189	0.228248159	2.745971808	5.520180184	5.46E-08	7.086821637
NQO1	0.810716682	5.69915494	5.518708137	5.51E-08	7.07916725
PJA2	-0.255241706	4.490497453	-5.518037375	5.53E-08	7.075680006
CAV2	-0.522478302	3.359399621	-5.516716159	5.57E-08	7.068812192
SOD3	-0.520809508	3.980362822	-5.514760211	5.63E-08	7.058647644
AP000695.6	-0.237321628	0.656950081	-5.514592569	5.63E-08	7.057776602
LLGL2	0.379806725	4.202413431	5.513836222	5.65E-08	7.053847028
SETD4	0.208503726	1.803015024	5.513233096	5.67E-08	7.050713847
RIPK3	-0.290989681	2.267244764	-5.5124751	5.69E-08	7.046776565
RP11-793H13.11	0.232460403	1.049123035	5.5116786	5.72E-08	7.04263979
RNU6-137P	0.265666791	0.945084944	5.511383179	5.73E-08	7.041105603
LNX1	0.257503081	1.502991885	5.511374748	5.73E-08	7.041061819
DNASE1L3	-0.314917738	0.679491713	-5.510604642	5.75E-08	7.037062827
NDUFS8	0.271443448	4.17182715	5.510565339	5.75E-08	7.036858749
RP11-367G18.1	-0.255137489	0.59635506	-5.509439998	5.79E-08	7.031016028
PCDHB7	-0.220444586	0.712648203	-5.509091444	5.80E-08	7.029206565
LEMD2	0.205217934	3.102863962	5.508549357	5.82E-08	7.026392605
NPC2	-0.56126042	7.413801182	-5.50641962	5.88E-08	7.015339576
MMP3	-0.413514921	0.722369641	-5.505072204	5.92E-08	7.00834863
SCGB1A1	-1.638256036	4.620295314	-5.504998467	5.93E-08	7.007966094
NFE2L3	-0.453185071	3.313813472	-5.5045149	5.94E-08	7.005457549
RP11-61L19.3	0.206694573	1.103208729	5.504012116	5.96E-08	7.002849524
RP11-366M4.11	-0.217786072	0.587164647	-5.503641536	5.97E-08	7.000927395

TRAPPC9	0.269837623	2.861998253	5.502492196	6.01E-08	6.994966718
SOCS3	-0.467223752	5.631272389	-5.500948241	6.06E-08	6.98696123
TMEM97	0.39849027	3.878996932	5.499643593	6.10E-08	6.980198116
HERC6	-0.362923564	2.366462933	-5.498172155	6.15E-08	6.972572084
PICALM	-0.2135269	5.107630921	-5.497274693	6.18E-08	6.96792169
COPZ1	0.200016875	5.613748977	5.497065236	6.18E-08	6.966836438
COX6A1	0.309917592	6.213678942	5.493175792	6.31E-08	6.946690879
LUZP1	-0.255126149	2.284555707	-5.492939873	6.32E-08	6.945469331
IGLV3-32	-0.223785394	0.347825604	-5.492614649	6.33E-08	6.943785454
RP3-407E4.4	0.714620975	2.006865887	5.492591958	6.33E-08	6.943667973
MIR568	-0.431159782	2.908735962	-5.490092221	6.42E-08	6.930728352
ATP1B1	0.532453531	7.425998343	5.48969448	6.43E-08	6.928669964
ITGB3	-0.395904099	0.990627365	-5.489671128	6.43E-08	6.928549118
IL6ST	-0.350090547	4.510049577	-5.487736335	6.50E-08	6.918538128
RAP2C	-0.221677772	3.500307505	-5.487505075	6.51E-08	6.917341753
LYN	-0.316110945	4.254996919	-5.485290038	6.59E-08	6.905884991
KLF13	-0.324689664	3.37683768	-5.481418173	6.72E-08	6.885868508
GID8	0.234480299	4.298024153	5.481068522	6.74E-08	6.884061526
RP4-647J21.1	-0.558341752	1.430073067	-5.480150556	6.77E-08	6.879317997
RPN2	0.256884961	7.368191555	5.477964108	6.85E-08	6.868022502
AC244250.3	-0.553108628	1.069171931	-5.477470897	6.87E-08	6.865475061
LYPD5	-0.296791488	0.811099129	-5.477301008	6.87E-08	6.864597628
FNDC1	-0.613625704	2.209901697	-5.47665804	6.90E-08	6.861277078
E2F5	0.284381111	1.853849834	5.476304535	6.91E-08	6.859451583
GADD45B	-0.427747846	4.675662506	-5.475806465	6.93E-08	6.856879739
JDP2	-0.257131784	2.452814458	-5.475098146	6.95E-08	6.853222593
B4GALNT4	0.61197794	1.559028381	5.475055755	6.95E-08	6.853003738
RP11-703I16.1	0.400245246	1.974791289	5.473344725	7.02E-08	6.844171262
CMTM7	-0.325941471	3.078024203	-5.473243037	7.02E-08	6.84364642
DHRS11	0.240891648	1.64680461	5.471522329	7.09E-08	6.834766596
CHSY1	-0.296024863	3.200500933	-5.470725735	7.12E-08	6.830656563
KIAA1598	0.301537824	2.992603449	5.469307899	7.17E-08	6.823342536
SLC35B2	0.250900842	5.353761296	5.467166649	7.25E-08	6.812299896

C14orf79	0.24092487	1.995144078	5.466062521	7.30E-08	6.806607295
IGLV3-24	-0.220808607	0.287014558	-5.463062967	7.41E-08	6.791147515
ZNF664	0.277089785	4.686740747	5.46136229	7.48E-08	6.782385522
TNFAIP8L1	-0.266812492	2.029534491	-5.460593294	7.51E-08	6.7784244
HCN3	0.29265555	1.37796358	5.459640221	7.55E-08	6.773515781
IGHD4-17	-0.506177161	0.707300269	-5.459471288	7.56E-08	6.772645802
MMP11	-0.810445825	3.275133022	-5.458168771	7.61E-08	6.765938847
GSTK1	-0.294066404	4.662926714	-5.457906352	7.62E-08	6.764587764
TMEM256	0.359763972	4.973351641	5.456855207	7.66E-08	6.759176446
TMUB1	0.309801754	4.528694249	5.455930147	7.70E-08	6.754414982
GNG12	-0.375988432	4.503380549	-5.455148698	7.73E-08	6.750393271
MEX3A	0.536204924	2.22482565	5.454377145	7.76E-08	6.746422989
CCDC8	-0.397783988	1.703818488	-5.45422274	7.77E-08	6.745628507
CCDC90B	-0.268552092	2.375447703	-5.454071454	7.78E-08	6.744850091
TRIM38	-0.207803789	2.746397231	-5.451616371	7.88E-08	6.732220591
MAP7	0.311931288	3.205440239	5.450967387	7.90E-08	6.72888291
DOLPP1	0.215029797	3.532284139	5.45068294	7.92E-08	6.727420133
RND3	-0.495129536	3.14456952	-5.449156631	7.98E-08	6.719572186
PPP1R14BP3	0.366179035	4.996469403	5.446968988	8.07E-08	6.708327205
PARVA	-0.242436737	3.233657059	-5.446629199	8.09E-08	6.706580973
IGHD6-6	-0.398961704	0.493277715	-5.446025745	8.11E-08	6.703479961
PPARGC1A	0.372019176	0.697827712	5.445069424	8.16E-08	6.69856627
AP006285.2	0.236729059	0.647157249	5.443535945	8.22E-08	6.690688667
CASC9	0.63266653	1.172699766	5.441174881	8.33E-08	6.67856354
HERC1	-0.237903021	2.002013103	-5.439938592	8.38E-08	6.672216503
ZNHIT2	0.283224644	3.384086296	5.439031636	8.42E-08	6.667561058
PSMG3	0.33447208	4.397299651	5.438884487	8.43E-08	6.666805801
TBRG4	0.259873324	3.580388003	5.438580854	8.44E-08	6.665247431
SRRT	0.234476772	4.516420709	5.437254012	8.50E-08	6.658438433
PRKRIP1	0.23808589	3.180508781	5.435781955	8.57E-08	6.650885953
MCM7	0.38290412	4.725237092	5.43560743	8.57E-08	6.649990662
PRPF19	0.207100934	5.481623106	5.432765824	8.70E-08	6.635417176
TONSL	0.337152071	2.004874543	5.432487362	8.72E-08	6.633989417

DGUOK-AS1	0.24615132	1.328955716	5.430591063	8.81E-08	6.624268249
SNORA16	-0.243729012	0.609359832	-5.429806085	8.84E-08	6.620245029
SFTPC	-1.685512793	5.146181058	-5.429699351	8.85E-08	6.619698027
MRS2	0.241660921	3.2180375	5.429672298	8.85E-08	6.619559383
TRIT1	0.251106241	3.250632802	5.426709577	8.99E-08	6.60437963
SLC45A4	0.369070923	2.558636784	5.425218012	9.06E-08	6.596740256
HGD	0.808538206	2.09157809	5.424806259	9.08E-08	6.594631701
RP11-499O7.7	0.550486581	0.854815318	5.424202862	9.11E-08	6.591542009
TMEM44-AS1	0.314173939	2.210248003	5.424190017	9.11E-08	6.591476238
CADM4	0.448661249	3.443056638	5.423134078	9.16E-08	6.586070055
PPP1R35	0.343044826	3.96650571	5.422194568	9.21E-08	6.581260743
ATP6V1H	0.202679382	3.567244235	5.421436333	9.24E-08	6.577379913
AC092066.1	0.298931464	2.444909584	5.421172306	9.25E-08	6.576028672
RP11-279F6.1	0.698354923	1.247690243	5.420163359	9.30E-08	6.57086561
AKAP1	0.278802281	4.206276802	5.419478928	9.34E-08	6.567363673
C8orf88	-0.248003627	0.63344382	-5.417622273	9.43E-08	6.557865946
TGFB2	-0.467580348	1.623327529	-5.417170817	9.45E-08	6.555556957
WNK2	0.33820526	0.93530102	5.416476753	9.49E-08	6.552007474
HIP1R	0.330492358	3.242017499	5.416073296	9.51E-08	6.549944356
RP11-61J19.5	0.235064731	0.89790787	5.415641555	9.53E-08	6.547736759
AC244250.2	-1.050106892	3.65440518	-5.415612496	9.53E-08	6.547588179
GAR1	0.210730721	3.361156147	5.413390702	9.64E-08	6.536230122
C3orf33	0.23027374	2.131194362	5.412770489	9.67E-08	6.533060269
SMAD7	-0.284992025	3.118858658	-5.4120922	9.71E-08	6.529593958
SCPEP1	-0.438104237	5.154638967	-5.409489043	9.84E-08	6.516294437
TRIM11	0.214180979	2.391966433	5.408825007	9.88E-08	6.512902788
NOP2	0.281616664	3.396963131	5.408124789	9.91E-08	6.509326741
BACH1	-0.26265439	2.907514732	-5.406903494	9.98E-08	6.503090503
GPAA1	0.295187793	5.389692711	5.406557649	1.00E-07	6.50132476
PARD3-AS1	0.241824326	0.591260919	5.406461683	1.00E-07	6.500834814
ITGB5	-0.324283824	4.58918711	-5.405951396	1.00E-07	6.498229723
CTD-2531D15.4	-0.212566603	0.263348526	-5.405898006	1.00E-07	6.497957171
C1QBP	0.312694604	4.906154038	5.405818358	1.00E-07	6.497550581

NARS2	0.246827364	2.968775179	5.405243747	1.01E-07	6.494617433
TBCEL	-0.213299699	2.069810864	-5.403941769	1.01E-07	6.4879724
NUP88	0.213489923	3.091589322	5.403594732	1.02E-07	6.486201432
ANPEP	-0.673984232	2.826744563	-5.403534337	1.02E-07	6.485893242
UBE2Q1	0.201690768	4.862638436	5.402520166	1.02E-07	6.480718454
NUTF2	0.225103831	4.584575484	5.402180615	1.02E-07	6.478986093
IL10RB	-0.252864563	4.374662874	-5.402135687	1.02E-07	6.478756884
RIPPLY3	0.360238327	0.744662247	5.401924813	1.02E-07	6.477681079
PHKA1	0.279963999	2.276487211	5.401055616	1.03E-07	6.473247137
RP11-396C23.2	0.224259252	0.891227264	5.400325461	1.03E-07	6.469522964
RECQL4	0.44791996	2.566337583	5.399733009	1.04E-07	6.466501482
NELFE	0.234860411	4.871581148	5.398289407	1.04E-07	6.459140395
KRTAP5-1	0.233316329	0.635314476	5.398067429	1.05E-07	6.458008656
CTSE	-1.191144311	5.058892658	-5.396871447	1.05E-07	6.451911764
FGL1	1.057363001	1.862419532	5.396866306	1.05E-07	6.451885558
ARHGAP4	-0.4213109	3.088979543	-5.396643763	1.05E-07	6.450751207
PITX2	0.468572808	0.812257837	5.395132969	1.06E-07	6.443051462
PLXND1	-0.40028234	4.319857296	-5.394327886	1.07E-07	6.438949146
MN1	-0.286539122	0.986072367	-5.394249976	1.07E-07	6.438552186
TMEM241	0.242542796	2.18221102	5.393970908	1.07E-07	6.437130335
AMBP	0.696325897	1.184368775	5.392850207	1.07E-07	6.431421022
CKMT1A	0.309721588	0.747091604	5.392732833	1.08E-07	6.430823137
OSGIN1	0.606216738	2.433679206	5.391107625	1.08E-07	6.422545697
CLC	-0.350308241	0.376201543	-5.390187709	1.09E-07	6.417861405
CYTH3	-0.405245692	3.248241273	-5.390012741	1.09E-07	6.416970529
NOLC1	0.254368453	4.905869458	5.389100681	1.10E-07	6.412327077
PTDSS2	0.218933133	2.455983	5.387992436	1.10E-07	6.406685752
NHLRC4	-0.283513515	0.798684553	-5.387952855	1.10E-07	6.40648429
MYBBP1A	0.266641928	3.449239994	5.387429282	1.11E-07	6.40381951
RAB40C	0.339642464	3.794546571	5.38712705	1.11E-07	6.402281377
INADL	0.263119789	2.806706106	5.38704254	1.11E-07	6.401851297
IGHV1-68	-0.263853473	0.44463592	-5.386525096	1.11E-07	6.399218104
TYSND1	0.225074272	2.761626117	5.386337322	1.11E-07	6.398262607

TMEM100	-0.514944228	1.414251376	-5.385029663	1.12E-07	6.391609347
EAF2	-0.261630981	1.670221933	-5.384471455	1.12E-07	6.38876967
CHST7	-0.217373935	1.406337237	-5.384330646	1.12E-07	6.388053398
SH3D19	-0.241263556	3.261916702	-5.383441273	1.13E-07	6.383529682
COPS5	0.233761571	3.073514239	5.382745179	1.13E-07	6.379989531
ENDOG	0.253245655	2.239078387	5.380301392	1.15E-07	6.367564285
HSPB7	-0.290560637	1.047033839	-5.379603614	1.15E-07	6.364017407
NDUFA8	0.218706081	5.425346514	5.378839177	1.16E-07	6.360132171
NRSN2-AS1	0.259423562	2.022913902	5.375557071	1.18E-07	6.343456489
RRNAD1	0.244647569	3.641962003	5.375516857	1.18E-07	6.34325223
KIAA1522	0.312216239	5.200547219	5.375186929	1.18E-07	6.341576449
JUP	0.36427819	6.303501938	5.37389098	1.19E-07	6.334994907
ST3GAL6	-0.264812065	1.256152951	-5.37362263	1.19E-07	6.333632255
CAB39	-0.223750445	4.5004006	-5.373460754	1.19E-07	6.332810295
CDC42EP3	-0.400847091	3.066331352	-5.373393306	1.19E-07	6.332467816
FBXO46	0.246278775	2.866775656	5.371426637	1.20E-07	6.322483513
ADSSL1	0.403501444	1.778685286	5.371373098	1.20E-07	6.322211755
CCL24	-0.454437203	1.230565306	-5.371042048	1.20E-07	6.320531425
RP11-1C8.7	0.336715247	0.373091753	5.370325685	1.21E-07	6.316895665
CCL26	-0.305130698	0.705115913	-5.370075782	1.21E-07	6.315627431
CACFD1	0.302820826	3.787703375	5.366577001	1.23E-07	6.297876978
ZNF3	0.20956743	2.898789074	5.366032354	1.24E-07	6.295114731
RP11-180M15.7	-0.231912132	1.085312334	-5.36546703	1.24E-07	6.292247885
GEMIN8P4	0.24361044	1.144609259	5.364269531	1.25E-07	6.286176067
IGLL1	-0.257586479	0.404107115	-5.36354727	1.25E-07	6.282514487
KDELC1	-0.296700834	1.670234213	-5.363124611	1.26E-07	6.280371972
RGS5	-0.440449137	3.626217325	-5.362691295	1.26E-07	6.278175596
PCDH7	-0.471893237	1.403692521	-5.36169556	1.27E-07	6.273129047
ILK	-0.213288362	2.089020067	-5.361530596	1.27E-07	6.272293062
MNAT1	0.205961281	2.42109158	5.360411318	1.27E-07	6.266621529
CTSL	-0.442443017	5.95891289	-5.35910328	1.28E-07	6.259994858
IGKV2OR2-1	-0.357630772	0.5961177	-5.359021615	1.28E-07	6.259581183
GGCT	0.342335135	5.006140613	5.357627798	1.29E-07	6.252521624

IRS2	0.522676996	2.732252958	5.356848277	1.30E-07	6.248574132
DCAF16	0.239365559	3.119445298	5.35109064	1.34E-07	6.219433337
RARB	-0.290095434	1.503376824	-5.350498646	1.34E-07	6.216438689
FENDRR	-0.259209088	0.578070082	-5.350241125	1.34E-07	6.215136094
DLG4	-0.253221584	1.587349092	-5.349615138	1.35E-07	6.211969951
WASH6P	0.295728711	1.337947027	5.348309726	1.36E-07	6.205368449
COMMD4	0.206283456	3.058001312	5.344635749	1.38E-07	6.186796756
MCF2L	0.318035072	1.293798492	5.343987024	1.39E-07	6.183518679
RP5-967N21.11	0.288972341	1.320127728	5.341323298	1.41E-07	6.170062295
CACNA2D1	-0.3052462	0.994768197	-5.340396845	1.41E-07	6.165383524
LAMC2	-0.713683893	4.804608861	-5.338210252	1.43E-07	6.154343659
SLC39A4	0.432931671	3.347131964	5.337341398	1.44E-07	6.149958029
KIF21A	0.299314841	2.006778325	5.336201028	1.45E-07	6.144202861
DVL1	0.32950477	4.582307151	5.333679302	1.46E-07	6.131480221
RP3-449M8.6	-0.259215483	0.70513672	-5.333105202	1.47E-07	6.128584515
APEX1	0.244813216	6.285867305	5.331588425	1.48E-07	6.120935365
RP11-284F21.9	0.502954603	0.890425157	5.329443341	1.50E-07	6.110120967
CCS	0.24636127	3.874385443	5.328987959	1.50E-07	6.107825665
DGAT1	0.315251636	3.884238039	5.325465134	1.53E-07	6.09007518
TSTA3	0.341036053	4.934318342	5.325153845	1.53E-07	6.088507188
RAB2A	0.222364959	4.962756994	5.324680409	1.53E-07	6.086122604
CLIC5	-0.539721268	1.78553885	-5.321597515	1.56E-07	6.070599433
RP11-26F2.1	0.223817305	0.360826722	5.321337423	1.56E-07	6.069290168
SMPDL3A	-0.465933631	3.441312812	-5.317694179	1.59E-07	6.050956629
TRAM2	-0.291506015	3.045953709	-5.317608886	1.59E-07	6.050527547
PPARG	-0.458504111	2.101904923	-5.316503925	1.60E-07	6.044969455
ACTR2	-0.202553868	6.224788955	-5.314963313	1.61E-07	6.0372217
IGKV2-18	-0.276115223	0.410441691	-5.314727704	1.62E-07	6.036036998
NRSN2	0.296395227	3.949166593	5.312936408	1.63E-07	6.027031425
RNF13	-0.222993956	4.28368364	-5.31085416	1.65E-07	6.016566524
ZMAT3	-0.230854457	2.056668797	-5.309712578	1.66E-07	6.010830743
SPTBN2	0.377666127	2.701789327	5.307816046	1.68E-07	6.001304221
ABCG2	-0.228524337	0.910326973	-5.306841508	1.68E-07	5.996410175

PPAP2C	0.404428604	3.63501861	5.306526139	1.69E-07	5.994826589
IGLV5-37	-0.732388806	1.569925818	-5.306341123	1.69E-07	5.993897595
C14orf1	0.239055305	4.472882148	5.305890846	1.69E-07	5.991636803
TRPS1	-0.274947816	1.249589998	-5.304830483	1.70E-07	5.986313513
HIBCH	0.205727805	2.143544911	5.301080562	1.74E-07	5.967495574
MIER1	-0.205980668	2.788912021	-5.300955038	1.74E-07	5.966865874
GSDMA	-0.241093942	0.653310922	-5.300881512	1.74E-07	5.966497029
C19orf66	-0.244742969	2.743918572	-5.300624354	1.74E-07	5.965207035
ADRBK2	-0.313334774	2.145018722	-5.300408493	1.74E-07	5.964124242
OLMALINC	0.302813945	2.096623284	5.299476431	1.75E-07	5.959449319
CTD-2139B15.5	0.551861741	0.710568058	5.299173634	1.75E-07	5.957930744
PRDM8	-0.262470589	0.883113822	-5.298421812	1.76E-07	5.954160575
ZNF513	0.263663513	3.201125407	5.298083386	1.76E-07	5.952463624
OR7E38P	0.370370278	2.470699042	5.296294901	1.78E-07	5.943497321
RP11-400K9.4	-0.271159532	0.646344186	-5.295057247	1.79E-07	5.93729411
WDR5	0.212073185	3.73375783	5.295035865	1.79E-07	5.937186956
PHLPP1	0.247067005	1.652798871	5.29451151	1.80E-07	5.934559247
TIMELESS	0.351686056	3.080790936	5.293755896	1.80E-07	5.930773042
CTD-3065J16.9	0.21728649	0.998324595	5.29347108	1.80E-07	5.92934602
AP3S1	-0.202681584	3.965992226	-5.293221867	1.81E-07	5.928097438
SNX18	-0.291983662	3.141712334	-5.292773469	1.81E-07	5.925851049
PRDX6	0.236481132	6.710944477	5.291842167	1.82E-07	5.921185946
AFMID	0.255848879	3.107701139	5.290029401	1.84E-07	5.912107496
KRT18	0.38704654	7.754976288	5.288967808	1.85E-07	5.90679226
RPS6KA1	-0.23767862	4.037876363	-5.288708513	1.85E-07	5.905494154
HIVEP2	-0.238222236	2.390036212	-5.288700623	1.85E-07	5.905454654
S100A8	-0.810510548	3.882928315	-5.288257987	1.85E-07	5.903238825
TOMM40	0.285771479	4.169653041	5.286748394	1.87E-07	5.895683063
ATP6V1C2	0.508709833	1.588440093	5.286725517	1.87E-07	5.895568575
INPPL1	0.30573672	4.617279951	5.286011214	1.88E-07	5.891994057
CELSR3-AS1	0.209461838	0.913727685	5.284546099	1.89E-07	5.884663675
VAMP1	-0.29603359	1.715053732	-5.281322108	1.92E-07	5.86853953
COL5A3	-0.393484955	1.770430388	-5.280064562	1.93E-07	5.862252548

MAP4	-0.223254867	4.03464543	-5.277082126	1.96E-07	5.847347491
RP4-533D7.5	0.237103846	0.573770774	5.276756936	1.97E-07	5.845722774
FAM83G	0.277715843	2.131398274	5.273871479	2.00E-07	5.831310337
MSI2	0.238460665	2.227068523	5.273198678	2.00E-07	5.827950807
THEM5	0.478063303	2.670324009	5.269678549	2.04E-07	5.810379824
IGHD5-12	-0.297617565	0.33224088	-5.269251548	2.05E-07	5.808249132
SRP9	0.243486312	6.60992336	5.268684401	2.05E-07	5.805419361
STAT2	-0.262805018	4.589621805	-5.261888653	2.12E-07	5.771533273
AL928761.1	-0.596624532	1.808711942	-5.261367757	2.13E-07	5.768937507
IL13RA1	-0.267545643	5.314623827	-5.258515736	2.16E-07	5.754729196
BCAT1	-0.579940387	2.636089434	-5.254689471	2.20E-07	5.735678181
CHCHD1	0.22703651	4.467774637	5.254232459	2.21E-07	5.733403543
IGKV2-29	-0.894735659	1.713994308	-5.25330423	2.22E-07	5.728784106
ACY1	0.20429657	1.335844065	5.252831236	2.23E-07	5.726430484
TTC6	0.224783402	0.537918206	5.251326368	2.24E-07	5.718943494
RNF213	-0.3655241	3.742388568	-5.250338086	2.25E-07	5.71402766
RAB27A	-0.324874372	3.702179855	-5.249631046	2.26E-07	5.710511263
NOXA1	0.467330028	3.075748959	5.249462033	2.26E-07	5.709670758
FAM229B	-0.322922395	1.741615692	-5.247293894	2.29E-07	5.698890677
RPS20P14	0.32201062	1.991862755	5.246924278	2.29E-07	5.697053327
COLGALT1	-0.284197815	4.671183083	-5.246900127	2.29E-07	5.696933279
BAIAP2L1	0.314566969	4.267316668	5.245754344	2.31E-07	5.691238389
MRPL15	0.320858906	5.392918829	5.245462588	2.31E-07	5.689788451
PAPSS1	-0.201732225	4.210352965	-5.24522718	2.31E-07	5.688618601
IGHV3-75	-0.310029823	0.601024582	-5.243538193	2.33E-07	5.680226602
MEGF6	-0.45550933	2.636434892	-5.241914483	2.35E-07	5.672161229
INTS8	0.23022886	2.746098312	5.24157481	2.36E-07	5.67047427
SYCP2L	0.205393872	0.359688901	5.240918741	2.37E-07	5.667216235
BTBD6	0.250593711	4.808894554	5.239210526	2.39E-07	5.658734956
ACN9	0.286652816	2.902978942	5.236306362	2.42E-07	5.64432148
COX5B	0.294369937	5.906419035	5.236054339	2.43E-07	5.643071016
IGLL3P	-0.259819077	0.423331288	-5.233657919	2.46E-07	5.63118339
AC098614.2	-0.276792488	1.560046892	-5.232630584	2.47E-07	5.626088712

FAM126A	-0.337699363	1.825551613	-5.229593866	2.51E-07	5.611034482
USMG5	0.2676919	5.782330497	5.229479811	2.51E-07	5.610469221
FHL5	-0.27258264	0.909341691	-5.22880347	2.52E-07	5.607117459
MIEN1	0.316149505	4.33585804	5.228739191	2.52E-07	5.606798929
GPR110	-0.737364227	2.109214124	-5.228726252	2.52E-07	5.606734809
SOD1	0.259929316	6.295060092	5.228470844	2.52E-07	5.605469195
SCNN1A	0.553964434	5.593277213	5.225866762	2.56E-07	5.592568433
TMEM43	-0.26192646	5.140031274	-5.225236996	2.56E-07	5.589449405
B3GNT2	-0.250821438	3.821938094	-5.224930288	2.57E-07	5.587930498
SKI	-0.270522345	4.121412671	-5.224689892	2.57E-07	5.586740047
ANK2	-0.228268316	0.651325106	-5.222936585	2.60E-07	5.57805906
TNK1	0.212995468	2.186253511	5.221102186	2.62E-07	5.568979372
INHA	0.764072775	1.164772463	5.217115465	2.67E-07	5.549256218
RAE1	0.218112462	2.801304752	5.216484739	2.68E-07	5.546137119
CHKA	0.351249104	3.251949306	5.21572994	2.69E-07	5.542404892
MTX1	0.213046053	2.913670487	5.214595294	2.71E-07	5.536795362
DDX26B	-0.223520818	1.259916501	-5.214143	2.71E-07	5.534559586
MEN1	0.225973867	3.581122652	5.214075854	2.72E-07	5.534227688
GDF5	-0.26148052	0.491459283	-5.21392454	2.72E-07	5.533479762
NOP14	0.200055982	3.88805777	5.213798462	2.72E-07	5.532856591
RAB25	0.387902207	6.196045292	5.213426059	2.72E-07	5.53101597
CRIM1	-0.370968786	3.504191485	-5.213214349	2.73E-07	5.529969637
ECM1	-0.658388336	3.816304512	-5.212141521	2.74E-07	5.52466799
TAF1A-AS1	0.203363725	1.018417951	5.211136683	2.76E-07	5.519703217
SH3PXD2A	-0.298128946	3.498572661	-5.210957535	2.76E-07	5.518818164
NFKBID	-0.266594342	1.555760553	-5.210207172	2.77E-07	5.515111392
BIRC7	-0.355349934	1.000797234	-5.209859735	2.77E-07	5.513395227
PARP14	-0.312057803	4.049530893	-5.209527457	2.78E-07	5.511754036
STX4	-0.212827435	3.722467933	-5.208225852	2.80E-07	5.505326033
RP11-103B5.4	0.309097255	0.666367794	5.207768362	2.80E-07	5.503067048
COL4A3BP	-0.226304797	2.664752843	-5.207519389	2.81E-07	5.501837752
TATDN1	0.232243104	2.244181562	5.207169996	2.81E-07	5.500112722
LGSN	0.603369899	1.693741411	5.206070965	2.83E-07	5.494687243

EMC9	0.279808164	3.085431881	5.204878365	2.85E-07	5.48880101
SQLE	0.392133027	3.885578094	5.204298622	2.85E-07	5.485940051
PTH1R	-0.243633545	0.84394241	-5.204234015	2.86E-07	5.485621241
TRABD2A	-0.283682888	0.845603606	-5.203678035	2.86E-07	5.482877847
ILF3	0.2122938	4.579763369	5.201996646	2.89E-07	5.474582906
UQCRB	0.232225351	4.0134416	5.201510868	2.90E-07	5.472186821
CXCL6	-0.538873494	1.109652588	-5.2012313	2.90E-07	5.47080795
IGHV3-20	-0.735269473	2.423002541	-5.201209596	2.90E-07	5.470700908
PDAP1	0.226984395	5.327315577	5.200677206	2.91E-07	5.468075279
GPR15	-0.324871777	0.662264511	-5.198178032	2.95E-07	5.45575313
ZMYND15	-0.267534379	1.480388982	-5.196704901	2.97E-07	5.448492361
APBB2	-0.242662861	1.926317176	-5.195613058	2.98E-07	5.443112071
UBAP2L	0.225244379	4.766546058	5.19467905	3.00E-07	5.438510355
C20orf194	-0.228628952	1.80875115	-5.194090286	3.01E-07	5.435609989
TFEB	-0.261264574	2.338491747	-5.19366209	3.01E-07	5.433500794
CTTNBP2NL	-0.217640257	2.697562602	-5.19341726	3.02E-07	5.432294886
NPR1	-0.385187077	1.630585111	-5.192754969	3.03E-07	5.429033038
AC010894.3	0.21374965	0.800102505	5.191946513	3.04E-07	5.425051822
PSME1	-0.249837763	6.777108282	-5.190813129	3.06E-07	5.419471441
IL6	-0.523138397	1.789290781	-5.190030435	3.07E-07	5.415618368
ARPC5	-0.211831241	4.452499648	-5.189741686	3.07E-07	5.414197041
SLCO2A1	-0.519729809	3.406675338	-5.188925093	3.09E-07	5.410177846
UQCC3	0.299889065	3.291121545	5.188262786	3.10E-07	5.406918453
RP4-758J18.10	0.390899471	2.062148956	5.187777513	3.11E-07	5.404530527
ALG8	0.227233445	3.671516454	5.187113591	3.12E-07	5.401263828
RP11-794G24.1	0.201766188	0.480608276	5.185992909	3.13E-07	5.39575059
RP11-532F12.5	0.38067568	3.081685803	5.185067389	3.15E-07	5.391198265
GS1-44D20.1	-0.223858893	1.070321792	-5.184902594	3.15E-07	5.390387767
ELF1	-0.226614299	4.132957304	-5.181948719	3.20E-07	5.375863928
SPATA13	-0.242844337	2.236017186	-5.18107692	3.21E-07	5.371578815
GCNT2	0.259575215	2.144370262	5.178121238	3.26E-07	5.357055714
ACTN1	-0.342219692	4.250928589	-5.176723031	3.29E-07	5.350188046
EFNB2	-0.439716318	3.398213055	-5.175486817	3.31E-07	5.344117436

SSBP2	-0.224812945	1.308113543	-5.174150403	3.33E-07	5.337556244
S100P	1.309577943	5.377488848	5.173563345	3.34E-07	5.334674534
ZNF474	-0.240666851	0.4407323	-5.169360961	3.41E-07	5.314054724
RP5-1033H22.2	0.30094426	0.99973707	5.168685548	3.42E-07	5.310742079
CENPO	0.23771541	1.725511041	5.168559867	3.42E-07	5.310125707
MPST	0.293025241	3.324153475	5.167870797	3.44E-07	5.306746556
ADRM1	0.25174405	5.64353829	5.166414278	3.46E-07	5.299605225
TMEM45A	-0.527393368	2.210118825	-5.166330255	3.46E-07	5.299193314
MIR621	0.349634922	3.693494379	5.165536728	3.48E-07	5.295303456
TMEM81	0.20161646	1.615865194	5.165140117	3.48E-07	5.293359478
MAMLD1	-0.294695423	1.085176588	-5.162208323	3.54E-07	5.278993512
YRDC	0.211521585	3.679088163	5.162080699	3.54E-07	5.278368316
PIGM	0.232303302	2.951810248	5.161693393	3.55E-07	5.276471081
CD38	-0.429743706	1.891452127	-5.161487602	3.55E-07	5.27546306
STX2	-0.243244479	2.331774918	-5.160483474	3.57E-07	5.270545074
IGLV3-22	-0.334440899	0.406585439	-5.159408068	3.59E-07	5.265278936
MYEF2	0.233065142	1.18437262	5.159051914	3.59E-07	5.263535113
RCAN2	-0.461539541	2.431195146	-5.158497784	3.60E-07	5.260822157
C11orf98	0.23792445	2.489937858	5.154804756	3.67E-07	5.242748205
NDUFB4	0.246752115	5.408560635	5.154417902	3.68E-07	5.240855583
NTHL1	0.317475536	3.344196352	5.152930746	3.71E-07	5.233581109
MRPL21	0.242764434	3.522556501	5.151561025	3.73E-07	5.226882736
ALDH3A2	0.461283358	4.605925942	5.150067882	3.76E-07	5.219582612
SLBP	0.233106801	4.836165422	5.150043333	3.76E-07	5.219462607
C2orf40	-0.417228521	0.831471875	-5.149309581	3.78E-07	5.215875934
CTB-63M22.1	0.424652546	3.329012145	5.147895301	3.80E-07	5.208964048
ABCG1	-0.36342653	2.801646113	-5.147745014	3.81E-07	5.208229666
ZBTB38	-0.229246036	2.858495559	-5.146976816	3.82E-07	5.204476133
CSE1L	0.285821378	4.665317491	5.146878789	3.82E-07	5.203997191
AF131215.9	-0.206250447	0.628727347	-5.144729138	3.86E-07	5.193496528
MIR7152	-0.211339543	0.31100051	-5.142558043	3.91E-07	5.18289511
U47924.27	0.436132161	2.844819956	5.140546979	3.95E-07	5.173078707
FAM111A	-0.255350439	2.873883129	-5.13992744	3.96E-07	5.170055312

RASGRF1	-0.434606745	1.058150846	-5.139009232	3.98E-07	5.165574993
MRTO4	0.231951217	4.110363754	5.137475119	4.01E-07	5.158091015
IGHE	-0.374821622	0.665352081	-5.137473846	4.01E-07	5.158084804
NRTN	0.282869146	0.942106906	5.136743441	4.02E-07	5.154522325
WFDC21P	-0.564316736	2.212394247	-5.13604646	4.04E-07	5.15112329
LAMTOR5	0.258041325	4.895545223	5.134383879	4.07E-07	5.143016897
TRPT1	0.235687434	3.5712561	5.133445007	4.09E-07	5.138440197
MYO7A	-0.256392413	1.415449699	-5.132577618	4.11E-07	5.134212621
RP11-53B5.1	0.429991172	0.574101464	5.130243907	4.16E-07	5.122841519
LINC00884	0.218634202	0.80480269	5.130004679	4.16E-07	5.121676135
FZD7	-0.331485367	2.351414576	-5.129206248	4.18E-07	5.117786976
RGL2	0.27892826	4.325660326	5.126712077	4.23E-07	5.105641374
RP13-582O9.5	0.286361682	1.665444468	5.126697743	4.23E-07	5.105571587
GALE	0.316410212	4.335274519	5.121713267	4.34E-07	5.081315189
GTPBP3	0.222573224	2.233541267	5.1210224	4.35E-07	5.077954835
CTD-2521M24.9	-0.283036984	1.755135839	-5.12091891	4.36E-07	5.0774515
RNF186	0.358991411	0.578341677	5.120216598	4.37E-07	5.074035958
MIR3671	-0.26832249	0.806913605	-5.118000579	4.42E-07	5.063261597
SPATS2L	-0.341087449	3.7160389	-5.117906225	4.42E-07	5.062802939
TWIST1	-0.396999788	0.971281904	-5.115876876	4.47E-07	5.052940018
EFNA3	0.351205259	1.887255257	5.115728871	4.47E-07	5.052220832
SNHG11	0.217507269	2.090024262	5.113946555	4.51E-07	5.043561652
IKBIP	-0.251294647	2.597393846	-5.11388346	4.51E-07	5.043255161
CTHRC1	-0.623566588	4.894822281	-5.113146406	4.53E-07	5.039675103
AKR7L	0.275083173	0.50331417	5.111589251	4.57E-07	5.032113129
XPO5	0.247417958	3.02277189	5.111365061	4.57E-07	5.031024572
CD109	-0.481796747	1.933883274	-5.111055297	4.58E-07	5.029520579
DENND5A	-0.208733912	3.116420364	-5.108350776	4.64E-07	5.016392845
SLC50A1	0.348838897	5.472602478	5.105394015	4.71E-07	5.002047895
CRLF2	-0.239428856	0.474103244	-5.102152463	4.79E-07	4.986329844
UBE2T	0.471912128	3.978248087	5.101390479	4.81E-07	4.982636345
KLC2	0.246165681	2.954919431	5.100154617	4.84E-07	4.976646913
PFAS	0.233917652	2.333775718	5.098857369	4.87E-07	4.970361388

FAM174B	0.392406716	3.19973862	5.098093716	4.89E-07	4.966661951
MPDU1	0.241168624	4.44123426	5.097042163	4.91E-07	4.961568632
RP11-481H12.1	-0.316053044	1.308788766	-5.096780999	4.92E-07	4.960303797
RNU6-638P	-0.208520853	0.476677161	-5.096054866	4.94E-07	4.956787401
TRIM45	0.242093083	1.253627772	5.09537029	4.95E-07	4.953472663
CBLC	0.480742087	3.516402692	5.094220684	4.98E-07	4.947907135
STK35	0.233514068	3.057570319	5.094036139	4.99E-07	4.947013813
C6orf48	0.301770983	5.758048575	5.093887071	4.99E-07	4.946292242
C5orf30	0.28863778	2.016100712	5.092989559	5.01E-07	4.941948209
PARD3B	-0.225389343	1.295952496	-5.091579336	5.05E-07	4.935124002
MIR6503	-0.200703462	0.36514894	-5.090541538	5.08E-07	4.930103081
FAXDC2	-0.536449941	2.434816056	-5.090448252	5.08E-07	4.929651806
TRMT61A	0.248795769	2.988788028	5.089915997	5.09E-07	4.92707713
GPIHBP1	-0.399934374	1.12167672	-5.088481384	5.13E-07	4.920138685
LINC01123	0.204931037	0.666625785	5.087738553	5.15E-07	4.916546708
NFXL1	0.203658388	1.996872145	5.08766367	5.15E-07	4.916184636
TROAP	0.448128242	1.938698966	5.086679437	5.17E-07	4.911426143
RP11-417E7.2	-0.34524354	0.865335497	-5.085888404	5.20E-07	4.907602319
TMEM184A	0.39575434	2.618952927	5.084592932	5.23E-07	4.901341213
IFI27	-0.688049222	5.473143106	-5.084555023	5.23E-07	4.901158019
UAP1L1	-0.28808777	2.016372605	-5.083867286	5.25E-07	4.897834743
GOT1	0.323688679	4.520012204	5.081618758	5.31E-07	4.886972262
RNA5SP498	-0.242908895	0.635750998	-5.081238575	5.32E-07	4.885136051
CTTN	0.257585146	5.485521452	5.081236966	5.32E-07	4.885128284
AKR1C1	1.093872527	2.578163458	5.080877888	5.33E-07	4.883394122
CHML	0.40547159	2.584082155	5.079248222	5.37E-07	4.875525058
EML1	-0.241186991	1.181856795	-5.078636153	5.39E-07	4.872570189
RP11-474D1.3	0.383079413	0.35885363	5.075005637	5.49E-07	4.855049834
DDX54	0.238022325	4.007270492	5.07334884	5.53E-07	4.847058115
HPDL	0.365650535	0.932233984	5.070592815	5.61E-07	4.833769383
TFCP2L1	0.563194601	2.659599813	5.067024107	5.71E-07	4.816571801
BCAN	0.211508329	0.41488046	5.066725262	5.72E-07	4.815132163
TBX2	-0.352098943	2.209219382	-5.066085722	5.74E-07	4.812051543

RP11-325K4.2	-0.22848899	1.024451454	-5.065641189	5.75E-07	4.809910462
ITPRIPL2	-0.284894084	3.201813896	-5.065450231	5.75E-07	4.80899077
SH3RF3	-0.249859308	1.4218589	-5.065040989	5.77E-07	4.807019887
PXMP2	0.244719621	2.807125715	5.063564582	5.81E-07	4.799910792
HAL	0.480707662	1.148370425	5.062643112	5.84E-07	4.79547474
IARS2	0.229756776	5.237333999	5.061602109	5.87E-07	4.790464121
CHI3L2	-0.552681756	2.129645197	-5.06101863	5.88E-07	4.787656089
PARD3	0.26545938	3.379135793	5.06065168	5.89E-07	4.785890267
EPHB2	-0.411432423	1.818940749	-5.060495885	5.90E-07	4.78514059
HSP90AB1	0.239425259	8.84825913	5.056719858	6.01E-07	4.766976961
SPSB2	0.361105094	3.372111074	5.055706604	6.04E-07	4.762105039
DGKA	-0.277180649	1.993394658	-5.055298092	6.05E-07	4.760141083
EIF3B	0.253642949	4.764957706	5.054597276	6.07E-07	4.756772184
AC115522.3	-0.229684576	0.607177138	-5.054283718	6.08E-07	4.755265015
PTPN21	-0.257424512	1.741388559	-5.053439067	6.11E-07	4.751205472
TRAP1	0.216736048	3.666759138	5.0515808	6.17E-07	4.742276464
SRXN1	0.385621387	1.105850258	5.051343714	6.17E-07	4.741137475
MRPL9	0.224890752	4.891007985	5.049851344	6.22E-07	4.733969058
ATP5G3	0.231630583	4.46633994	5.049405223	6.23E-07	4.73182654
IFT81	0.238055983	1.842982864	5.048766855	6.25E-07	4.728761045
TJP3	0.391615425	3.449694821	5.04798444	6.28E-07	4.725004303
BANF1	0.23160859	6.069552187	5.045942755	6.34E-07	4.715203682
TLR5	-0.276874986	1.78175495	-5.043828969	6.41E-07	4.705060727
SDPR	-0.526484695	3.327873653	-5.042244585	6.46E-07	4.697460612
CEP85	0.241159848	2.339362667	5.040533969	6.51E-07	4.689257397
RANBP1	0.266558361	3.803478904	5.038930243	6.57E-07	4.681569046
IGHJ1P	-0.220615744	0.260723662	-5.037671539	6.61E-07	4.675536303
ADD3	-0.411020693	3.479147865	-5.03715696	6.62E-07	4.673070406
PIP5K1A	0.228232444	4.021779329	5.036790275	6.64E-07	4.671313367
MVK	0.202685686	2.206203694	5.034707036	6.71E-07	4.661333336
CTA-384D8.31	-0.523622289	1.353714142	-5.034057065	6.73E-07	4.658220325
FEM1C	-0.213190482	2.793630197	-5.033737913	6.74E-07	4.65669189
TRIB3	0.396215382	3.334295185	5.033530387	6.75E-07	4.65569809

MIR559	0.366590294	1.569036805	5.031926442	6.80E-07	4.648018339
C1orf43	0.25013733	6.75372333	5.03056496	6.85E-07	4.641501239
CTD-3074O7.5	0.213535623	1.269092509	5.029631636	6.88E-07	4.637034559
PRPSAP2	0.216304198	2.791413303	5.028548255	6.91E-07	4.631850675
HSPG2	-0.468184748	4.009560672	-5.028338919	6.92E-07	4.630849136
PDGFD	-0.348399677	1.739924303	-5.028247106	6.92E-07	4.630409878
GNA12	-0.267300022	3.962578468	-5.026950517	6.97E-07	4.624207475
PLXDC1	-0.247043138	1.402678171	-5.02526362	7.03E-07	4.616140144
CDKN1A	-0.352758273	5.210840402	-5.023473111	7.09E-07	4.607579972
RP11-334C17.6	0.283844687	1.384663397	5.022885108	7.11E-07	4.604769415
C11orf63	-0.219923997	1.046222985	-5.022741419	7.12E-07	4.604082652
HSPB6	-0.458605167	2.026134686	-5.021167375	7.17E-07	4.596560636
TICRR	0.210845164	0.7188898	5.020214123	7.21E-07	4.59200629
AKR1C3	0.927961033	3.652295664	5.019816332	7.22E-07	4.590105997
MME	-0.394320994	1.139084873	-5.018955932	7.25E-07	4.585996229
PQLC3	-0.26593878	3.969821766	-5.018540906	7.27E-07	4.584014055
RP11-123B3.2	0.328125759	0.528743212	5.017557623	7.30E-07	4.579318458
PBK	0.476664746	2.127776342	5.017106372	7.32E-07	4.57716382
AP000251.3	0.219614051	0.462939898	5.01662314	7.33E-07	4.574856675
RASSF1	-0.200269274	3.04295879	-5.014100874	7.43E-07	4.562817607
NIFK	0.204650156	4.126689135	5.013649031	7.44E-07	4.560661488
PUS7	0.257403465	2.58691651	5.01344811	7.45E-07	4.559702777
SLC3A2	0.273993781	5.063464116	5.013279656	7.46E-07	4.558899017
SLC44A5	0.445718317	1.32429203	5.013076911	7.46E-07	4.557931675
NAT9	0.225773569	3.386361491	5.012458375	7.49E-07	4.554980711
RP11-1C8.4	0.209360736	0.225524267	5.012103501	7.50E-07	4.553287793
AKR1B1	-0.499130376	4.316470753	-5.012080633	7.50E-07	4.553178706
TMEM106C	0.33690853	4.458800374	5.011798198	7.51E-07	4.551831445
RP11-496H1.1	0.259943466	0.819020982	5.011015816	7.54E-07	4.548099706
PGM5	-0.318687848	1.08296331	-5.010203419	7.57E-07	4.54422536
ZNF687	0.233156477	3.20222596	5.010026018	7.58E-07	4.543379404
SERPINE2	-0.422864259	1.893263205	-5.009241934	7.61E-07	4.539640743
PLTP	-0.483313934	5.704528118	-5.008409518	7.64E-07	4.535672204

MMAB	0.200518105	2.402679645	5.008052216	7.65E-07	4.533968951
GDAP1	0.307501145	1.702396253	5.007587668	7.67E-07	4.53175462
TLE6	0.270137497	1.311366995	5.007474895	7.67E-07	4.531217103
EGR1	-0.568825182	5.693159421	-5.005897752	7.73E-07	4.523700983
SLC5A6	0.227376122	2.897468043	5.004954103	7.77E-07	4.519204903
FA2H	0.541198099	2.707318375	5.004560306	7.79E-07	4.517328855
DSCAM-AS1	0.834742333	0.966957947	5.004296459	7.80E-07	4.516071963
PKIG	-0.365380794	3.830981527	-5.000239976	7.95E-07	4.496755585
NME2P1	0.222954951	1.018810592	4.996893728	8.09E-07	4.480831897
FMO4	-0.253388017	1.4845404	-4.996835261	8.09E-07	4.480553759
SOAT1	-0.236327059	3.896217264	-4.990918455	8.33E-07	4.452421524
RP11-465N4.5	0.306089514	2.058362637	4.990372278	8.35E-07	4.449826173
MCAM	-0.384311425	3.550863642	-4.989639175	8.38E-07	4.446342982
PGD	0.429959294	6.201353274	4.988940825	8.41E-07	4.443025341
DKC1	0.234920529	4.251790964	4.987016484	8.49E-07	4.433885581
FAM167B	-0.279014303	2.400996604	-4.985004495	8.57E-07	4.424332942
OAZ1	-0.214524253	7.021492687	-4.984385606	8.60E-07	4.421395243
SHROOM4	-0.325077315	1.6044678	-4.983961905	8.62E-07	4.419384244
SUN2	-0.260898755	4.432558927	-4.983937404	8.62E-07	4.419267958
IGHD3-16	-0.38378175	0.619692778	-4.983683316	8.63E-07	4.418062066
TRAFD1	-0.208925186	4.051252986	-4.983440012	8.64E-07	4.416907409
AIFM1	0.206773935	3.985043751	4.983408933	8.64E-07	4.416759916
EIF3H	0.21712857	5.27742799	4.983265911	8.65E-07	4.416081199
ANKEF1	0.211512296	1.860054765	4.9819662	8.70E-07	4.409914153
PRG4	-0.470863072	0.995944386	-4.98136767	8.73E-07	4.407074659
DTYMK	0.290474552	3.757308817	4.980427592	8.77E-07	4.402615442
NDUFS5	0.251318853	7.630433312	4.977399235	8.90E-07	4.388255744
EFNA1	0.421384408	6.138980589	4.976417807	8.94E-07	4.383603762
UBE2M	0.206336895	5.016056313	4.975801815	8.97E-07	4.380684371
ATF5	-0.304228995	4.036155736	-4.975046436	9.00E-07	4.377104828
CEP70	0.277447688	2.88254125	4.974139513	9.05E-07	4.372807809
SLC48A1	0.226056381	2.898368005	4.972129218	9.13E-07	4.363285513
TLDC2	-0.255616058	1.028677458	-4.970891212	9.19E-07	4.357423103

VIM-AS1	-0.283531775	1.430170497	-4.970735305	9.20E-07	4.356684921
MST1	0.270442067	1.079000149	4.967929221	9.33E-07	4.343402361
FAM110A	0.250109309	3.042060431	4.964873627	9.47E-07	4.328946466
GCA	-0.237490111	3.315487272	-4.963675883	9.52E-07	4.323282182
UTRN	-0.31486177	2.960516338	-4.963491417	9.53E-07	4.322409929
DUSP10	-0.340740167	2.946402318	-4.961318424	9.63E-07	4.312137078
SLC29A4	0.543087881	2.073056106	4.959950215	9.70E-07	4.305670942
CSNK2A1	0.208956049	4.027335114	4.958968465	9.74E-07	4.301032198
SLC12A6	-0.208104985	2.150708598	-4.9586723	9.76E-07	4.299632988
CD81	-0.265022244	6.103732452	-4.957167571	9.83E-07	4.292525182
C8orf59	0.269717873	3.675100553	4.956841367	9.85E-07	4.290984567
SNHG6	0.350260524	5.412832774	4.956171496	9.88E-07	4.287821153
CILP2	-0.456537872	1.635011974	-4.954238844	9.97E-07	4.278696518
FTL	-0.366747926	12.07011372	-4.95417881	9.98E-07	4.27841313
CDA	-0.787376201	3.067197339	-4.953763529	1.00E-06	4.276452907
AGPAT6	0.284400001	3.612588554	4.952230777	1.01E-06	4.269219242
PAPOLA	0.203959808	4.040732439	4.951529362	1.01E-06	4.26590966
PPP2R5B	-0.231976667	2.822602758	-4.950321966	1.02E-06	4.26021364
GYG1	-0.220066663	3.727666523	-4.947132663	1.03E-06	4.245173803
FURIN	0.508138169	5.851756232	4.946818229	1.03E-06	4.243691498
PODNL1	-0.489204833	1.843295482	-4.945949808	1.04E-06	4.239598031
MRPL17	0.212561151	4.621249066	4.94552819	1.04E-06	4.237610889
RAB23	-0.237386215	1.571523171	-4.94453347	1.05E-06	4.232923252
UNG	0.263120802	4.028186411	4.944091149	1.05E-06	4.230839081
CCT5	0.305517243	5.297475489	4.943332322	1.05E-06	4.227263955
CCBE1	-0.368231453	0.802590443	-4.943174197	1.05E-06	4.226519029
PLIN3	-0.281536473	4.823484956	-4.942355542	1.06E-06	4.222662704
RP3-395M20.9	-0.242717923	0.617344647	-4.940326828	1.07E-06	4.213108808
EIF5A	0.253713068	6.945088123	4.939930727	1.07E-06	4.211243848
CD207	-0.721023634	1.836775867	-4.939314235	1.07E-06	4.208341496
SGCB	-0.273109816	2.800216607	-4.939229359	1.07E-06	4.207941941
PGBD5	-0.27027225	0.726817283	-4.938684788	1.08E-06	4.205378493
C5orf15	-0.227485269	5.264839529	-4.937915514	1.08E-06	4.201757747

RP11-474D1.4	0.222898147	0.179572134	4.937275389	1.08E-06	4.198745253
APH1B	-0.235792445	1.885279999	-4.936664074	1.09E-06	4.195868675
GOT2	0.205245776	4.649217734	4.936641113	1.09E-06	4.195760636
GALNT5	-0.482514841	2.244129418	-4.93442447	1.10E-06	4.185332839
CMTM6	-0.208648638	4.949995029	-4.933168987	1.11E-06	4.179428531
APCDD1	-0.34239455	1.841503718	-4.932634383	1.11E-06	4.176914798
MIR4664	0.435103704	2.579698351	4.93147328	1.11E-06	4.171456092
SLC25A15	0.264218639	2.068048105	4.931162277	1.12E-06	4.169994166
SULT1C4	-0.223394257	0.718987292	-4.926732593	1.14E-06	4.149180726
SOX17	-0.226559316	0.902857231	-4.92668411	1.14E-06	4.148953014
CYP4F11	0.621900423	1.21967133	4.924792577	1.15E-06	4.140070651
C14orf2	0.236370716	4.018291004	4.924400808	1.15E-06	4.138231345
PRUNE	0.226287052	3.887951913	4.923145586	1.16E-06	4.13233914
RP4-694B14.8	0.236902144	1.057780384	4.921630696	1.17E-06	4.12522982
FIGF	-0.475816269	1.218521176	-4.920364045	1.18E-06	4.119287002
LAPTM4B	0.411339418	6.510791195	4.920107203	1.18E-06	4.118082131
ZHX2	-0.22408938	2.957809041	-4.91984817	1.18E-06	4.116867041
SPIDR	0.227220091	3.418733956	4.918592189	1.19E-06	4.110976211
LRRC18	-0.216290283	0.339199176	-4.917216312	1.19E-06	4.104524613
DDC	0.607369072	0.961100567	4.91617736	1.20E-06	4.099653966
AC068580.6	-0.247376642	1.348354538	-4.914916603	1.21E-06	4.09374474
IGHD2-21	-0.306392121	0.398759713	-4.914836338	1.21E-06	4.093368582
GOLPH3	0.229344415	6.009390863	4.914177374	1.21E-06	4.090280584
MACF1	-0.294404442	2.867090831	-4.913760709	1.22E-06	4.088328224
TFG	0.219879168	4.665192228	4.912461249	1.22E-06	4.082240338
SEMA4G	0.407276574	1.767684569	4.911914333	1.23E-06	4.079678508
MBP	-0.211120767	1.876731962	-4.911857303	1.23E-06	4.079411384
HDDC3	0.206003497	2.131205086	4.911672116	1.23E-06	4.078544011
TDRD7	-0.203834391	2.65344061	-4.908626488	1.25E-06	4.064283237
CNIH3	-0.212435156	0.754499875	-4.908590119	1.25E-06	4.064112994
IDH2	0.316344104	5.682191853	4.90814669	1.25E-06	4.062037379
SLC52A2	0.297859553	4.824303641	4.907651867	1.25E-06	4.059721391
EMCN	-0.322487556	1.583308215	-4.907158609	1.25E-06	4.057412946

PLA1A	-0.460087094	1.907245394	-4.904931063	1.27E-06	4.046990653
MRGBP	0.237318405	3.307995557	4.903243553	1.28E-06	4.039097948
CSNK1E	0.248534923	4.279316978	4.899741549	1.30E-06	4.022726489
SAA2-SAA4	-0.393880001	0.674058705	-4.897965602	1.31E-06	4.0144282
DPH2	0.211639587	3.3852522	4.896195626	1.32E-06	4.006160527
IGLV1-41	-0.707862379	1.74322234	-4.895945223	1.33E-06	4.004991097
KAT2A	0.311463711	4.201019658	4.894656265	1.33E-06	3.998972278
UQCRH	0.259634781	6.128846382	4.894025133	1.34E-06	3.996025718
GBP6	-0.344353478	0.638325266	-4.892725003	1.35E-06	3.989956899
FTLP14	0.369564989	1.948692273	4.892682698	1.35E-06	3.989759451
PIEZO1	-0.315881019	3.47646051	-4.892343604	1.35E-06	3.98817686
ETV4	0.489888884	3.599076497	4.892101037	1.35E-06	3.987044835
SLC18A2	-0.213896637	0.538058129	-4.88972286	1.37E-06	3.97594892
PRPF6	0.22021807	5.619079959	4.888513928	1.37E-06	3.97031026
F7	0.237276997	0.28107308	4.888433841	1.37E-06	3.969936763
RUNX1	-0.251254504	3.312597788	-4.88667074	1.39E-06	3.961715756
SF3B4	0.235542224	5.611384495	4.884944861	1.40E-06	3.953670915
CPLX1	0.3537288	1.431477522	4.88438608	1.40E-06	3.951066822
AC004967.7	0.275497825	2.933856122	4.882624112	1.41E-06	3.942857273
VNN1	-0.483721652	1.252182606	-4.882037593	1.42E-06	3.940125096
SLC4A7	-0.237341979	1.747051044	-4.881858434	1.42E-06	3.939290584
TCEB1	0.260860888	3.59264578	4.881092777	1.42E-06	3.9357245
RP11-211C9.1	0.261509913	0.573377414	4.880358251	1.43E-06	3.932303888
PMEPA1	-0.441810759	4.307246711	-4.88010864	1.43E-06	3.931141579
ISYNA1	0.398373039	3.737468147	4.879423182	1.44E-06	3.927950042
PAXIP1-AS1	0.272887899	2.27483934	4.879215073	1.44E-06	3.926981152
PLEKHH3	0.258967187	2.847567571	4.878579359	1.44E-06	3.924021706
RP11-325K4.3	-0.233228456	1.130137688	-4.877249633	1.45E-06	3.917832551
PSMB6	0.219795789	5.865850041	4.87516766	1.47E-06	3.908145167
DENND3	-0.241975779	1.946300052	-4.874986076	1.47E-06	3.907300434
MFF	0.206358565	3.406136517	4.873860738	1.47E-06	3.902066001
RAD21	0.263683984	5.243222832	4.872876457	1.48E-06	3.89748858
NPC1	-0.259299805	3.03227923	-4.872786146	1.48E-06	3.897068628

BCHE	-0.280561838	0.752085978	-4.872736091	1.48E-06	3.896835874
AC007283.4	-0.204601223	0.639403024	-4.8703443	1.50E-06	3.885716594
RPL8	0.319281279	9.503655859	4.869887819	1.50E-06	3.883595008
RP5-908M14.9	0.314993582	1.533948682	4.867192155	1.52E-06	3.871070056
FBXO6	-0.309362905	3.781587901	-4.867175092	1.52E-06	3.870990794
SLC7A1	0.307945628	2.858679501	4.866054937	1.53E-06	3.865788049
P4HA2	-0.272343888	3.329472302	-4.864693516	1.54E-06	3.859466175
COX8A	0.231293036	7.755527475	4.864422413	1.54E-06	3.858207475
SCML2	0.205815031	0.490538413	4.863856001	1.55E-06	3.8555779
AURKA	0.43959921	2.928729066	4.862226161	1.56E-06	3.848012891
NDUFB11	0.23845448	5.888146181	4.861124353	1.57E-06	3.842900085
NDUFS6	0.287100026	5.37127308	4.860986406	1.57E-06	3.842260035
ATP7B	0.332744105	1.225784782	4.859590129	1.58E-06	3.83578246
RP11-111M22.4	0.23738091	0.848286305	4.858919272	1.58E-06	3.832670838
NME4	0.30811649	4.601711611	4.857629775	1.59E-06	3.826690887
CTD-2135D7.5	-0.205074341	0.444984231	-4.852210853	1.64E-06	3.8015768
ADAMTS7P3	-0.209783383	0.291679914	-4.850796338	1.65E-06	3.795025405
RPAIN	0.211601424	2.268304344	4.849422566	1.66E-06	3.78866437
KIAA1324	0.725051629	2.767505973	4.848177837	1.67E-06	3.782902264
ATP6V0D1	-0.203839529	3.950345248	-4.845352651	1.69E-06	3.769828896
U8	0.509414756	0.884409057	4.845123421	1.69E-06	3.768768451
CPS1	1.057552153	1.578529534	4.844851652	1.70E-06	3.767511277
EDNRB	-0.367602269	1.801642561	-4.84070231	1.73E-06	3.748324827
WDR72	0.469453605	0.902512762	4.840454402	1.73E-06	3.747178982
SOX4	0.346438039	5.296039865	4.840367833	1.73E-06	3.746778866
RPL7	0.285936526	7.476415334	4.840233439	1.73E-06	3.746157719
NR2F1	-0.377242017	2.870103131	-4.839857538	1.74E-06	3.744420455
RNASEH1-AS1	0.261628028	2.254296206	4.839181761	1.74E-06	3.741297593
MCU	0.281304927	3.704980316	4.838695532	1.75E-06	3.739050905
CFAP57	-0.291936871	0.69953032	-4.83624752	1.77E-06	3.727742642
RECQL	-0.293166285	2.819919409	-4.835433549	1.77E-06	3.723983767
HSPB8	-0.442028576	3.099839449	-4.835396901	1.77E-06	3.723814543
TNFRSF11B	-0.387621577	1.461609766	-4.834697249	1.78E-06	3.720584069

AC026271.5	0.218468089	1.740509437	4.834029613	1.79E-06	3.717501815
RP11-390F4.6	0.231099209	0.44482881	4.833664435	1.79E-06	3.715816073
SHFM1	0.238712945	3.110946017	4.832818556	1.80E-06	3.711911761
KCNN4	-0.628014206	3.616647349	-4.830060358	1.82E-06	3.699185093
SIGLEC12	-0.301903137	0.779682863	-4.829124154	1.83E-06	3.694866837
CDC25A	0.260622524	1.13797279	4.828024555	1.84E-06	3.689795888
SAA4	-0.264380214	0.460335454	-4.826835755	1.85E-06	3.684314763
FAM162B	-0.307531433	1.483295973	-4.82628445	1.85E-06	3.681773309
B4GALT2	0.232346177	4.13347436	4.82466806	1.87E-06	3.674323466
BATF	-0.410354457	3.684453703	-4.824080331	1.87E-06	3.671615219
OBSL1	0.431949788	2.982984404	4.824028958	1.87E-06	3.671378512
SLC35A2	0.260666743	4.290840726	4.823894687	1.88E-06	3.670759838
GAS2	0.296591038	0.625459888	4.823769326	1.88E-06	3.670182238
CPLX2	0.632646341	0.579934064	4.823642071	1.88E-06	3.669595923
TMED3	0.250866523	3.015760238	4.8221754	1.89E-06	3.662839398
LYRM4	0.201983696	1.877139018	4.821054098	1.90E-06	3.657675151
ESPL1	0.321357513	1.311566173	4.820476707	1.91E-06	3.655016354
PHACTR1	-0.253432287	1.11312883	-4.820208867	1.91E-06	3.653783093
GLI3	-0.309678999	1.155398425	-4.818253883	1.93E-06	3.644783301
B4GALT3	0.211650302	4.250036629	4.816358158	1.94E-06	3.636059491
RP11-21L23.4	0.330946524	0.461337108	4.81467396	1.96E-06	3.628311711
FOXRED1	0.210883778	2.488501327	4.810335604	2.00E-06	3.608365431
PANX2	0.485485123	2.300013825	4.809852162	2.01E-06	3.606143746
AC093673.5	-0.32469463	3.228896551	-4.809268044	2.01E-06	3.603459667
NCOA4	-0.220057635	5.639452365	-4.808219895	2.02E-06	3.598644065
PSRC1	0.291514916	1.8014024	4.80600624	2.04E-06	3.588476821
MSH2	0.260157	3.24556974	4.805526203	2.05E-06	3.586272589
AC009005.2	0.347481813	1.792563297	4.805267202	2.05E-06	3.585083393
E2F1	0.409497544	3.119871405	4.804308127	2.06E-06	3.580680337
SUGCT	-0.313726995	1.178579514	-4.804214526	2.06E-06	3.580250662
CCNJ	0.206138885	1.984425937	4.803132261	2.07E-06	3.575283096
RP11-672A2.1	0.270804275	0.286704937	4.798123667	2.12E-06	3.552307064
NOL6	0.220521773	3.761481597	4.797857109	2.12E-06	3.551084892

HLA-G	-0.503533783	2.248194856	-4.797768678	2.12E-06	3.550679444
RP11-44K6.4	-0.205945723	0.378077337	-4.797546348	2.13E-06	3.549660121
SUV420H2	0.24345473	1.721091829	4.796760334	2.14E-06	3.546056801
PLS3	-0.386767142	5.419694536	-4.796258747	2.14E-06	3.543757659
OSBPL3	-0.289465209	2.579904054	-4.795774599	2.15E-06	3.54153866
STIP1	0.221709629	5.351104377	4.791440378	2.19E-06	3.521682696
MAPRE2	-0.245992936	3.103680938	-4.786471438	2.24E-06	3.498939084
PRRT3-AS1	0.311034361	1.506797903	4.786297613	2.24E-06	3.498143849
TRMT6	0.223821911	2.859413652	4.784354266	2.27E-06	3.489254993
RP11-626G11.4	0.243835759	0.6499477	4.781714739	2.29E-06	3.477187084
IGKV1-13	-0.44608437	0.874320191	-4.779799458	2.31E-06	3.468434229
PLEKHA4	-0.340683685	2.643601553	-4.778130968	2.33E-06	3.46081182
FAM107A	-0.388934173	1.33328037	-4.776932923	2.35E-06	3.455340109
RAB20	-0.287242328	4.530442472	-4.775677483	2.36E-06	3.449607603
CH17-360D5.2	-0.289626783	0.589663284	-4.774843901	2.37E-06	3.44580212
RP11-799B12.1	0.383932701	2.336814246	4.773664913	2.38E-06	3.440420816
CCDC89	-0.209627462	0.558466661	-4.773490365	2.39E-06	3.439624225
GATA6	-0.381678787	1.93541991	-4.772216438	2.40E-06	3.433811139
GUCY2EP	0.253911788	0.26107337	4.771852294	2.40E-06	3.432149767
LRCH2	-0.266944613	0.853661272	-4.771722551	2.41E-06	3.431557853
CHD7	0.264676343	1.604144857	4.770869419	2.42E-06	3.427666072
PSME2	-0.265027943	5.391310137	-4.770867483	2.42E-06	3.427657238
NUDT5	0.211565352	3.817008176	4.770831667	2.42E-06	3.42749387
ZDHHC16	0.223817068	4.309152358	4.770489324	2.42E-06	3.425932373
SH3BP5	-0.284904538	1.888527183	-4.770462701	2.42E-06	3.425810945
ARHGEF19	0.37178614	3.364512515	4.770422595	2.42E-06	3.42562802
LRRC45	0.269162596	3.075112348	4.765814531	2.47E-06	3.4046199
ESAM	-0.386192214	3.831687093	-4.765702743	2.48E-06	3.404110486
LRRK2	-0.603447793	2.324168882	-4.764018577	2.49E-06	3.396437175
SHMT2	0.296958344	4.485352055	4.76334212	2.50E-06	3.393355836
LINC01273	0.202899546	0.868700101	4.762681031	2.51E-06	3.390344883
DLX6	0.213993001	0.285726997	4.761846258	2.52E-06	3.386543422
TIMM13	0.23872423	4.456124085	4.76173749	2.52E-06	3.386048149

DDRGK1	0.252298718	5.054128357	4.760711948	2.53E-06	3.381378883
TGIF1	0.239008197	3.483387799	4.760444954	2.54E-06	3.380163414
IFIT1	-0.519449059	2.783799068	-4.759773858	2.55E-06	3.377108582
DHX58	-0.294083855	2.7784833	-4.759591369	2.55E-06	3.376277962
TNS1	-0.392900851	3.859201011	-4.758388667	2.56E-06	3.370804436
GPT	0.316721724	0.932418875	4.7581241	2.57E-06	3.36960055
C9orf172	0.211273834	1.095629071	4.757108341	2.58E-06	3.364979024
RP11-394O4.5	-0.32463107	1.465256766	-4.754574008	2.61E-06	3.353452172
CTSH	-0.561523346	5.977533441	-4.751735395	2.64E-06	3.340548029
RP11-16K12.1	-0.227586897	0.465650225	-4.751291753	2.65E-06	3.338531899
RRP1	0.205626282	3.179937342	4.750565448	2.66E-06	3.335231576
ZNF444	0.239225213	2.661865102	4.750532069	2.66E-06	3.335079911
SLC25A39	0.237637921	5.690098555	4.750029088	2.67E-06	3.332794658
CSRP1	-0.207978878	4.628563906	-4.749707863	2.67E-06	3.331335313
FKBP2	0.288799978	5.580531399	4.748733923	2.68E-06	3.326911191
CHURC1	-0.215645752	3.152771864	-4.748672105	2.68E-06	3.326630413
POLR3H	0.309218509	3.018535843	4.747944671	2.69E-06	3.323326621
SLC25A1	0.23764655	5.237376369	4.74626621	2.71E-06	3.315705319
HSPBP1	0.243950049	4.237167196	4.746005478	2.72E-06	3.314521644
MFSD10	0.285929898	4.977913587	4.74399987	2.74E-06	3.305418565
FCGR3B	-0.267463267	0.709720043	-4.74061162	2.79E-06	3.290047908
RP11-264I13.2	-0.213672312	1.876240723	-4.740525342	2.79E-06	3.289656641
COQ3	0.210235758	2.248555227	4.738415348	2.82E-06	3.280089966
CDT1	0.386209937	2.398145982	4.737538849	2.83E-06	3.276117081
SLIRP	0.216364031	3.407274263	4.737535772	2.83E-06	3.276103134
CYFIP2	-0.33720031	2.35576665	-4.736971702	2.84E-06	3.273546745
KLF9	-0.311088858	3.354476466	-4.734762464	2.87E-06	3.263537055
WEE1	0.254944621	2.444239021	4.73463388	2.87E-06	3.262954594
GS1-124K5.4	0.281089286	1.660777247	4.733515283	2.88E-06	3.25788817
TENM3	-0.228468406	0.582983612	-4.732857959	2.89E-06	3.254911485
FRY	-0.22573805	1.356749638	-4.732223546	2.90E-06	3.252038909
FBXO32	-0.401330029	2.972626811	-4.731150375	2.91E-06	3.247180472
RANGRF	0.276043038	3.072229489	4.731113001	2.91E-06	3.247011292

TTC7A	-0.214090192	3.386502381	-4.730205723	2.93E-06	3.242904694
RNASE1	-0.617669666	7.919319035	-4.730008549	2.93E-06	3.242012328
MEDAG	-0.408822702	1.806460657	-4.727789112	2.96E-06	3.231969946
ADAM28	-0.383364107	2.019788048	-4.727527577	2.96E-06	3.230786849
RAPGEFL1	0.335885616	1.657025166	4.726544837	2.98E-06	3.226341806
GLIS2	-0.295925369	3.102951443	-4.726113091	2.98E-06	3.224389236
MRPS33	0.220421169	2.907318936	4.723003921	3.03E-06	3.210332839
ATP5G2	0.218446821	5.788890352	4.722795727	3.03E-06	3.209391908
CHMP1B	0.255889368	4.804535745	4.722098548	3.04E-06	3.206241285
RP11-395B7.4	-0.285575792	0.721797507	-4.721644234	3.05E-06	3.204188424
AC083900.1	0.200866899	0.542345961	4.721025224	3.06E-06	3.201391656
TIPARP	-0.426130308	3.586885162	-4.720904123	3.06E-06	3.200844547
DPP3	0.215648907	4.152860918	4.720363313	3.07E-06	3.198401429
HYLS1	0.20994118	1.686402746	4.719990054	3.07E-06	3.196715376
ARG2	0.365772085	1.71140022	4.719635247	3.08E-06	3.195112782
VDAC2	0.21582699	4.409668023	4.719359675	3.08E-06	3.193868156
MERTK	-0.276293534	2.362625984	-4.718905013	3.09E-06	3.191814808
TRIB2	-0.364000065	3.37985088	-4.71776339	3.10E-06	3.186659805
LL21NC02-1C16.2	-0.462808146	1.305615919	-4.717308608	3.11E-06	3.184606554
DUSP1	-0.530417549	6.833497278	-4.717005406	3.12E-06	3.183237757
CBX3	0.227387118	5.500033779	4.716612965	3.12E-06	3.181466213
PLGRKT	-0.282255663	3.715850803	-4.715297271	3.14E-06	3.175527928
KNSTRN	0.282658594	2.323386631	4.714748232	3.15E-06	3.17305033
LINC01224	0.254046539	0.477108541	4.713033551	3.17E-06	3.165314348
HSPA9	0.211578483	5.681413814	4.711157142	3.20E-06	3.156851657
KIAA0930	-0.212659772	3.339018119	-4.710374088	3.21E-06	3.153320958
HNRNPA1P16	0.202427955	1.164469334	4.709034645	3.23E-06	3.14728281
CMTM4	0.292904515	3.168824906	4.708832307	3.24E-06	3.146370817
MRPL23	0.227941625	3.238959068	4.708550204	3.24E-06	3.145099362
SLC31A1	-0.219137205	3.865606295	-4.707020923	3.26E-06	3.138208
LDOC1	-0.551193019	4.41026897	-4.705380312	3.29E-06	3.130817234
RP4-755D9.1	-0.347284968	0.827298399	-4.70526752	3.29E-06	3.130309205
CBLN3	-0.229830274	1.506744874	-4.703981576	3.31E-06	3.124517947

ATAD3B	0.293457351	2.14848683	4.702634871	3.33E-06	3.118454604
ATPIF1	0.232788111	5.047633975	4.70238173	3.34E-06	3.11731505
KLF5	0.371957492	4.788618936	4.702280399	3.34E-06	3.11685891
GPX3	-0.471899935	5.585648429	-4.700921443	3.36E-06	3.110742437
IGHD6-19	-0.323627375	0.449409878	-4.700657609	3.36E-06	3.109555143
PAH	0.332924933	0.374795052	4.700151817	3.37E-06	3.107279176
RP11-876N24.5	-0.223529611	1.40510854	-4.699916898	3.38E-06	3.106222161
COL4A1	-0.409270166	5.495224059	-4.699290044	3.39E-06	3.103401876
MANBA	-0.223270897	2.801589157	-4.698531099	3.40E-06	3.099987758
SELM	-0.371428684	4.078442719	-4.69838132	3.40E-06	3.099314034
WHSC1	0.245014304	2.40774513	4.6979253	3.41E-06	3.097262932
RAB7B	-0.472187095	2.574597703	-4.697392643	3.42E-06	3.094867355
NPW	0.557820018	1.094346717	4.69738325	3.42E-06	3.09482511
C1orf95	0.418956297	0.657071588	4.696690126	3.43E-06	3.091708226
SLC1A1	-0.432876958	2.172802485	-4.694679714	3.46E-06	3.08267006
NR0B1	0.569776302	0.622838046	4.69448146	3.46E-06	3.081778964
CAPN5	0.47306461	2.912778801	4.693883243	3.47E-06	3.079090359
SMARCA1	0.350655835	4.155481623	4.693085469	3.49E-06	3.075505361
NRIP1	-0.303896932	2.861034675	-4.691883269	3.50E-06	3.070104025
NOC4L	0.213220836	3.504783952	4.691341067	3.51E-06	3.067668395
NUDT18	-0.237005286	2.511244427	-4.690310615	3.53E-06	3.063040197
FBP1	-0.429245797	5.69904626	-4.690296256	3.53E-06	3.062975714
RP11-579D7.4	0.223034534	0.619354449	4.688509049	3.56E-06	3.054950836
CHMP4C	0.307008339	3.848407925	4.687309686	3.58E-06	3.049567058
ADCY7	-0.291315855	1.768310663	-4.686756511	3.59E-06	3.047084353
HRSP12	0.24871261	3.486556226	4.685696249	3.61E-06	3.042326541
TMED6	0.442227675	1.191389943	4.684716812	3.62E-06	3.037932298
TP53INP1	-0.333773652	3.7698625	-4.683605873	3.64E-06	3.032949089
UCKL1	0.243167628	3.652669108	4.68216146	3.67E-06	3.026471668
C8orf33	0.236260072	4.134330397	4.681175717	3.68E-06	3.022052189
MAZ	0.218823643	3.91633781	4.681109032	3.69E-06	3.021753246
NEK7	-0.226022548	3.828802216	-4.677765243	3.74E-06	3.006768247
COX17	0.232595457	3.690474734	4.677147574	3.75E-06	3.004001272

TGFBR1	-0.250048759	3.336529738	-4.67470531	3.80E-06	2.993063926
GFPT1	0.255142144	4.41356321	4.673984091	3.81E-06	2.989835045
NECAB2	0.226572699	0.328481689	4.672853289	3.83E-06	2.984773389
NEK8	0.21674713	1.798892587	4.672472766	3.84E-06	2.983070354
U47924.32	0.223688017	0.762594395	4.672222443	3.84E-06	2.981950103
PHPT1	0.300050607	5.439869387	4.671757354	3.85E-06	2.97986887
RNF19B	-0.227331995	4.546518582	-4.670074637	3.88E-06	2.972340445
FZD1	-0.346770494	2.95716932	-4.670057703	3.88E-06	2.972264695
ZNF239	0.252194718	1.748946401	4.66904696	3.90E-06	2.967743868
SNRPGP2	0.272270637	3.381023213	4.668929222	3.90E-06	2.967217309
PEPD	-0.215477355	3.949332398	-4.6685211	3.91E-06	2.96539217
IMPDH2	0.233927412	5.589691255	4.667803045	3.92E-06	2.962181348
MCL1	-0.223053508	6.92382282	-4.66434205	3.99E-06	2.94671166
GADD45A	-0.404375119	4.057955849	-4.663376009	4.00E-06	2.942395602
SGMS2	-0.331387029	3.17897884	-4.661320986	4.04E-06	2.933216934
SUSD1	-0.226221848	2.62961077	-4.661239007	4.04E-06	2.932850855
CRB3	0.29894862	3.994084633	4.660633296	4.06E-06	2.930146228
NR3C1	-0.245718014	3.34379802	-4.660601556	4.06E-06	2.930004509
IGLV3-29	-0.384621507	0.7416901	-4.658887514	4.09E-06	2.922352736
SRPK1	0.229794129	3.821164334	4.658415522	4.10E-06	2.920246139
FAM117B	0.200629897	2.174543394	4.656175707	4.14E-06	2.910252037
GOLGA2	0.224132648	4.005836994	4.655893527	4.15E-06	2.908993255
CKLF	-0.289109238	3.458387529	-4.654432507	4.17E-06	2.902476889
GPX7	-0.372269287	2.943842892	-4.653977402	4.18E-06	2.900447437
NSUN2	0.251484587	4.25844847	4.651769888	4.23E-06	2.890606035
MYOF	-0.381413955	4.802251734	-4.651518807	4.23E-06	2.889486953
MX1	-0.483759183	3.699226242	-4.64775257	4.31E-06	2.872707252
DENND1C	-0.233716128	2.878884842	-4.647219482	4.32E-06	2.870333197
AOX1	-0.357094039	1.182838097	-4.646739215	4.33E-06	2.868194586
ENTPD6	0.246356365	4.275301521	4.646325845	4.33E-06	2.866354024
NAA38	0.284718023	4.150855649	4.643802669	4.38E-06	2.85512266
C17orf89	0.224678113	3.606072623	4.643302208	4.40E-06	2.852895632
REPIN1	0.224598259	4.55914852	4.639312411	4.48E-06	2.835149094

STEAP2	-0.407345533	2.143823561	-4.638673181	4.49E-06	2.83230711
FCGRT	-0.297826693	5.338356906	-4.637528927	4.51E-06	2.827220714
TSNAX	0.200285088	3.14307268	4.637325235	4.52E-06	2.826315389
GAS5	0.393664686	5.586175424	4.635744055	4.55E-06	2.819288963
COA7	0.201624529	2.736362503	4.633128701	4.61E-06	2.807671708
CTD-2132N18.4	0.211830403	1.031273171	4.632835964	4.61E-06	2.806371763
CTD-2396E7.11	0.343431215	4.358960732	4.631912075	4.63E-06	2.802269574
ITGA9	-0.364611391	2.226545327	-4.631882163	4.63E-06	2.802136776
MAGED1	0.261262883	5.477848326	4.630892532	4.66E-06	2.797743544
PAQR8	-0.307574829	2.013532903	-4.63088732	4.66E-06	2.797720405
CYP26B1	-0.260604254	0.990545058	-4.624662655	4.79E-06	2.770107246
EXOSC5	0.266197961	3.836163251	4.624572884	4.79E-06	2.769709265
EMB	-0.444385649	4.008865426	-4.624520882	4.79E-06	2.769478727
PIGR	-1.097469205	5.83366572	-4.624351499	4.80E-06	2.768727827
MCM4	0.373400514	3.649719719	4.624113671	4.80E-06	2.76767354
IRF6	0.292102843	3.891812001	4.624034499	4.81E-06	2.767322582
PDIA3P1	0.245101618	2.495410717	4.623683832	4.81E-06	2.765768201
SLC46A3	-0.324677322	2.729475569	-4.621828563	4.85E-06	2.75754625
SORBS3	-0.267117667	3.58088633	-4.619366975	4.91E-06	2.746641966
DCBLD2	-0.469534228	3.15695646	-4.617092427	4.96E-06	2.736570967
HLA-S	-0.327630007	1.014509121	-4.616270299	4.98E-06	2.732931953
EVA1A	-0.444078912	3.136434724	-4.615955478	4.99E-06	2.731538611
TPM1	-0.313348756	3.674183023	-4.615889042	4.99E-06	2.731244586
SRMS	0.329178079	0.962192623	4.614995207	5.01E-06	2.72728915
IGIP	-0.227302204	2.011760898	-4.608453812	5.16E-06	2.698363294
CCM2L	-0.201675042	0.994081029	-4.607259892	5.19E-06	2.693087878
DMBT1	-0.883594429	3.0013187	-4.607246123	5.19E-06	2.693027046
PFKFB3	-0.381821532	4.493912056	-4.606562615	5.21E-06	2.690007495
XDH	-0.444093492	1.73120737	-4.605598185	5.23E-06	2.685747605
AC010518.2	-0.31373228	0.79606976	-4.604921318	5.25E-06	2.68275837
PPP1R12B	-0.286382301	1.913395545	-4.604380099	5.26E-06	2.680368484
RAMP2	-0.334770635	3.447296505	-4.603375211	5.29E-06	2.675931839
RFX5	-0.213084443	3.936657762	-4.6030541	5.29E-06	2.674514303

ASAP1	-0.267273133	2.773779642	-4.602948441	5.30E-06	2.674047893
RBP4	-0.522534374	1.708966363	-4.602280836	5.31E-06	2.671101115
STMN3	-0.480125596	3.462161679	-4.602191717	5.32E-06	2.67070778
FABP5	-0.411450138	2.693676646	-4.602112542	5.32E-06	2.670358338
RANBP6	-0.207308457	2.66625662	-4.602038486	5.32E-06	2.670031491
LINC00676	0.801754899	0.849018014	4.601331894	5.34E-06	2.666913207
OIP5	0.311459265	1.504626477	4.600763711	5.35E-06	2.664406058
MIR222HG	-0.238728223	0.890359638	-4.598616762	5.40E-06	2.654935055
IL1RL1	-0.334833837	0.691966314	-4.59740056	5.43E-06	2.649571729
HSD17B10	0.207165563	5.770727117	4.59717366	5.44E-06	2.648571266
RUNX2	-0.265880577	1.645886108	-4.596475402	5.46E-06	2.645492752
TMEM200B	-0.284772954	1.447062923	-4.5964745	5.46E-06	2.645488772
TMEM59L	0.7496812	1.934990316	4.596174185	5.46E-06	2.64416486
AMN	0.415654939	1.391106474	4.595320778	5.49E-06	2.640403124
MEST	0.323038715	3.912611193	4.594640863	5.50E-06	2.637406582
HHIP	-0.382990866	0.818691219	-4.593824332	5.52E-06	2.633808482
AGAP2-AS1	-0.359576292	2.309301623	-4.593494926	5.53E-06	2.632357095
PDCD2L	0.249794351	2.760630901	4.592429506	5.56E-06	2.627663437
NOP16	0.217605803	2.833851489	4.5922712	5.56E-06	2.626966114
FAM189B	0.217963256	3.288413222	4.592059926	5.57E-06	2.626035505
MRPS21	0.265331347	5.311295108	4.590752269	5.60E-06	2.620276479
RP11-783K16.5	0.32528469	1.614131615	4.585289198	5.74E-06	2.596232962
CAPN10	0.215494919	1.974354849	4.584516806	5.77E-06	2.592835709
TXN	0.329037509	7.308583836	4.582243712	5.83E-06	2.582840895
ELF3	0.368351601	5.382968551	4.581562288	5.84E-06	2.579845553
TMEM198	0.256049329	1.227788925	4.5811693	5.85E-06	2.578118281
ADAMTS14	-0.273807411	1.179618137	-4.580578918	5.87E-06	2.57552367
SPOCD1	-0.295382865	1.01681868	-4.580260953	5.88E-06	2.574126404
RAB15	0.421839159	3.587600438	4.578768834	5.92E-06	2.567570623
ULK1	0.247004734	3.313732126	4.577917666	5.94E-06	2.563831809
CHD1L	0.234219138	4.305806622	4.57765613	5.95E-06	2.562683125
SPP1	-0.821786412	6.922765232	-4.577193299	5.96E-06	2.560650481
AC145343.1	-0.286943836	1.172580833	-4.577045932	5.97E-06	2.560003322

SARAF	-0.212323889	6.339309835	-4.576397397	5.98E-06	2.55715551
PI4K2B	0.221521348	3.392502204	4.575202348	6.02E-06	2.551908856
MYL12A	-0.212694244	6.593385436	-4.574918213	6.02E-06	2.550661593
COA6	0.2843047	4.270492986	4.573726982	6.06E-06	2.545433254
KCNMB2-AS1	0.383625643	0.854190091	4.572799224	6.08E-06	2.541362166
TMEM130	-0.501631365	1.620875145	-4.572745823	6.08E-06	2.54112786
SCAND1	0.282024224	4.39977181	4.572375096	6.10E-06	2.539501306
GJB2	-0.636997207	3.207395012	-4.572112077	6.10E-06	2.538347392
FGFR1	-0.36412021	2.346378663	-4.571094626	6.13E-06	2.533884213
ECHDC1	-0.246849827	3.465983751	-4.570927356	6.14E-06	2.533150548
MAP6	-0.254125204	1.006322013	-4.567714905	6.23E-06	2.519065193
VAMP7	-0.203286437	4.251644632	-4.565304671	6.30E-06	2.508503224
IGFBP2	0.722394507	4.368868978	4.564736122	6.31E-06	2.506012511
SAPCD2	0.376754552	2.217474227	4.560975233	6.42E-06	2.489543912
FSCN1	-0.517501259	4.522881928	-4.559867425	6.45E-06	2.4846953
CYP4F3	0.498943898	0.730268337	4.559772884	6.46E-06	2.484281566
C14orf159	-0.248201	2.56820596	-4.55949835	6.47E-06	2.483080188
C7orf55	0.250021711	2.363662836	4.559125941	6.48E-06	2.481450606
ZWINT	0.389663344	3.63721335	4.55847302	6.50E-06	2.478593862
EIF5B	0.245335882	4.361738417	4.55834416	6.50E-06	2.478030101
SSH3	0.242418154	4.088735499	4.556493597	6.55E-06	2.46993553
SYTL4	-0.244687125	1.478628337	-4.554146939	6.63E-06	2.45967533
SLC34A2	-0.794641227	7.905265435	-4.549750068	6.76E-06	2.440464147
RNF5	0.208647097	5.351330389	4.549052656	6.78E-06	2.437418523
CTD-2517O10.6	0.215643445	1.306976399	4.549019287	6.78E-06	2.437272811
SAMD10	0.26226982	2.600999057	4.548871875	6.79E-06	2.436629116
LRP11	0.271344083	4.171627659	4.54854376	6.80E-06	2.435196431
SURF6	0.200781489	3.183176687	4.547987235	6.81E-06	2.432766622
CHST2	-0.391452491	2.235885571	-4.547755921	6.82E-06	2.431756778
TPST1	-0.263211784	3.019550618	-4.546980862	6.85E-06	2.428373461
ATP13A4-AS1	-0.287183741	0.467788285	-4.546871587	6.85E-06	2.42789649
CACHD1	-0.420705247	1.869398397	-4.546416303	6.86E-06	2.42590936
ATF4	0.205407613	6.666023953	4.544692076	6.92E-06	2.418385455

ATP5EP2	0.204762121	1.101229959	4.543898634	6.94E-06	2.414924044
RPP25L	0.22424297	3.988753912	4.543773837	6.95E-06	2.414379662
RPL37A	0.232443426	6.672635231	4.542638967	6.98E-06	2.409429857
MCM3	0.263670309	4.783040204	4.542189268	7.00E-06	2.40746878
RP11-728F11.4	0.229683619	0.461319038	4.540259123	7.06E-06	2.399053705
BTNL10	0.230505516	0.62624604	4.537043778	7.16E-06	2.385042706
C20orf24	0.27720055	4.454497	4.535038653	7.23E-06	2.376309919
ABHD11	0.316369228	4.440292909	4.53319367	7.29E-06	2.368277723
CHST3	-0.401001679	2.092618777	-4.531359883	7.35E-06	2.360297251
LZTS1	-0.246303458	1.4796639	-4.529602892	7.41E-06	2.352653772
BPIFA2	0.585998854	1.12887163	4.527902652	7.47E-06	2.345259779
ATP6V0E1	-0.217235971	6.818252943	-4.527544323	7.48E-06	2.343701808
NEK6	-0.225481363	4.246737579	-4.526715354	7.51E-06	2.34009798
VPS13C	-0.232003097	2.438328424	-4.52586438	7.54E-06	2.336399126
GDF10	-0.344058336	0.926153521	-4.525716211	7.54E-06	2.335755158
MT1L	-0.489842993	2.505279205	-4.523650715	7.61E-06	2.326780172
TUSC8	0.285720489	0.34503332	4.519714065	7.75E-06	2.309685102
CCDC34	0.277385146	2.047536218	4.519078399	7.77E-06	2.306925979
KLF4	-0.442253184	2.968487964	-4.518906404	7.78E-06	2.306179494
YBEY	0.245943367	2.626955521	4.517114749	7.84E-06	2.298404975
POLR2K	0.227114675	4.9029677	4.517100587	7.84E-06	2.298343533
B3GALNT1	-0.322912906	1.973593794	-4.511847054	8.03E-06	2.275563336
FASTKD5	0.200124876	3.039875417	4.511359672	8.05E-06	2.273451205
RPL6P27	0.275667112	2.820751157	4.509612766	8.11E-06	2.265882492
AC093375.1	0.219079667	0.62061934	4.508340095	8.16E-06	2.260370168
PDCD6	0.225529943	3.798276409	4.508056197	8.17E-06	2.259140718
IQCG	-0.214409097	1.423663977	-4.507316224	8.20E-06	2.255936514
COL4A2	-0.400624406	5.504900784	-4.507173228	8.20E-06	2.255317375
NFKB2	-0.252087366	3.968379095	-4.506692927	8.22E-06	2.253237915
LINC00858	0.231376273	0.420549649	4.506648735	8.22E-06	2.253046593
RP11-284F21.10	0.560870922	1.633168999	4.505169605	8.28E-06	2.24664405
SURF2	0.213155339	3.820903484	4.505130279	8.28E-06	2.246473848
RP11-616M22.5	-0.323988666	0.718079209	-4.502881237	8.36E-06	2.236742463

BATF3	-0.282460102	1.339008398	-4.500329103	8.46E-06	2.225705046
PFN2	0.534518052	4.390804291	4.495865647	8.63E-06	2.206415448
NDRG2	-0.349193776	2.465694833	-4.495588827	8.64E-06	2.205219702
CA8	0.339770949	0.840764983	4.495179344	8.66E-06	2.203451034
TBL1X	0.283632539	2.82084344	4.494861273	8.67E-06	2.202077302
SERPINB9P1	-0.302589489	1.319903479	-4.490857381	8.83E-06	2.18479236
JADE1	0.265562311	2.435655305	4.490093961	8.86E-06	2.181498263
IGHD4-4	-0.29032332	0.366285006	-4.489922759	8.87E-06	2.180759611
C8orf76	0.227780693	2.299901382	4.488572806	8.92E-06	2.174936136
CHST15	-0.322050707	3.599960951	-4.487744124	8.95E-06	2.171362138
AC017060.1	-0.569845343	2.049531343	-4.486848969	8.99E-06	2.167502133
FAM43A	-0.28382185	1.946677145	-4.486506613	9.00E-06	2.166026043
RIMBP2	0.240232377	0.289620443	4.485422204	9.05E-06	2.161351235
CBX4	0.222474357	4.004380546	4.484938457	9.07E-06	2.159266174
POP7	0.25410474	4.765989866	4.483829184	9.11E-06	2.154485733
PLVAP	-0.337294981	5.10508333	-4.483778019	9.12E-06	2.154265262
FOXA3	0.513560565	1.719094502	4.48364704	9.12E-06	2.153700881
RPRD1A	0.227014466	2.940138362	4.483264752	9.14E-06	2.152053715
JPH1	0.359108943	1.712065599	4.482427635	9.17E-06	2.148447278
YBX2	0.369029552	0.914912386	4.482122141	9.18E-06	2.147131315
ABCF1	0.205563753	4.56064693	4.47757719	9.37E-06	2.127562989
TDG	0.218261102	2.579636486	4.477193575	9.39E-06	2.125912169
CDCA3	0.29716176	1.47421723	4.477151016	9.39E-06	2.125729031
HSF4	0.41838642	2.0912332	4.476769688	9.41E-06	2.124088196
ATP5G1	0.248439481	4.32543205	4.474727435	9.49E-06	2.115302685
TUBB4B	0.254165271	7.174245093	4.473874515	9.53E-06	2.111634626
HSDL1	0.212906317	3.014834702	4.472937922	9.57E-06	2.107607464
TAF1D	0.209507272	3.145328588	4.472549741	9.59E-06	2.105938592
CLDN3	0.584834633	5.763109226	4.47101477	9.65E-06	2.099340738
LAMP3	-0.562240375	4.216788272	-4.470821739	9.66E-06	2.098511172
IGHD6-25	-0.471242553	0.998580189	-4.470646832	9.67E-06	2.097759518
PPP1R26	0.235966133	2.904360663	4.468836392	9.75E-06	2.089980879
ECT2	0.360519443	3.196356922	4.466681166	9.84E-06	2.080724636

NEIL3	0.343611527	1.124996307	4.466317553	9.86E-06	2.0791634
CNTROB	0.221418206	2.947180859	4.466209214	9.87E-06	2.078698251
PFDN4	0.251459655	3.436214357	4.465547583	9.89E-06	2.075857789
RP11-140K17.3	0.239750067	1.957061738	4.465314713	9.91E-06	2.074858145
BARX1-AS1	0.229873212	0.299291188	4.463872628	9.97E-06	2.068668741
GNAS	0.224095931	6.356210604	4.463543848	9.98E-06	2.067257882
NKAIN1	0.222396665	0.305648194	4.463091772	1.00E-05	2.065318092
TMEM205	0.228091947	5.33788252	4.462981144	1.00E-05	2.064843428
IGLV5-52	-0.252227916	0.573795573	-4.461632295	1.01E-05	2.059056928
CHEK2	0.260884696	2.111765128	4.461559411	1.01E-05	2.058744306
CDH24	0.322781098	2.071675997	4.461045574	1.01E-05	2.056540427
CARD14	0.312446113	0.963770307	4.460028986	1.01E-05	2.052180911
RP11-417L19.6	0.212582406	1.259202874	4.459479505	1.02E-05	2.049824906
KB-1208A12.3	0.220607136	1.556861417	4.459171116	1.02E-05	2.048502746
HPN	0.54145358	3.804632584	4.457869224	1.02E-05	2.042922063
CLK2	0.236542486	3.851478815	4.457356016	1.03E-05	2.040722565
RALGDS	-0.212476979	3.488336824	-4.457140839	1.03E-05	2.039800431
SNORA42	0.209577271	0.457782926	4.4557666	1.03E-05	2.033912144
SOX18	-0.277216191	2.071958745	-4.455163975	1.04E-05	2.031330571
RFC3	0.264206428	2.499800501	4.45494634	1.04E-05	2.030398326
RP11-21L23.3	0.244059452	0.787027306	4.454548603	1.04E-05	2.028694721
PALMD	-0.231817625	1.191818167	-4.451900526	1.05E-05	2.017355933
MIR3135B	-0.201950431	0.296433007	-4.451733288	1.05E-05	2.016640047
DZIP1L	-0.22553111	0.939879995	-4.448970822	1.07E-05	2.004818503
NIPSNAP3A	-0.212270726	3.081548735	-4.448962767	1.07E-05	2.00478404
CDR2	-0.224591796	3.296794503	-4.447894296	1.07E-05	2.00021351
PHB2	0.224034131	6.096570443	4.447709785	1.07E-05	1.999424344
SPINT1	0.292008586	6.250914431	4.447608913	1.07E-05	1.998992916
SNHG19	0.397067768	4.000321386	4.447381087	1.07E-05	1.998018551
LINC00467	0.221365514	2.016409478	4.446616647	1.08E-05	1.994749522
SLC9A7	-0.242613075	1.933455559	-4.44500256	1.08E-05	1.987848798
AC093323.3	0.222515819	3.111652803	4.444876901	1.09E-05	1.987311663
GMNN	0.279651262	2.998308038	4.44387508	1.09E-05	1.98302984

FCHSD1	-0.214982254	2.041016581	-4.442697122	1.10E-05	1.977996341
ACSL3	0.234585804	3.963937879	4.442256689	1.10E-05	1.976114657
CCDC120	0.231944582	2.500060846	4.440734781	1.11E-05	1.969613853
FOXO3	-0.233491206	2.891841153	-4.440424927	1.11E-05	1.968290569
TMEM14A	0.267100972	4.776274162	4.438830876	1.12E-05	1.961484253
KLF11	-0.236032688	2.672487906	-4.437246148	1.12E-05	1.954719986
KIAA0101	0.346046952	2.325694237	4.436097465	1.13E-05	1.949818329
RP11-400N13.3	-0.218753445	0.530660594	-4.434292635	1.14E-05	1.942119133
KIF22	0.234054711	3.638012543	4.432281981	1.15E-05	1.933545322
RP11-291L22.6	0.240581833	1.375487056	4.431935111	1.15E-05	1.932066565
DCLRE1A	0.201574876	2.210486162	4.430038227	1.16E-05	1.923981767
S100A9	-0.787425649	7.596394429	-4.428848125	1.17E-05	1.91891101
BHLHA15	0.439155178	1.873588575	4.428761359	1.17E-05	1.918541369
HSD17B7	0.203217671	2.093073894	4.427391391	1.17E-05	1.912705908
SLC22A23	0.365327469	2.581162934	4.42416971	1.19E-05	1.898989541
BCL9	0.262710287	3.153741528	4.421895597	1.20E-05	1.889313018
RP11-156K13.1	0.338344848	0.911899842	4.42170333	1.20E-05	1.888495119
CCDC28A	-0.216112324	4.005002778	-4.421560037	1.20E-05	1.887885571
ACACA	0.217241027	3.001195313	4.421316747	1.21E-05	1.8868507
TRAF2	0.253347437	3.236856526	4.421190002	1.21E-05	1.886311592
SLC25A21	0.206932595	0.397680706	4.420670495	1.21E-05	1.88410202
C17orf53	0.28852304	1.566870351	4.420643172	1.21E-05	1.883985814
MRPL47	0.225523019	4.519964108	4.420332801	1.21E-05	1.882665866
MRPL57	0.21104884	4.181874267	4.420228237	1.21E-05	1.882221194
MRPS25	0.277562603	3.424253197	4.419300169	1.22E-05	1.878274892
BRI3BP	0.240049721	2.489630886	4.419025631	1.22E-05	1.877107656
PAIP1	0.217514862	4.355908454	4.41838594	1.22E-05	1.874388181
CELSR2	0.242735889	1.51040471	4.418053241	1.22E-05	1.872973944
IGHD	-0.944535989	4.046720888	-4.417246662	1.23E-05	1.869545748
MVD	0.249117725	3.143911925	4.416853633	1.23E-05	1.867875471
CRNDE	0.379957533	2.909952388	4.416516476	1.23E-05	1.866442748
FSCN2	0.225984206	0.963812076	4.416373097	1.23E-05	1.865833497
NPTXR	-0.33784308	1.3462618	-4.415606	1.24E-05	1.862574246

ATP5B	0.215647163	7.659261055	4.41471267	1.24E-05	1.858779309
ACADVL	0.24359486	5.574554116	4.414539297	1.24E-05	1.858042892
HS6ST1	0.28018502	3.233007632	4.414478408	1.24E-05	1.857784266
RP11-672L10.6	0.225938298	1.068335541	4.414259201	1.24E-05	1.856853213
LINC00493	0.215030272	4.506538955	4.413162114	1.25E-05	1.852194116
FAAH2	0.283078022	2.953845774	4.412931596	1.25E-05	1.851215292
EPHA3	-0.297927764	0.859603769	-4.411161653	1.26E-05	1.84370134
GJA1	-0.481108251	4.77396588	-4.410285597	1.27E-05	1.839983243
DUSP5	-0.407959283	3.947555332	-4.408913065	1.27E-05	1.83415941
ABR	-0.23499111	3.444617107	-4.408812371	1.27E-05	1.833732221
AFAP1	-0.226181865	2.544285585	-4.408421232	1.28E-05	1.832072908
CEP170B	0.273244626	3.633520754	4.408333572	1.28E-05	1.831701051
TFPI	-0.490881527	3.573730315	-4.408097272	1.28E-05	1.830698689
TEP1	-0.211580978	2.423496691	-4.407437062	1.28E-05	1.827898408
HHIP-AS1	-0.413447651	1.269530591	-4.407201595	1.28E-05	1.82689977
RP11-1038A11.3	0.266806338	0.515955181	4.406589204	1.29E-05	1.824302785
STEAP3	-0.359731032	4.062406329	-4.402901684	1.31E-05	1.808672084
FAM195A	0.266805822	2.788237058	4.397000837	1.34E-05	1.783684676
C9orf173	0.205920051	0.511727355	4.39609052	1.35E-05	1.77983266
FCN3	-0.474228185	1.937855721	-4.394098552	1.36E-05	1.771406192
PPP1R9B	-0.211094736	4.411088162	-4.393883146	1.36E-05	1.770495189
CFI	-0.418662793	4.128822851	-4.393729162	1.36E-05	1.769843976
ADAMTS4	-0.332649665	1.477107514	-4.39317862	1.37E-05	1.767515865
TWSG1	-0.262373312	3.626354952	-4.392513794	1.37E-05	1.764704837
SIDT1	-0.263878297	1.319841037	-4.391921893	1.37E-05	1.76220248
MGAT3	-0.330396252	1.086064589	-4.39158878	1.38E-05	1.760794327
RHPN1	0.394327126	3.082354634	4.389949591	1.39E-05	1.753866506
TMEM176A	-0.445877667	4.170295592	-4.389076652	1.39E-05	1.750178119
PLA2G2A	-0.537291812	1.348501443	-4.388237926	1.40E-05	1.746634928
SORD	0.249188957	2.25650425	4.387952756	1.40E-05	1.745430372
ARHGEF10L	-0.242798063	2.648490365	-4.385929058	1.41E-05	1.736884375
RP11-420A23.1	0.204040258	0.787673114	4.38464167	1.42E-05	1.731449684
WDR43	0.207031413	3.697402742	4.383891219	1.42E-05	1.728282347

STOML2	0.230185702	5.469117337	4.382069845	1.44E-05	1.720597178
G6PD	0.497823261	5.058098416	4.380214819	1.45E-05	1.712773057
AC019117.2	-0.599806566	2.430285008	-4.379359201	1.45E-05	1.709165268
CTD-2527I21.15	0.272955986	0.741546792	4.37888441	1.46E-05	1.707163551
NME1	0.300670554	4.140202271	4.378878447	1.46E-05	1.70713841
HSD17B14	-0.404087303	3.1794143	-4.377426474	1.46E-05	1.701018151
CNN2	-0.248067208	5.91642872	-4.376595452	1.47E-05	1.697516132
CALCB	0.227580819	0.215974765	4.375964962	1.47E-05	1.694859585
AC079922.2	0.267623858	2.130693059	4.375806518	1.48E-05	1.694192039
RAC3	0.425658178	3.079706133	4.373802511	1.49E-05	1.685750867
RASA1	-0.230817701	3.115471208	-4.373505191	1.49E-05	1.684498816
ADAMTS15	-0.230772613	0.80584651	-4.371930981	1.50E-05	1.677870936
RP11-443P15.2	0.477396195	0.935462984	4.370005683	1.51E-05	1.669767878
GRK5	-0.270787453	1.891163994	-4.369865876	1.51E-05	1.669179594
POGZ	0.223079104	3.355513865	4.36915668	1.52E-05	1.666195703
PLAT	-0.627685802	3.539685106	-4.368825332	1.52E-05	1.664801732
RGS4	-0.265535149	0.906300941	-4.367249705	1.53E-05	1.65817446
FCHO1	-0.262698638	1.675781532	-4.367177787	1.53E-05	1.657872019
RAB17	0.289827793	2.927370479	4.367033007	1.53E-05	1.657263177
C5orf22	0.205468901	3.25491178	4.366355315	1.54E-05	1.654413535
HUNK	0.266277398	0.934954705	4.364093442	1.55E-05	1.644905509
DDX11	0.26641314	1.973562877	4.361781703	1.57E-05	1.635192576
CDH15	0.281883242	0.845834943	4.359824966	1.58E-05	1.626974934
PNN	0.268160362	4.497425967	4.356846937	1.60E-05	1.614474762
FAM64A	0.334777655	1.545732619	4.351269846	1.64E-05	1.591086395
FLJ22447	-0.352789278	0.538722856	-4.350497685	1.65E-05	1.58785041
SIGMAR1	0.208447117	4.785768885	4.350359225	1.65E-05	1.587270207
AC019117.1	-0.378773635	0.858018804	-4.350153232	1.65E-05	1.586407044
EPS8L2	0.296820623	4.424678759	4.349754436	1.65E-05	1.584736095
C15orf48	-0.656580193	4.619107702	-4.348440588	1.66E-05	1.579232093
RCN3	-0.451711235	4.068291918	-4.342637564	1.71E-05	1.55494036
HBB	-0.711633271	3.948470574	-4.341706458	1.71E-05	1.551045509
MESP1	0.303629753	1.348561247	4.341640199	1.71E-05	1.550768371

CDC42EP1	-0.423104923	5.387636254	-4.340205999	1.73E-05	1.544770656
PRKAA2	0.273145338	1.159984847	4.338978973	1.73E-05	1.53964078
FGA	1.134596256	2.833417141	4.337337298	1.75E-05	1.532779471
ERH	0.207593524	6.057779643	4.337225515	1.75E-05	1.532312365
AL450992.2	0.412638209	3.154515947	4.33586228	1.76E-05	1.526616749
ZNF793-AS1	0.249101388	0.964273466	4.335560092	1.76E-05	1.525354423
IGHV3-64	-0.643917188	2.237236671	-4.335105491	1.76E-05	1.523455585
C8G	0.271945219	0.80167471	4.334435607	1.77E-05	1.520657861
ADA	-0.27243424	1.9775355	-4.334254562	1.77E-05	1.519901808
CTD-2124B8.2	0.240614799	1.89201573	4.331695543	1.79E-05	1.509218343
LSM2	0.243722375	5.16561224	4.331102178	1.80E-05	1.506741979
FECH	0.239457239	2.8737891	4.330299583	1.80E-05	1.503392913
CXCL16	-0.298455808	5.56359561	-4.329877156	1.81E-05	1.501630441
RP11-126L15.4	0.2205154	1.066786893	4.329055058	1.81E-05	1.498200901
CBL	-0.201311547	2.086067599	-4.328064806	1.82E-05	1.494070674
DNMT3B	0.268758934	1.185458261	4.328017099	1.82E-05	1.493871716
KRT8	0.340876791	7.642619326	4.327943529	1.82E-05	1.493564903
SERPINA1	-0.686135903	6.784842496	-4.327460878	1.82E-05	1.491552192
CYBA	-0.352353404	5.398905478	-4.32681392	1.83E-05	1.488854626
GAREM	0.24942553	1.972962285	4.326637549	1.83E-05	1.488119293
GPR162	-0.261042799	1.156880419	-4.326514699	1.83E-05	1.487607115
SKA1	0.30654224	1.432224078	4.326138526	1.84E-05	1.486038892
RP11-101E13.5	0.214534798	3.869970268	4.325351488	1.84E-05	1.482758226
ALOX12P2	0.208705071	0.602713416	4.324979578	1.84E-05	1.481208159
C20orf85	-0.735662492	1.84963831	-4.324810118	1.85E-05	1.48050192
COMTD1	0.332549642	3.031386027	4.324196033	1.85E-05	1.477942864
HOOK2	0.242286139	2.923045382	4.32217004	1.87E-05	1.469502418
CATSPER1	-0.282324857	0.878478628	-4.321855719	1.87E-05	1.468193259
AC079630.2	-0.342677067	0.685056677	-4.321445517	1.87E-05	1.466484886
FNIP2	-0.323017381	2.76369739	-4.320994899	1.88E-05	1.464608367
SLC19A2	0.254098368	3.149648895	4.320571258	1.88E-05	1.462844351
UFSP1	0.21365986	1.460894881	4.320413031	1.88E-05	1.462185545
MAFG-AS1	0.255428759	2.128606082	4.318826998	1.89E-05	1.455583054

ANXA2	-0.307264814	6.538442989	-4.318097163	1.90E-05	1.452545584
ARL4C	-0.337032156	4.480488959	-4.317674075	1.90E-05	1.450784968
CHAF1B	0.233911875	1.801276442	4.315169405	1.93E-05	1.440365444
FHL2	-0.424869261	3.128321062	-4.314918754	1.93E-05	1.439323039
BCL10	-0.225172295	3.495203359	-4.313675485	1.94E-05	1.434153352
LIMK1	-0.277905055	3.822174676	-4.313119606	1.94E-05	1.431842379
PLXNB1	0.310107001	3.116558449	4.312571912	1.95E-05	1.429565702
MMP15	0.368633632	4.368328256	4.311857803	1.95E-05	1.426597666
DUSP7	-0.231169221	2.606243413	-4.310948644	1.96E-05	1.422819611
PLA2G4A	0.608595177	3.551952837	4.310758567	1.96E-05	1.422029832
AKR1C4	0.337930945	0.377675678	4.310353418	1.97E-05	1.420346519
C4A	-0.3474835	1.578153072	-4.309905593	1.97E-05	1.41848607
IL1A	-0.285806192	0.596255216	-4.309598464	1.97E-05	1.417210233
ERBB3	0.344522294	4.841498958	4.309277363	1.98E-05	1.415876448
TRIM24	0.228851263	2.723610495	4.308315807	1.98E-05	1.411882892
IGSF10	-0.214425192	0.556787255	-4.307901932	1.99E-05	1.410164236
PPAPDC1B	0.268629187	3.77628357	4.307778707	1.99E-05	1.409652561
IGHD3-10	-0.385001729	0.715019803	-4.306169237	2.00E-05	1.402970701
NOTCH4	-0.224924188	1.709256761	-4.304549791	2.02E-05	1.396249766
RAP2A	-0.213415437	3.163597019	-4.302577452	2.03E-05	1.388067437
AB019441.29	0.246436044	2.265871694	4.302217943	2.04E-05	1.386576375
RP11-660L16.2	0.293458917	2.390825059	4.302157936	2.04E-05	1.386327509
ROMO1	0.283542714	6.252546148	4.301385252	2.04E-05	1.383123237
ZNF428	0.228341517	3.674073637	4.298695927	2.07E-05	1.371974941
INPP4B	-0.272567456	1.511820674	-4.29811291	2.07E-05	1.369558963
AC005336.4	0.362705471	0.583799648	4.297717435	2.08E-05	1.367920318
PCNA	0.282573621	5.840554002	4.297505138	2.08E-05	1.367040729
TNFRSF12A	-0.435882511	5.287012329	-4.294064376	2.11E-05	1.35279054
MIR221	-0.278660788	0.87195932	-4.292946447	2.12E-05	1.348162826
RSPH4A	-0.370106527	0.9359168	-4.292804556	2.12E-05	1.347575543
PDRG1	0.201841753	3.531828899	4.292582123	2.12E-05	1.346654935
RP11-663N22.1	0.227046962	0.414779969	4.290944602	2.14E-05	1.339878906
RP11-132F7.2	0.331800126	1.192163642	4.290797724	2.14E-05	1.339271243

TTC32	0.228565993	1.970051888	4.290623409	2.14E-05	1.338550096
NRN1	-0.363500649	1.809803329	-4.290535103	2.14E-05	1.338184782
PER3	-0.309521426	1.940771515	-4.290157448	2.15E-05	1.33662253
RNU6-529P	-0.21269444	1.062459604	-4.289161999	2.16E-05	1.332505257
RNU7-75P	0.27054897	0.725906072	4.288575089	2.16E-05	1.330078157
SNRPG	0.250952753	4.607895762	4.288520658	2.16E-05	1.329853077
UBE2S	0.355368226	2.792550016	4.287529143	2.17E-05	1.325753529
RP11-977G19.5	0.279485387	3.035031805	4.286950442	2.18E-05	1.323361217
DNAJB9	-0.255488545	4.358434636	-4.283616106	2.21E-05	1.309583145
KIAA0907	0.258909961	3.385313109	4.28283241	2.22E-05	1.306346218
PCYT2	0.210947135	2.767573702	4.282673669	2.22E-05	1.305690629
VDAC3	0.25242707	4.824741061	4.28199045	2.22E-05	1.302869253
GRAMD4	0.29466044	3.87799671	4.281164805	2.23E-05	1.299460278
SH3RF2	-0.35168317	1.245973783	-4.278993075	2.25E-05	1.290496423
ANXA3	-0.396238027	3.251097211	-4.278335728	2.26E-05	1.287784042
AGER	-0.736797848	3.747498668	-4.276039129	2.28E-05	1.278310733
RBL2	-0.217663623	3.597512991	-4.275891602	2.28E-05	1.277702358
WDR18	0.219964384	3.688406919	4.273634471	2.31E-05	1.268396756
FAM24B	0.208437938	1.262572685	4.27335637	2.31E-05	1.26725053
TMEM243	-0.291381234	3.213201368	-4.272992772	2.31E-05	1.265752019
FKBP4	0.299552185	4.442977735	4.271561541	2.33E-05	1.259854588
GPR4	-0.209820216	1.613432939	-4.271160249	2.33E-05	1.258201384
CBFA2T2	0.205906168	2.300410239	4.270896979	2.33E-05	1.257116866
H2AFX	0.286926736	4.725373882	4.270281931	2.34E-05	1.254583472
FAM166B	-0.363064122	1.02943398	-4.270155875	2.34E-05	1.254064285
ETHE1	-0.281212464	3.924101301	-4.269140115	2.35E-05	1.24988121
MARCKSL1	0.338290063	6.795213779	4.268997112	2.35E-05	1.249292374
PRRC2A	0.225829705	5.150461871	4.267980526	2.36E-05	1.245106951
TOMM20	0.203573809	6.124440069	4.264253183	2.40E-05	1.229768897
ANXA2R	-0.231628076	1.349432545	-4.263374146	2.41E-05	1.226153465
FANCA	0.206957386	1.368194867	4.263349206	2.41E-05	1.226050901
LRRC26	0.270544542	0.44062595	4.263156531	2.41E-05	1.225258534
TNIK	-0.335219853	1.781329899	-4.263063118	2.41E-05	1.224874389

HIPK1	-0.206944834	3.281122797	-4.263034541	2.41E-05	1.224756876
PCK1	0.245922245	0.208382327	4.263010179	2.41E-05	1.224656691
MMP28	-0.617729108	2.480343267	-4.262631883	2.42E-05	1.223101119
SRGAP3-AS2	-0.413647712	0.743815153	-4.26231796	2.42E-05	1.221810347
GAL3ST1	0.298418901	1.208771899	4.262224449	2.42E-05	1.221425869
TEKT1	-0.368632407	0.700664114	-4.262209215	2.42E-05	1.221363236
MRPL13	0.240917478	3.219873194	4.26002452	2.45E-05	1.212382987
CTA-398F10.2	0.208300007	0.603906947	4.258529489	2.46E-05	1.206240087
XPOT	0.245559502	3.894035054	4.258476233	2.46E-05	1.206021299
CROT	-0.201201502	1.961364168	-4.258172346	2.47E-05	1.204772925
SNED1	-0.25151053	1.744139925	-4.257222167	2.48E-05	1.200870098
GSTO2	0.248577612	1.194654251	4.257075133	2.48E-05	1.200266235
STRA13	0.287182996	4.708225129	4.256645257	2.48E-05	1.198500858
PRKAB1	0.236603865	3.575901783	4.255624078	2.49E-05	1.194307837
MIR22HG	-0.244306543	2.806769786	-4.252744468	2.52E-05	1.182489017
ADAMTS9	-0.226461025	1.18459157	-4.249143323	2.56E-05	1.16771926
CH507-42P11.8	0.420967603	2.035654249	4.24867263	2.57E-05	1.165789619
FAM84A	0.378768049	1.485441158	4.247615206	2.58E-05	1.16145535
TRIM47	-0.298170781	3.922782128	-4.24706326	2.59E-05	1.15919338
CRLF1	0.921985555	3.330101306	4.246737074	2.59E-05	1.157856739
NINJ1	-0.236469018	4.612844605	-4.245987584	2.60E-05	1.15478586
HIF1A	-0.281059939	5.41699783	-4.245575938	2.60E-05	1.153099439
RP11-48B3.4	0.216007249	1.019181712	4.245394978	2.60E-05	1.152358132
PDCD11	0.2002287	3.091377176	4.244342535	2.62E-05	1.148047371
PRRG2	0.228631653	2.820707446	4.243793634	2.62E-05	1.145799492
SPON2	-0.39042597	2.811656235	-4.240681078	2.66E-05	1.133057949
PSMD11	0.202130894	4.191428435	4.239666892	2.67E-05	1.12890816
PRC1	0.337871928	2.625283667	4.237678065	2.69E-05	1.120773073
DPP4	-0.638334741	4.039357705	-4.237272227	2.70E-05	1.119113471
KIAA0895L	0.28385899	2.230754675	4.236745719	2.70E-05	1.116960632
HCAR1	0.400649925	1.284888537	4.236687587	2.70E-05	1.116722949
SNX9	-0.205837075	3.963162957	-4.235307898	2.72E-05	1.11108279
HSD17B6	-0.533093584	2.666180255	-4.233367796	2.74E-05	1.103154551

CEBPA	-0.427572947	2.959695789	-4.232557815	2.75E-05	1.099845562
CCDC146	-0.283142304	1.272840449	-4.231681341	2.76E-05	1.096265591
SEPHS2	0.252236529	5.375548401	4.227709444	2.81E-05	1.080050971
SNHG1	0.297568676	3.36177299	4.226244317	2.83E-05	1.074073404
UCHL1	0.799951155	3.402531241	4.225565027	2.84E-05	1.071302625
CACNB3	0.268788509	2.578233237	4.222800962	2.87E-05	1.060032465
TMED2	0.216079642	6.654627567	4.222750524	2.87E-05	1.059826876
FOXA1	0.428442269	4.105534492	4.221473307	2.89E-05	1.054621541
RHBDL1	0.351676863	1.715243009	4.220261234	2.90E-05	1.049683064
RP11-494O16.3	-0.293859093	1.054509745	-4.219780535	2.91E-05	1.047724864
BUB1B	0.330871021	1.953472976	4.218887234	2.92E-05	1.044086424
FAR2	-0.247847644	1.23784519	-4.217258383	2.94E-05	1.037453909
RASL11A	-0.376538806	2.564070362	-4.212509096	3.00E-05	1.018128905
POLR3GL	-0.209575546	3.99942438	-4.211957444	3.01E-05	1.015885529
GNRH2	0.214558864	0.264343716	4.21099595	3.02E-05	1.011976125
SMPD1	-0.217913588	3.980828791	-4.210757617	3.02E-05	1.011007199
VMO1	-0.379807384	2.480331044	-4.209404261	3.04E-05	1.005506189
TUBA4B	-0.329937732	0.675864119	-4.208487123	3.05E-05	1.001779221
NUDT19	0.222322986	3.304962282	4.207231057	3.07E-05	0.996676178
SNHG7	0.277499964	2.883601994	4.205630069	3.09E-05	0.990173867
RP11-130L8.2	0.260257733	0.865963893	4.205256364	3.09E-05	0.988656423
ELMO3	0.303022863	4.109916574	4.204825714	3.10E-05	0.986907904
RP11-504P24.8	0.294947296	1.847177468	4.203696617	3.11E-05	0.982324351
PROC	0.377605251	1.034804147	4.202814344	3.13E-05	0.978743575
MTA1	0.2220639	3.099240975	4.201991606	3.14E-05	0.975405057
CD47	-0.281463128	4.873362912	-4.201136799	3.15E-05	0.971937056
ARHGAP23	-0.27068021	2.474938801	-4.20061391	3.16E-05	0.969815989
SRCIN1	0.254824774	1.150040643	4.200220458	3.16E-05	0.968220136
BSPRY	0.287854812	3.87293543	4.199698719	3.17E-05	0.966104163
FAM168B	0.204523186	4.390550047	4.197679829	3.20E-05	0.957918626
RP11-51F16.1	-0.283324152	1.338200779	-4.197137916	3.20E-05	0.955722075
AUNIP	0.225988651	1.02919656	4.194338207	3.24E-05	0.94437816
CEP72	0.211937583	1.683449563	4.193182968	3.26E-05	0.939699392

CTD-2134A5.3	0.258358935	1.507503104	4.192748672	3.26E-05	0.937940787
CDIP1	-0.254259461	2.942827489	-4.19122659	3.28E-05	0.931778719
NPM3	0.264793781	4.580040112	4.191002068	3.29E-05	0.93086993
RP5-1119A7.14	0.265109609	0.52210172	4.189749577	3.31E-05	0.925801104
RP11-329L6.2	0.281496105	1.512120912	4.189696891	3.31E-05	0.925587917
RP11-476D10.1	-0.298384143	0.552629471	-4.188896222	3.32E-05	0.922348391
CTSV	0.357084722	1.215158739	4.187926574	3.33E-05	0.91842594
ZMYND8	0.205710771	3.301295461	4.186800883	3.35E-05	0.913873323
PLA2G10	0.516470594	1.811125391	4.18517182	3.37E-05	0.907286948
APOO	0.212575758	3.453927053	4.183875555	3.39E-05	0.902047794
NHS	-0.270014014	1.266581634	-4.183394611	3.40E-05	0.900104332
C20orf96	0.269207901	2.870851593	4.180499597	3.44E-05	0.88841018
RP11-164P12.5	0.262624236	2.718182421	4.179576249	3.45E-05	0.884681984
PPP2R1B	0.229995745	2.755911537	4.179464866	3.45E-05	0.884232306
LMTK2	0.219220994	2.885309362	4.179130762	3.46E-05	0.882883519
FAM83F	0.243583974	0.560923596	4.179112545	3.46E-05	0.882809979
RP11-649A18.5	0.243210916	1.152543412	4.178310331	3.47E-05	0.879571842
CTD-2256P15.2	0.249136569	1.500791462	4.177921287	3.48E-05	0.878001679
DROSHA	0.217283673	3.266622817	4.177045313	3.49E-05	0.874466776
AMIGO2	-0.468999187	3.824687081	-4.175709518	3.51E-05	0.869077649
NUDT14	0.274091119	3.817871059	4.175065443	3.52E-05	0.866479769
AC124789.1	-0.223426318	0.484573449	-4.173556039	3.54E-05	0.860393042
RP11-717A5.2	-0.247805937	0.979709683	-4.171546143	3.57E-05	0.852291246
KIFC2	0.352804012	2.263626303	4.1714993	3.57E-05	0.852102468
BMP3	-0.517753898	1.956188328	-4.169405997	3.60E-05	0.843668418
CUL7	0.21676707	3.760052057	4.169224408	3.61E-05	0.842936973
ERICH5	0.410431656	1.603728576	4.167966824	3.63E-05	0.83787219
CIT	0.519566537	2.866961343	4.167391739	3.63E-05	0.835556574
ARID5B	-0.217411744	3.037868661	-4.16540992	3.67E-05	0.827578933
PTPLAD1	0.209367657	4.189080883	4.165320365	3.67E-05	0.827218523
VAT1	-0.24361151	5.795313283	-4.162688119	3.71E-05	0.816628327
FAM177A1	0.269176988	3.427568132	4.161848291	3.72E-05	0.8132508
SERPIND1	-0.598996652	1.466603314	-4.161645263	3.72E-05	0.812434378

MGLL	-0.374165912	4.17194856	-4.160066145	3.75E-05	0.806085664
RP11-498C9.12	0.205865066	0.626530018	4.159958972	3.75E-05	0.805654866
DLGAP1-AS1	0.287334075	2.335008622	4.159806204	3.75E-05	0.805040808
LIPG	-0.20682006	0.777574047	-4.159159625	3.76E-05	0.80244209
C10orf107	-0.325458192	0.923007559	-4.159029829	3.77E-05	0.801920463
TTC29	-0.208265018	0.336376339	-4.15694579	3.80E-05	0.79354714
NSUN7	0.20807764	1.745363295	4.156602869	3.80E-05	0.792169718
MAP1A	-0.273447392	1.419222016	-4.15599524	3.81E-05	0.789729291
DACT2	0.456264495	1.184046209	4.154493431	3.84E-05	0.783698984
TMBIM1	-0.238008655	5.539970642	-4.154050449	3.85E-05	0.78192064
BRAT1	0.214516542	3.327414632	4.1527998	3.87E-05	0.776900885
LRRC75A-AS1	0.327711148	5.931110201	4.152665735	3.87E-05	0.776362868
TBC1D8	0.305063468	2.736860359	4.1522669	3.87E-05	0.774762399
EMC6	0.201802074	2.092604238	4.151742981	3.88E-05	0.772660208
CAMK2N1	-0.465217299	3.451548545	-4.151306156	3.89E-05	0.770907663
CGREF1	0.367470904	1.155233581	4.150940294	3.90E-05	0.769439951
SNHG3	0.307799281	2.932403279	4.149201913	3.93E-05	0.76246783
POLE	0.206926336	1.803226198	4.14901365	3.93E-05	0.761712929
CALCOCO1	-0.219901675	3.193866847	-4.147960545	3.95E-05	0.757490748
MASTL	0.211805864	2.397635807	4.146552916	3.97E-05	0.751848743
SH3YL1	0.212767905	2.667982098	4.144624345	4.00E-05	0.744121624
ASNS	0.309148035	3.135949945	4.144269402	4.01E-05	0.742699853
MT1E	-0.524386749	4.526026552	-4.143474955	4.02E-05	0.739518008
HCG4P5	-0.550461485	2.896677718	-4.142982428	4.03E-05	0.737545668
URB1	0.227198075	2.569943204	4.141740764	4.05E-05	0.732574363
UCK2	0.341675743	2.211330741	4.137312933	4.13E-05	0.714857772
TEAD3	0.217031264	4.014939974	4.137017153	4.13E-05	0.713674928
LOXL2	-0.424482654	3.048696127	-4.136791012	4.14E-05	0.71277063
TSPYL4	-0.202327465	2.647684869	-4.134632945	4.17E-05	0.704143223
C11orf88	-0.272828373	0.487149883	-4.134537757	4.18E-05	0.703762783
AKAP14	-0.26513561	0.476426909	-4.132480995	4.21E-05	0.695544453
PAQR6	0.275162139	1.117065682	4.131182672	4.23E-05	0.690358629
CDC45	0.341323097	2.041049103	4.129576088	4.26E-05	0.68394364

GS1-358P8.4	-0.222353976	2.198177221	-4.124812459	4.35E-05	0.664936465
FDPS	0.209050526	4.852573277	4.124562936	4.35E-05	0.663941414
PPP1R3B	0.31774203	3.582295004	4.124439822	4.36E-05	0.663450482
MROH1	0.233533504	2.747285082	4.124239987	4.36E-05	0.662653645
RAB30-AS1	0.205156371	1.850060883	4.123989316	4.36E-05	0.661654147
FANCI	0.266626926	2.206467915	4.122900802	4.38E-05	0.657314592
CCT6A	0.247739903	5.780862983	4.121884114	4.40E-05	0.65326235
FZD2	-0.31333912	2.213087835	-4.120428149	4.43E-05	0.647460895
ZMAT4	0.254145573	0.273712971	4.118627043	4.46E-05	0.640286835
EHD3	-0.211600787	2.021272906	-4.116540093	4.50E-05	0.631977879
SLC12A4	-0.203682743	2.762313953	-4.115999376	4.51E-05	0.629825718
ARNTL2	-0.405357309	2.139395512	-4.114849862	4.54E-05	0.625251299
CYS1	-0.366080785	1.306967216	-4.114646573	4.54E-05	0.624442448
RASSF3	-0.224539653	3.945013476	-4.114639756	4.54E-05	0.624415325
FOXP4	0.269601459	4.191883836	4.111616216	4.60E-05	0.612389611
PCGF2	0.231855908	3.807053575	4.111379068	4.60E-05	0.611446736
HCG4	-0.216824276	0.709545184	-4.110140046	4.63E-05	0.606521345
ACTL6A	0.220192433	4.041591117	4.108458968	4.66E-05	0.599840901
AC007318.5	0.260360111	1.792089176	4.108010668	4.67E-05	0.598059833
CXCL1	-0.568041251	3.581694296	-4.107304502	4.68E-05	0.595254644
MAMSTR	0.218013888	0.91432226	4.105732966	4.71E-05	0.589013458
SPTLC3	-0.290949634	1.388089962	-4.104875071	4.73E-05	0.585607359
STEAP1	-0.500811635	3.443155768	-4.103996411	4.75E-05	0.582119512
ITGA6	-0.411097794	3.378256821	-4.103258944	4.76E-05	0.579192665
NUF2	0.356645094	2.020752191	4.102593677	4.77E-05	0.576552789
PPFIA3	0.224069497	1.331397899	4.102080695	4.78E-05	0.574517476
ZBTB7B	0.215651022	4.333019025	4.100930398	4.81E-05	0.569954404
RAP1GAP	0.500741412	3.610272552	4.099672151	4.83E-05	0.564964481
EPB41L5	0.234931198	2.608453918	4.099273326	4.84E-05	0.563383127
SLC6A14	-0.611243855	3.588043989	-4.097970219	4.87E-05	0.558217277
MYCN	0.43434803	0.808896239	4.095882376	4.91E-05	0.54994373
IGHD2-8	-0.219726652	0.322945019	-4.094809699	4.93E-05	0.545694541
AURKB	0.395098343	2.572819587	4.094677988	4.93E-05	0.545172868

ZNF579	0.202819551	2.100299964	4.092624417	4.98E-05	0.537041212
ALDH1L2	-0.257317634	1.20319915	-4.092257397	4.98E-05	0.5355883
ADORA1	-0.383898786	1.381083494	-4.09074988	5.02E-05	0.529621815
PPP2R2C	0.37493817	0.793307741	4.09017758	5.03E-05	0.527357291
RP11-466H18.1	0.300048892	4.462282292	4.090093876	5.03E-05	0.52702611
KLHDC9	0.291007429	2.049917627	4.090034493	5.03E-05	0.52679116
RP11-169F17.1	0.360102797	0.787408923	4.089938898	5.03E-05	0.526412944
ERLIN2	0.227346102	3.101765128	4.086415214	5.11E-05	0.512477426
PVT1	0.261241112	1.709058542	4.085922785	5.12E-05	0.510530857
CKS1B	0.308929205	3.579464238	4.083331708	5.17E-05	0.500291946
DLX4	0.209849163	0.924997416	4.081487226	5.21E-05	0.493006982
GUCY1B2	0.23869351	0.849672335	4.081471484	5.21E-05	0.492944824
MIR4635	-0.355593551	1.506828144	-4.08071466	5.23E-05	0.489956572
TSNARE1	0.255940933	2.230692637	4.079239497	5.26E-05	0.484133516
ENKUR	-0.270407199	0.69599778	-4.078292101	5.28E-05	0.480394806
SLC25A21-AS1	0.209624539	1.066669189	4.075067877	5.35E-05	0.467677131
PLAC4	0.228970628	0.190809615	4.074373409	5.37E-05	0.464939091
CECR2	0.265588981	0.971987202	4.072533856	5.41E-05	0.457688501
DENND4C	-0.203386168	2.579432767	-4.072429758	5.41E-05	0.457278291
FBXL6	0.241320007	2.984371056	4.072390519	5.41E-05	0.457123669
ECH1	0.220055164	5.646811297	4.071434932	5.44E-05	0.453358556
SHANK3	-0.220533143	2.007479652	-4.069793282	5.47E-05	0.446892219
BCL2L10	0.219486978	0.5273713	4.068240145	5.51E-05	0.440776776
PRKACB	-0.276440749	2.71160758	-4.067911665	5.52E-05	0.439483669
CCL28	-0.356701144	1.701214996	-4.067520968	5.53E-05	0.437945768
IFFO2	-0.26054159	2.339192818	-4.065757167	5.57E-05	0.431004632
NR2F2	-0.248408564	3.068632747	-4.063670082	5.61E-05	0.422794907
C1orf226	0.279274323	2.491561974	4.061690393	5.66E-05	0.415011277
RP11-307C12.11	0.217031851	1.409707092	4.061044584	5.68E-05	0.412472888
FMN1	-0.211346261	1.083963262	-4.059684366	5.71E-05	0.407127712
RP11-320G10.1	0.211429883	0.283059578	4.05850847	5.74E-05	0.402508204
PFKM	0.20706906	2.767597007	4.058286778	5.74E-05	0.401637427
AC098973.2	0.336531703	0.453591058	4.055758504	5.80E-05	0.391709856

TSTD1	0.305736585	5.035534042	4.055140597	5.82E-05	0.389284449
ZDHHC12	0.209095013	4.064238966	4.055074105	5.82E-05	0.389023477
RIMKLA	0.29276035	1.129469778	4.054337217	5.84E-05	0.386131542
KLHL5	-0.277919146	3.206744404	-4.052189675	5.89E-05	0.377706269
SMIM10	-0.243601365	1.817317629	-4.050055611	5.94E-05	0.369338016
PLK2	-0.3218143	3.299315583	-4.049964921	5.94E-05	0.368982487
CTPS1	0.234046665	2.856061005	4.049883555	5.94E-05	0.368663517
C22orf15	-0.217590632	0.40932145	-4.049714504	5.95E-05	0.368000822
KIFC1	0.374789748	2.858319941	4.04868781	5.97E-05	0.363976656
FAM118B	-0.206214584	2.755544458	-4.048332732	5.98E-05	0.36258514
RPL7P1	0.287888762	1.697927961	4.046211686	6.03E-05	0.354275331
CLEC3B	-0.423703971	2.682223499	-4.045714209	6.05E-05	0.352326911
MICA	-0.230941895	3.137364708	-4.045570065	6.05E-05	0.351762397
GPR87	-0.612950464	1.881044888	-4.044744202	6.07E-05	0.348528422
SORBS2	0.323818847	1.59275218	4.044243476	6.08E-05	0.346567947
PRSS23	-0.342371147	3.553696316	-4.042992565	6.12E-05	0.341671282
WDR38	-0.419972283	0.991801753	-4.039913539	6.19E-05	0.329624545
CRABP2	-0.816225266	6.143848942	-4.039124211	6.21E-05	0.326537671
CA3	-0.27545133	0.967870851	-4.038868005	6.22E-05	0.325535833
NATD1	-0.219168591	2.019179442	-4.037455642	6.26E-05	0.320014155
MIR34A	-0.245031131	1.047956635	-4.036412155	6.28E-05	0.315935771
RASAL1	0.312709069	1.487566077	4.036336416	6.29E-05	0.315639788
RP11-284F21.7	0.412462421	1.44379031	4.035105018	6.32E-05	0.310828325
ITGB6	-0.454304822	4.435498129	-4.034842083	6.32E-05	0.309801131
OGDHL	0.256779657	0.494357144	4.033179143	6.37E-05	0.303306071
RP11-1398P2.1	-0.236291873	1.582486209	-4.032936637	6.37E-05	0.302359111
POU6F2-AS2	0.288536765	0.453512462	4.031998664	6.40E-05	0.298696915
UPP1	-0.333951326	3.754950889	-4.031922786	6.40E-05	0.298400695
DYRK2	-0.209576128	2.343161036	-4.031239706	6.42E-05	0.295734249
FUT1	0.217173183	1.581664817	4.027594753	6.52E-05	0.281513087
ACYP1	0.201707621	1.87154307	4.025251263	6.58E-05	0.272376079
STOML3	-0.287016143	0.542567931	-4.024928726	6.59E-05	0.271118934
SPC24	0.285384507	1.877843639	4.022972775	6.64E-05	0.263497287

SFTPD	-0.848118414	5.840230296	-4.020416839	6.71E-05	0.253542939
PIGT	0.220467787	6.242908508	4.020164243	6.72E-05	0.252559502
SDF2L1	0.262936439	4.868748489	4.017826558	6.78E-05	0.243460881
ATP5I	0.218399474	5.985097594	4.015417492	6.85E-05	0.234089621
IGHD1-20	-0.21955851	0.267213657	-4.014181839	6.89E-05	0.229284979
TRMT2A	0.204555968	3.265710387	4.013577127	6.90E-05	0.226934157
DLGAP1-AS2	0.255351696	1.132531434	4.012874564	6.92E-05	0.224203354
GNG11	-0.37933394	3.292640635	-4.011837319	6.95E-05	0.220172491
ATRAID	0.205699548	5.912158425	4.010208247	7.00E-05	0.213843685
AC068580.5	-0.23507198	1.317399426	-4.008117946	7.06E-05	0.205726571
KDR	-0.394461248	3.194257873	-4.008108495	7.06E-05	0.20568988
SQRDL	-0.278370429	4.328878892	-4.007148193	7.09E-05	0.201962148
UST	-0.273184198	1.46644558	-4.006975401	7.09E-05	0.201291486
ANKRD1	-0.350943408	0.905943772	-4.006432159	7.11E-05	0.199183165
MCM8	0.210413346	1.647716984	4.006390423	7.11E-05	0.199021202
STRAP	0.221811852	5.618558518	4.006151141	7.12E-05	0.198092641
SLC7A2	0.641784873	2.838584006	4.006047339	7.12E-05	0.197689843
PARD6A	0.258016165	2.157226202	4.005924418	7.12E-05	0.19721287
CHCHD10	0.263453805	4.246649653	4.005690453	7.13E-05	0.196305044
PAK1IP1	0.218166534	3.687331624	4.004196329	7.17E-05	0.190508742
KCNK6	-0.258954387	2.38169497	-4.001752265	7.25E-05	0.18103162
ARHGAP29	-0.242503363	2.00463702	-4.000198619	7.29E-05	0.175010008
HEPACAM2	0.380871483	0.451507324	3.998820348	7.33E-05	0.169669949
FSTL3	-0.376659285	3.614959172	-3.995794852	7.42E-05	0.157953831
MMP13	-0.664177981	2.070357191	-3.993296712	7.50E-05	0.148286145
RFC4	0.281283766	2.733878762	3.99033944	7.59E-05	0.136848967
CDH3	-0.543124802	3.730476272	-3.987171362	7.69E-05	0.124605319
ZNF467	0.276181919	2.314525684	3.986929091	7.70E-05	0.12366939
LRRN2	-0.206014201	0.856436663	-3.986328304	7.72E-05	0.121348693
GRB7	0.295839646	3.706154666	3.985869927	7.73E-05	0.119578312
AC079630.4	-0.44133209	1.417412304	-3.984858928	7.76E-05	0.115674229
ERICH3	-0.251324386	0.412996732	-3.984816086	7.77E-05	0.115508808
CENPV	0.31522968	2.289889717	3.984441521	7.78E-05	0.114062637

CDCP1	-0.365539293	3.416973863	-3.982815506	7.83E-05	0.107786163
LINC00152	-0.242201779	2.270304497	-3.982127361	7.85E-05	0.105130624
PAK4	0.201642413	3.403161968	3.981264281	7.88E-05	0.101800621
HRCT1	-0.255077174	1.226469692	-3.981237499	7.88E-05	0.101697298
IGHV7-81	-0.336367735	1.27679915	-3.979984176	7.92E-05	0.096862866
DRAM1	-0.394894263	4.790400876	-3.979181904	7.95E-05	0.09376902
BCOR	0.204424247	2.755006498	3.978940092	7.95E-05	0.092836622
TMEM54	0.266685542	5.452758917	3.97836747	7.97E-05	0.090628869
IL17RE	0.312258565	2.43037987	3.977733078	7.99E-05	0.088183311
DNPH1	0.242637073	4.534630608	3.97748871	8.00E-05	0.087241379
HTATIP2	0.240030828	4.560845684	3.977176574	8.01E-05	0.086038309
PDZK1IP1	-0.580911679	5.131075674	-3.976529377	8.03E-05	0.083544093
RNU7-45P	-0.27577624	1.108567991	-3.974712908	8.09E-05	0.076545683
SHANK2	0.200781458	1.3883826	3.974491862	8.10E-05	0.075694252
GINS1	0.309267886	2.097967871	3.974337312	8.10E-05	0.075098976
DSCC1	0.273315796	1.827399672	3.973786974	8.12E-05	0.072979439
CTDSP2	-0.202221041	5.612354041	-3.972133281	8.18E-05	0.066612168
RPS24P8	0.208784705	1.300218759	3.971428358	8.20E-05	0.063898732
IGLV3-6	-0.21759614	0.417861566	-3.970792422	8.22E-05	0.061451234
PAIP2B	0.226948586	1.546298884	3.969449384	8.27E-05	0.056283555
RBP1	-0.323461299	2.010104596	-3.969170066	8.28E-05	0.055209015
RP11-553L6.5	-0.290416536	3.243640327	-3.968886618	8.29E-05	0.054118659
DDR1	0.250237221	5.063990991	3.968786792	8.29E-05	0.053734668
PLEKHG2	0.325977601	2.788607374	3.967173366	8.34E-05	0.047529745
PPIB	0.218910264	7.14846469	3.966062583	8.38E-05	0.043259265
DLL3	0.387462294	0.702697165	3.965932774	8.39E-05	0.042760278
PTPN3	0.208695072	2.337359771	3.964054668	8.45E-05	0.035542546
RP11-776H12.1	0.238879213	0.419854269	3.963999489	8.45E-05	0.035330537
RP11-452N17.1	0.233698127	1.074851136	3.963968647	8.45E-05	0.035212037
CH17-360D5.3	-0.278559296	0.591927479	-3.963595061	8.47E-05	0.033776725
PRRX2	-0.42375782	1.93850683	-3.961491196	8.54E-05	0.025696072
MIR5195	-0.257331344	0.832843946	-3.961178714	8.55E-05	0.024496215
HSPA1B	0.286034003	5.121118735	3.956800781	8.70E-05	0.007695334

CST2	-0.421359334	1.58351153	-3.956787233	8.70E-05	0.00764337
CNKSR1	0.228610013	3.342211995	3.956478078	8.71E-05	0.006457612
EFCAB1	-0.271783797	0.547456919	-3.954106332	8.80E-05	-0.002636285
PDIA4	0.254332622	7.127944506	3.954037228	8.80E-05	-0.002901172
IGF2BP1	0.340091637	0.503708422	3.953549379	8.82E-05	-0.004771047
CMBL	0.487652119	3.637267945	3.952487427	8.86E-05	-0.008840655
CCNB1IP1	0.221586046	3.220786617	3.952364926	8.86E-05	-0.009310035
CTD-2021H9.3	0.400975985	0.780352791	3.952094839	8.87E-05	-0.010344867
AARS	0.200752951	5.245361256	3.951944903	8.88E-05	-0.010919317
COX7A2	0.23647158	5.490982037	3.951873819	8.88E-05	-0.011191651
SELE	-0.20248445	0.592899419	-3.95039656	8.93E-05	-0.016850261
CPSF1	0.246302342	4.199145542	3.949881816	8.95E-05	-0.01882151
FABP6	-0.428680126	1.696308445	-3.949674191	8.96E-05	-0.019616559
C9orf24	-0.437558819	1.16059326	-3.949202579	8.98E-05	-0.021422329
CD302	-0.275712699	2.088540383	-3.948810945	8.99E-05	-0.022921719
LGMN	-0.236341061	5.853577076	-3.948071344	9.02E-05	-0.025752931
SMKR1	0.430961182	1.688795797	3.947630196	9.03E-05	-0.027441418
COA3	0.228975793	5.018008326	3.947394514	9.04E-05	-0.028343417
ITPR1	-0.255405672	1.980510267	-3.94327945	9.19E-05	-0.044084364
ARHGEF40	-0.223083561	2.265635729	-3.941623627	9.25E-05	-0.05041387
TESC	0.762336382	4.05669803	3.940400903	9.30E-05	-0.055086221
HHLA2	-0.669850715	1.55598104	-3.938921392	9.36E-05	-0.060738
C11orf96	-0.375635587	3.409684824	-3.93856911	9.37E-05	-0.062083437
RP11-513G11.4	0.252874675	0.872834797	3.937549149	9.41E-05	-0.065978232
FAM102A	-0.233719307	4.025539865	-3.935719407	9.48E-05	-0.07296286
F11R	0.201848552	5.454243068	3.935211287	9.50E-05	-0.074901953
DECR2	0.219229035	2.678580398	3.934631178	9.52E-05	-0.077115484
DENND2A	-0.219734031	1.350679591	-3.929101112	9.73E-05	-0.09820125
MAGEA1	0.423625143	0.457712424	3.928931499	9.74E-05	-0.098847534
TBX15	0.388933672	1.507808496	3.92787256	9.78E-05	-0.102881852
WI2-87327B8.2	-0.22817159	0.648496726	-3.927861233	9.78E-05	-0.102925001
SLC38A8	0.217630189	0.232573965	3.925308984	9.88E-05	-0.11264427
CLIP4	-0.214771406	2.408306927	-3.924699584	9.91E-05	-0.114964062

ENC1	-0.310589648	3.969573382	-3.924558147	9.91E-05	-0.115502416
DCK	-0.214833964	3.323646597	-3.923080892	9.97E-05	-0.121124257
ZNF512B	0.253339739	3.191096189	3.922897769	9.98E-05	-0.121821013
SHISA3	-0.662280918	1.678490582	-3.922714925	9.99E-05	-0.122516674
RPP40	0.200818607	2.018325326	3.919822625	0.000101051	-0.133516884
SLC16A7	-0.257857971	1.270293977	-3.919099454	0.000101346	-0.136266112
TSPAN19	-0.218784107	0.333166403	-3.918526029	0.00010158	-0.138445719
RPL10AP6	0.222746637	1.917613209	3.917675427	0.000101928	-0.141678335
SLC26A6	0.229836	2.02154729	3.917346713	0.000102063	-0.1429274
LDHD	0.354271693	2.220348417	3.916533201	0.000102397	-0.146018199
C11orf53	0.319127347	0.438509399	3.916393498	0.000102455	-0.146548917
WWC2	-0.207023822	1.98116089	-3.91608566	0.000102582	-0.147718296
MET	-0.566338659	4.584485207	-3.915596473	0.000102784	-0.149576387
OAT	0.309262447	5.168263017	3.914716854	0.000103148	-0.152916918
SPAG5	0.329673459	2.555879381	3.913648944	0.000103591	-0.156971575
RSPO4	-0.248303632	0.724875931	-3.910629756	0.000104856	-0.168429245
ATP2A2	0.205503166	4.928906905	3.908670303	0.000105684	-0.175860826
MAGEH1	-0.250109751	3.789496627	-3.908664818	0.000105686	-0.175881622
CDC25C	0.231151075	1.217684213	3.90854241	0.000105738	-0.176345759
RP11-295M3.4	-0.538393729	1.845949874	-3.906623283	0.000106556	-0.183620776
PODXL2	0.541898144	4.097929858	3.906218098	0.000106729	-0.185156321
HIST1H4I	0.309723105	3.257339834	3.904569463	0.000107437	-0.191402668
HOXB4	-0.254903149	1.000630486	-3.9043119	0.000107548	-0.192378295
EXO1	0.286049298	1.551799357	3.903772832	0.000107781	-0.194420053
NDNF	-0.538501859	3.720088389	-3.903248163	0.000108008	-0.196407017
RNF19A	-0.269689263	4.040001927	-3.902806377	0.0001082	-0.198079901
DNAH9	-0.213281633	0.32069169	-3.902567759	0.000108303	-0.198983388
C6orf132	0.226513492	2.644760739	3.902559139	0.000108307	-0.199016025
BTG2	-0.393394428	5.496078791	-3.901173354	0.00010891	-0.204262022
SRC	0.206331345	3.905496952	3.898206019	0.000110213	-0.215489215
FOXC1	-0.329667086	1.879278361	-3.89686696	0.000110805	-0.22055304
ACAT2	0.214122298	2.681107578	3.893717454	0.000112211	-0.232456853
RP11-115H13.1	0.211844019	0.311514194	3.892703006	0.000112667	-0.236289114

F2	0.251512281	0.237902641	3.892473579	0.000112771	-0.237155688
ORC6	0.229981852	1.402249048	3.891839165	0.000113057	-0.23955169
IFITM2	-0.291529605	6.211081226	-3.891798454	0.000113076	-0.239705433
EFHC2	-0.267100229	0.966860099	-3.890194119	0.000113804	-0.245762854
PTPN13	-0.441994136	2.62306609	-3.889907792	0.000113934	-0.246843684
HMGN4	-0.210824328	5.290518178	-3.889224602	0.000114246	-0.249422285
H6PD	-0.224559947	3.848451581	-3.888686626	0.000114492	-0.251452496
FKBP3	0.210620525	4.489584065	3.886802249	0.000115357	-0.25856167
DAW1	-0.272753193	0.741846506	-3.885897372	0.000115775	-0.261974339
PPP1R13B	0.246831393	2.755811998	3.885719309	0.000115857	-0.262645799
RP11-98D18.3	0.229064235	1.687952329	3.883095351	0.000117078	-0.27253719
FAM222A	0.21699834	0.91848308	3.881626414	0.000117766	-0.278071814
RBPMS2	0.304146906	1.580388768	3.880370218	0.000118358	-0.282803319
AURKAIP1	0.211307028	5.44056229	3.879994644	0.000118536	-0.284217651
SGK2	0.246321288	0.824764916	3.879245805	0.00011889	-0.287037232
BPGM	-0.225313517	3.552008909	-3.877842502	0.000119557	-0.292319672
CHTF18	0.245537562	1.918930267	3.876110812	0.000120386	-0.298835775
PNKD	0.243983375	4.711207606	3.874172059	0.00012132	-0.306127779
TRPM2-AS	0.228099244	0.514831047	3.873867094	0.000121467	-0.307274497
C11orf84	0.218057352	3.30890825	3.873433018	0.000121677	-0.308906544
PRDX1	0.247901236	7.779782815	3.872409619	0.000122174	-0.312753657
FOXS1	-0.202219574	1.24174023	-3.871127034	0.0001228	-0.31757374
CDHR4	-0.311194268	0.642367316	-3.869650119	0.000123524	-0.323122275
CHAC1	0.256319545	1.724388197	3.867899549	0.000124388	-0.329696305
GPER1	-0.21335804	0.882903549	-3.86268572	0.000126994	-0.349259545
ATP13A4	-0.487983588	2.213079509	-3.8620046	0.000127338	-0.351813394
ITGB8	-0.369560986	1.466696563	-3.861821453	0.000127431	-0.35250003
FSTL4	0.435338321	1.303222941	3.861704098	0.00012749	-0.352939988
MIR23A	-0.275153637	0.839153456	-3.861576231	0.000127555	-0.353419344
RP11-187E13.1	0.224869291	0.39768752	3.861138343	0.000127777	-0.355060804
ELANE	-0.222437063	0.382274722	-3.860654955	0.000128023	-0.35687262
RP11-379K22.3	0.263135927	0.414030338	3.859455663	0.000128634	-0.361366844
RP11-56A10.1	-0.270096022	0.675132605	-3.85864723	0.000129048	-0.364395621

LRRN4	-0.542798681	2.268476677	-3.857803671	0.000129481	-0.367555357
HMGCS2	0.387677745	0.59327386	3.857577223	0.000129598	-0.368403458
IGHD1-26	-0.268010964	0.422372238	-3.855433887	0.000130705	-0.376428431
RASD1	0.639821974	4.764572939	3.855318469	0.000130765	-0.376860455
KIAA0319	0.299866184	0.752007557	3.855244727	0.000130803	-0.377136475
MYH9	-0.256062873	7.140234295	-3.853237577	0.000131849	-0.384647389
MRPL55	0.207343274	4.251094289	3.853057124	0.000131943	-0.385322481
MAFG	0.207745457	3.131228582	3.852304465	0.000132338	-0.388137916
ADI1	0.209470589	4.367619612	3.851498959	0.000132761	-0.391150459
GARS	0.20254845	5.01439159	3.850949642	0.000133051	-0.393204533
RNU6-850P	0.291604374	1.418384745	3.85082846	0.000133114	-0.393657633
CTD-2006H14.2	0.218622126	1.918869675	3.850768208	0.000133146	-0.393882912
PDE4D	0.336550381	1.957818488	3.848764407	0.000134208	-0.401373096
FOXI3	0.337165597	0.607294941	3.848484007	0.000134357	-0.402420934
PGAP3	0.250987295	4.029255745	3.846070124	0.000135648	-0.411438504
TRIL	-0.301058606	1.427664652	-3.843640152	0.000136959	-0.420510795
INTS1	0.24759615	4.178685151	3.841988334	0.000137858	-0.426674766
TTC39C	0.263918486	2.281302028	3.838837985	0.000139586	-0.438423779
PVRL3	-0.301088955	1.522452627	-3.838027438	0.000140034	-0.44144519
SEMA3G	-0.203224957	1.341478917	-3.838027398	0.000140034	-0.441445338
ZMIZ1	-0.205501602	3.279633452	-3.836900575	0.00014066	-0.445644709
POLE2	0.231725492	1.554014652	3.836445929	0.000140913	-0.447338724
TMEM190	-0.449652131	1.135204221	-3.835630217	0.000141368	-0.450377601
ALDH6A1	0.235239786	2.739763522	3.835550987	0.000141412	-0.450672733
HDHD3	0.220021999	3.891371901	3.835072032	0.00014168	-0.452456728
VWA3A	-0.248587058	0.46431153	-3.834832255	0.000141814	-0.453349762
SPRR1B	-0.692884937	1.509974119	-3.834726101	0.000141873	-0.453745106
ORC1	0.264282484	1.592961157	3.833942152	0.000142313	-0.456664431
MIR4645	-0.284701461	1.070911224	-3.833695748	0.000142452	-0.457581891
OSMR	-0.326989505	4.761046156	-3.832465345	0.000143146	-0.462162338
PDGFB	-0.255698235	2.698525741	-3.832085881	0.000143361	-0.463574697
CLDN1	-0.483675404	4.056611049	-3.827898999	0.000145749	-0.479149457
KLHL35	0.237087109	0.981161401	3.827024948	0.000146252	-0.48239881

RPL37	0.225708304	6.402804659	3.824423069	0.00014776	-0.49206736
DPM3	0.316377098	5.177060835	3.824374793	0.000147789	-0.492246696
MANSC1	0.269826731	2.702016373	3.823880915	0.000148077	-0.494081217
CXADR	0.258190424	3.199507364	3.823214769	0.000148466	-0.496555278
KCNH2	0.3492733	1.123118722	3.821277744	0.000149603	-0.503747071
S1PR3	-0.205532535	1.586034941	-3.819350409	0.000150743	-0.510899476
IFT57	-0.356739482	4.320776205	-3.815207641	0.000153221	-0.526261909
MRPS35	0.220571189	5.114097396	3.814976772	0.00015336	-0.527117566
PAM	-0.266000003	4.712265917	-3.814852841	0.000153435	-0.527576868
CCNO	0.383422394	2.323506573	3.814295275	0.000153772	-0.529643082
XRCC2	0.21657122	1.186008073	3.814149187	0.00015386	-0.530184402
RNU6-140P	0.413848403	0.676451363	3.813717355	0.000154122	-0.531784424
CAD	0.225657078	3.165492159	3.812393941	0.000154926	-0.536686857
IGHD1-1	-0.307401439	0.587604358	-3.80911291	0.000156936	-0.548834137
TGFB2-AS1	-0.243722216	0.82617623	-3.808387113	0.000157384	-0.551519903
TEF	-0.26046888	2.694976222	-3.80611331	0.000158796	-0.55993085
ESR1	-0.238265941	0.79107667	-3.802208928	0.000161248	-0.574362361
ABCC9	-0.207634791	1.092255843	-3.801978199	0.000161394	-0.575214751
ZNF608	-0.232472523	1.872458203	-3.799740015	0.000162816	-0.583480832
PCSK2	0.593451458	0.814998817	3.799353456	0.000163063	-0.584908009
RP5-1057J7.7	0.215004084	0.943026996	3.799298326	0.000163098	-0.58511154
DEGS2	0.36202902	1.481299407	3.798142427	0.000163839	-0.589378247
CYP2J2	0.234566072	1.805450067	3.798098248	0.000163867	-0.589541299
GGT7	0.261735622	3.356462955	3.793882881	0.000166596	-0.605090711
NWD1	-0.242841611	0.538633075	-3.79337399	0.000166928	-0.60696678
PDE4A	-0.203700914	2.7309716	-3.791751656	0.000167991	-0.612946055
PRKCA	-0.282551598	1.987871079	-3.791463595	0.000168181	-0.614007481
SMIM22	0.399415956	4.421409732	3.789922006	0.000169198	-0.61968652
SEL1L3	-0.231401769	4.798265241	-3.789915417	0.000169203	-0.619710791
KCNJ15	-0.444551494	2.111242293	-3.786011034	0.000171806	-0.634084323
RP11-291L22.7	0.239086471	1.766689438	3.785621072	0.000172068	-0.635519152
SPAG6	-0.367278889	0.991621997	-3.78538572	0.000172226	-0.636385044
VAMP5	-0.386375679	5.673177075	-3.783970581	0.000173181	-0.641590446

ARID3A	0.231277891	1.795515763	3.782561057	0.000174137	-0.646773374
IGLV2-33	-0.248239791	0.696449141	-3.78182139	0.000174641	-0.649492455
ZC3HAV1L	0.203579321	1.943750169	3.779521313	0.000176216	-0.657944537
AC093838.4	0.239739684	2.487788098	3.778839609	0.000176685	-0.660448657
VWA5A	-0.27128548	2.886354108	-3.778210932	0.000177119	-0.662757616
SLC38A3	0.215307142	0.326838061	3.777541555	0.000177583	-0.665215655
KCTD1	0.206817939	1.408342822	3.776660625	0.000178194	-0.668449916
IGKV6D-41	-0.202816394	0.297081845	-3.776034404	0.00017863	-0.670748603
C16orf13	0.219473014	4.793615989	3.775573755	0.000178951	-0.67243929
PTPRB	-0.238546585	1.724135546	-3.774461598	0.000179728	-0.676520357
LIMA1	-0.215428062	3.92402994	-3.774033062	0.000180029	-0.678092572
WLS	-0.374724183	3.991171829	-3.772801691	0.000180895	-0.682609287
GPR98	0.235786571	0.795685825	3.771758127	0.000181632	-0.686436028
AC007879.7	-0.298843221	0.889981394	-3.771720445	0.000181659	-0.68657419
RP11-713M15.2	0.368713444	1.879717431	3.770248871	0.000182703	-0.691968695
GOLM1	0.305971855	5.981951712	3.770196692	0.00018274	-0.692159935
RPLP0	0.215056106	8.289632858	3.76951682	0.000183225	-0.69465151
COL21A1	-0.288463725	1.187440643	-3.769038659	0.000183566	-0.696403606
COLEC11	0.231165523	0.706464062	3.768389997	0.00018403	-0.698780123
NEK2	0.343498625	2.328624606	3.767289464	0.000184821	-0.702811287
PRSS16	0.206090894	2.406887418	3.765805027	0.000185891	-0.708246896
MSLNL	0.35061992	0.769994218	3.763654649	0.000187453	-0.716117413
GINS4	0.214824326	1.120485551	3.76113933	0.000189295	-0.725318252
CATSPERB	0.221139527	0.700956547	3.761069154	0.000189347	-0.725574868
SFTPA1	-1.091489257	7.880445059	-3.760664523	0.000189645	-0.727054409
UGCG	-0.204082366	4.235753019	-3.759759855	0.000190313	-0.730361796
CTD-2591A6.2	0.219061729	0.341893654	3.759183325	0.000190739	-0.732469149
VGF	0.339682234	0.548070886	3.757468795	0.000192014	-0.738734355
RPL37P2	0.236265115	1.295899745	3.757179798	0.00019223	-0.739790139
FAM69B	0.352644547	2.544414442	3.756674719	0.000192607	-0.741635141
NUP155	0.221182147	2.727984211	3.755874581	0.000193206	-0.744557484
NR4A3	-0.314589785	1.281771385	-3.753390911	0.000195078	-0.753624841
RPS24	0.216318257	7.371429448	3.751244745	0.000196709	-0.76145548

RP11-675F6.3	0.205144175	0.302770799	3.750226477	0.000197487	-0.76516932
FNDC3B	-0.20112443	3.447255277	-3.74976278	0.000197843	-0.766860201
HJURP	0.337953104	2.111428066	3.749759673	0.000197845	-0.766871533
DNAJB4	-0.24439542	2.370599464	-3.745527632	0.000201116	-0.782294654
OXLD1	0.208925116	3.817022052	3.742235183	0.000203697	-0.79428217
SYCE3	0.242807377	1.044311512	3.74223302	0.000203698	-0.794290043
LINC00973	-0.386836979	0.59639467	-3.741708923	0.000204112	-0.796197315
GOLIM4	-0.212613349	3.807147228	-3.741689932	0.000204127	-0.796266421
SLC43A3	-0.31669943	3.908144793	-3.741381682	0.000204371	-0.797388067
IGHD6-13	-0.277200059	0.44912368	-3.740953095	0.00020471	-0.798947445
PEX6	0.25731758	3.548829046	3.740163024	0.000205337	-0.801821612
FAAH	0.260952236	3.168108862	3.738398027	0.000206743	-0.808240349
PKIB	-0.375668136	1.95740244	-3.736964386	0.000207893	-0.813451938
NXPH4	0.410911137	1.14011031	3.733767402	0.000210477	-0.825066854
CREG1	-0.210518416	5.689121435	-3.733301702	0.000210856	-0.826757998
CELSR3	0.238895781	1.077971267	3.732511811	0.0002115	-0.829625954
CHST10	0.228906908	1.897675347	3.732398967	0.000211593	-0.830035624
CLPSL2	0.266155899	0.66602971	3.730959118	0.000212772	-0.835261835
LRRC36	-0.253982748	0.695483403	-3.727650957	0.000215506	-0.847262231
BPIFB2	0.638359173	1.271272321	3.726138528	0.000216767	-0.852745234
AKAP13	-0.235457698	3.265592174	-3.725555892	0.000217255	-0.854856901
SOCS2	-0.29999736	1.815581807	-3.725431606	0.000217359	-0.855307311
SLC45A3	-0.249965347	2.149815419	-3.724459139	0.000218176	-0.858831048
GINS2	0.287822082	2.044176701	3.724414213	0.000218213	-0.858993817
THBS4	-0.425381346	1.214884771	-3.723872576	0.000218669	-0.860956041
TARBP1	0.219569514	2.799914804	3.721810747	0.000220414	-0.868423093
IVL	-0.528284189	1.246034201	-3.720328382	0.000221676	-0.873789162
GADD45G	0.386034726	3.575551077	3.718677755	0.00022309	-0.879761951
GLDC	0.311944467	0.790089267	3.717916411	0.000223745	-0.882516025
RNU1-106P	0.24728658	1.219878297	3.717745365	0.000223892	-0.883134691
ZYG11A	0.203893371	0.695448895	3.716289344	0.00022515	-0.88839997
SLC25A25-AS1	0.216128727	1.220759336	3.715490351	0.000225843	-0.891288468
RALGPS2	-0.202780558	2.180677528	-3.715095983	0.000226186	-0.892713959

RP11-455F5.3	0.275633209	0.987391445	3.714925218	0.000226335	-0.893331163
NID1	-0.308300516	3.752846126	-3.713829782	0.000227291	-0.897289828
RP11-408B11.2	0.219980792	0.302986816	3.71345094	0.000227622	-0.898658624
GPR56	0.313116934	4.343790204	3.713017641	0.000228002	-0.900224015
DNAI2	-0.262526723	0.516533726	-3.712846562	0.000228152	-0.900842031
TNFRSF18	-0.376527931	2.465033384	-3.711702099	0.000229158	-0.904975651
SLC6A20	-0.316658532	0.83739789	-3.710714795	0.000230029	-0.908540673
PODXL	-0.232054123	3.535115199	-3.709280548	0.0002313	-0.913717946
EFCC1	-0.205880728	1.182510009	-3.708800663	0.000231727	-0.915449787
CERCAM	-0.288624154	3.271698981	-3.708485373	0.000232008	-0.91658751
IPCEF1	-0.255995502	1.035385897	-3.707651855	0.000232752	-0.919594821
F12	0.255008656	1.385993365	3.703723733	0.000236288	-0.933758773
SPSB1	-0.22930745	4.182609582	-3.702655768	0.000237259	-0.937607169
PLEKHH2	-0.239850504	1.479677517	-3.701896437	0.000237951	-0.940342766
HBA2	-0.482040693	2.473923862	-3.700908492	0.000238854	-0.943901182
FCHO2	-0.218323603	3.373923542	-3.697169465	0.000242302	-0.957360408
ATP5J2	0.22285119	5.354381744	3.696928979	0.000242525	-0.958225635
CCNA1	-0.206148672	0.483373054	-3.696741577	0.0002427	-0.958899841
MGST1	0.328270829	5.116091971	3.696533761	0.000242893	-0.959647448
MYBPH	-0.372936138	0.883866212	-3.696386827	0.00024303	-0.960176012
ID3	-0.288774244	4.464717538	-3.695632081	0.000243733	-0.962890736
VEPH1	-0.314352738	1.362116729	-3.694157125	0.000245114	-0.968194448
SOX2	0.563672737	2.349699722	3.693981841	0.000245279	-0.968824608
IDH1	0.24669565	5.162971152	3.693178126	0.000246034	-0.971713668
SLC4A3	0.2534186	1.237772884	3.693158335	0.000246053	-0.971784801
Six3os1_1	0.234403104	0.451823384	3.690382001	0.000248681	-0.981760073
TTTY15	0.235469554	0.538211143	3.688287112	0.000250682	-0.989282238
SERF2	0.200740278	5.838731294	3.687140545	0.000251783	-0.99339753
RP1-86C11.7	0.229423074	0.779818329	3.684066104	0.000254759	-1.004426425
ZFP57	-0.219199121	0.522054526	-3.683970273	0.000254852	-1.004770057
MORN5	-0.339526925	0.818308934	-3.682021582	0.000256756	-1.011755885
HSD11B2	0.30255541	1.857403403	3.681574797	0.000257194	-1.013357065
HNF1A	0.203956908	0.437551781	3.679578764	0.000259161	-1.020508159

PLAC8	-0.399546869	2.010192445	-3.678818312	0.000259915	-1.023231627
RP11-138J23.1	0.211111233	0.41242155	3.677709616	0.000261016	-1.027201335
HOXD13	0.2193735	0.210309673	3.677568688	0.000261157	-1.02770585
SYT12	-0.410539738	1.335999681	-3.676044249	0.000262679	-1.033162089
PALM	-0.252496646	2.082831731	-3.674533741	0.000264197	-1.038566354
UNC13B	0.319931897	4.073594514	3.674416273	0.000264315	-1.038986537
S100A10	-0.33158171	8.007779763	-3.673465641	0.000265274	-1.042386507
E2F8	0.227598792	1.447672277	3.673266971	0.000265475	-1.043096954
TRIM16	0.266979663	2.060210868	3.671422906	0.000267347	-1.049689607
IFITM1	-0.398738665	6.592403814	-3.670533522	0.000268254	-1.05286809
PABPC1L	0.356012137	2.76398881	3.667092408	0.000271792	-1.06515909
SLC24A3	-0.291261094	1.750641901	-3.666266192	0.000272647	-1.06810855
ZNF281	0.203342532	3.473285817	3.662726303	0.000276343	-1.080738262
CRYAB	-0.274156477	1.926830242	-3.662271507	0.000276821	-1.082360059
GNG4	0.39253712	0.925928167	3.661720627	0.000277401	-1.084324234
ME1	0.340971644	3.275074238	3.660553121	0.000278635	-1.088486075
GPI	0.20417764	5.328738212	3.658015568	0.000281334	-1.097527428
RPS20	0.204693437	8.021172444	3.655757901	0.000283756	-1.105566549
DSCR8	0.232968545	0.229722142	3.655680041	0.00028384	-1.105843709
SLC15A2	-0.322175359	2.092513944	-3.655220225	0.000284335	-1.107480417
NCAPG2	0.247216611	2.319099389	3.654325507	0.000285302	-1.11066459
SIX2	0.416454556	1.367915418	3.652504559	0.00028728	-1.117142807
FBXL19	0.203419579	2.802579069	3.652427206	0.000287364	-1.117417931
CAMP	-0.210710265	0.582574483	-3.649983054	0.00029004	-1.126108273
SNORD104	0.374956091	3.703511505	3.649022575	0.000291098	-1.129521812
C9orf171	-0.241482253	0.513304574	-3.648895361	0.000291238	-1.129973864
SIVA1	0.200382992	3.625592411	3.648725629	0.000291425	-1.130576983
CCDC42B	-0.301729401	0.852770714	-3.64798938	0.00029224	-1.133192832
ST6GAL1	-0.281665452	3.840013339	-3.647180339	0.000293137	-1.136066726
ARFGAP1	0.202717525	4.017688235	3.646299409	0.000294117	-1.1391953
MYCL	0.293610641	1.757663242	3.646092087	0.000294348	-1.139931489
KIAA1244	0.270837152	2.333078684	3.645590806	0.000294907	-1.141711345
OVGP1	0.23572463	1.359442209	3.643493559	0.000297258	-1.149155349

CHRD	0.29579874	1.348537977	3.643138987	0.000297657	-1.150413473
SPC25	0.253787786	1.726870796	3.64308908	0.000297713	-1.150590547
BEX1	0.391089294	0.636546672	3.642225114	0.000298688	-1.153655619
HIGD1B	-0.266252466	1.816748092	-3.640941729	0.000300142	-1.158207383
RP11-806H10.4	0.203838459	1.059766421	3.640191291	0.000300995	-1.160868246
RP3-395M20.8	-0.204234045	0.933542231	-3.640040352	0.000301167	-1.161403373
SCTR	-0.491789931	2.086795821	-3.637357473	0.000304237	-1.170911556
GLI4	0.221515624	2.290298741	3.635973615	0.000305833	-1.175813382
APCDD1L	-0.201733099	0.502401708	-3.635179679	0.000306752	-1.178624822
CLGN	0.342982373	1.118201554	3.63348116	0.000308726	-1.184637559
JAG1	-0.282757624	2.642175717	-3.633440412	0.000308773	-1.184781773
MYH14	0.3135215	4.344312304	3.6325394	0.000309826	-1.187970229
LYPLA1	0.252021538	4.411959315	3.630270649	0.00031249	-1.195995452
RP11-403P17.6	-0.241544291	1.80694505	-3.629644941	0.000313229	-1.198207926
RCC1	0.202756088	4.118216212	3.629439813	0.000313471	-1.198933169
SPRY1	-0.236932911	3.580292446	-3.627863341	0.000315341	-1.204505593
NASP	0.203430976	3.714197848	3.627325852	0.000315981	-1.206404953
B4GALT4	0.20365085	2.438530086	3.625385672	0.000318301	-1.213258879
RAD54L	0.246687581	1.445930626	3.624198925	0.000319727	-1.217449493
SGPP2	-0.386597351	4.355849054	-3.623348299	0.000320754	-1.220452404
PROSER2	0.21586566	2.479517142	3.622731157	0.0003215	-1.222630646
SLC15A1	0.277622762	0.654505004	3.621412344	0.000323101	-1.227284294
FGB	0.975671726	2.207247513	3.619022667	0.000326021	-1.23571257
FOXO6	0.291427792	1.392258667	3.618953756	0.000326106	-1.235955535
CCDC64B	0.294067575	2.691831282	3.618792336	0.000326304	-1.236524656
KIF1A	0.477865509	0.891196767	3.618718941	0.000326394	-1.236783417
CENPF	0.318930404	2.222772488	3.617125193	0.000328358	-1.242401108
SMAD9	0.229054287	1.1387666	3.616875675	0.000328666	-1.243280407
RP11-157P1.4	0.299258522	2.037722932	3.616333102	0.000329337	-1.245192223
MIR635	-0.213573854	2.59826035	-3.612945015	0.00033356	-1.257124376
PABPC1	0.220640194	8.261754908	3.612013891	0.000334729	-1.260401746
ZBBX	-0.208898431	0.436094661	-3.608927867	0.000338632	-1.271258204
EDN1	-0.381220788	3.322266226	-3.608544639	0.000339119	-1.272605763

MAFK	0.325305008	3.578154557	3.606711666	0.000341461	-1.279049242
RP3-438O4.4	0.212105117	0.295045941	3.603808061	0.000345201	-1.28924997
CCT2	0.241546334	5.067097686	3.602481429	0.000346923	-1.293907999
GCLC	0.468207388	3.049656456	3.60060398	0.000349373	-1.300497261
ST8SIA6-AS1	0.278761895	0.367814491	3.600061658	0.000350084	-1.302400035
CLASRP	0.200075773	2.932249543	3.599233085	0.000351172	-1.305306616
WNT5A	-0.279764767	2.109035489	-3.597575509	0.00035336	-1.311119378
RHOC	-0.218627628	5.426394492	-3.597193459	0.000353865	-1.312458786
MCM10	0.246371049	1.294007825	3.595137962	0.000356599	-1.319662717
PAX9	0.33558274	1.314278407	3.594172008	0.000357891	-1.323046762
SPTSSA	0.298410581	5.578572238	3.593050611	0.000359396	-1.326974288
CFB	-0.355308915	2.632629025	-3.592497422	0.00036014	-1.328911324
MIR34C	-0.270696958	0.484364332	-3.592284071	0.000360428	-1.329658311
NFKBIZ	-0.307088042	3.101206444	-3.591878049	0.000360975	-1.33107977
SMPDL3B	0.347966361	4.398402206	3.589810397	0.000363777	-1.338316121
PRTFDC1	0.257096464	2.291273344	3.589446227	0.000364273	-1.339590233
KIF2C	0.344750702	2.606363611	3.588764642	0.000365202	-1.341974543
GCLM	0.309973736	3.146749991	3.588290491	0.00036585	-1.343632955
EEF1A1	-0.200626275	9.385798518	-3.587461336	0.000366985	-1.346532548
RP3-340N1.2	-0.436729831	1.717548118	-3.586685239	0.000368051	-1.349246017
VPS9D1-AS1	0.281279514	1.770457761	3.585082688	0.00037026	-1.354847256
POLB	0.214500966	2.911522162	3.58465203	0.000370856	-1.356352088
KIF18B	0.296349289	1.735748798	3.584055567	0.000371683	-1.358436002
AREG	-0.621114776	3.8685346	-3.583907195	0.000371889	-1.358954332
PXDN	-0.356875806	3.384591244	-3.578914107	0.000378882	-1.376385513
C11orf97	-0.250206466	0.495614311	-3.577902068	0.000380314	-1.379915792
TRAF5	-0.209745567	1.935886576	-3.577453098	0.000380952	-1.381481624
CD99	-0.219408538	5.667003987	-3.577017494	0.000381571	-1.38300066
TXNRD1	0.46442099	5.17312013	3.576169179	0.000382779	-1.385958395
GPRC5C	0.360336464	3.416471767	3.574655009	0.000384944	-1.39123605
ZNF316	0.20904058	2.97089248	3.572689557	0.000387773	-1.398083487
TPBG	-0.248018218	2.721953292	-3.570175835	0.000391418	-1.406835826
RP11-279F6.2	0.281605932	0.38535628	3.569760659	0.000392023	-1.40828083

EBP	0.212890222	4.36996519	3.567404328	0.000395475	-1.416478945
CGNL1	-0.262482236	2.237883573	-3.566542458	0.000396744	-1.419476266
RP11-543P15.1	0.312092112	4.614166729	3.56628927	0.000397118	-1.420356646
RP13-401N8.1	0.283702124	1.352584378	3.564453671	0.000399837	-1.426737576
RP11-505K9.1	0.220261604	1.228029503	3.563329726	0.00040151	-1.430643106
HSD3B7	-0.218174989	3.241365331	-3.562844467	0.000402235	-1.43232894
FAM149A	0.232430125	1.41717966	3.560389517	0.000405919	-1.440854328
TPI1	0.200600346	7.626095121	3.55982375	0.000406772	-1.442818293
CALCA	0.882359822	1.335366714	3.558831947	0.000408273	-1.44626046
RP11-1260E13.1	0.412750739	1.021767155	3.558647447	0.000408552	-1.446900686
COX6A1P2	0.239194583	1.91725696	3.556570914	0.000411712	-1.454104223
GREB1	0.218036254	0.706677363	3.554311562	0.000415177	-1.46193742
KRT10	0.213475333	3.708322464	3.553998767	0.000415658	-1.463021512
ANXA8	-0.218472214	0.393716521	-3.552221107	0.000418406	-1.469180842
TOB1	0.281209079	4.78608447	3.549722241	0.000422298	-1.477834088
NEBL	-0.261579754	2.279214323	-3.547552461	0.000425704	-1.485343057
DNAJC12	0.54067728	2.763394548	3.54690502	0.000426725	-1.487582814
NME1-NME2	0.223784724	4.637765897	3.545880299	0.000428347	-1.491126942
CAPSL	-0.365615542	0.945193062	-3.544864568	0.000429959	-1.494639014
GRB14	0.325139228	1.710377808	3.544796692	0.000430067	-1.494873675
RP11-356K23.1	-0.298024253	0.691236204	-3.542930941	0.000433045	-1.501322235
LAMA5	0.31733062	3.703179737	3.542926721	0.000433052	-1.501336816
KIF14	0.213288934	1.138080682	3.542061236	0.00043444	-1.504327075
CRABP1	0.414786589	0.889477066	3.541657481	0.000435089	-1.505721816
FAM3B	0.463674537	2.628578149	3.538784118	0.000439733	-1.515643262
MT1F	-0.280556305	2.288402146	-3.53713373	0.000442422	-1.521338429
EHBP1L1	-0.214775128	3.851654466	-3.53671004	0.000443115	-1.522800094
SCGB3A1	-0.969070899	5.413209781	-3.534221136	0.000447204	-1.531383065
MIR5572	0.212553272	0.308771583	3.532079215	0.000450752	-1.538764866
LINC00578	-0.246687737	1.293006999	-3.532048422	0.000450803	-1.538870958
SLC7A5	0.4023973	4.40105306	3.531651943	0.000451463	-1.540236883
CSGALNACT1	-0.225600533	1.961382581	-3.531262292	0.000452112	-1.541579143
POU6F2	0.214944588	0.431896976	3.53086102	0.000452782	-1.542961291

MFSD6L	0.340315719	1.391652745	3.530204042	0.00045388	-1.545223868
CLDN5	-0.310160015	2.690480284	-3.529691931	0.000454737	-1.54698726
RP11-245P10.4	0.2407656	1.042536795	3.524723532	0.000463137	-1.564082697
PRR7	0.200022167	1.327953963	3.523758202	0.000464786	-1.56740158
MIR6835	0.227915895	0.952067878	3.522455344	0.00046702	-1.571879539
RPL36A	0.202332682	4.171460086	3.521536088	0.000468602	-1.575038102
AC009299.3	0.206878679	1.624433011	3.519901568	0.000471428	-1.580652373
SPATA18	-0.31718168	1.41490747	-3.519664563	0.000471839	-1.581466233
FBXL8	0.207888981	1.84339492	3.518514224	0.000473839	-1.585415695
DLK2	0.205806237	1.021004618	3.516765211	0.000476895	-1.591418231
SEC61G	0.206085666	5.154232623	3.514872011	0.000480224	-1.597912408
PDK3	-0.204917166	2.112889924	-3.51416083	0.00048148	-1.600351089
GSTA4	0.298095659	3.473167333	3.512807362	0.000483879	-1.60499091
INSL4	0.426667427	0.581825406	3.512559142	0.00048432	-1.605841646
ARVCF	0.232088169	2.085723814	3.512162258	0.000485027	-1.60720179
FZD4	-0.228270402	2.225018495	-3.511075577	0.000486965	-1.610925159
CYCS	0.209447306	4.762847295	3.511043349	0.000487023	-1.611035567
RP11-190J1.3	0.265598785	0.62343699	3.510768296	0.000487514	-1.611977817
CRIPAK	0.230053618	1.802934212	3.510357493	0.00048825	-1.613384976
PIDD1	0.200458297	1.961287742	3.508455789	0.000491668	-1.619897004
RPS7	0.226968132	6.369180662	3.507421926	0.000493536	-1.623435869
RP11-465B22.8	0.311094009	1.750024101	3.507083981	0.000494148	-1.624592421
CEP131	0.216402531	2.697307497	3.506670985	0.000494896	-1.626005679
TMPRSS13	0.290877486	2.617974302	3.50560858	0.000496828	-1.629640463
HSH2D	-0.221445766	2.873718581	-3.505119074	0.00049772	-1.631314847
KIF23	0.260337234	1.811146716	3.501431112	0.000504489	-1.643922587
MTL5	0.213020843	0.81651569	3.501310252	0.000504713	-1.644335549
MIR7111	0.206750891	1.079252014	3.499960246	0.000507213	-1.648947396
MLEC	0.205318506	5.277587676	3.498232225	0.000510431	-1.654848135
AGA	0.220966233	3.736046662	3.497497258	0.000511805	-1.657357013
MBOAT2	-0.26390083	2.911085533	-3.496588488	0.000513509	-1.660458493
TRGJP2	-0.267395895	0.673650308	-3.496041555	0.000514538	-1.662324713
KLK12	0.674817639	1.26782669	3.493078789	0.000520141	-1.672429299

PITPNC1	0.267453935	2.457201417	3.492796517	0.000520677	-1.67339157
NOVA1-AS1	0.213551798	0.438923898	3.490619372	0.000524835	-1.680811008
TTLL12	0.21120912	3.70354231	3.490564311	0.000524941	-1.680998592
PLS1	0.231929181	3.6116731	3.488968732	0.000528008	-1.686433244
LINC01207	0.322219174	1.022135676	3.488958821	0.000528027	-1.686466997
CTB-193M12.5	0.219424727	2.950910189	3.486914674	0.000531981	-1.693426028
PLA2G6	0.237915828	1.606804415	3.486298728	0.000533178	-1.695522175
SESN3	-0.271895849	2.648201099	-3.485445337	0.000534841	-1.698425799
TMSB4Y	0.204214492	0.468098901	3.484470708	0.000536745	-1.7017411
SNORA80B	0.30489835	0.672139239	3.484204906	0.000537266	-1.702645098
CACNA1D	0.21428879	1.024258281	3.48313983	0.000539356	-1.706266789
RP11-638I8.1	0.240043351	1.585007498	3.482758898	0.000540106	-1.707561859
CCNB2	0.310004771	2.787073312	3.481513477	0.000542563	-1.71179502
TARS	0.206244534	4.19817808	3.481494282	0.000542601	-1.711860253
TNRC18	0.218832325	3.947728822	3.479475392	0.000546607	-1.718719319
ABCA4	-0.402175347	1.218136105	-3.478948643	0.000547657	-1.720508297
WNT3	0.214634655	1.707323092	3.478772279	0.000548009	-1.721107218
ADCY9	-0.226755716	2.271512389	-3.478144961	0.000549262	-1.723237313
RP11-452F19.3	0.204774219	2.638290949	3.477427191	0.000550699	-1.725674096
DSTNP2	0.203039195	3.131175508	3.476225538	0.000553113	-1.729752557
PAPSS2	-0.297947187	4.175287881	-3.475954727	0.000553659	-1.730671518
GPRC5B	-0.234219344	1.613616167	-3.474249843	0.000557104	-1.736455237
SNORD69	0.219733825	1.173735385	3.47352209	0.000558581	-1.738923273
RP11-627G23.1	0.25752188	0.754375127	3.472970034	0.000559704	-1.740795136
TSPAN5	-0.247401852	2.282690498	-3.472729523	0.000560193	-1.741610553
GAREML	0.209509418	1.236483735	3.470955677	0.000563818	-1.747622843
FLT1	-0.214718111	2.348855255	-3.47093566	0.000563859	-1.74769067
SERPINB1	-0.261098851	6.007718255	-3.470600053	0.000564547	-1.748827847
RNF183	0.268377265	0.714063031	3.469973978	0.000565833	-1.750948966
BHLHE40	-0.273542145	6.15951199	-3.469879063	0.000566028	-1.751270501
PRAME	0.565523612	1.62581631	3.469001254	0.000567837	-1.754243793
MT-ND4	0.310108038	12.14359427	3.468916765	0.000568011	-1.754529936
RUSC1-AS1	0.207186823	1.501213933	3.468291933	0.000569302	-1.756645865

RP5-857K21.7	0.332533403	2.010990452	3.468085867	0.000569728	-1.757343608
MPZL2	-0.339156964	4.978565003	-3.467731076	0.000570463	-1.758544842
TPM2	-0.297560138	4.573435573	-3.467536402	0.000570867	-1.759203911
RP11-1260E13.2	0.230536719	0.864177561	3.466327213	0.000573379	-1.763296825
NOTCH1	-0.208516607	2.656822083	-3.465111449	0.000575915	-1.767410622
GCNT1	-0.271968342	1.852061988	-3.462717332	0.000580941	-1.775507612
ATP2B4	-0.250296792	4.176258635	-3.459763999	0.000587196	-1.785488545
FAM60A	0.237522637	3.696106311	3.457336278	0.000592386	-1.793687069
PLEKHF1	-0.23967486	2.648406511	-3.456925265	0.000593269	-1.795074536
PCSK1	0.52385452	0.792099794	3.45446236	0.000598584	-1.803385331
SNTN	-0.261221351	0.706431672	-3.453985957	0.000599618	-1.804992249
ALDOA	0.206020421	7.749501819	3.450411462	0.000607424	-1.817042355
IGLVI-70	-0.359952833	0.979590052	-3.44786866	0.000613035	-1.825607245
DCLK1	-0.229297315	1.136718657	-3.447513975	0.000613822	-1.826801451
FOXM1	0.34518176	2.709044734	3.447176307	0.000614571	-1.827938249
ARSJ	-0.242589463	1.790854328	-3.444059499	0.000621531	-1.83842634
ADCK3	0.206961956	2.778606964	3.443639863	0.000622474	-1.839837727
RAD51	0.213304349	1.607482137	3.44324609	0.000623359	-1.841161978
MAP2	0.31614189	1.737232569	3.443121138	0.000623641	-1.841582161
MARK1	0.251573805	1.223015946	3.441424214	0.000627473	-1.847287044
ZFP36	-0.352803964	6.58025592	-3.43784272	0.000635633	-1.859318851
D2HGDH	0.228913854	2.497691148	3.431198147	0.000651036	-1.881609244
MVP	-0.237835791	5.923299331	-3.431143583	0.000651164	-1.881792118
MIR210HG	0.23395742	1.957886692	3.430589584	0.000652464	-1.883648721
RP11-747H7.3	0.210611051	1.161247283	3.430463764	0.000652759	-1.884070337
NRBP2	0.264774312	3.054066853	3.427862093	0.000658899	-1.892785122
URB1-AS1	0.212441966	2.720520027	3.426398668	0.000662377	-1.897684365
ATP6V0E2	0.236763749	3.477243761	3.426076228	0.000663145	-1.898763559
AL590226.1	0.239973593	0.60533794	3.425509991	0.000664497	-1.900658497
RPL39P40	0.244320614	0.956225064	3.425509638	0.000664498	-1.900659679
MTND4P24	0.309771801	0.730529606	3.423196782	0.000670045	-1.908396659
APOLD1	-0.208251615	1.795247374	-3.423007819	0.0006705	-1.909028559
CTD-2017D11.1	0.211928616	1.340277391	3.420590483	0.000676348	-1.91710929

SKP2	0.241767556	2.506582491	3.41999149	0.000677804	-1.919110779
TTK	0.258773232	1.544972664	3.41856233	0.000681291	-1.923884852
RP11-46B11.2	0.324166863	1.039255509	3.417514186	0.000683859	-1.927384941
TENM1	0.250230216	0.476581033	3.416813646	0.00068558	-1.929723698
GAA	-0.235804837	5.094228999	-3.416639143	0.000686009	-1.930306206
PTPRM	-0.23342078	2.505275584	-3.415710991	0.000688297	-1.933403994
CCDC167	0.220447964	4.709601532	3.412065616	0.000697352	-1.94556297
CHIAP2	-0.209577173	0.34013758	-3.411346493	0.000699152	-1.947960109
NAT14	0.214256063	3.186027283	3.408487227	0.000706349	-1.957486469
NAPRT	0.299164011	4.049267267	3.408379411	0.000706622	-1.957845536
L1CAM	-0.227495775	0.430976123	-3.407956325	0.000707693	-1.959254463
GP2	0.302179685	0.306106266	3.407802989	0.000708081	-1.959765051
SGCE	-0.297348357	3.256003112	-3.406858702	0.000710479	-1.962908902
ZDHHC8P1	0.209765816	0.757196863	3.406075924	0.000712472	-1.965514406
RP11-93B14.10	-0.218316687	1.3479031	-3.40481849	0.000715685	-1.969698622
CAPN2	-0.209571512	5.755254061	-3.404537453	0.000716405	-1.970633595
C6orf118	-0.218065923	0.452777608	-3.403257614	0.000719692	-1.974890516
C1GALT1	-0.210633906	2.912002512	-3.402808382	0.000720849	-1.976384363
RP11-305L7.1	0.204174912	1.036725494	3.402755062	0.000720986	-1.976561655
BCAS1	0.399978031	1.546877303	3.402591809	0.000721407	-1.977104473
FEN1	0.231360016	3.764094214	3.402083082	0.00072272	-1.978795828
RPS21	0.24055416	8.344016951	3.40162611	0.000723902	-1.98031491
CGN	0.310721362	4.562544918	3.401469685	0.000724307	-1.980834859
SNHG8	0.216913565	5.142323955	3.400898259	0.000725788	-1.982734053
P2RY6	-0.322417647	2.207669162	-3.399116284	0.000730424	-1.988654686
RP11-390K5.1	0.22392144	0.87118556	3.397653861	0.000734249	-1.993511387
LINC01547	-0.223542401	1.372826595	-3.393762058	0.000744521	-2.006426336
CYP2S1	-0.379505627	2.822112624	-3.393305526	0.000745734	-2.00794041
CENPA	0.293303716	1.846716737	3.392871321	0.00074689	-2.009380261
PPIAP29	0.345161326	2.043036387	3.389380592	0.000756244	-2.020949322
CISH	-0.283251887	3.215646921	-3.389019255	0.000757219	-2.022146225
MYH10	-0.255420352	3.521003442	-3.386068109	0.000765222	-2.031917126
RDH10	0.392166243	4.022228218	3.384682274	0.000769007	-2.036502658

FAM154B	-0.226100871	0.69558911	-3.38340192	0.000772519	-2.040737579
CNGA3	0.334080148	0.736099078	3.380947041	0.000779296	-2.048853094
LINC01564	0.272377448	0.871775846	3.378295781	0.000786677	-2.057611503
KIT	0.467494866	3.04424417	3.376733135	0.000791057	-2.062770613
ATAD2	0.25530108	2.799109562	3.376189632	0.000792586	-2.064564468
CTH	0.206856063	1.868036967	3.375865379	0.0007935	-2.065634547
CCDC64	0.271538427	2.188652466	3.375313781	0.000795056	-2.067454669
OAS3	-0.290215959	3.822803075	-3.372377177	0.000803389	-2.077139873
CFAP52	-0.232613163	0.616474124	-3.370875695	0.000807681	-2.082088796
TP63	-0.321225695	1.021274307	-3.369952543	0.00081033	-2.085130482
SP5	0.336847422	1.447248847	3.369692091	0.000811079	-2.085988501
NCAPG	0.272610257	1.820861675	3.369263979	0.000812312	-2.087398708
TMSB4XP8	-0.381160088	4.615200242	-3.368268414	0.000815184	-2.09067746
PCOLCE2	-0.286894251	1.248509279	-3.367662792	0.000816937	-2.092671534
DDX39B	0.269765687	3.778670861	3.365265471	0.000823907	-2.100561608
RFTN1	-0.27426074	3.77124837	-3.36331753	0.000829612	-2.106968726
CERS4	0.322116253	3.305064946	3.362103112	0.000833186	-2.110961366
ST3GAL1	-0.291249923	3.377140869	-3.361266025	0.000835659	-2.113712654
TOX3	0.382864171	2.283625443	3.360736224	0.000837227	-2.115453634
ZMYND10	-0.318806098	1.227612791	-3.359906973	0.000839688	-2.118178113
C1orf53	0.248580379	2.735145423	3.359476083	0.000840969	-2.119593533
PLIN2	-0.295983211	3.850955562	-3.358670781	0.000843368	-2.122238387
RNA5SP283	-0.20333148	0.947182327	-3.357341765	0.000847342	-2.126601951
SKA3	0.234517702	1.599030375	3.356442685	0.000850039	-2.129552966
MST1P2	0.252464984	1.250828173	3.355020031	0.000854325	-2.134220949
RP11-434I12.3	0.313360592	0.87776505	3.354745986	0.000855152	-2.135119922
NEURL1	0.236565734	0.492256244	3.354404239	0.000856186	-2.136240886
PHLDA1	-0.303553583	3.33461356	-3.354106472	0.000857087	-2.137217504
RIPK2	-0.201681598	3.304410093	-3.350607561	0.000867744	-2.148687023
C1QTNF6	-0.24724344	2.287017337	-3.349807729	0.000870197	-2.151307286
ITGA3	-0.336547017	5.481643896	-3.349721858	0.000870461	-2.151588565
MAGEA10	0.32321804	0.359876822	3.347791144	0.000876412	-2.157911
RP11-109L13.1	-0.202968179	0.627091471	-3.346971393	0.000878949	-2.16059435

TFF1	0.857421302	2.271713776	3.346051623	0.000881805	-2.163604356
AC005077.14	0.246440962	0.902190191	3.345861079	0.000882398	-2.164227825
PRKAR2B	-0.267272864	2.022825018	-3.345843102	0.000882454	-2.164286645
CLDN18	-0.648842526	2.401840537	-3.345179152	0.000884522	-2.166458841
FHOD3	-0.227594146	0.839114373	-3.343563271	0.000889574	-2.171743682
HEYL	-0.210688024	2.613437363	-3.342776717	0.000892043	-2.174315271
SDS	-0.290567622	1.883634356	-3.341596226	0.000895761	-2.178173729
IL1RAP	-0.209008174	1.719362026	-3.339231102	0.000903252	-2.185900263
IL23A	-0.238548582	1.491407796	-3.335575609	0.000914945	-2.197831966
RP4-666F24.3	-0.209773334	0.446019413	-3.335446504	0.000915361	-2.198253143
MT-ND2	0.30836051	11.26957424	3.333774659	0.000920757	-2.203705759
ANO9	0.292403401	2.633228579	3.333773118	0.000920762	-2.203710783
TMEM233	-0.213718238	0.730720775	-3.33357462	0.000921405	-2.204357997
RP4-639F20.1	-0.213448477	2.347551822	-3.33343609	0.000921854	-2.204809662
POC1A	0.200972833	2.331883249	3.332662848	0.000924363	-2.207330412
CTNND2	0.318103947	0.586101964	3.332600279	0.000924566	-2.207534361
LGI3	-0.269060792	0.634929156	-3.32942541	0.000934936	-2.21787831
MLLT11	0.302296337	1.794793523	3.327437284	0.000941485	-2.224350967
RP11-43F13.1	0.238025221	2.154943349	3.327262268	0.000942063	-2.224920582
CDC20	0.372659752	3.797550343	3.325107491	0.000949213	-2.23193127
ALDH3A1	0.634888773	2.047955581	3.322956593	0.0009564	-2.238925005
RP11-304L19.3	0.344846432	1.562900575	3.321306649	0.000961948	-2.244286934
GPX1P1	-0.285704646	1.620944419	-3.318903464	0.000970082	-2.25209216
CCNB1	0.316040207	3.816897705	3.315528108	0.000981613	-2.263045743
ABHD17C	0.29436171	3.959041295	3.315527849	0.000981614	-2.263046584
CDC6	0.281735581	2.180086797	3.312851303	0.000990849	-2.271724835
RASL10B	0.215127016	0.794754394	3.311469054	0.00099565	-2.27620392
NOTUM	0.413482645	1.172570337	3.311340333	0.000996098	-2.27662094
RORC	0.309081628	3.148895421	3.309923197	0.001001044	-2.281211047
C1orf189	-0.287216935	0.840250416	-3.309681046	0.001001891	-2.281995185
ABCC2	0.440902644	0.884170266	3.307568729	0.001009312	-2.288833015
CHP1	0.210603593	5.309114043	3.307414271	0.001009857	-2.289332852
HN1	0.235222917	5.429823314	3.305182501	0.001017757	-2.296552507

TPD52L1	0.267358267	1.885836862	3.304809589	0.001019082	-2.297758402
BEX4	-0.283535827	4.216850038	-3.30173302	0.00103008	-2.307702217
IRX5	0.348190201	2.416425246	3.301597652	0.001030566	-2.308139535
MT-ND4L	0.281096538	10.4411212	3.299893135	0.001036709	-2.313644694
PKIA	-0.259512788	1.402705469	-3.298463999	0.001041886	-2.318258342
HOXC13	0.258099159	0.673685457	3.295816181	0.001051541	-2.326801185
MYO10	0.208837749	3.063936839	3.292951131	0.001062081	-2.336037498
RP1-27K12.2	0.357809054	0.647348893	3.29265914	0.001063161	-2.336978385
RP11-845M18.6	0.222869035	1.08890963	3.292327907	0.001064387	-2.338045622
NRGN	-0.445202485	3.756743242	-3.291503443	0.001067444	-2.340701615
CHEK1	0.212342357	1.720936059	3.290607516	0.001070776	-2.3435871
AMOTL1	-0.213091561	2.18561174	-3.286726988	0.001085318	-2.356076321
RP11-10A14.5	0.27923959	0.847506795	3.285654405	0.001089369	-2.359525869
TMEM229A	0.260379716	0.375046419	3.285353692	0.001090508	-2.360492804
CBR3	0.346253408	2.610871024	3.281841219	0.001103887	-2.371780781
AC012512.1	0.240148964	0.308242559	3.281745085	0.001104255	-2.372089561
C6orf52	0.200519816	1.077766671	3.280511224	0.001108993	-2.376051944
SFTPA2	-0.94222275	8.159513311	-3.278589771	0.001116408	-2.3822196
RPS7P1	0.255721141	3.026753805	3.278499013	0.001116759	-2.382510838
RHPN2	0.277075691	2.900358709	3.277291713	0.001121443	-2.386384265
CSAG3	0.259150664	0.397714313	3.275500626	0.001128425	-2.392128165
SPINK4	0.397742485	0.659524864	3.274000813	0.001134303	-2.396935653
FLNC	-0.327365228	1.483042544	-3.273630277	0.00113576	-2.398123043
MT-CO2	0.280484626	12.23867031	3.273202918	0.001137442	-2.399492362
LY6E	-0.357452219	6.931063124	-3.271500472	0.001144165	-2.404945541
ZFP42	0.271114035	0.414521135	3.271320227	0.001144879	-2.40552273
VSIG1	-0.590726621	1.987851927	-3.271169249	0.001145478	-2.406006179
EFNA4	0.215269848	4.009883662	3.268155838	0.001157482	-2.41565097
DEPDC1	0.237028252	1.345191993	3.267453932	0.001160295	-2.417896283
COL2A1	0.287871737	0.303708849	3.265544008	0.001167981	-2.424003565
CGA	0.381824142	0.398005005	3.264864771	0.001170725	-2.426174706
C16orf45	-0.233292999	1.876283478	-3.261925172	0.001182673	-2.435565977
FGFRL1	0.21948911	3.434439879	3.259368836	0.001193155	-2.443726232

LRP4	0.261194083	1.193829826	3.258957058	0.001194851	-2.445040123
MT2P1	-0.225637407	1.125164185	-3.257128646	0.001202411	-2.450872256
TMEM159	-0.254751914	4.529627099	-3.256759837	0.001203941	-2.452048276
EZH2	0.230961987	2.368967347	3.256279397	0.001205937	-2.453580061
TRPV4	-0.247524856	1.718039071	-3.256156081	0.00120645	-2.453973194
LRRC4	-0.272153782	1.418715492	-3.249212079	0.001235657	-2.476087748
MTMR7	0.265261954	0.707562798	3.247827611	0.001241558	-2.480491453
EPPK1	0.207125701	1.291940984	3.247036439	0.001244941	-2.483007198
ALG1L	0.38260142	2.751972932	3.242130833	0.001266114	-2.49859279
CTD-2600O9.1	0.211823526	0.916750801	3.242006473	0.001266655	-2.498987601
AKR1B10	0.782070409	2.181688732	3.241222214	0.001270072	-2.501477082
RP11-3P17.3	0.240653579	2.684315122	3.238465506	0.001282151	-2.51022315
RP11-304L19.1	0.32248899	1.538973142	3.237602528	0.001285954	-2.512959612
AQP7	0.223184788	0.806194104	3.236300496	0.001291711	-2.517086969
KISS1R	0.242512439	0.783199362	3.23373427	0.001303129	-2.525217076
SCD5	-0.291535321	2.064989758	-3.233204523	0.001305497	-2.526894609
XK	0.217058331	1.120808268	3.231527601	0.001313021	-2.532203122
DBN1	0.230732437	4.37774813	3.231414674	0.00131353	-2.532560513
TUBB4A	0.242867076	0.687108565	3.230572925	0.001317323	-2.535224094
PLEKHG6	0.240719914	1.924636898	3.230173647	0.001319126	-2.536487311
IDI1	0.200039751	4.123028583	3.228428334	0.001327033	-2.542007302
CTD-2547H18.1	0.295278728	1.084939857	3.228206214	0.001328043	-2.542709609
LINC00665	0.291653189	1.774975078	3.227634375	0.001330645	-2.544517452
JSRP1	-0.314175896	1.548871852	-3.227278623	0.001332266	-2.545641991
CELF3	0.238002338	0.337671848	3.226493881	0.001335849	-2.548122156
HCG4B	-0.239132517	1.320955664	-3.226234833	0.001337033	-2.548940745
SH3PXD2B	-0.232175754	3.156504961	-3.226070826	0.001337784	-2.549458975
PRR29	-0.224506552	1.280187836	-3.224364063	0.001345617	-2.554850497
C6orf222	-0.202851293	0.446195106	-3.219104251	0.001370025	-2.571448596
PLEKHB1	0.292053966	2.850969573	3.218898937	0.001370986	-2.572095966
RASSF7	0.254402022	4.645793665	3.216069531	0.001384294	-2.581013269
PHGDH	0.35762617	2.260024087	3.215535727	0.001386818	-2.582694792
PPP1R14C	-0.391425805	2.768599359	-3.213376696	0.00139707	-2.589493164

KLB	0.20649523	0.406094098	3.211139688	0.001407766	-2.596532454
MYOZ1	-0.204295671	0.995608269	-3.210592513	0.001410394	-2.598253558
RPL4P4	0.224298943	1.802760684	3.210539319	0.001410649	-2.598420861
B4GALNT2	0.26141387	0.519844942	3.208708085	0.001419478	-2.604178749
FREM2	0.203382623	0.902540038	3.208442394	0.001420763	-2.60501389
ERO1LB	0.310809098	2.216827032	3.208134518	0.001422254	-2.605981548
PGF	-0.208489447	1.42304382	-3.207672823	0.001424492	-2.607432494
AC092171.4	0.216218827	2.243218095	3.207658584	0.001424561	-2.60747724
SLC2A4RG	0.201285053	4.911429659	3.20683305	0.001428571	-2.610071094
ERVMER34-1	0.225019047	0.944422103	3.204343966	0.001440725	-2.617887998
RP11-303G3.6	-0.223434519	0.864208567	-3.204247816	0.001441197	-2.618189839
HLF	-0.322981286	1.622694336	-3.204179355	0.001441532	-2.618404748
PRKAG2-AS1	0.214666509	1.437920042	3.203172158	0.00144648	-2.621566014
RARG	-0.205259544	2.845268644	-3.202990356	0.001447375	-2.62213653
RP5-875H18.9	-0.252595962	0.863235959	-3.202875267	0.001447942	-2.622497678
PTPRE	-0.218888335	2.645787338	-3.202611607	0.001449241	-2.623324992
CFAP221	-0.2413582	1.201178763	-3.202495369	0.001449814	-2.623689701
SCG3	0.264058298	0.359581377	3.201120678	0.001456606	-2.628001999
PLCXD1	0.279281645	2.461487923	3.197658464	0.001473845	-2.638854819
GNPNAT1	0.208449598	3.374100331	3.196582379	0.001479241	-2.642225672
MCIDAS	0.212320408	0.930010798	3.195905554	0.001482645	-2.644345277
KLK13	0.384796144	0.930512429	3.195655311	0.001483905	-2.645128851
TIMM8AP1	-0.360078961	1.671598879	-3.193285408	0.001495888	-2.6525467
PMM1	0.220774523	3.136825266	3.193118479	0.001496736	-2.653068993
MAGEA3	0.533122982	1.042251631	3.188573263	0.001519981	-2.667280128
HMGA1	0.301882383	6.536767919	3.184681293	0.001540151	-2.679433376
IGF1R	0.234135929	2.646248805	3.184401703	0.001541609	-2.680305888
SEMA3A	-0.30969041	1.429643134	-3.182912986	0.001549396	-2.684950465
SNORD19	0.24632818	1.845637218	3.181903017	0.0015547	-2.688100233
FKBP5	-0.287562091	3.164883241	-3.180824251	0.001560383	-2.691463496
C1QTNF3	-0.253674576	1.471730799	-3.180459635	0.001562308	-2.692600011
AC006042.6	0.252244947	2.043356438	3.180131032	0.001564045	-2.693624165
GPR39	0.227242408	2.315576579	3.179951399	0.001564996	-2.694183982

COL17A1	-0.506772886	2.19817492	-3.179566494	0.001567034	-2.695383418
CHCHD2	0.203376165	7.501695662	3.178942106	0.001570345	-2.69732883
KIF26B	-0.233851584	1.95147641	-3.178850332	0.001570832	-2.697614742
ATP2C2	0.278513809	2.294221786	3.174961661	0.001591606	-2.709722124
GPR115	-0.292897601	1.130997612	-3.171306028	0.001611366	-2.721090964
NCS1	-0.211863476	2.276515472	-3.171210293	0.001611886	-2.721388526
CDCA8	0.28128377	2.935158442	3.171135811	0.001612291	-2.721620026
LINC01508	0.207626757	0.777257638	3.171102957	0.00161247	-2.721722136
JAG2	0.243788901	2.621956164	3.170887389	0.001613643	-2.722392109
RP11-76C10.6	0.371992156	1.895012941	3.170150891	0.001617657	-2.724680768
IRX3	0.368891449	3.800686264	3.166426928	0.001638092	-2.736245134
ATAD3C	0.247450681	1.828241537	3.161484135	0.001665584	-2.751574282
BMP4	-0.327977639	2.265751004	-3.160125343	0.001673216	-2.75578429
GAS2L2	-0.223171517	0.665428259	-3.157106748	0.001690286	-2.765130721
NDC80	0.254727841	1.909852889	3.156400011	0.001694305	-2.767317741
PRKDC	0.218194894	3.857967038	3.155461894	0.001699655	-2.770220048
GALNT18	-0.22397144	3.014165498	-3.153353105	0.001711736	-2.776741105
MPP7	0.216383867	3.08857129	3.151762518	0.001720902	-2.781656942
CAMK1D	-0.293458095	2.726948967	-3.149294138	0.001735214	-2.789280951
PTGR1	0.306690746	3.683911523	3.147928534	0.00174318	-2.793496384
MIR4292	0.255470328	2.366597593	3.147658021	0.001744762	-2.794331211
ENTPD2	0.243244014	1.151411195	3.147504321	0.001745661	-2.794805513
MIR25	0.285182619	2.209037355	3.146639276	0.001750731	-2.797474537
RN7SL2	-0.435413899	7.39340146	-3.143713889	0.001767977	-2.806495341
ASCL1	0.604656488	1.026531472	3.143592263	0.001768697	-2.806870216
KSR1	0.234252021	2.104149987	3.143014975	0.00177212	-2.808649338
ALDH3B2	0.338746857	1.843115249	3.140545857	0.00178683	-2.816255279
TMSB4XP1	-0.221193674	1.436424397	-3.139230435	0.001794712	-2.820304999
PRDX2	0.206187675	5.973654449	3.13685992	0.001808998	-2.827598857
IQGAP3	0.260694861	2.300610991	3.134252264	0.001824834	-2.835616259
GABRE	-0.302268569	1.531049816	-3.13420493	0.001825122	-2.835761731
INSM1	0.284926033	0.302962096	3.13303069	0.001832296	-2.839369863
UPK3B	-0.357083301	1.409699751	-3.132758959	0.00183396	-2.840204637

PPDPF	0.236115816	7.645095701	3.132262898	0.001837001	-2.841728384
SNTA1	0.236003843	3.453096487	3.127791248	0.001864622	-2.855453452
MT-RNR2	0.262495136	11.54830773	3.125919617	0.001876297	-2.861192552
CA2	-0.372415946	2.888773277	-3.125185782	0.001880893	-2.863441852
AL590431.1	0.243353173	2.114733	3.125141124	0.001881173	-2.863578721
ZP3	0.215796604	1.638203454	3.124909214	0.001882627	-2.86428944
LAGE3	0.20916941	4.537668764	3.122214218	0.001899609	-2.872544906
PKMYT1	0.214122852	1.639311115	3.117158054	0.001931849	-2.888014761
ZFAS1	0.225266678	5.331647792	3.116654213	0.001935089	-2.889554991
LINC00086	0.254849951	1.351902838	3.115610541	0.001941817	-2.892744717
PPM1H	0.253042208	2.161293015	3.115451878	0.001942841	-2.89322954
BEAN1	-0.212207994	1.084671767	-3.114283673	0.001950401	-2.896798477
HIST3H2A	0.430782486	2.687842743	3.113945419	0.001952595	-2.897831621
ATP6V0A4	0.321022411	1.142054816	3.108196829	0.00199023	-2.915373316
CDCA5	0.282821579	2.532704242	3.106326448	0.002002619	-2.921074021
TPPP3	-0.412500985	3.386670995	-3.104666296	0.002013674	-2.926131208
RP11-16E12.2	-0.215188556	1.507968833	-3.097582605	0.00206148	-2.947680459
RP11-66N24.6	0.404337333	1.857764896	3.096611512	0.002068115	-2.950630926
APOD	-0.506806573	3.661850027	-3.094046092	0.002085736	-2.95842114
SLC16A3	-0.27557287	4.237084812	-3.093542722	0.00208921	-2.959948958
NXN	-0.218417872	3.378976991	-3.093359729	0.002090474	-2.960504315
FGG	0.845149936	4.002112372	3.093237113	0.002091322	-2.960876417
SYBU	0.309088334	1.820622108	3.092404027	0.002097088	-2.963404215
AFAP1-AS1	-0.482486303	2.79325095	-3.088223459	0.002126245	-2.976079239
RP11-302F12.1	-0.399158306	2.57764416	-3.088162623	0.002126672	-2.976263566
FKBP1B	0.211663772	1.633079079	3.08603186	0.002141679	-2.982717337
IFI6	-0.39528805	7.478270446	-3.080230256	0.002183033	-3.00026782
MT1G	-0.362952052	2.158409901	-3.080215517	0.002183139	-3.000312368
DUSP4	0.425932024	3.148957523	3.079848747	0.002185778	-3.001420812
IFITM3	-0.215359902	8.300135576	-3.079043227	0.002191585	-3.003854794
RP11-323H21.3	0.214340166	0.222604644	3.078619261	0.002194646	-3.005135618
RP11-93B14.9	-0.226349791	1.87564513	-3.078485274	0.002195615	-3.005540362
JUN	-0.249733014	5.767456618	-3.077503861	0.00220272	-3.008504484

EREG	-0.461578802	1.298731561	-3.076980669	0.002206516	-3.010084286
FGFBP1	-0.454763899	2.062851842	-3.074088463	0.002227612	-3.018812776
KLF15	0.274851854	1.594777676	3.073973413	0.002228455	-3.019159826
C2	-0.319940457	3.632476716	-3.072041287	0.002242656	-3.024986246
SCD	0.291532676	6.046474418	3.071615698	0.002245795	-3.026269155
SPOCK1	-0.314323133	1.262247905	-3.066486558	0.002283947	-3.041717155
RP11-664D7.4	-0.338644146	1.631623594	-3.065945135	0.002288009	-3.043346368
GSPT2	-0.225720525	2.103566038	-3.065493073	0.002291405	-3.044706469
GGTLC1	-0.511478278	2.543333973	-3.065087673	0.002294455	-3.045926017
GIGYF1	0.200070601	3.498507899	3.063442062	0.002306873	-3.050874843
LILRP1	0.236508502	0.234793628	3.060951124	0.002325788	-3.058360944
TDRD5	0.217704161	0.695943363	3.059835618	0.002334305	-3.06171151
TTYH3	-0.222257583	4.625626872	-3.055203977	0.002369974	-3.075610666
WBSCR17	-0.200885955	0.894676461	-3.054928784	0.002372109	-3.076435858
FAM171A2	0.21537302	1.142410795	3.05445256	0.002375808	-3.077863694
CKB	0.372014374	4.885795486	3.053619644	0.00238229	-3.080360461
CENPU	0.240250722	2.503933739	3.053548325	0.002382846	-3.080574217
CAT	-0.202224377	4.737821447	-3.05336506	0.002384274	-3.081123476
TMC4	0.236723193	5.046774241	3.052092083	0.00239422	-3.084937805
ERVH48-1	0.309757742	0.456154222	3.051445804	0.002399284	-3.086873719
LPHN2	-0.228405618	2.340738528	-3.050998367	0.002402795	-3.088213777
SEMA4B	0.238264592	4.743855151	3.049455835	0.002414937	-3.092832149
C6	-0.209567829	0.578562424	-3.049201912	0.002416942	-3.093592184
FZD10	0.333413658	0.788362817	3.04821737	0.002424727	-3.096538509
SRPK3	0.212024628	1.011019392	3.047325679	0.002431798	-3.099206181
WNT7A	-0.207687619	0.512082097	-3.046900479	0.002435176	-3.100477988
HILPDA	0.305617341	3.406847551	3.045340029	0.002447612	-3.105143947
CTD-2589M5.4	0.337356198	1.439660699	3.04488419	0.002451256	-3.106506533
H19	0.592627756	2.384344019	3.044149856	0.002457136	-3.108701181
GMDS	0.201318409	2.932110584	3.038235154	0.002504972	-3.126359389
ITGB1	-0.207458355	5.895097941	-3.0358064	0.002524862	-3.133600797
FOSB	-0.502719808	2.852220584	-3.030328078	0.002570258	-3.149914104
SLC44A3	0.210355333	3.14462824	3.029595138	0.002576388	-3.152094493

MAGEA12	0.406279046	0.713682003	3.027829379	0.002591211	-3.157345283
XIST	-0.377729326	1.231252535	-3.027506208	0.002593932	-3.158305966
FAM46A	0.250686953	3.196190724	3.02528212	0.002612732	-3.164914798
SYT8	-0.330579414	1.209595004	-3.024389413	0.002620313	-3.167566141
CEBPD	0.278357685	5.382008451	3.02365852	0.002626535	-3.169736334
HOTAIRM1	-0.242082271	1.522090585	-3.023582052	0.002627187	-3.169963356
CACNG6	-0.230487627	0.550225562	-3.023233433	0.00263016	-3.170998287
RP1-27K12.4	0.208896631	0.62158163	3.023145924	0.002630907	-3.171258053
TST	0.249354422	4.601125842	3.018075389	0.002674511	-3.186297288
MIR3682	0.223290261	1.896992002	3.017930312	0.002675769	-3.186727228
MMP1	-0.581264169	4.040723501	-3.01588925	0.002693515	-3.192773882
ZSCAN31	0.244979249	2.193468232	3.014005396	0.00270999	-3.198351303
LOXL4	-0.316589327	2.110612261	-3.013776434	0.002711999	-3.19902895
EPHB3	0.24708409	2.772057769	3.013723566	0.002712463	-3.199185413
KIF11	0.24139816	2.505507142	3.010138013	0.002744101	-3.209790726
PLK1	0.254921169	2.490350283	3.009232377	0.002752146	-3.212467482
ACSL5	-0.335139347	5.117979631	-3.00389336	0.002800013	-3.228232036
HIF3A	0.276561229	1.14708105	3.001470617	0.002821984	-3.235376781
CDCA7	0.293869511	2.631601437	3.000936391	0.00282685	-3.236951484
FOXQ1	-0.445769651	3.043586727	-3.000523108	0.00283062	-3.238169505
C7orf57	-0.221539887	0.909187357	-3.000206913	0.002833507	-3.239101279
TM4SF5	0.372958808	0.60928673	2.998065999	0.002853127	-3.245407715
TMEM45B	0.300274635	4.055235208	2.993772035	0.002892854	-3.258043246
FADS1	0.21967492	2.821343202	2.992247777	0.002907077	-3.262524368
RN7SKP51	-0.200319205	0.72647079	-2.985101945	0.002974615	-3.28350284
HOXC9	0.224392532	0.773270167	2.984845977	0.002977061	-3.284253403
LINC00942	0.384054451	0.866071903	2.98136105	0.003010541	-3.294465926
EIF4EBP1	0.293190223	5.017262298	2.974939225	0.003073141	-3.313254831
MT1X	-0.29227149	3.747694828	-2.973963259	0.003082759	-3.31610688
HOXC11	0.230852064	0.615540349	2.973645148	0.003085899	-3.317036296
CFAP43	-0.205644869	0.698413198	-2.973566374	0.003086677	-3.317266433
ARHGAP11A	0.207319106	1.76369804	2.973497498	0.003087358	-3.317467647
NUSAP1	0.253327041	3.316251863	2.968774014	0.003134358	-3.331256157

CCDC78	-0.231435944	1.103922687	-2.967751074	0.003144622	-3.334239475
GRAMD2	0.257335672	2.117900112	2.96303503	0.003192342	-3.347980584
IGKV1D-13	-0.459939267	1.984364675	-2.962500542	0.003197792	-3.349536589
KIF12	0.33471347	1.956692936	2.961630513	0.003206682	-3.352068842
MSMB	0.777484803	3.228039716	2.959717615	0.003226306	-3.357633879
DYNLRB2	-0.204759654	0.689857351	-2.959074699	0.003232926	-3.359503483
AC137932.5	0.233279197	1.586005397	2.956296482	0.003261677	-3.36757805
SMOC1	0.434278112	1.493834399	2.95175719	0.003309155	-3.38075522
OLFML2A	-0.215762992	2.072067783	-2.949236765	0.003335789	-3.388063356
LRRC56	0.20457657	1.838136982	2.943705821	0.003394924	-3.404079555
MAGEA6	0.457460669	0.91304859	2.942757645	0.003405157	-3.406822316
CTD-2147F2.1	0.208788728	0.39087077	2.942207058	0.003411112	-3.408414592
EDARADD	0.210524929	0.7848362	2.942080819	0.003412479	-3.408779628
ARRDC3	-0.236813771	5.053508603	-2.94180251	0.003415494	-3.409584346
CHL1	0.29920908	1.581817232	2.941074013	0.003423397	-3.411690406
ANKS4B	0.238529694	0.468585283	2.940554858	0.003429039	-3.413190958
TLE2	0.227525301	3.498632894	2.938561252	0.003450785	-3.418950851
RIMKLB	0.261805808	2.138244783	2.937418154	0.003463311	-3.42225177
PBLD	0.20976098	1.60639221	2.936336891	0.003475197	-3.425372987
SMIM1	0.226397225	2.069613704	2.933232061	0.003509535	-3.434329339
TFPI2	-0.647436182	3.653781814	-2.932361405	0.00351922	-3.436839238
ZNF710	0.246203283	3.257756539	2.930052511	0.003545021	-3.443491749
RP11-909N17.2	0.206345546	0.566815829	2.926386235	0.003586344	-3.454044827
CCDC17	-0.253897588	1.047685708	-2.925982607	0.00359092	-3.455205857
MT1A	-0.336768833	1.46295402	-2.923318946	0.003621253	-3.462863962
CTD-3128G10.7	-0.23633506	1.002123725	-2.923066864	0.003624136	-3.463588359
MCM2	0.243087217	3.421380985	2.922789077	0.003627315	-3.464386548
MT-ND1	0.273349835	11.2296548	2.919804328	0.003661635	-3.472958284
TMEM238	0.21248983	2.37554896	2.917988416	0.003682659	-3.478169161
ZC3H12A	-0.225958768	3.202627759	-2.916674243	0.003697943	-3.48193831
MACROD2	-0.258562359	1.10778067	-2.90970306	0.003779991	-3.50190477
RP11-359M6.1	0.200854856	0.567288234	2.90866977	0.003792293	-3.504860332
HLA-K	-0.289869592	2.474231699	-2.908667547	0.003792319	-3.504866688

IL37	-0.520181976	1.663908802	-2.907967268	0.003800677	-3.506869146
PRSS8	0.262146376	6.19909617	2.907153263	0.003810413	-3.509196219
MAPK8IP2	0.228123111	1.205746184	2.906775951	0.003814934	-3.510274662
FOXD3-AS1	0.216252634	0.765273357	2.90614576	0.003822495	-3.512075591
ROPN1L	-0.244269377	0.873933619	-2.902652169	0.003864661	-3.522052556
LA16c-312E8.4	0.231510146	0.485645816	2.90203072	0.003872206	-3.523826068
CAPN12	0.202255284	1.454468181	2.901251061	0.003881691	-3.526050567
CALML3	0.247159122	0.325732528	2.8943534	0.003966529	-3.545705592
EPHX1	0.331418235	6.729470704	2.89250658	0.003989529	-3.550960467
RP11-12G12.7	0.215582056	2.677391172	2.888223181	0.004043341	-3.563135813
HOXB2	-0.319223658	2.020588845	-2.884153995	0.004095074	-3.574686109
IGFBP5	-0.335285902	5.551787449	-2.881931441	0.004123584	-3.58098813
USP9Y	0.2303606	0.703373621	2.876995901	0.004187541	-3.594965988
RP11-234A1.1	0.247968738	3.559757145	2.870673514	0.004270789	-3.612837654
TRIM17	0.225484686	1.015917532	2.86738538	0.004314676	-3.622117281
CIB2	0.221999679	1.808066019	2.866806039	0.004322451	-3.623751208
BIK	0.26587431	2.869094429	2.865610848	0.004338531	-3.627121014
LINC00958	0.218416521	0.51659949	2.865178336	0.004344364	-3.628340133
MTND2P28	0.349561186	6.633096583	2.86456763	0.004352611	-3.630061228
PPBP	-0.282334989	0.876879546	-2.86443058	0.004354464	-3.630447411
GOLGA2P10	0.218950483	2.187144125	2.863517045	0.004366833	-3.633021154
RET	0.293966728	0.77044254	2.860425206	0.004408931	-3.641726039
TSPAN12	0.226386203	3.584318857	2.860053803	0.004414013	-3.64277109
PIP	-0.360545317	1.253186368	-2.858024776	0.00444187	-3.64847803
CSF3R	-0.316758875	2.536688852	-2.857790875	0.004445091	-3.649135659
C2orf72	0.250177606	0.899532901	2.851088888	0.004538304	-3.667956647
RRAGD	-0.216884994	2.515193945	-2.847766947	0.004585161	-3.677269705
LSR	0.21374864	6.332065149	2.847273647	0.004592156	-3.678651773
HS3ST1	-0.225261589	1.823310397	-2.845812719	0.004612931	-3.682743471
NR1D1	-0.216458174	2.979257055	-2.845488282	0.004617556	-3.683651862
EDN2	-0.337814572	1.68396405	-2.845063306	0.004623621	-3.684841599
ST6GALNAC2	-0.205820708	1.444257486	-2.843979147	0.004639125	-3.687875971
RP11-76C10.5	0.311106529	1.672252354	2.839331714	0.004706125	-3.700870644

BIRC5	0.309292097	3.174583943	2.837519107	0.004732493	-3.705933291
VEGFA	0.247394796	4.248469568	2.835328817	0.004764535	-3.712046641
TDRP	0.210012223	2.8787784	2.828861351	0.004860301	-3.7300714
EIF1AY	0.31977468	1.041887203	2.825391121	0.004912402	-3.739726466
RP11-175B9.3	0.212855087	3.40243804	2.822745948	0.004952456	-3.747078303
DLC1	-0.269736315	2.690065513	-2.821288025	0.004974659	-3.751127518
DEPDC1B	0.200334791	1.44533337	2.819249539	0.005005854	-3.756785785
BRINP1	-0.314978666	0.868454017	-2.814599181	0.005077685	-3.769679052
ST14	0.203842634	6.091568444	2.81317136	0.005099927	-3.773633596
RNY3P8	0.254296286	1.202435678	2.811997431	0.005118279	-3.776883494
RP11-359M6.2	0.214493918	0.802296663	2.811752173	0.005122121	-3.7775623
HMGCS1	0.2060152	3.691270661	2.804514422	0.005236678	-3.797568538
ELFN2	-0.22686348	1.180862611	-2.8037007	0.005249702	-3.799814665
HIST1H2BK	0.271319534	6.004259455	2.799098811	0.005323908	-3.812505431
CTAG2	0.303895619	0.42231145	2.798513718	0.005333411	-3.814117512
RP11-475C16.1	0.223644989	3.562440472	2.797957005	0.005342466	-3.815651097
KRT5	-0.393505447	1.202212968	-2.795079121	0.005389502	-3.823574123
ID1	0.343772877	5.041873408	2.793100148	0.005422064	-3.829017796
SPRR2D	-0.345150585	0.770162043	-2.792400714	0.005433615	-3.830940877
LYPD6B	0.255379839	1.086446558	2.789479922	0.005482092	-3.838966492
LRRN1	-0.293961756	1.481169803	-2.782003626	0.005607966	-3.859472419
DRC1	-0.222624913	0.769628283	-2.780798614	0.005628497	-3.862772526
HIP1	-0.209142289	3.351391789	-2.779723655	0.00564687	-3.865715294
CENPM	0.202652852	2.382970597	2.776997744	0.005693703	-3.873172701
RP11-129M6.1	0.217338989	0.575867968	2.769649067	0.005821715	-3.893241496
DSP	0.360343611	4.776380637	2.766916004	0.005869984	-3.900692175
COX6CP1	0.319464614	1.391824041	2.766802611	0.005871994	-3.901001144
SLC5A8	-0.270009534	0.803888648	-2.766451928	0.005878216	-3.901956598
BAIAP2L2	0.283659779	1.488761945	2.766064075	0.005885104	-3.903013185
CSAG1	0.397682933	0.805359045	2.761134143	0.005973293	-3.916430762
SYTL5	0.213306694	0.880040648	2.758075061	0.006028614	-3.92474486
MAP1B	-0.264465166	2.049191492	-2.757158248	0.006045284	-3.927234869
IGFBPL1	0.251341659	1.386185136	2.756063992	0.006065235	-3.930205756

SLC28A2	0.249517575	0.542944723	2.756027725	0.006065897	-3.9303042
PKP1	0.413636531	1.593973475	2.754108055	0.006101044	-3.93551324
CFTR	-0.262653941	1.305099797	-2.750991976	0.006158488	-3.943961249
NTS	0.487817551	0.918158166	2.750255103	0.006172143	-3.945957631
FAM83E	0.275268437	3.716361262	2.748097601	0.006212281	-3.951799885
RACGAP1	0.209671441	2.996078619	2.747826346	0.006217345	-3.952534098
RAB27B	-0.279580918	2.666545809	-2.747352085	0.006226206	-3.953817621
WNT4	0.217488795	1.126851571	2.745711492	0.006256948	-3.958256001
RASGEF1A	0.205667625	1.455526219	2.744304009	0.00628343	-3.962061691
AC105009.1	0.229879334	1.222558595	2.743899789	0.006291055	-3.963154309
AOC1	0.470399245	2.348896541	2.743424734	0.006300026	-3.964438199
CDKN2B	-0.245503841	2.568327628	-2.741705085	0.006332597	-3.969083939
GPR116	-0.364695608	4.596699507	-2.739359731	0.006377264	-3.975415502
SH3BGRL2	0.246446616	3.632022417	2.736212472	0.006437651	-3.983903645
MT1H	-0.211981879	0.844468979	-2.733214921	0.006495644	-3.991979224
GATM	-0.246729316	1.766505607	-2.728889152	0.006580166	-4.003617956
MS4A8	-0.346609661	1.221838746	-2.72717653	0.006613902	-4.008220922
S100A16	-0.289010691	6.624796421	-2.72602748	0.006636624	-4.01130762
CALB2	-0.266881988	0.916369751	-2.723378545	0.006689274	-4.018418657
ENDOD1	-0.226203233	4.001584282	-2.72027731	0.006751392	-4.02673536
SUSD2	-0.473599607	3.92958629	-2.714667785	0.006865074	-4.041755277
MIA	-0.214244452	0.373508698	-2.711924651	0.006921292	-4.049089266
VILL	-0.263431757	1.661151954	-2.708900575	0.006983747	-4.057166033
MT-TM	0.248074176	0.97331278	2.707782801	0.007006961	-4.060149192
ALDOC	-0.256557844	3.187571367	-2.705963653	0.007044889	-4.065001653
TOX	-0.222511036	1.889750359	-2.704698152	0.007071382	-4.068375426
FAM81B	-0.223345762	1.024392451	-2.701701364	0.007134479	-4.076358631
AC072052.7	0.200976678	1.402803223	2.698816337	0.007195702	-4.084035996
SOX15	0.212572744	1.108871722	2.695222853	0.007272619	-4.093587499
FOS	-0.330101376	6.100449931	-2.694677143	0.007284364	-4.095036919
DDIT4L	0.295442916	1.722687092	2.694442432	0.007289421	-4.09566023
UGT1A6	0.298846589	0.659932764	2.690117409	0.007383172	-4.10713655
BBOX1-AS1	0.239303168	0.978490585	2.681547028	0.007572159	-4.129824899

DUSP23	0.218363477	5.814623688	2.68069252	0.007591238	-4.132083183
LGALS3BP	-0.21133464	8.259954888	-2.677209469	0.007669455	-4.141280907
CHRNA9	0.2684212	0.538569032	2.675361843	0.00771124	-4.146155234
BUB1	0.219051968	2.132620919	2.673446335	0.007754775	-4.151205193
MMP24	-0.242510769	2.135826785	-2.672270884	0.0077816	-4.154302361
CHMP4BP1	0.200601968	1.913468032	2.660427936	0.008056557	-4.185433202
MAD2L1	0.205066176	1.958805568	2.659134985	0.008087097	-4.188823769
GLCCI1	-0.204603185	2.352980865	-2.657692761	0.008121286	-4.19260389
CITED2	-0.238549205	5.329937595	-2.655391367	0.008176111	-4.1986318
UGT2B4	0.242743758	0.422300356	2.654416928	0.008199425	-4.201182565
CKS2	0.239990923	5.260702672	2.652129559	0.008254385	-4.207166574
LDHB	0.255118822	7.076099262	2.650600872	0.0082913	-4.211162992
RP11-108M9.4	0.217968043	2.79185733	2.649726342	0.008312485	-4.213448252
HES1	0.213264129	4.542448085	2.64944649	0.008319274	-4.214179389
SCARA3	-0.238230328	2.287192719	-2.648277324	0.008347693	-4.217233111
P3H3	-0.219749451	2.521697647	-2.646124647	0.008400246	-4.222852224
C1orf194	-0.266770351	1.067954611	-2.645321619	0.008419926	-4.224947223
C9orf152	0.300792605	2.780314046	2.644471558	0.008440803	-4.227164251
MFSD4	-0.36167958	2.643133775	-2.637025356	0.008625679	-4.24655494
RPS2P46	0.219103774	2.622824454	2.636631411	0.00863556	-4.247579332
AZGP1	-0.546979291	3.164166618	-2.63550991	0.008663746	-4.250494808
CITED4	0.244927709	2.350804248	2.634605498	0.008686536	-4.252845056
MT-TP	0.203784449	9.201085313	2.62870531	0.008836537	-4.268158318
HOXC10	0.349962195	1.116063869	2.623055381	0.008982348	-4.282790764
TRIP13	0.248098637	2.537828923	2.622516106	0.008996377	-4.284185802
CNFN	0.242158655	2.066811939	2.620758273	0.009042244	-4.288731163
RHOV	0.382721583	3.20666835	2.620721182	0.009043214	-4.28882704
NAPSA	-0.567995274	7.675956083	-2.616535537	0.009153283	-4.299638098
SLC1A7	-0.400995099	1.47725309	-2.610746598	0.009307491	-4.314562584
PRSS3P2	-0.480221373	1.619192659	-2.609592081	0.009338522	-4.317535202
LGR4	0.210670093	3.381162382	2.609336114	0.009345415	-4.318194086
CMAHP	-0.241022738	2.592038415	-2.602330097	0.009535842	-4.336203827
KCNJ8	-0.214845738	2.388694943	-2.597996978	0.009655348	-4.347319007

NEUROD1	0.207917521	0.235154206	2.597924007	0.009657372	-4.347506034
ATP10B	-0.220174571	0.885007271	-2.592295529	0.009814631	-4.361916719
RP11-632C17__A.1	0.262198445	3.074438965	2.591143864	0.00984709	-4.364861598
ATP11A	0.26387122	4.321651677	2.591133364	0.009847386	-4.364888439
ANXA2P2	-0.216081473	3.188416583	-2.590652668	0.009860963	-4.366117227
PHLDA3	-0.216850507	4.308719522	-2.589945827	0.009880957	-4.367923699
TTR	0.215897268	0.338870868	2.586227102	0.009986744	-4.377419731
SPINK13	-0.227751829	0.703614597	-2.584381444	0.010039623	-4.382127821
CCNL2	0.215478352	4.112332062	2.580513074	0.010151265	-4.391985032
PLEKHG4B	0.211400883	0.969313691	2.575536687	0.010296515	-4.404644503
RPS4Y1	0.693975675	2.773691078	2.571759332	0.010408003	-4.414237864
LHFPL3-AS2	-0.280005933	1.213504382	-2.571001675	0.010430495	-4.416160435
MISP	0.27858371	4.355421286	2.570956182	0.010431846	-4.416275857
TMEM125	0.241674807	5.215393111	2.567246437	0.010542612	-4.425681302
CYP4X1	-0.291062144	2.228029339	-2.567188473	0.010544351	-4.425828154
UBE2C	0.347699222	4.378032018	2.563510089	0.010655232	-4.435140824
MEG3	-0.249747606	1.045311167	-2.562661449	0.010680961	-4.437287506
LRFN4	0.219384694	2.56112533	2.558094493	0.010820372	-4.44882798
KIF20A	0.20511473	2.397055134	2.556596937	0.010866439	-4.452607865
KIF4A	0.23089644	2.184699531	2.555294713	0.010906639	-4.455892972
WBSCR27	0.224188634	2.145847627	2.553445424	0.010963955	-4.460555355
SEC11C	0.259453802	4.132422052	2.548609399	0.01111511	-4.472732282
TXLNGY	0.22352604	0.751698675	2.547831571	0.011139595	-4.474688724
ASMTL-AS1	0.237576682	1.698487158	2.545265821	0.011220701	-4.481138135
CAPN9	0.300682466	1.604962847	2.545106192	0.011225764	-4.481539179
NPC1L1	0.202679422	0.490528818	2.538605725	0.011433693	-4.497849778
BAI1	0.213107484	0.713629913	2.534582666	0.011564089	-4.507923846
RP11-357D18.1	-0.204150739	0.798460534	-2.533142426	0.011611091	-4.511526542
C1orf116	-0.36233939	4.816684665	-2.530853416	0.011686143	-4.51724829
AC007405.6	0.215614319	2.331324561	2.530549155	0.011696151	-4.518008458
CEMIP	-0.30797981	2.679737263	-2.529129352	0.011742955	-4.521554537
KDM5D	0.284494402	1.034737321	2.52832791	0.011769449	-4.523555348
HIST1H4H	0.282762998	2.60463214	2.527900068	0.011783614	-4.524623208

RP11-169K16.8	0.216050469	1.492750938	2.527171588	0.011807767	-4.526441035
CNTD2	0.233274152	1.24168551	2.525679629	0.011857372	-4.530162426
SLC16A1	-0.260084581	2.049511567	-2.525231813	0.011872297	-4.531278995
DUSP6	-0.26606164	5.032709197	-2.524356523	0.011901517	-4.533460857
AGTR2	-0.260674559	0.895661835	-2.523784528	0.011920647	-4.534886287
CST1	-0.480067647	2.867219243	-2.519429338	0.012067202	-4.545729243
MIR6772	0.241699986	1.721333814	2.514825878	0.012223851	-4.55717046
FTLP3	-0.227855123	2.913122028	-2.511062404	0.012353256	-4.566508862
CLDN8	0.266161436	0.922553684	2.510740699	0.012364374	-4.567306486
MUC13	0.52118939	2.15349254	2.509374879	0.012411675	-4.570691734
RP11-599J14.2	0.218734625	1.181163099	2.50487836	0.012568536	-4.581823878
IGSF9	0.215355192	2.223160621	2.504555405	0.012579869	-4.582622676
MYBL2	0.330704241	3.770620535	2.504121402	0.012595115	-4.583695983
AGPAT2	0.205283605	5.986583943	2.502579927	0.012649394	-4.587506646
MIR429	0.258892549	1.688648945	2.500333668	0.012728863	-4.593055505
UMODL1	0.200446743	0.628773262	2.490803966	0.013070959	-4.616542366
RP11-132A1.4	0.217138008	1.605002768	2.488598525	0.013151283	-4.621965423
EGFR	-0.269085661	3.472097575	-2.488047915	0.013171405	-4.623318611
FUT2	0.219965931	2.735488042	2.487839518	0.013179027	-4.623830696
IFITM10	-0.204045343	2.044133087	-2.486020204	0.013245742	-4.62829943
RP11-510N19.5	0.244147955	1.854952962	2.481934354	0.013396662	-4.638323778
RP11-359E19.2	0.323997789	0.872996456	2.478579098	0.013521735	-4.646543637
CMB9-22P13.1	0.213009212	1.794737708	2.477538355	0.013560739	-4.649091092
TGFA	-0.252364976	2.825057314	-2.47314866	0.013726356	-4.659824385
TC2N	0.214295044	4.047947347	2.46925012	0.013874941	-4.669341177
TPX2	0.284637688	3.647957366	2.468997505	0.013884618	-4.669957334
OBP2A	0.201047321	0.390085901	2.466301694	0.013988259	-4.676528902
PNMAL1	-0.26614492	1.735199248	-2.465976288	0.014000816	-4.67732167
CLDN10	0.373669614	1.828668313	2.457954361	0.014313531	-4.696832735
CDKL2	-0.220617569	1.652194881	-2.455701834	0.014402445	-4.702300212
GSTA1	0.471532143	2.935400112	2.452692883	0.014521978	-4.709596097
SFTA1P	-0.407226	3.570521755	-2.451720859	0.01456078	-4.711951122
IBSP	-0.211031027	0.781810374	-2.44710942	0.014746114	-4.723111336

MLPH	0.264145809	4.430310447	2.446639153	0.01476513	-4.724248286
ZNF385B	-0.264847657	1.32605422	-2.440877325	0.014999893	-4.738161136
ATOH8	0.289361169	2.032125046	2.440125655	0.015030762	-4.739973803
RP11-785D18.3	0.339464936	0.981510293	2.43856934	0.015094853	-4.743725152
TMEM27	-0.274414976	1.698723787	-2.434570057	0.015260656	-4.753354324
WSB1	0.229399548	4.773007725	2.429908824	0.01545593	-4.764557815
LINC00342	-0.318352669	2.422176301	-2.42414417	0.01570048	-4.778384434
UPK1B	0.330111461	0.590380364	2.423616766	0.015723023	-4.779647819
PPP1R3C	-0.202569542	2.064572823	-2.418394109	0.015947804	-4.792144098
GPR35	0.211696584	1.218918719	2.417834703	0.015972048	-4.793481031
RPL3P4	0.210370356	4.579481405	2.415592485	0.01606955	-4.798836711
PTK6	0.253606479	3.135291347	2.414310812	0.016125518	-4.801895886
NCAPH	0.205511437	2.190257952	2.41411493	0.016134087	-4.80236329
RHOU	0.203053443	3.724981382	2.408509216	0.016381017	-4.815723655
BHMT2	0.350274597	1.717316467	2.407506465	0.016425537	-4.818110363
NES	-0.207529688	3.079555386	-2.406985535	0.016448707	-4.819349874
SLC22A17	-0.201495514	2.125506814	-2.405859369	0.016498897	-4.822028604
GLB1L3	-0.284953217	1.066818213	-2.393607473	0.017053698	-4.851092146
AKAP12	-0.264787842	2.192646097	-2.393555864	0.017056069	-4.851214263
OXCT1	-0.206840711	2.671208274	-2.388613359	0.017284515	-4.862897422
CYP2T1P	0.215021335	1.553035038	2.386174848	0.017398213	-4.86865291
LINC00261	0.262963492	1.459490689	2.385957313	0.017408388	-4.869166069
TRIM29	-0.312068168	1.325898651	-2.383148537	0.017540233	-4.875787764
KCNK5	-0.26367995	3.814757202	-2.381855872	0.017601205	-4.878832665
RP11-806O11.1	0.246813423	1.877760247	2.381269747	0.017628913	-4.880212762
GSTM3	0.20466569	2.427288546	2.378816175	0.017745316	-4.885986371
HIST1H1C	0.282880572	5.918447003	2.377762384	0.017795518	-4.888464308
MT-ATP6	0.209871803	11.66005295	2.375961755	0.017881587	-4.892695913
SORCS2	-0.243298723	1.802061534	-2.372100503	0.018067386	-4.901759563
MT-CYB	0.21548228	11.32072833	2.371201369	0.018110895	-4.903868062
RAB38	-0.205026182	3.170254854	-2.368737971	0.018230568	-4.90964081
ADRA2A	-0.270867854	1.731857444	-2.363961935	0.018464569	-4.920816301
DDIT4	-0.253312791	5.569625271	-2.359371804	0.018691943	-4.931536012

CDK1	0.221034738	3.012852384	2.359362671	0.018692398	-4.93155732
C1orf233	-0.228855847	2.652459357	-2.348140323	0.019258688	-4.957679892
MACC1	-0.221309807	2.740617711	-2.347960342	0.019267892	-4.958097846
AQP4	-0.386738739	2.915764267	-2.345360186	0.01940128	-4.964132469
STMN1	0.208198633	4.412409697	2.341783395	0.019586092	-4.972423048
EPHA2	-0.24384536	3.900385876	-2.33705841	0.019832594	-4.983356033
SPRR2C	-0.224001498	0.473071237	-2.332331549	0.020081908	-4.994271743
NPTX2	-0.231583069	1.069103254	-2.331907454	0.02010441	-4.995250047
LRRC46	-0.203394802	1.277711235	-2.331219374	0.020140966	-4.996836941
PLEKHS1	-0.301337658	1.903348596	-2.327227633	0.020354186	-5.006033908
MBIP	0.214955324	4.415096735	2.322364814	0.020616602	-5.017217004
TFF3	0.549609688	4.467681632	2.322271184	0.020621684	-5.0174321
RP11-497G19.1	0.254277617	0.679971077	2.322148746	0.02062833	-5.017713367
MELK	0.22087305	2.3258451	2.321367938	0.020670761	-5.019506707
XKRX	-0.25933668	1.985689189	-2.321116486	0.020684442	-5.020084109
TF	0.294195852	0.842224155	2.31918267	0.02078992	-5.024522636
C2orf54	-0.268484831	2.422118651	-2.3118164	0.021196024	-5.041396663
MT-CO3	0.20338319	12.30343754	2.310224463	0.021284693	-5.045036432
ITIH2	0.215894757	0.654786064	2.308781922	0.021365321	-5.04833251
PTP4A3	0.212745198	4.625007181	2.305547417	0.021547076	-5.055715737
RP11-567G11.1	0.256760546	1.635820595	2.305395056	0.021555671	-5.056063272
DDX3Y	0.322805536	1.378941421	2.301032069	0.021803064	-5.066005728
CXCL2	-0.289529428	4.027162199	-2.297023045	0.022032567	-5.075125316
RASSF10	0.201297158	1.455920528	2.291810882	0.022334096	-5.086958484
RRM2	0.234713679	3.231348526	2.290939513	0.022384855	-5.088934182
FAM83D	0.206214603	2.563150775	2.285945015	0.02267774	-5.100244276
ABO	0.236286809	2.445462836	2.280077885	0.02302606	-5.11349958
RP11-497G19.2	0.250429784	0.661729369	2.277696999	0.023168732	-5.118869079
RP11-750B16.1	0.33659182	1.788455357	2.277568092	0.023176479	-5.119159639
ANKRD65	0.267804701	3.339729136	2.276069195	0.023266719	-5.122537027
TOP2A	0.253729536	3.933863051	2.271937611	0.023517046	-5.131835234
PLOD2	-0.256227782	3.587002984	-2.260412828	0.024227745	-5.157684518
PVRL4	0.203594457	4.173299221	2.252889743	0.024701681	-5.174488806

AADAC	-0.251163736	1.09433067	-2.24506934	0.025202866	-5.191899028
HK2	-0.209040594	3.590765694	-2.241105089	0.025460278	-5.200701809
GJB3	-0.302918461	1.78566962	-2.233626408	0.02595211	-5.217267016
RSPH1	-0.214832013	1.464567248	-2.229007934	0.02625994	-5.227469787
SLPI	-0.412347352	8.052018699	-2.227343432	0.026371656	-5.231141799
C5	0.26874627	2.12275263	2.2258468	0.026472456	-5.234441183
HIST1H2BD	0.219072028	4.338595426	2.22391401	0.026603125	-5.238698877
ENPP3	-0.247377534	1.329016397	-2.221958587	0.026735894	-5.243002741
AC016739.2	0.203197648	3.058824695	2.221013963	0.026800236	-5.245080518
BPIFA1	0.705477755	4.144556001	2.22071639	0.026820533	-5.245734874
CEACAM6	-0.418455381	8.296539745	-2.21860922	0.026964641	-5.250366035
EEF1A1P5	-0.252588354	4.202593928	-2.206103836	0.027833743	-5.277761805
KLK14	0.312202634	0.879999742	2.201150558	0.028184626	-5.288571058
IGFBP3	-0.272391722	5.732983369	-2.200877376	0.028204089	-5.289166514
IRX2	-0.334271194	2.563682115	-2.199790819	0.028281615	-5.291534169
SLC6A8	0.218697162	2.803149666	2.196344748	0.028528711	-5.299035726
MDK	0.250957712	6.81103843	2.194470884	0.028663856	-5.303109993
LRRC31	-0.232223206	1.142716384	-2.187036296	0.029205508	-5.319241108
BRDT	0.219838927	0.694449363	2.179494055	0.029764007	-5.335550929
KCNE4	0.251371552	1.950761827	2.177532117	0.029910788	-5.33978449
PSAT1	0.247709688	3.357731055	2.1765601	0.02998374	-5.341880567
NMU	0.281048443	1.944761205	2.176292972	0.030003816	-5.342456447
RP11-156K13.3	0.201844702	0.661348447	2.174270821	0.030156162	-5.34681359
ALOX15	-0.22833834	0.963526214	-2.172589336	0.03028335	-5.350433669
KRT23	-0.244286468	0.787114494	-2.163668955	0.030965849	-5.369592459
RSPO3	-0.227290721	1.105320148	-2.158869416	0.031338513	-5.379868685
RP5-857K21.6	0.267735187	5.5435284	2.156717494	0.031506849	-5.384468863
ALDH1A1	-0.309470329	4.523612455	-2.154940808	0.031646418	-5.388263504
EPHX3	0.230970426	2.993632266	2.1512184	0.03194056	-5.396203861
HNRNPA1P27	-0.226348592	1.461248332	-2.150812412	0.031972783	-5.397069069
MT-TL1	0.237239255	2.145615161	2.147936777	0.032201818	-5.403192794
MIR647	0.202954011	2.636921525	2.135507649	0.03320805	-5.429568377
RP11-470M17.2	0.266615742	1.087282312	2.131178841	0.033564781	-5.438719164

FAM183A	-0.237890566	1.63922378	-2.124870118	0.034090558	-5.452022702
VIL1	0.304036923	1.223893988	2.122602782	0.034281238	-5.456794493
GKN2	0.330409626	1.457308479	2.11973513	0.034523712	-5.462822532
ATF3	-0.21922075	3.397544574	-2.109316114	0.0354171	-5.48465679
GPC4	-0.215638614	4.370403377	-2.104261394	0.035857603	-5.495211475
C8orf4	0.292899218	6.781452413	2.103495804	0.035924728	-5.496807923
OAS1	-0.205133929	3.993428599	-2.102623066	0.03600138	-5.498627106
CTD-3252C9.4	-0.205599874	3.137847947	-2.102220914	0.036036747	-5.499465122
NUPR1L	0.23465422	1.029331225	2.101146074	0.03613142	-5.501704139
SERPINF2	0.222526796	2.803533709	2.096283308	0.036562401	-5.511819779
HPGD	-0.33632965	3.409476221	-2.094393701	0.036731055	-5.515744373
BPIFB1	-0.478737852	3.668325474	-2.089983071	0.037127309	-5.524891438
S100A6	-0.221012964	10.26809962	-2.086159553	0.037473765	-5.532805579
LINC00460	-0.214241747	0.926494596	-2.080430285	0.037998068	-5.544637699
RP5-857K21.11	0.294538252	2.298011347	2.076960633	0.038318619	-5.551787699
NR4A2	0.230116007	2.744528221	2.076157483	0.038393148	-5.5534411
BAALC	0.225915791	1.130264623	2.076034207	0.038404598	-5.553694825
MUC20	0.250590509	3.24677043	2.068257114	0.039132874	-5.56967165
MTND1P23	0.374321265	1.820646819	2.066608697	0.03928874	-5.573050496
SCG2	0.232886191	1.039585184	2.066347997	0.039313439	-5.573584624
MAOA	0.239790932	4.193080384	2.052565038	0.040638204	-5.601729121
C4BPA	-0.413902632	5.206922308	-2.049255885	0.040961858	-5.60845877
KCNK1	0.204668891	3.367683898	2.047804865	0.041104465	-5.611406266
TNS4	-0.308697007	1.968784132	-2.044495275	0.041431314	-5.618121445
GOLT1A	0.221226584	3.543160834	2.039202502	0.041958606	-5.628838317
GDF15	0.294137893	4.708279192	2.036307453	0.042249427	-5.634688689
CYP24A1	0.383978643	2.944675973	2.033758096	0.042506937	-5.639833714
TREM1	-0.262132384	3.307279565	-2.028523676	0.043039838	-5.65037777
CACNG4	-0.27523664	1.852887315	-2.02077935	0.043838649	-5.66592868
FOXA2	0.279653108	3.009381692	2.016398248	0.044296086	-5.674700192
F5	-0.214804404	1.273986799	-2.016056227	0.044331966	-5.675384173
ALPK3	0.207008789	2.17753467	2.004150105	0.04559642	-5.699123127
SST	0.32615919	0.644798877	2.00172633	0.04585753	-5.703938811

NFE4	-0.203813156	0.577390932	-1.999684047	0.046078522	-5.707992073
PEBP4	-0.341890245	2.760893514	-1.997995846	0.046261878	-5.711339525
KRT6A	-0.419127267	2.001009288	-1.995610975	0.04652195	-5.716063629
CDH17	0.261831233	0.959935347	1.995430351	0.046541697	-5.716421194
ITGB4	-0.256418291	4.654369032	-1.986212807	0.047558873	-5.734626062
BMP6	0.243510869	1.732194354	1.984432101	0.047757523	-5.738133438
LY6D	-0.304085323	1.403478395	-1.979764079	0.048281596	-5.747313132
MS4A15	-0.323220684	1.770668483	-1.977475071	0.048540345	-5.751806702
PAX7	0.216753545	0.573088702	1.976505385	0.04865031	-5.753708759
EPS8L3	0.223542559	0.602288436	1.972617346	0.049093334	-5.761325994
SEZ6L2	-0.233840289	4.278856233	-1.96998663	0.049395013	-5.766471574
RP11-469H8.6	0.202181036	1.031170796	1.967399415	0.049693225	-5.771525474

Table S2. The Kaplan-Meier analysis and univariate Cox regression recognized potential prognostic genes (p-value < 0.05).

ID	KM_pvalue	HR	HR_95L	HR_95H	Cox_pvalue
GJB3	4.91E-05	1.286595672	1.172188403	1.412169256	1.14E-07
KRT6A	0.00429395	1.155784859	1.094348355	1.220670397	2.05E-07
ARNTL2	0.001496175	1.368158001	1.201317673	1.558169297	2.31E-06
GNG7	1.09E-05	0.575059938	0.455897653	0.725368798	3.01E-06
VEGFC	0.011221481	1.373019086	1.201386191	1.569171866	3.27E-06
CERS4	0.000678228	0.708313369	0.612468808	0.819156537	3.33E-06
PLK1	8.54E-05	1.404974984	1.215060666	1.624572961	4.46E-06
FKBP4	0.000656198	1.512177196	1.263554203	1.809720443	6.41E-06
ECT2	0.002446829	1.415865748	1.214438723	1.650701495	8.94E-06
EXO1	0.003641936	1.419279618	1.213129347	1.660461549	1.23E-05
CMAHP	0.000300256	0.709203538	0.607583927	0.82781923	1.33E-05
ABCC2	0.01641664	1.190954913	1.100806709	1.288485611	1.35E-05
RRM2	0.000569177	1.322748251	1.164307198	1.502750252	1.73E-05
PRC1	6.63E-05	1.383039781	1.188043079	1.610041814	2.89E-05
FSCN1	0.002628796	1.272965405	1.136732038	1.425525866	2.93E-05
HMGA1	0.000804859	1.31631808	1.154320989	1.501049799	4.10E-05
KIF14	3.57E-05	1.483223959	1.227639286	1.792019316	4.40E-05
HJURP	0.000696022	1.328728707	1.158829645	1.523537118	4.67E-05
CCR2	0.000290542	0.65747613	0.536857988	0.805194059	5.01E-05
TPX2	0.002890157	1.25449589	1.123257827	1.401067413	5.78E-05
FOXM1	0.001110135	1.284586442	1.136168491	1.452392264	6.39E-05
OIP5	0.00029949	1.391319232	1.177332901	1.644198684	0.000106219
KIF4A	0.004864936	1.318787916	1.146095595	1.517501311	0.000111456
UCK2	0.000577045	1.348553105	1.158686123	1.569532456	0.000112307
SLC16A3	0.000238722	1.361666929	1.163643214	1.593389453	0.000118064
PCDH7	0.004697663	1.295245062	1.13477863	1.478402682	0.000126275
FAAH	0.002680337	0.68516452	0.564724029	0.831291738	0.00012645
LAMC2	0.003018706	1.208478654	1.096782472	1.33154996	0.000129739
CTSH	0.014136377	0.797222352	0.709852616	0.895345688	0.000129958

NEK2	0.000473885	1.300506898	1.1367318	1.487877959	0.000130159
SPC25	0.001912394	1.393815562	1.173296875	1.655780272	0.000157647
CHEK1	0.009400996	1.423292762	1.183868351	1.711138139	0.000172602
DPYSL2	0.009173965	0.742423181	0.635405919	0.86746466	0.000176654
FCGRT	0.001430341	0.688742608	0.56535989	0.839052059	0.000213682
DENND1C	0.000574151	0.614974746	0.474843797	0.796459679	0.000228842
BTG2	0.002973992	0.790595595	0.696935382	0.896842678	0.000259922
CD40LG	0.004450403	0.603035786	0.459661858	0.791129724	0.000260791
SLC34A2	0.007399669	0.881398002	0.823566105	0.94329093	0.000266352
FUCA1	0.018101883	0.697457644	0.574214114	0.847152923	0.000281248
MTL5	0.000132248	1.413577904	1.172183018	1.70468473	0.000291438
KIF11	0.001155491	1.324318256	1.13730774	1.542079404	0.000298648
TGIF1	4.80E-05	1.592238359	1.237244662	2.049087841	0.000301447
LRFN4	0.003396672	1.332934729	1.139913358	1.558640382	0.000317361
CDK1	3.48E-05	1.273785278	1.116573093	1.453132756	0.00031753
RPS6KA1	0.004880836	0.590566723	0.443361394	0.786647325	0.000317583
TPI1	0.015972759	1.536454559	1.214783084	1.943303824	0.000339279
CCNB2	0.002231108	1.284570323	1.119763106	1.473633938	0.00035072
NEIL3	0.000554053	1.297642464	1.124423134	1.497546532	0.000365046
PAICS	0.012166354	1.461870099	1.186330761	1.801406703	0.000366017
KIF23	0.000396967	1.331130474	1.13718014	1.558159763	0.000370964
NPC2	0.000685934	0.801342934	0.709049175	0.905650159	0.000389156
MBIP	0.008232649	0.774035589	0.671596865	0.8920993	0.000405683
NCAPG	0.006722682	1.300531936	1.124044937	1.504729268	0.000413278
PBK	0.00264619	1.257299261	1.106847348	1.428201852	0.00042976
IL16	0.000807067	0.656299107	0.518522636	0.830684116	0.000460091
GPR116	0.003731309	0.841316618	0.763093469	0.927558262	0.000519904
RAD51	0.018434057	1.400749598	1.157339712	1.695353071	0.000539485
FANCI	0.005103834	1.383204154	1.150918743	1.662370819	0.000543197
ALDOA	0.00197006	1.446680773	1.172732992	1.784622138	0.000565712
MS4A1	0.000646014	0.79028853	0.691254095	0.903511408	0.000570454
KIAA0101	0.000806402	1.304912968	1.121265709	1.518639016	0.00058392

BUB1B	0.000607945	1.301070344	1.119384097	1.512245926	0.00060453
CHRDL1	0.000134592	0.806123407	0.712575441	0.911952491	0.000616066
UNC13B	0.000216597	0.771897618	0.665524772	0.895272359	0.000620998
STRAP	0.009623053	1.481561896	1.182124991	1.856847346	0.000644008
CBFA2T3	0.009016829	0.613714545	0.46329009	0.812979926	0.000665978
BTK	0.000297861	0.722614395	0.599281982	0.871328656	0.00066777
B4GALT4	0.010819408	1.452028985	1.171202148	1.800191519	0.000671274
CD1D	0.000958449	0.637923665	0.491145459	0.828566355	0.000752791
RNASE1	0.001974356	0.850188854	0.773462434	0.93452643	0.000770455
CKS1B	0.002766141	1.307176886	1.118166873	1.52813632	0.000774864
CENPF	0.004575644	1.263007639	1.101983238	1.447561307	0.000792097
VDAC2	0.007902772	1.592458648	1.213269074	2.090158398	0.000798918
BIRC5	0.000512744	1.225187869	1.087336185	1.380516288	0.000853495
SFTPD	0.006936968	0.903031336	0.850333872	0.958994602	0.000884913
MAD2L1	0.000517454	1.305173287	1.115466397	1.527143547	0.000888771
SLAMF1	0.000487719	0.629991215	0.479697178	0.827373914	0.00089156
CDC20	0.038200527	1.211368754	1.081783089	1.356477349	0.00089451
CAMK1D	0.025627835	0.785676028	0.681362642	0.905959299	0.00090401
CBX7	0.008603131	0.678481609	0.539228569	0.853696039	0.000934413
MS4A2	0.022220796	0.658943112	0.51442748	0.844056824	0.000959723
NUSAP1	0.000263608	1.273267628	1.103049945	1.469752535	0.000968624
NLRP1	0.012702655	0.696946124	0.562001947	0.8642922	0.001008142
CDT1	0.035667821	1.283511767	1.104826966	1.491095445	0.001101184
CENPM	0.005548155	1.313851391	1.114825436	1.548408764	0.001126297
GDF10	0.005455362	0.707451543	0.574417114	0.871296613	0.001129017
TRAF3IP3	0.001737377	0.644054759	0.493829543	0.839979175	0.001167106
MCM4	0.007709112	1.283214988	1.103847104	1.491728972	0.001170049
MELK	0.005327625	1.240160619	1.088455639	1.413009687	0.001224346
CCDC28A	0.01171666	0.64258189	0.491460809	0.840171746	0.001224826
SKA1	0.037868553	1.310862579	1.112472032	1.544632722	0.001225086
HTATIP2	0.021106499	1.408803894	1.144263827	1.734502451	0.001238191
CDC6	0.001765597	1.265332592	1.096653265	1.459956962	0.001264623

MGP	0.002194157	0.830807679	0.7420004	0.930243971	0.001310817
ADRB2	0.006821284	0.696115979	0.557993829	0.868427986	0.00132683
RNF130	0.016814914	0.620321529	0.463190729	0.830756697	0.001354817
ICAM3	0.002295168	0.655897123	0.506335788	0.849635846	0.001403203
TFEB	0.009860164	0.664306628	0.516552394	0.854324365	0.001439479
PTGDS	0.016458927	0.844159954	0.760570386	0.936936332	0.00145074
MAP3K3	0.031267071	0.652159901	0.50070813	0.849422072	0.001522556
HLF	0.00133174	0.791660362	0.685024281	0.914896225	0.00155135
CYCS	0.000678607	1.410693187	1.139902451	1.745811904	0.001555754
PLOD2	0.037826572	1.200844484	1.070346461	1.347252994	0.001819781
RASGRP2	0.004290593	0.655902627	0.501605446	0.85766265	0.002055809
KLRB1	0.016591323	0.754918691	0.631276391	0.902777671	0.002065098
RACGAP1	0.005580173	1.2839374	1.09504127	1.505418371	0.002082826
ZWINT	0.010076308	1.248155849	1.083554449	1.437761641	0.002125476
CDCA3	0.000383509	1.319608841	1.105576582	1.575076319	0.0021295
DDIT4	0.008468696	1.213711587	1.07204438	1.374099657	0.002224228
SMAD9	0.002726069	0.684471749	0.536410755	0.873400786	0.002300667
UBE2C	0.036479194	1.160711037	1.05461661	1.277478562	0.002309194
ARHGEF6	0.00245767	0.734230157	0.601403281	0.896393386	0.002411675
FAM64A	0.003535517	1.26617469	1.086269015	1.475875979	0.002542333
TTLL12	0.013201781	1.375299408	1.117939474	1.691905963	0.002572896
CD1E	0.006654779	0.77776713	0.66018187	0.916295548	0.002653727
ORC1	0.009018638	1.307014356	1.09724771	1.5568832	0.00270233
HMHA1	0.000694399	0.733156122	0.597729124	0.899266704	0.002892284
DRAM1	0.001556136	0.818104642	0.716463613	0.934164965	0.003015942
ABCA8	0.00579955	0.654925263	0.494989523	0.866537736	0.003049058
KIFC1	0.019722175	1.216917773	1.068563591	1.38586873	0.00307908
FCHSD2	0.017554359	0.626772888	0.459723916	0.854522115	0.003136116
CENPA	0.020733675	1.227965784	1.070859705	1.408120934	0.003280693
ATIC	0.04499318	1.560894876	1.159870722	2.100572734	0.003294379
SYNE1	0.045716589	0.717043718	0.574109582	0.895563686	0.003363569
SASH3	0.003178058	0.803210553	0.692838209	0.931165725	0.003665778

NDRG2	0.010944236	0.767929429	0.642204634	0.918267445	0.003795399
DTYMK	0.00100326	1.361395764	1.104525531	1.678004152	0.003829857
ACAP1	0.006225947	0.733745668	0.594380183	0.905788452	0.003968495
SFTPC	0.027058298	0.941646911	0.903875969	0.980996216	0.003995106
MFAP4	0.011584424	0.865995943	0.784999594	0.955349506	0.004083028
TLE2	0.00145303	0.787367271	0.668779984	0.926982316	0.004100582
CD22	0.008611604	0.747988818	0.612582124	0.913326149	0.004375362
CDC45	0.009352153	1.225039592	1.06487204	1.409297968	0.004523127
RND3	0.031056314	1.2203881	1.063527699	1.400383944	0.004548109
PRKCB	0.000859776	0.713159816	0.5642391	0.901385463	0.004673366
MRPL15	0.010193791	1.322321925	1.089414211	1.605023374	0.004708933
MCM10	0.01959606	1.278414254	1.077065821	1.517403091	0.004969475
ARHGAP25	0.002241871	0.722668555	0.575922361	0.906805979	0.005036302
PIK3IP1	0.007705053	0.755408741	0.620750152	0.919278657	0.005107216
TTK	0.000391439	1.254481101	1.070391202	1.470231472	0.005108957
ESPL1	0.000353859	1.279716478	1.076166847	1.521766137	0.005263057
POLD2	0.003499067	1.416466661	1.108337866	1.810258281	0.005405488
PHACTR1	0.008377535	0.697098967	0.540584221	0.898929252	0.005414876
CORO1A	0.014010648	0.80447836	0.690094065	0.937822053	0.005429974
GPR87	0.033693036	1.123176567	1.034826077	1.219070168	0.005453762
MYBL2	0.018666811	1.142482881	1.039451662	1.25572663	0.005738024
CD74	0.001898211	0.862733636	0.776662786	0.958342979	0.005897007
RFTN1	0.003769824	0.804969971	0.689369592	0.939955377	0.006091113
TOP2A	0.036115354	1.168331155	1.045268179	1.305882753	0.006151619
RASSF2	0.001849044	0.788485363	0.664620078	0.93543543	0.006422079
RORA	0.011606109	0.620457045	0.43999247	0.874939849	0.006492207
CR2	0.035123918	0.823195681	0.714769129	0.948069946	0.006933875
ZNF671	0.009481218	0.684985843	0.520255825	0.901874776	0.007021035
CYLD	0.01487111	0.666656154	0.49643933	0.895236135	0.007024076
GSTM5	0.038373843	0.547116354	0.350408888	0.854248609	0.007979612
CEACAM21	0.006989718	0.725195612	0.571789379	0.919759435	0.008055579
IL33	0.03771703	0.840990499	0.739801353	0.956020175	0.008106942

BANK1	0.021815051	0.744175472	0.597738873	0.92648673	0.008219128
MAP4K1	0.008817633	0.777056314	0.643487362	0.938350232	0.008762023
PPM1G	0.001115905	1.50853687	1.109054012	2.05191403	0.008807902
PIK3CD	0.000226691	0.768414865	0.630254624	0.936861679	0.009190376
HSD17B6	0.001290348	0.864959715	0.775368309	0.964903131	0.009312407
CD2	0.005080979	0.836896621	0.730983758	0.958155289	0.009904834
GPR18	0.037110876	0.630993691	0.444531878	0.895668136	0.009980401
FXYD1	0.042849616	0.504099876	0.299234491	0.849222576	0.010048788
CD37	0.005834908	0.822509537	0.708779689	0.954488325	0.010069267
S100P	0.038375595	1.069965238	1.016166739	1.12661197	0.010191582
CD28	0.012535004	0.695249235	0.526337095	0.918368671	0.010478446
ANXA2P2	0.00653376	1.219723814	1.047172123	1.420708353	0.010703135
TXNDC15	0.005957687	0.683361799	0.508886188	0.917657739	0.011365009
NMB	0.025933109	1.190930438	1.0399165	1.363874223	0.011546041
TBRG4	0.008213706	1.399149612	1.077564644	1.816707374	0.011715504
CD48	0.022706016	0.835903142	0.727110721	0.960973401	0.01175097
CCR7	0.037353112	0.815682235	0.69600317	0.955940341	0.011850655
SLC3A2	0.029349342	1.344914499	1.067044187	1.695145365	0.01208975
KIF18B	0.000712551	1.197819213	1.039986835	1.379604837	0.012285439
ADRBK2	0.013189868	0.763333995	0.616600617	0.944985735	0.013157032
FHL1	0.028304554	0.836997518	0.727103824	0.963500427	0.013222234
STEAP1	4.88E-05	1.140063667	1.027349666	1.265143901	0.013588133
SELP	0.006373339	0.791596917	0.657289781	0.953347667	0.013755197
PSTPIP1	0.001047455	0.745607296	0.589258229	0.943440775	0.014490901
LYL1	0.0417565	0.71135494	0.540895565	0.935533369	0.014820564
SLC6A8	0.024162805	1.173805677	1.031842508	1.335300451	0.014827249
CYFIP2	0.019350481	0.808511809	0.681252394	0.959543556	0.014991586
TDG	0.039754396	1.356670401	1.060062357	1.736270103	0.015378056
LTB	0.015423105	0.863861593	0.766179603	0.973997283	0.016834565
PIK3R1	0.001224051	0.740850366	0.578366037	0.948982528	0.017573195
PPP1R16B	0.016315428	0.797299169	0.661318333	0.9612405	0.017582256
PIGR	0.033746966	0.936841376	0.887638642	0.988771469	0.017778395

E2F8	0.020649681	1.263560925	1.039861756	1.535383144	0.018615154
HIP1	0.032059997	0.813950046	0.685567963	0.966373451	0.018746922
MYO1F	0.016429967	0.787456742	0.645201516	0.961076664	0.018748186
TRIP13	0.003552683	1.175426574	1.026963555	1.345352154	0.018967331
IL24	0.037623566	0.732022308	0.56375759	0.950508993	0.019239729
A2M	0.03635591	0.865952386	0.767586037	0.976924407	0.019312523
IL7R	0.012523275	0.855911177	0.751178096	0.975246677	0.019473474
IL6ST	0.004395105	0.792793347	0.652347799	0.96347576	0.019597084
CCT2	0.014569142	1.180702395	1.026677411	1.357834632	0.019852626
GPSM3	0.006949632	0.823132687	0.698572051	0.96990342	0.020072179
IFT57	0.019130454	0.849668442	0.740289112	0.975208806	0.020503416
MAN1C1	0.031762223	0.739401507	0.572368446	0.955179471	0.020834899
PLCB2	0.02674174	0.808637791	0.67524432	0.968382936	0.020929398
CCDC90B	0.004602258	1.329754012	1.042509858	1.696142938	0.021718952
CBX3	0.016374991	1.345818677	1.042315564	1.737696311	0.022737391
CACNA1D	0.041320529	0.766943202	0.608204376	0.967112205	0.024922931
ELMO1	0.003851411	0.77020752	0.613011565	0.967713592	0.02497775
PSMB6	0.009610775	1.361219884	1.038741282	1.783812395	0.025384425
BIN2	0.02037405	0.806824291	0.66791385	0.974624852	0.02597534
CEBPA	0.042085772	0.861070567	0.753582571	0.983890219	0.027899723
DSP	0.043365352	1.12255188	1.012121033	1.245031652	0.028670042
CD81	0.017532619	0.784995783	0.631821924	0.975303888	0.028834014
PTK2B	0.048794168	0.790313446	0.639468535	0.976741323	0.029424904
FBLN5	0.006211839	0.842754242	0.720859452	0.98526101	0.031853444
TUBG1	0.001926231	1.252297476	1.019588004	1.538120262	0.031960361
PTPN7	0.045858515	0.806825682	0.662661847	0.98235274	0.032577933
RALGPS2	0.019528207	1.284763307	1.018645615	1.620403337	0.03434768
SLC22A17	0.033232019	0.836828005	0.70926402	0.987334885	0.034771404
ECE2	0.024829003	1.271083468	1.017293218	1.588188297	0.034785835
FLI1	0.009818162	0.777658772	0.613065627	0.986441156	0.038221262
FYN	0.049419978	0.811923559	0.665774867	0.990154327	0.039620574
FAM129A	0.008783242	0.856453441	0.737727304	0.994286768	0.041828939

NINJ1	0.018987155	0.796490929	0.635762401	0.997853599	0.04785556

Table S3. Candidate research for comparison to our signature.

Authors	Published online date	PubMed ID	Signature found	Gene-signature composition	Equation
Zhong, Haihui, et al.	2021 Sep 1	34540825	9-gene signature	CBFA2T3<br>CR2<br>SEL1L3<br>TM6SF1<br>TSPAN32<br>ITGA6<br>MAPK11<br>RASA3<br>TLR6	risk score = CBFA2T3 * (-0.283625763) + CR2 * (-0.162111327) + SEL1L3 * (-0.231743871) + TM6SF1 * (-0.527168072) + TSPAN32 * (-0.614785144) + ITGA6 * (-0.169271203) + MAPK11 * 0.528539595 + RASA3 * 0.323227079 + TLR6 * 0.3453755
Xu, Qian, et al.	2021 Jul 2	34277626	6-gene signature	APOC3<br>EPOR<br>H2AFX<br>MXD1<br>PLCG2<br>YWHAZ	risk score = APOC3 × 0.043262193 + EPOR × (-0.056851922) + H2AFX × 0.008373958 + MXD1 × 0.043730173 + PLCG2 × (-0.095876138) + YWHAZ × 0.003148576
Cheng, Yang, et al.	2021 Jun 24	34249701	5-gene signature	KIF20A<br>KLF4<br>KRT6A<br>LIFR<br>RGS13	risk score = KIF20A * 0.3013 + KLF4 * 0.2402 + KRT6A * 0.0859 + LIFR * (-0.1823) + RGS13 * (-1.100)

Zhang, Anran, et al.	2021 Jun 24	34249899	15-gene signature	RELA<br>ACSL3<br>YWHAE<br>EIF2S1<br>CISD1<br>DDIT4<br>RRM2<br>PANX1<br>TLR4<br>ARNTL<br>LPIN1<br>HERPUD1<br>NCOA4<br>PEBP1<br>GLS2	risk score = RELA * 0.31121564 + ACSL3 * 0.292079905 + YWHAE * 0.160823922 +EIF2S1 * 0.159470859 + CISD1 * 0.156274215 + DDIT4 * 0.079830296 + RRM2 * 0.043449128 + PANX1 * 0.013029525 + TLR4 * (-0.010188709) + ARNTL * (-0.052913519) + LPIN1 * (-0.179354454) + HERPUD1 * (-0.213560347) + NCOA4 * (-0.253508649) + PEBP1 * (-0.262091825) + GLS2 * (-0.267566406)
Chen, Fangyu, et al.	2021 Jun 4	34150632	8-gene signature	ACVR1B<br>BIRC5<br>NR2E1<br>INSR<br>TGFA<br>BMP7<br>CD28<br>NUDT6	risk score = ACVR1B * (-0.1831) + BIRC5 * 0.1300 + NR2E1 * 0.1798 + INSR * (-0.2200) + TGFA * 0.1697 + BMP7 * 0.1577 + CD28 * (-0.2649) + NUDT6 * 0.2473

Jiang, Wei, et al.	2021 Mar 18	33869220	10-gene signature	PKMYT1<br>ETF1<br>ECT2<br>BUB1B<br>RECQL4<br>TFRC<br>COCH<br>TUBB2B<br>PITX1<br>CDC6	risk score = PKMYT1 * (-0.212) + ETF1 * (-0.655) + ECT2 * 0.22 + BUB1B * 0.178 + RECQL4 * 0.099 + TFRC * 0.106 + COCH * 0.042 + TUBB2B * (-0.119) + PITX1 * 0.094 + CDC6 * 0.122
Wang, Yan, et al.	2021 Feb 8	33628734	4-gene signature	CD69<br>KLRB1<br>PLCB2<br>P2RY13	risk score = CD69 * (-0.03437) + KLRB1 * (-0.06325) + PLCB2 * (-0.0434) + P2RY13 * (-0.11558)
Zhang, Yuan, et al.	2020 Oct 27	33194633	3-gene signature	RELA<br>CYCS<br>FADD	risk score = RELA * 5.0637 + CYCS * 2.5219 + FADD * 0.0003

Table S4. The Spearman coefficient assessed the correlations of risk score and the 22 TICs.

TIC	r	p-value
T cells CD4 memory resting	-0.32388583	1.13E-13
Mast cells resting	-0.32271729	1.39E-13
Dendritic cells resting	-0.24035849	5.30E-08
Monocytes	-0.19706697	9.03E-06
Plasma cells	-0.12625893	0.00469141
T cells regulatory (Tregs)	-0.11871295	0.00787785
B cells memory	-0.11712731	0.00875386
B cells naive	-0.06761287	0.13109094
T cells gamma delta	-0.02993084	0.50429419
Macrophages M2	0.017053316	0.70355433
T cells CD4 naive	0.027845874	0.53445835
Eosinophils	0.050938853	0.25557393
NK cells activated	0.05338664	0.23340885
Macrophages M1	0.08369091	0.06148611
Dendritic cells activated	0.094668955	0.03431769
NK cells resting	0.097345223	0.02952323
T cells CD8	0.122005832	0.00633263
T cells follicular helper	0.146199073	0.00104338
Neutrophils	0.168647316	0.00015138
Mast cells activated	0.212236759	1.68E-06
T cells CD4 memory activated	0.229710015	2.07E-07
Macrophages M0	0.340625248	4.76E-15

Table S5. The Kaplan–Meier curves measured the prognostic abilities of the 22 TICs.

TIC	P-value
B cells naive	0.73195573
B cells memory	0.14526478
Plasma cells	0.07168164
T cells CD8	0.94945777
T cells CD4 naive	0.15157197
T cells CD4 memory resting	0.98342753
T cells CD4 memory activated	0.06333732
T cells follicular helper	0.61289909
T cells regulatory (Tregs)	0.57035866
T cells gamma delta	0.87726184
NK cells resting	0.42005935
NK cells activated	0.65030923
Monocytes	0.34777235
Macrophages M0	0.15773795
Macrophages M1	0.07390928
Macrophages M2	0.25109349
Dendritic cells resting	0.04434603
Dendritic cells activated	0.67117819
Mast cells resting	0.0001638
Mast cells activated	0.00810307
Eosinophils	0.60211224
Neutrophils	0.9221555
